# Associations between maternal psychological distress and mother-infant bonding: a systematic review and meta-analysis

**DOI:** 10.1007/s00737-023-01332-1

**Published:** 2023-06-15

**Authors:** Gypsy A. O’Dea, George J. Youssef, Lauryn J. Hagg, Lauren M. Francis, Elizabeth A. Spry, Larissa Rossen, Imogene Smith, Samantha J. Teague, Kayla Mansour, Anna Booth, Sasha Davies, Delyse Hutchinson, Jacqui A. Macdonald

**Affiliations:** 1 School of Psychology, Centre for Social and Emotional Early Development, Deakin University, Geelong, Australia; 2 Centre for Adolescent Health, Murdoch Children’s Research Institute, Melbourne, Australia; 3 Department of Paediatrics, University of Melbourne, Parkville, Australia; 4 Counselling Psychology Department, Trinity Western University, Langley Township, BC Canada; 5 Division of Tropical Health and Medicine, Department of Psychology, College of Healthcare Sciences, James Cook University, Townsville, Australia; 6 La Trobe University, School of Psychology and Public Health, The Bouverie Centre, Brunswick, Australia; 7 Faculty of Psychology, Counselling, and Psychotherapy, The Cairnmillar Institute, Hawthorn East, Australia; 8 National Drug and Alcohol Research Centre, University of New South Wales, Sydney, Australia

**Keywords:** Maternal-infant bonding, Perinatal, Mental health, Systematic review, Meta-analysis

## Abstract

**Purpose:**

Maternal psychological distress and mother-infant bonding problems each predict poorer offspring outcomes. They are also related to each other, yet the extensive literature reporting their association has not been meta-analysed.

**Methods:**

We searched MEDLINE, PsycINFO, CINAHL, Embase, ProQuest DTG, and OATD for English-language peer-reviewed and grey literature reporting an association between mother-infant bonding, and multiple indicators of maternal psychological distress.

**Results:**

We included 133 studies representing 118 samples; 99 samples (110,968 mothers) were eligible for meta-analysis. Results showed concurrent associations across a range of timepoints during the first year postpartum, between bonding problems and depression (*r* = .27 [95% CI 0.20, 0.35] to *r* = .47 [95% CI 0.41, 0.53]), anxiety (*r* = .27 [95% CI 0.24, 0.31] to *r* = .39 [95% CI 0.15, 0.59]), and stress (*r* = .46 [95% CI 0.40, 0.52]). Associations between antenatal distress and subsequent postpartum bonding problems were mostly weaker and with wider confidence intervals: depression (*r* = .20 [95% CI 0.14, 0.50] to *r* = .25 [95% CI 0.64, 0.85]), anxiety (*r* = .16 [95% CI 0.10, 0.22]), and stress (*r* = .15 [95% CI − 0.67, 0.80]). Pre-conception depression and anxiety were associated with postpartum bonding problems (*r* = − 0.17 [95% CI − 0.22, − 0.11]).

**Conclusion:**

Maternal psychological distress is associated with postpartum mother-infant bonding problems. Co-occurrence of psychological distress and bonding problems is common, but should not be assumed. There may be benefit in augmenting existing perinatal screening programs with well-validated mother-infant bonding measures.

**Supplementary Information:**

The online version contains supplementary material available at 10.1007/s00737-023-01332-1.

## Introduction

Maternal psychological distress is common in the perinatal period and is linked to adverse outcomes for both mothers and offspring (Rogers et al., 2020). Prevalence estimates are as high as 19% for perinatal depression (Howard et al., 2014), and 13% for perinatal anxiety (Howard et al., 2014). Prevalence estimates of maternal stress range from 12% in pregnancy (Kingston et al., 2012) to 8% for postpartum stress (Bener et al., 2012 ). Approximately 10–25% of postpartum women referred to psychiatric services present with concurrent mother-infant bonding difficulties (Brockington, 1996; Brockington et al., 2006). In addition, maternal depression, anxiety, and stress, identified during pregnancy, have been linked with poorer mother-infant bonding early in the postpartum period (Kokubu et al., 2012; Rossen et al., 2016), and also at 12 months postpartum (Le Bas et al., 2021). Comorbidity of maternal psychological distress and bonding problems may compound risks to mother and infant wellbeing; yet a meta-analysis determining the extent to which they are related has not been undertaken.

The maternal bond represents the mother’s emotional response to her infant (Condon and Corkindale, 1998). Impaired bonding can manifest as maternal emotional ambivalence, anger, and in extreme cases, a heightened risk of infant neglect, abuse, or rejection (Brockington, 2004). Given that there are no formal diagnostic criteria defining the presence or severity of maternal-infant bonding problems, definitions, cut-off scores, and assessment methods of impaired bonding vary widely across studies. As a result, prevalence rates are challenging to estimate. Nonetheless, the reported estimates are cause for consideration and monitoring in mother-infant perinatal care. Delayed onset of mother-infant bonding is common after childbirth, but usually transient (Yoshida et al., 2012). Prevalence estimates of mild or moderate bonding problems range from 3 to 22% in community samples using self-report instruments (Edhborg et al., 2011; Garcia-Esteve et al., 2016; Macdonald et al., 2018; O'Higgins et al., 2013; Reck et al., 2006; Taylor et al., 2005; Vengadavaradan et al., 2019), and as high as 24% (Vengadavaradan et al., 2019) using the criterion standard psychiatrist administered Stafford Interview (Brockington et al., 2017b). These studies included samples from both low- and high-income countries, and varied in the timing of assessment, from the first few days after birth to 12 months postpartum. Estimates of severely impaired bonding in community samples range from 0.6 to 4% (Edhborg et al., 2011; Figueiredo et al., 2007; Garcia-Esteve et al., 2016) using self-report instruments, and up to 11% (Vengadavaradan et al., 2019) using the Stafford Interview. Prevalence of mother-infant bonding problems in populations of mothers with concurrent psychiatric disorders are generally higher than in community samples. For example, Brockington (1996) reported bonding problems in 10% to 25% of mothers referred postnatally for psychiatric care. More recently, rates as high as 45% were reported in a small sample (*n* = 31) of Indian mothers with a lifetime history of psychiatric conditions which included psychosis, bipolar affective disorder, depressive and anxiety disorders, and adjustment disorder (Vengadavaradan et al., 2019). Estimates of bonding problems in mothers with concurrent depression are as high as 24% (O'Higgins et al., 2013). Other populations may also have specific vulnerabilities, for example 12% of mothers of infants in NICU experience difficulties bonding with their infants (Bienfait et al., 2011).

Reduced maternal bonding is associated with poorer infant outcomes, including less secure attachment, difficult temperament, higher colic ratings, and less positive infant mood (Le Bas et al., 2020). Without intervention, poor bonding is likely to persist beyond the first year postpartum (de Cock et al., 2016). Correspondingly, perinatal depression and anxiety are associated with poorer offspring outcomes spanning from infancy through to adolescence, across mental health (Srinivasan et al., 2020), cognitive, language, motor, and adaptive behaviour domains (Rogers et al., 2020).

Three prior narrative reviews (Edwards, 2017; McNamara et al., 2019; Tichelman et al., 2019) reported that maternal depression, anxiety, and stress symptoms were associated with mother-infant bonding problems, however, no meta-analysis of these associations was conducted. Further, these reviews did not examine links between pre-conception mental health and postpartum bonding. Emerging evidence points to origins as early as adolescence of both perinatal psychological distress and mother-infant bonding problems (Macdonald et al., 2018; Patton et al., 2015). A synthesis of the literature reporting on prospective, longitudinal associations between women’s history of pre-birth psychological distress and postpartum bonding problems may inform early identification of postpartum risk, which may in turn increase intergenerational risk for offspring development (Le Bas et al., 2020).

Extending upon extant literature, we conducted a systematic, meta-analytic review examining the associations between maternal psychological distress and postpartum mother-infant bonding. This study aimed to (1) assess the strength of associations between common domains of maternal psychological distress and mother-infant bonding problems, (2) examine whether effect sizes vary as a function of (a) psychological distress domain or (b) timing in the postpartum period, and (3) examine longitudinal relationships between historic and antenatal psychological distress and postpartum bonding.

## Method

### Search strategy

This review was conducted in accordance with the Preferred Reporting Items for Systematic Reviews and Meta-Analyses (PRISMA) guidelines (Moher et al., 2009) and MOOSE Reporting Guidelines for Meta-analyses of Observational Studies (Brooke et al., 2021). PROSPERO registration: CRD42018107218. We searched MEDLINE, PsycINFO, CINAHL, Embase, ProQuest DTG, and OATD databases for peer reviewed and grey literature published up to 9 October 2020. Broad and inclusive search terms were developed for four concepts: *maternal*, *bonding*, *psychological distress*, and *postpartum* (eTable 1). The search included free text terms and subject headings to allow for retrieval of relevant records regardless of the words used in titles, abstracts, or key words. Searches were limited to human populations and records available in English. Reference lists of relevant reviews and included studies were also searched. Authors were systematically contacted to request unadjusted coefficients of association where not reported, and to request data from identified unpublished studies. Of 34 authors contacted, 7 supplied the requested data, 3 advised data was not available, and 24 authors did not respond.

### Inclusion criteria

Eligibility criteria were (1) included a standardised self-report measure of mother-infant bonding between birth and 12 months postpartum; (2) reported on an association between bonding and depression, anxiety, stress, psychological distress, or postpartum blues. Grey literature was included to reduce potential publication bias. Reviews, qualitative studies, case reports, or intervention studies not reporting data separately for a control group were excluded. Retrieved records were independently double screened for eligibility by two blinded study authors at title and abstract level using Covidence (Veritas Health Innovation, n.d.). At full text level records were independently reviewed by [GO] and a second blinded study author (LH, LR, or KM). Screening conflicts were resolved by an independent senior reviewer (GO, DH or JM), and at full text review by discussion and consensus.

### Measurement of maternal-infant bonding

This review defined mother-infant bonding in accordance with Condon (1998) and Kinsey and Hupcey (2014) as the mother’s perceived emotional connection to her infant. Eighty-two measures potentially assessing the mother-infant bond in retrieved studies were assessed (by GO, JM, and DH) to determine whether they assessed the mother’s felt, emotional bond to her infant. Of these, 13 were excluded because they were an observer rated measure, 49 measured a construct other than bonding, and four were excluded because they measured the maternal-fetal bond during pregnancy. Sixteen eligible measures of self-reported maternal bonding were identified, and of these, 14 were included in this review (some studies were excluded for reasons not related to the bonding measure).

### Data analysis

Data were extracted by GO for consistency and verified by LF. Extracted data included study characteristics, bonding and psychological distress measures, time of data collection, sample size, sample characteristics, and effect size coefficients. For meta-analysis, we extracted unadjusted correlation coefficients as a measure of effect size, or these were provided upon request by authors. When a correlation coefficient was not reported, group mean differences, odds ratios, and chi-square coefficients were converted to correlation coefficients (*r*) for analysis. Mother-infant bonding instruments are scaled such that high scores may represent either optimal or poor bonding; where applicable, we reversed the direction of reported coefficients to ensure alignment. Where multiple studies reporting on the same sample duplicated a specific analysis, we retained the effect estimate with the largest sample size. Effects utilising subscales of bonding measures were included only where effects derived from total scores were not reported. Risk of bias of included studies was independently assessed by two authors (GO and either LR or LHs) using a 10-point quality assessment tool, adapted from the National Institutes of Health (NIH) Quality Assessment Tool for Observational Cohort and Cross-sectional Studies (NHLBI, 2018). Conflicts were resolved by consensus agreement. NHLBI quality assessment tools aim to assist reviewers in evaluating concepts indicative of a study’s internal validity, and are widely used to evaluate the quality of studies in review processes informing the NIH clinical health guidelines (NHLBI, 2018). The tool was adapted for use in accordance with the NIH instructions, and a full description of items assessed and criteria for ratings can be found in the supplementary (eTable 10).

A series of random effects meta-analyses was conducted to estimate the pooled associations between maternal psychological distress and mother-infant bonding problems, using R software, version 4.0.5 (R Core Team, 2021). An overall meta-analysis of all included effect sizes was conducted, and data were also stratified by psychological distress domain (depression, anxiety, stress, blues) and by bonding timepoint across the postpartum period (birth to 1 week; > 1 week to < 3 months; 3 to < 6 months; 6 to 12 months). We also meta-analysed associations of preconception and antenatal psychological distress and postpartum bonding problems. Meta-analyses were conducted if at least two independent effects were available. When a meta-analysis comprised clustered effects, we used a robust variance meta-analysis approach utilising the robumeta package, version 2.0 (Fisher et al., 2017). When a meta-analysis included only independent effect sizes, we used the Metafor package, version 2.4.0 (Viechtbauer, 2010).

Meta-regressions were conducted to explore whether associations between psychological distress and bonding were moderated by domains of psychological distress, sample type (e.g. clinical, community), parity, assessment timepoint, country income classification, or publication type. Pairwise comparisons were also conducted where relevant. Levels of a moderator were only included in moderation analyses when at least two independent effects were available. When a meta-regression used a moderator with at least three levels, a Wald chi-square test of the overall moderation was conducted using the clubSandwich package, version 0.5.3 (Pustejovsky, 2021). Meta-analytic effect sizes were interpreted according to guidelines whereby *r* = .1 is considered *weak*, *r* = .2 is *moderate*, and *r* = .3 is *strong* (Gignac and Szodorai, 2016). We used the *I*^*2*^ and *tau*^*2*^ statistics as estimates of heterogeneity. Potential publication bias was assessed by three methods using the Metafor package (Viechtbauer, 2010): Egger’s test of funnel plot asymmetry, visual inspection of funnel plots, and stratified analyses by publication type (peer-reviewed or grey literature) across psychological distress domains.

## Results

**Fig. 1:**
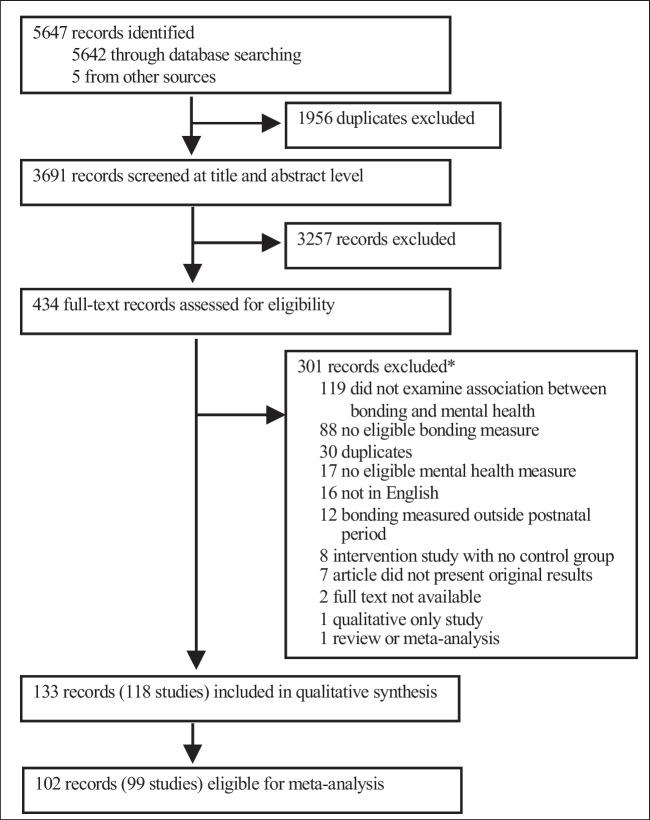
PRISMA diagram. *Note:* *primary reason for exclusion reported if multiple criteria applied to article. Records excluded at full text level, with reason for exclusion, are in supplementary eTable 3. The protocol for this review was registered in 2018 and the review commenced the same year, thus the design followed PRISMA 2009 guidelines that were current at that time

Our systematic search identified 5647 records. After duplicates were removed, 3691 were screened, and 434 full-text records were assessed for eligibility. A total of 133 records, including journal articles, doctoral theses/dissertations, and conference abstracts, met eligibility criteria. These 133 records reported on 118 discrete samples, comprising 119,498 mothers. Of these, 102 records, reporting on 99 study samples (110,968 mothers), were eligible for meta-analysis (see Fig. 1). Study characteristics and articles (records) excluded at full text are presented in the supplement (eTables 2 and 3). Included articles reported on four domains of maternal psychological distress: depression, anxiety, stress, and postpartum blues, assessed by 43 mental health measures. The Edinburgh Postnatal Depression Scale was the most commonly utilised instrument overall, and of depression, employed by 88 study samples (reported in 96 articles). The State Trait Anxiety Scale was the most commonly used anxiety measure, employed by 12 study samples (in 13 articles). No studies reporting on the construct of “psychological distress”, for example as measured by the Kessler Psychological Distress Scale (Kessler et al. 2002), were eligible for meta-analysis, and thus the term psychological distress is used herein as an umbrella term referring to the four domains stated above. Fourteen self-reported mother-infant bonding instruments were utilised; the PBQ (used by 67 studies across 71 articles) and the MPAS (21 studies in 24 articles) were the most commonly used bonding instruments. Included studies recruited participants from 20 high-income countries (HIC), 6 upper-middle income countries (UMIC), and 2 low-middle income countries (LMIC). Included articles were published between 1988 and 2020, with 111 (83%) published since 2011 (eTable 4).

Table 1 shows the meta-analytic associations between increased maternal psychological distress and impaired mother-infant bonding, overall and stratified by distress domain. Overall, a strong association was observed. Among specific domains, the strongest association with bonding was observed for depression (*r* = .39), with moderate to strong associations observed for anxiety, stress, and postpartum blues.

**Table 1:** Meta-analytic effects of associations between psychological distress and mother-infant bonding problems

Psychological distress domain^a^	*k*^b^	*n*^*c*^	N^d^	*r (*95% CI)	*I*^2^	Tau^2^
All	99	368	110,968	0.38 (0.34, 0.41)	97.30	0.08
Depression	94	234	109,957	0.39 (0.35, 0.42)	97.46	0.08
Anxiety	26	74	8335	0.26 (0.22, 0.31)	82.74	0.02
Stress	5	19	2068	0.24 (0.09, 0.38)	88.48	0.04
Blues	3	9	339	0.23 (− 0.11, 0.52)	63.06	0.02
Depression>Anxiety***, Depression>Stress*						

^a^ measured at any timepoint (i.e. preconception, antenatal, or postpartum)

^b^*k* = number of studies

^c^*n* = number of effects

^d^*N* = combined number of participants in included studies

*p < .05

**p < .01

***p < .001

Meta-regression analyses (eTable 5) indicated that these effects were robust across sample type (e.g. clinical, community), parity, income classification of country, and publication type (e.g. peer reviewed or grey literature), and for anxiety and bonding the effects were robust across all bonding measures used. However, for associations between impaired mother-infant bonding and overall maternal psychological distress, and between impaired bonding and depression, Wald chi-square test results revealed the strength of association was moderated by the bonding measure used. Pairwise comparisons revealed that the association between bonding utilising the Mother Infant Bonding Scale (MIBS; Taylor et al., 2005) and overall psychological distress was *r* = 0.25 [95% CI 0.2, 0.31], and with depression was *r* = 0.27 [95% CI 0.21, 0.33]. These were significantly smaller than associations between bonding assessed using the Maternal Postnatal Attachment Scale (MPAS; Condon and Corkindale, 1998; overall *r* = 0.42 [95% CI 0.32, 0.52]; depression *r* = 0.43 [95% CI 0.34, 0.53]), and the Postpartum Bonding Questionnaire (PBQ; Brockington et al. 2006; overall *r* = 0.43 [95% CI 0.38, 0.49]; depression *r* = 0.45 [95% CI 0.39, 0.51]). Moderation analyses of bonding measures used were not able to be conducted for associations with stress or blues, due to insufficient independent effects for analysis. Pairwise comparisons revealed a stronger correlation between depression and bonding than for anxiety or stress and bonding (see Table 1). Forest plots and funnel plots for all meta-analyses are in the Supplement (eTables 6–8, eFigures 1–19).

Table 2 shows cross-sectional associations between postpartum psychological distress and bonding problems, stratified by postpartum period. A majority of studies examined the relationship between bonding problems and depression (*n* = 94), with fewer examining relationships with anxiety (*n* = 26), stress (*n* = 5) or postpartum blues (*n* = 3). Overall, associations between postpartum psychological distress and mother-infant bonding problems were moderate to very strong (meta-analytic effect sizes ranging from *r* = .25 to *r* = .47). As shown in Table 2, meta-regression analyses indicated that the association between depression and bonding (*p* = .0021) was moderated by the timing of assessment, with the association during the first week smaller than associations for the rest of the postpartum year; however, timing did not moderate associations between either anxiety or stress and bonding.

**Table 2:** Meta-analytic cross-sectional associations between domains of psychological distress and mother-infant bonding problems

	*k*^a^	*n*^b^	*N*^c^	*r* (95% CI)	*I*^2^	Tau^2^
Depression						
Time 1 (birth to 1 week)	10	22	2970	0.27 (0.20, 0.35)	72.84	0.01
Time 2 (> 1 week to < 3 months)	39	63	12 416	0.41 (0.37, 0.46)	88.41	0.03
Time 3 (3 months to < 6 months)	21	29	4799	0.47 (0.41, 0.53)	76.96	0.02
Time 4 (6 months to 12 months)	15	21	6493	0.42 (0.35, 0.48)	87.8	0.02
Model Wald *χ*^2^ test: *p* = .0021: Time 1 < Time 2**, Time 3***, Time 4*; Time 3 > Time 2*						
Anxiety						
Time 1 (birth to 1 week)	NA	NA	NA	NA	NA	NA
Time 2 (> 1 week to < 3 months)	11	17	5658	0.31 (0.25, 0.36)	78.43	0.01
Time 3 (3 months to < 6 months)	4	7	723	0.39 (0.15, 0.59)	81.58	0.03
Time 4 (6 months to 12 months)	3	3	2402	0.27 (0.24, 0.31)	0.23	0.00
Model Wald *χ*^2^ test: *p* = 0.598						
Stress						
Time 1 (birth to 1 week)	NA	NA	NA	NA	NA	NA
Time 2 (> 1 week to < 3 months)	2	2	1802	0.28 (-0.17, 0.64)	97.68	0.11
Time 3 (3 months to < 6 months)	NA	NA	NA	NA	NA	NA
Time 4 (6 months to 12 months)	2	2	1616	0.46 (0.40, 0.52)	5.62	0.0009
Model Wald *χ*^2^ test *p* = 0.887						
Postpartum blues						
Birth to 1 week postpartum	3	5	339	0.25 (− 0.10, 0.55)	60.9	0.01

*NA* = not applicable, insufficient studies to conduct meta-analysis

^a^*k* = number of studies

^b^*n* = number of effects

^c^*N* = combined number of participants in included studies

*p < .05

**p < .01

***p < .001

Table 3 shows associations between antenatal psychological distress and postpartum impaired bonding, stratified by postpartum period. Associations between depression and bonding problems were moderate, and weak-to-moderate for anxiety and bonding. Weak-to-moderate associations for stress and bonding were derived from only two studies and show wide confidence intervals, sometimes crossing zero.

**Table 3:** Meta-analytic longitudinal associations between domains of psychological distress in pregnancy and postpartum mother-infant bonding problems

	*k*^a^	*n*^b^	*N*^c^	*r* (95% CI)	*I*^2^	Tau^2^
Depression in pregnancy						
Impaired bonding						
Time 1 (birth to 1 week)	3	6	1073	0.20 (0.14, 0.50)	79.27	< 0.01
Time 2 (> 1 week to < 3 months)	15	22	4964	0.21 (0.16, 0.27)	62.04	< 0.02
Time 3 (3 months to < 6 months)	2	5	497	0.25 (− 0.64, 0.85)	57.78	0.01
Time 4 (6 months to 12 months)	2	5	2669	0.25 (− 0.04, 0.49)	49.35	< 0.01
Model Wald *χ*^2^ test *p* = 0.581						
Anxiety in pregnancy						
Impaired bonding						
Time 1 (birth to 1 week)	3	7	1073	0.16 (− 0.08, 0.38)	48.95	< 0.01
Time 2 (> 1 week to < 3 months)	10	16	4154	0.16 (0.10, 0.22)	59.56	< 0.01
Time 3 (3 months to < 6 months)	NA	NA	NA	NA	NA	NA
Time 4 (6 months to 12 months)	NA	NA	NA	NA	NA	NA
Model Wald *χ*^2^ test *p* = 0.38						
Stress in pregnancy						
Impaired bonding						
Time 1 (birth to 1 week)	NA	NA	NA	NA	NA	NA
Time 2 (> 1 week to < 3 months)	2	4	1802	0.15 (− 0.67, 0.80)	81.46	0.01
Time 3 (3 months to < 6 months)	NA	NA	NA	NA	NA	NA
Time 4 (6 months to 12 months)	NA	NA	NA	NA	NA	NA

*NA* = not applicable, insufficient studies to conduct meta-analysis

^a^*k* = number of studies

^b^*n* = number of effects

^c^*N* = combined number of participants in included studies

The next group of meta-analyses involved effect sizes from two studies (Macdonald et al., 2022; Olsson et al., 2020) relating to maternal depression and anxiety measured across adolescence (13 to 17 years of age), and young adulthood (18 to 29 years of age). We meta-analysed group differences across four groups: symptoms of anxiety and/or depression (1) in adolescence, (2) in young adulthood, (3) persisting across adolescence and young adulthood, and (4) no symptoms of anxiety/depression. For these analyses of multiple group difference scores, we calculated Cohen’s *d* as the effect size allowing differences to be examined in standard deviation units. We also report a conversion to Pearson’s *r* for comparison with antenatal and postnatal effects using formulas in Ruscio (2008). Results showed that women who reported persistent depressive and/or anxiety symptoms across adolescence and young adulthood also reported poorer subsequent mother-infant bonding compared to mothers without prior psychological distress, with differences of one-third of a standard deviation (*d* = − 0.34 [95% CI − 0.44, − 0.23]; *r* = − 0.17 [95% CI − 0.22, − 0.11]). Smaller non-significant differences in bonding scores were observed for mothers reporting depression and/or anxiety only in adolescence (*d* = − 0.14 [95% CI − 0.41, 0.13]; *r* = − 0.07 [95% CI − 0.20, 0.07]), or only in young adulthood (*d* = − 0.25 [95% CI − 0.99, 0.49]; *r* = − 0.12 [95% CI − 0.44, 0.24), compared with mothers without prior psychological distress.

Risk of bias of included studies is in the Supplement (eTables 9–10). More than 90% of studies reported a clear research question, used valid and reliable assessment measures, and more than 85% used continuous data. Only 39 studies (29%) reported a recruitment rate greater than 50% of eligible participants, and only 18 longitudinal studies (19%) reported an attrition rate < 20%. No evidence of publication bias in this meta-analysis was indicated (see eTables 11–12, eFigures 20–24). With respect to the Funnel plots (see eFigures 20–24 in supplementary), while these figures showed symmetry, we note that effect sizes did not converge into the anticipated triangular shape, suggesting some variability between studies, even amongst those with large sample sizes and low standard errors.

Notably, based on reviewer recommendation, we conducted an informal update of our search on 19 March 2023, and found that the new data retrieved was entirely consistent with the direction and magnitude of meta-analytic effect sizes presented in this study. Interested readers can access this updated dataset elsewhere: https://osf.io/up8wq/?view_only=c6472b0e4c5a47a98bd7a788ca28370f.

## Discussion

This meta-analysis synthesises the extensive literature on the relationship between maternal psychological distress and mother-infant bonding problems, providing systematic evidence that maternal symptoms of depression, anxiety, and stress are associated with increased risk of poorer mother-infant bonding. A strong relationship between maternal psychological distress and bonding problems was evident (*r* = .38). Of the 368 included effect sizes, 367 were in the same direction, varying only by magnitude. The strength of associations varied across domains of psychological distress, with the strongest associations found for depression and bonding problems, possibly reflecting the predominant focus on depression in the included literature. The strength of associations between depression and psychological distress with bonding were moderated by the instrument used to assess bonding. Our results did not differ as a function of sample type (e.g. clinical, community), parity, income level of country, or publication type, suggesting these findings may be generalised to inform clinical practice guidelines pertaining to maternal mental health care.

Maternal depression was the predominant focus of the included literature, yielding 234 effects sizes for meta-analysis. For postpartum depression, 113 effect sizes were included, sufficient to confidently conclude a strong relationship (*r* = .41 to .47) after the first postpartum week. This relationship may be a function of affective features underlying both postpartum depression and mother-infant bond formation. The subjective bond represents a mother’s feelings of love and affection toward her infant (Condon and Corkindale, 1998). Formation of the bond is a reward-driven process, theoretically underpinned by a dynamic exchange of physiological and behavioural cues between mother and infant (Feldman, 2012; Nephew et al., 2015). This process is disrupted by features of depression such as anhedonia, social withdrawal, and reward deficits associated with reduced oxytocin (Nephew et al., 2015); thus depressed mothers may have less desire to receive or engage with affiliative bonding cues, and experience less pleasure and reward when they do (Vliegen et al., 2009). The weaker associations observed in the first postpartum week may be explained by evidence suggesting the formation of the mother-infant bond is a dynamic process that, for some mothers, is not yet established during the first postpartum week (Yoshida et al., 2012). Similarly, assessment of maternal depression in the first postpartum week may be confounded by symptoms of postpartum blues. Postpartum blues is a high prevalence, transient phenomenon, thought to be caused by hormonal changes after delivery, affecting up to half of new mothers in the first week postpartum (Bass III and Bauer, 2018; Rezaie-Keikhaie et al., 2020). Overlap of items measuring postpartum depression and postpartum blues is observed in commonly used assessment tools, such as the Kennerley Blues Scale (Kennerley and Gath, 1989) and the Edinburgh Postnatal Depression Scale (Cox et al., 1987).

Moderation analyses revealed that associations between maternal depression and bonding, and psychological distress and bonding, were smaller when bonding was assessed using the MIBS (Taylor et al., 2005) compared to the MPAS (Condon and Corkindale, 1998) or the PBQ (Brockington et al., 2006; Brockington et al., 2001). This may be explained by differences between these measures. The MIBS consists of eight adjectives only (e.g. loving, resentful, joyful) which respondents answer on a four-point Likert scale (“not at all” to “very much”) indicating how they felt about their infant “in the first few weeks”. By contrast, the MPAS is a 19-item scale, with Likert response options assessing frequency, intensity, and nature of emotions and cognitions relating to the mother’s subjective emotional bond to her infant. The PBQ is similarly nuanced, comprising 25-items, all assessed using a six-point Likert scale of frequency reflecting elements of emotions, behaviours, and cognitions relating to the infant. Moreover, prior literature (van Bussel et al., 2010) demonstrates strong correlations between the MPAS and the PBQ (*r* = .63 to .67), yet slightly weaker correlations between the MIBS and the MPAS (*r* = .45 to .50) and the MIBS and the PBQ (*r* = .56 to .60). Taken together, this suggests that the MIBS may be assessing different facets of mother-infant bonding than the MPAS or PBQ, which may then correlate less strongly with maternal depression or psychological distress.

Postpartum anxiety was robustly associated with mother-infant bonding. Given high prevalence rates of perinatal anxiety and effects on infant development, the smaller body of literature highlights the need for further research. It is worth noting that postpartum anxiety can manifest as either over-involvement with, or a rejection of, the infant (Brockington, 1996). Anxious-over-attentive mothers may be more likely to endorse bonding assessment items indicating a strong desire for affective connection and physical and emotional proximity to their infant (Brockington et al., 2006), whereas anxious-rejecting mothers would typically be less likely to endorse these items. Taken together, some level of anxiety may be associated with a *better* bond in some mothers and a *poorer* bond in others. However, for clinical practice, our results suggest that indications of maternal general anxiety or infant-related anxiety are worthy of further assessment and support.

Associations of postpartum stress and bonding were derived from substantially fewer studies than for depression or anxiety and should be interpreted with caution. These results nonetheless provide emerging evidence for maternal stress as a correlate of bonding and indicate further research is needed.

The magnitude of associations between postpartum depression and anxiety with bonding problems support prior reports and theoretical perspectives that, despite some affective overlap, these are distinct co

nstructs, and for many mothers they do not co-occur (Brockington et al., 2006; Le Bas et al., 2021). For example, even for the largest meta-analytic correlation between postpartum depression and bonding problems leaves a substantial proportion of variance unexplained. Accurate identification of bonding problems, as distinct from depression or anxiety, is important in practice, as interventions differ (Brockington et al., 2017a). Interventions for bonding difficulties typically focus on improving and increasing mother-infant interactions, maternal responsiveness to infant cues, and maternal representations of the infant (Holt et al., 2021), whereas perinatal mental health interventions typically focus on cognitive, behavioural, and pharmacological approaches to improving maternal mood. Psychotherapeutic programs aimed at depression are not always effective in improving mother-infant bonding (O'Mahen et al., 2014), however, combined programs specifically targeting both mood and bonding problems have shown some success (Holt et al., 2021). Of interest, repetitive transcranial magnetic stimulation appears effective for both postpartum depressive symptoms and mother-infant bonding (Garcia et al., 2010).

Our findings suggest standardised routine screening of maternal-infant bonding for mothers presenting with depression or anxiety symptoms should be considered. Current clinical practice guidelines in Australia (Austin et al., 2017), the UK (National Institute for Health and Care Excellence, 2014), and the USA (American College of Obstetricians & Gynecologists, 2018), recommend routine screening for perinatal psychological distress, but not for bonding difficulties, via validated self-report measures. While most guidelines support assessment of mother-infant relationships through brief observation or verbal self-report (Austin et al., 2017), this may not be sufficient to detect problems in the affective bond (Brockington et al., 2017a) that are associated with adverse offspring outcomes (Le Bas et al., 2020). Such problems could be identified through administration of a well-validated, brief self-report instrument.

In the longitudinal analyses, antenatal psychological distress domains were associated with a small to moderate increased risk of poor bonding postnatally with wide confidence intervals around some these associations. Few included studies examined bonding outcomes beyond 6 months postpartum, but pooled effects suggest associations between antenatal depression and bonding do not reduce across the first postpartum year. The small number of effect sizes, particularly for antenatal stress, limits the generalisability of the findings. More longitudinal research is needed to clarify associations between psychological distress in pregnancy and postpartum bonding.

Meta-analytic results from two prospective longitudinal studies further demonstrated that persistent preconception depression and/or anxiety during adolescence and young adulthood predict poorer subsequent postpartum bonding. Both studies’ results were attenuated after adjustment for concurrent psychological distress, albeit the effects remained (pooled effect *d* = − 0.34). This novel finding suggests women who report a history of persistent psychological distress may have augmented risk of postpartum bonding problems. Such women could be identified via primary care providers during pregnancy or as early as conception planning. Development of proactive and preventative mental health interventions may reduce the associated risk of subsequent bonding problems and, thus, decrease intergenerational risks. Having only two included studies limits the interpretability of the meta-analytic findings, with more prospective longitudinal studies required.

An important strength of this meta-analytic review is robust effects even after accounting for variation in assessed moderators. No difference was found in effects relative to a country’s income level, although it is notable that 90% of included studies were from HIC, limiting generalisability of these findings. Moreover, given the higher prevalence rates of maternal mental illness (Howard et al., 2014) and impaired bonding (Edhborg et al., 2011; Howard et al., 2014) in LIC, further research in LIC is warranted.

An additional strength of this meta-analysis lies in the substantial number of included studies, particularly recent studies, indicating an increasing interest in this aspect of perinatal care. Further, results were robust to a range of demographic and methodological moderators, highlighting the generalisability of our findings to perinatal care guidelines. A preregistered protocol, open access data, and inclusion of grey literature are important methodological strengths. Some limitations should also be considered. The focus of this review was on unadjusted associations only; future examinations of more complex study designs will enable a richer understanding of related factors and mechanisms that may influence this relationship, informing interventions for dyads at risk. This review did not examine the possibility that impaired mother-infant bonding may impact maternal psychological distress, because included studies predominantly measured psychological distress either prior to, or concurrently with, postpartum bonding. This is an important area for future research. Further, quality assessment of included studies suggested the possibility of some bias due to non-representative samples or attrition. There were also a limited number of studies on anxiety, stress, blues, and preconception exposures, resulting in lower precision for some estimates. Funnel plots also suggest that further examination of study characteristics would be important to explain variability in the strength of effect sizes observed even among studies with large sample size. Our search was also limited to studies published in English, which may have introduced bias to the effect estimates and may limit generalisability (Jackson and Kuriyama, 2019). Nonetheless, a consistent pattern of effects was identified across both high- and low-quality studies, regardless of methodological concerns such as sampling bias.

In conclusion, historic and antenatal symptoms of depression and anxiety indicate an increased risk of postpartum mother-infant bonding problems, and their presence warrants an assessment of the postpartum bonding relationship. In the postpartum period, the presence of either factor may indicate an increased concurrent risk of the other. Both depression and anxiety may disrupt the dynamic biobehavioural synchrony between a mother and her infant that underpins the bonding process. Our findings indicate these are robustly related but distinct constructs. In clinical settings co-occurrence should not be assumed, but should be considered and assessed, and we recommend this be reflected in clinical practice guidelines. Further, our findings suggest opportunities exist for preventative strategies addressing women’s mental health in the transition to pregnancy and parenthood to facilitate a reduction of intergenerational risks associated with bonding problems.

## Supplementary information

ESM 1 eTable 1. Search strategy for MEDLINE Complete via EBSCOHOST (search strategies for other databases available on request). eTable 2. Study charactistics table. eTable 3. Articles excluded at full text. eTable 4. Included articles by year of publication.eTable 5. Meta-regression analyses of various moderators on overall meta-analysis results. eTable 6. Forest plot data for overall meta-analysis of all included effects. eTable 7. Forest plot data meta-analysis of association between depression and bonding. eTable 8. Forest plot data for meta-analysis of association between anxiety and bonding. eFigure 1. Forest plot: Associations between Stress and Bonding. eFigure 2. Forest plot of all associations between postpartum blues and poorer mother-infant bonding. eFigure 3. Forest plot of cross-sectional associations between depression and poorer mother-infant bonding at Time 1 (birth to 1 week). eFigure 4. Forest plot of cross-sectional associations between depression and poorer mother-infant bonding at Time 2 (>1 week to <3 months). eFigure 5. Forest plot of cross-sectional associations between depression and poorer mother-infant bonding at Time 3 (3 months to <6 months). eFigure 6. Forest plot of cross-sectional associations between depression and poorer mother-infant bonding at Time 4 (6 months to 12 months). eFigure 7. Forest plot of cross-sectional associations between anxiety and poorer mother-infant bonding at Time 2 (>1 week to <3 months). eFigure 8. Forest plot of cross-sectional associations between anxiety and poorer mother-infant bonding at Time 3 (3 months to <6months). eFigure 9. Forest plot of cross-sectional associations between anxiety and poorer mother-infant bonding at Time 4 (6 months to 12 months). eFigure 10. Forest plot of cross-sectional associations between stress and poorer mother-infant bonding at Time 2 (>1 week to <3 months). eFigure 11. Forest plot of cross-sectional associations between stress and poorer mother-infant bonding at Time 4 (6 months to 12 months). eFigure 12. Forest plot of cross-sectional associations between stress and poorer mother-infant bonding at Time 1 (birth to 1 week). eFigure 13. Forest plot of longitudinal associations between depression in pregnancy and poorer mother-infant bonding at Time 1 (birth to 1 week). eFigure 14. Forest plot of longitudinal associations between depression in pregnancy and poorer mother-infant bonding at Time 2 (>1 week to <3 months). eFigure 15. Forest plot of longitudinal associations between depression in pregnancy and poorer mother-infant bonding at Time 3 (3 months to <6 months). eFigure 16. Forest plot of longitudinal associations between depression in pregnancy and poorer mother-infant bonding at Time 4 (6 months to 12 months). eFigure 17. Forest plot of longitudinal associations between anxiety in pregnancy and poorer mother-infant bonding at Time 1 (birth to 1 week). eFigure 18. Forest plot of longitudinal associations between anxiety in pregnancy and poorer mother-infant bonding at Time 2 (>1 week to <3 months). eFigure 19. Forest plot of longitudinal associations between stress in pregnancy and poorer mother-infant bonding at Time 2 (>1 week to <3 months). eTable 9. Risk of bias of included studies. eTable 10. Study risk of bias assessment criteria. eTable 11. Egger's test of asymmetry for publication bias. eFigure 20. Funnel plot of effects of associations between depression and postnatal bonding. eFigure 21. Funnel plot of effects of associations between depression and postnatal bonding (outlier removed). eFigure 22. Funnel plot of effects of associations between anxiety and postnatal bonding. eFigure 23. Funnel plot of effects of associations between stress and postnatal bonding. eFigure 24. Funnel plot of effects of associations between postnatal blues and postnatal bonding. eTable 12. Moderation analysis for type of article (published or grey literature). (DOCX 3.60 MB)

## Data Availability

All data used in analyses are available online: https://osf.io/up8wq/?view_only=e173c774560740819d7bcff6f2aa016d
