# Supplementary material for: Associations between maternal psychological distress and mother-infant bonding: a systematic review and meta-analysis
Source: Arch Womens Ment Health. 2023 Jun 15;26(4):441–52. doi: 10.1007/s00737-023-01332-1 (PMC10333415; doi:10.1007/s00737-023-01332-1)
Supplement: Supplementary file 1 — eTable 1. Search strategy for MEDLINE Complete via EBSCOHOST (search strategies for other databases available on request). eTable 2. Study charactistics table. eTable 3. Articles excluded at full text. eTable 4. Included articles by year of publication.eTable 5. Meta-regression analyses of various moderators on overall meta-analysis results. eTable 6. Forest plot data for overall meta-analysis of all included effects. eTable 7. Forest plot data meta-analysis of association between depression and bonding. eTable 8. Forest plot data for meta-analysis of association between anxiety and bonding. eFigure 1. Forest plot: Associations between Stress and Bonding. eFigure 2. Forest plot of all associations between postpartum blues and poorer mother-infant bonding. eFigure 3. Forest plot of cross-sectional associations between depression and poorer mother-infant bonding at Time 1 (birth to 1 week). eFigure 4. Forest plot of cross-sectional associations between depression and poorer mother-infant bonding at Time 2 (>1 week to <3 months). eFigure 5. Forest plot of cross-sectional associations between depression and poorer mother-infant bonding at Time 3 (3 months to <6 months). eFigure 6. Forest plot of cross-sectional associations between depression and poorer mother-infant bonding at Time 4 (6 months to 12 months). eFigure 7. Forest plot of cross-sectional associations between anxiety and poorer mother-infant bonding at Time 2 (>1 week to <3 months). eFigure 8. Forest plot of cross-sectional associations between anxiety and poorer mother-infant bonding at Time 3 (3 months to <6months). eFigure 9. Forest plot of cross-sectional associations between anxiety and poorer mother-infant bonding at Time 4 (6 months to 12 months). eFigure 10. Forest plot of cross-sectional associations between stress and poorer mother-infant bonding at Time 2 (>1 week to <3 months). eFigure 11. Forest plot of cross-sectional associations between stress and poorer mother-infant bonding at Time 4 (6 mon [file 737_2023_1332_MOESM1_ESM.docx]

**Associations Between Maternal Psychological Distress and Mother-Infant Bonding: A Systematic Review and Meta-analysis**

**Supplementary Materials**

Gypsy O’Dea, George J. Youssef, Lauryn J. Hagg, Lauren M. Francis, Elizabeth A. Spry, Larissa Rossen, Sam Teague, Kayla Mansour, Anna Booth, Sasha Davies, Imogene Smith, Delyse Hutchinson*, Jacqui A. Macdonald*.

*Joint senior authors

**Correspondence to**:

Dr Gypsy O’Dea, School of Psychology, Deakin University, Geelong, Victoria, Australia, 3125 Email: [gypsy.odea@research.deakin.edu.au](mailto:gypsy.odea@research.deakin.edu.au) Telephone: +61 3 9246 8164

**Table of Contents**

[eTable 1. Search strategy for Medline Complete via EBSCOHOST (search strategies for other databases available on request). 4](#_Toc99805907)

[eTable 2. Study charactistics table. 5](#_Toc99805908)

[eTable 3. Articles excluded at full text. 21](#_Toc99805909)

[eTable 4. Included articles by year of publication 46](#_Toc99805910)

[eTable 5. Meta-regression analyses of various moderators on overall meta-analysis results. 47](#_Toc99805911)

[eTable 6. Forest plot data for overall meta-analysis of all included effects. 49](#_Toc99805912)

[eTable 7. Forest plot data meta-analysis of association between depression and bonding. 71](#_Toc99805913)

[eTable 8. Forest plot data for meta-analysis of association between anxiety and bonding. 85](#_Toc99805914)

[eFigure 1. Forest plot: Associations between Stress and Bonding. 90](#_Toc99805915)

[eFigure 2. Forest plot of all associations between postpartum blues and poorer mother-infant bonding. 91](#_Toc99805916)

[eFigure 3. Forest plot of cross-sectional associations between depression and poorer mother-infant bonding at Time 1 (birth to 1 week). 92](#_Toc99805917)

[eFigure 4. Forest plot of cross-sectional associations between depression and poorer mother-infant bonding at Time 2 (>1 week to <3 months). 93](#_Toc99805918)

[eFigure 5. Forest plot of cross-sectional associations between depression and poorer mother-infant bonding at Time 3 (3 months to <6 months). 94](#_Toc99805919)

[eFigure 6. Forest plot of cross-sectional associations between depression and poorer mother-infant bonding at Time 4 (6 months to 12 months). 95](#_Toc99805920)

[eFigure 7. Forest plot of cross-sectional associations between anxiety and poorer mother-infant bonding at Time 2 (>1 week to <3 months). 96](#_Toc99805921)

[eFigure 8. Forest plot of cross-sectional associations between anxiety and poorer mother-infant bonding at Time 3 (3 months to <6months). 97](#_Toc99805922)

[eFigure 9. Forest plot of cross-sectional associations between anxiety and poorer mother-infant bonding at Time 4 (6 months to 12 months). 97](#_Toc99805923)

[eFigure 10. Forest plot of cross-sectional associations between stress and poorer mother-infant bonding at Time 2 (>1 week to <3 months). 98](#_Toc99805924)

[eFigure 11. Forest plot of cross-sectional associations between stress and poorer mother-infant bonding at Time 4 (6 months to 12 months). 98](#_Toc99805925)

[eFigure 12. Forest plot of cross-sectional associations between stress and poorer mother-infant bonding at Time 1 (birth to 1 week). 99](#_Toc99805926)

[eFigure 13. Forest plot of longitudinal associations between depression in pregnancy and poorer mother-infant bonding at Time 1 (birth to 1 week). 99](#_Toc99805927)

[eFigure 14. Forest plot of longitudinal associations between depression in pregnancy and poorer mother-infant bonding at Time 2 (>1 week to <3 months). 100](#_Toc99805928)

[eFigure 15. Forest plot of longitudinal associations between depression in pregnancy and poorer mother-infant bonding at Time 3 (3 months to <6 months). 101](#_Toc99805929)

[eFigure 16. Forest plot of longitudinal associations between depression in pregnancy and poorer mother-infant bonding at Time 4 (6 months to 12 months). 101](#_Toc99805930)

[eFigure 17. Forest plot of longitudinal associations between anxiety in pregnancy and poorer mother-infant bonding at Time 1 (birth to 1 week). 102](#_Toc99805931)

[eFigure 18. Forest plot of longitudinal associations between anxiety in pregnancy and poorer mother-infant bonding at Time 2 (>1 week to <3 months). 102](#_Toc99805932)

[eFigure 19. Forest plot of longitudinal associations between stress in pregnancy and poorer mother-infant bonding at Time 2 (>1 week to <3 months). 103](#_Toc99805933)

[eTable 9. Risk of bias of included studies. 104](#_Toc99805934)

[eTable 10. Study risk of bias assessment criteria. 112](#_Toc99805935)

[eTable 11. Egger's test of asymmetry for publication bias. 113](#_Toc99805936)

[eFigure 20. Funnel plot of effects of associations between depression and postnatal bonding. 114](#_Toc99805937)

[eFigure 21. Funnel plot of effects of associations between depression and postnatal bonding (outlier removed). 114](#_Toc99805938)

[eFigure 22. Funnel plot of effects of associations between anxiety and postnatal bonding. 115](#_Toc99805939)

[eFigure 23. Funnel plot of effects of associations between stress and postnatal bonding. 115](#_Toc99805940)

[eFigure 24. Funnel plot of effects of associations between postnatal blues and postnatal bonding. 116](#_Toc99805941)

[eTable 12. Moderation analysis for type of article (published or grey literature). 117](#_Toc99805942)

[eReferences: Reference list of included papers. 118](#_Toc99805943)

eTable 1. Search strategy for Medline Complete via EBSCOHOST (search strategies for other databases available on request).

| S33 | S4 AND S8 AND S17 AND S32 |
| --- | --- |
| S32 | S18 OR S19 OR S20 OR S21 OR S22 OR S23 OR S24 OR S25 OR S26 OR S27 OR S28 OR S29 OR S30 OR S31 |
| S31 | MH "Postpartum Period" OR MH "Peripartum Period" |
| S30 | TI postpartum OR AB postpartum OR SU postpartum |
| S29 | TI "post-partum" OR AB "post-partum" OR SU "post-partum" |
| S28 | TI postnatal OR AB postnatal OR SU postnatal |
| S27 | TI "post-natal" OR AB "post-natal" OR SU "post-natal" |
| S26 | TI perinatal OR AB perinatal OR SU perinatal |
| S25 | TI "peri-natal" OR AB "peri-natal" OR SU "peri-natal" |
| S24 | TI puerperal OR AB puerperal OR SU puerperal |
| S23 | TI puerperium OR AB puerperium OR SU puerperium |
| S22 | TI "after birth" OR AB "after birth" OR SU "after birth" |
| S21 | TI "after childbirth" OR AB "after childbirth" OR SU "after childbirth" |
| S20 | TI "after delivery" OR AB "after delivery" OR SU "after delivery" |
| S19 | TI "new mother*" OR AB "new mother*" OR SU "new mother*" |
| S18 | TI primipar* OR AB primipar* OR SU primipar* |
| S17 | S9 OR S10 OR S11 OR S12 OR S13 OR S14 OR S15 OR S16 |
| S16 | MH "Depression" OR MH "Depressive Disorder" OR MH "Depressive Disorder, Major" OR MH "Depression, Postpartum" OR MH "Anxiety" OR MH "Anxiety Disorders" OR MH "Stress, Psychological" |
| S15 | TI anxiety OR AB anxiety or SU anxiety |
| S14 | TI depress* OR AB depress* OR SU depress* |
| S13 | TI PND OR AB PND OR SU PND |
| S12 | TI PPD OR AB PPD OR SU PPD |
| S11 | TI “psychological distress” OR AB “psychological distress” OR SU “psychological distress” |
| S10 | TI stress OR AB stress OR SU stress |
| S9 | TI "negative affect" OR AB "negative affect" OR SU "negative affect" |
| S8 | S5 OR S6 OR S7 |
| S7 | MH “Mother-Child Relations” |
| S6 | TI attachment OR AB attachment OR SU attachment |
| S5 | TI bond* OR AB bond* OR SU bond* |
| S4 | S1 OR S2 OR S3 |
| S3 | MH "Mothers" OR MH "Maternal Behavior" |
| S2 | TI maternal OR AB maternal OR SU maternal |
| S1 | TI mother* OR AB mother* OR SU mother* |

eTable 2. Study characteristics table.

| **Paper** | **Country (income classification)** | **Sample type** | **Mean maternal age (years)** | **Parity** | **N (analytic)** | **Bonding measure used** | **Infant age at bonding assessment** | **Psychological distress domain**  **(Measure used)** | **Time of psychological distress assessment** | **Included Analysis Design** | **Included in meta-analysis** |
| --- | --- | --- | --- | --- | --- | --- | --- | --- | --- | --- | --- |
| **Agbagwara-Osuji**  **2015**^1^ | UK (HIC) | Community | 33 | Mixed | 223 | MIBS | 1 week  6 weeks | Depression (EPDS)  Anxiety (STAI)  Stress (SLES) | 36 weeks PG  6 weeks PP | L/CS | Y |
| **Badr**  **2018**^2^ | Lebanon (UMIC) | Community | 30.65 | Mixed | 125 | PBQ-German | 10-12 weeks | Depression (EPDS & Self-Reported History) | 1-4 days PP | L | Y |
| **Behrendt**  **2016**^3^ | Germany (HIC) | Clinical & community | Range =  31.58 - 31.79 | Primipara | 38 | MPAS | 6.35 months | Depression (BDI-II) | 6.35 months PP | CS | N |
| **Behrendt**  **2019**^4^ | Germany (HIC) | Community | 31.89 | Primipara | 61 | MPAS | 6.56 months | Depression (BDI-II) | 6.56 months PP | CS | Y |
| **Bicking Kinsey**  **2014**^5^ | USA (HIC) | Community | 27.4 | Primipara | 2995 | PBQ | 1 month | Stress (PHS)  Depression (EPDS) | 1 month PP | CS | N |
| **Bienfait**  **2011**^6^ | France (HIC) | Parents of infants in neonatal care | Median = 30 | Mixed | 78 | MIBS | >48 hours | Depression (EPDS) | >48 hours PP | CS | Y |
| **Bonacquisti**  **2020**^7^ | USA (HIC) | Parents of infants in neonatal care | 29.63 | Mixed | 127 | MAI^1^ | Range 1-52 weeks | Depression (EPDS)  Anxiety (DASS-42)  Stress (DASS-42) | 1-52 weeks PP | CS | Y |
| **Borji**  **2018**^8^ | Iran (UMIC) | Community | 27.04 | Mixed | 300 | PBQ | 8-10 weeks | Depression (EPDS)  Anxiety (STAI) | 8-10 weeks PP | CS | N |
| **Borschmann**  **2019**^9^ | Australia (HIC) | Community | Various | Mixed | 384 | PBQ | 2 months  12 months | Depression (EPDS)  Mixed Anxiety & Depression (CIS-R) | Trimester 3 PG  2 months PP  12 months PP  Preconception: maternal age 14-21 years | L | N |
| **Busonera**  **2017**^10^ | Italy (HIC) | Community | Range = 20-43 | NR | 123 | PBQ | 3 months | Depression (CES-D) | 3 months PP | CS | Y |
| **Carter**  **2015**^11^ | Data collected from 20 countries | Community | 27.2 | Mixed | 43 | MPAS | 0-12 months | Depression (EPDS) | 0-12 months PP | CS | Y |
| **Chenery**  **2013**^12^ | UK (HIC) | Community | 27.4 | Primipara | 64 | MPAS | 9.02 weeks | Depression (EPDS) | 9.02 weeks PP | CS | Y |
| **Choi**  **2017**^13^ | South Africa (UMIC) | Community | 25 | Mixed | 150 | PBQ | 12 months | Depression (EPDS)  Psychological distress (K10) | Range: Pregnancy - 6 months PP | L | N |
| **Chrzan-Dętkoś**  **2015**^14^ | Poland (HIC) | Community | 28.72 | Mixed | 64 | PBQ | 8 weeks | Depression (EPDS) | 8 weeks PP | CS | Y |
| **Condon**  **1998**^15^ | Australia (HIC) | Community | 27.4 | Mixed | 210 | MPAS | 4 months | Depression (ZSDS, HADS)  Anxiety (HADS) | 4 months PP | CS | Y |
| **Cuijlits**  **2016**^16^ | The Netherlands (HIC) | Community | 30.5 | Mixed | 1050 | PPBS | 8 months  12 months | Depression (EPDS) | 8 months PP  12 months PP | L | Y |
| **Cuijlits**  **2019**^17^ | The Netherlands (HIC) | Community | 30.56 | Mixed | 793 | PPBS | 8 months | Depression (EPDS) | 8 months | CS | N |
| **Daglar**  **2018**^18^ | Turkey (UMIC) | Community | 22.4 | Mixed | 227 | MIBS | 7-8 days | Depression (CES-D)  Anxiety (BAI) | 35 weeks PG  7-8 days | L/CS | Y |
| **Damato**  **2004**^19^ | USA (HIC) | Parents of twins | 32.1 | Mixed | 139 | MAI | 9.8 weeks | Depression (EPDS) | 9.8 weeks PP | CS | N |
| **Dubber**  **2015**^20^ | Germany (HIC) | Community | 32.8 | Mixed | 54 - 79 | PBQ-German | 12 weeks | Depression (EPDS)  Anxiety (STAI) | 32 weeks PG  12 weeks PP | L/CS | Y |
| **Edhborg**  **2005**^21^ | Sweden (HIC) | Community | 32.13 | Mixed | 106 | PBQ | 1 week  2 months | Depression (EPDS) | 1 week PP  2 months PP | L/CS | N |
| **Edhborg**  **2011**^22^ | Bangladesh (LMIC) | Community | 24.6 | Mixed | 671 | PBQ | 2-3 months | Depression (EPDS)  Anxiety (STAI) | 2-3 months PP | CS | Y |
| **Edhborg**  **2013**^23^ | Bangladesh (LMIC) | Clinical & community | 26.1 | Mixed | 45-55 | PBQ | 2-3 months | Depression (EPDS) | 2-3 months PP | CS | N |
| **Faisal-Cury**  **2020**^24^ | Brazil (UMIC) | Clinical | 26.5 | Mixed | 266-281 | PBQ | 6-8 months | Depression (PHQ9) | 6-8 months PP | CS | Y |
| **Fallon**  **2019**^25^ | UK (HIC) | Community | 31.25 | Mixed | 261 | PBQ | 16.1 weeks | Depression (BDI-II)  Anxiety (STAI) | 16.1 weeks PP | CS | Y |
| **Farré-Sender**  **2018**^26^ | Spain (HIC) | Clinical | 34.01 | Mixed | 249-251 | PBQ | 6-7 weeks | Depression (EPDS)  Anxiety (STAI) | Trimester 1-2 PG | L | Y |
| **Figueiredo**  **2009**^27^ | Portugal (HIC) | Community | 26.6 | Mixed | 315 | MIBS | 0-2 days | Depression (EPDS) | 0-2 days PP | CS | Y |
| **Figueiredo**  **2009a**^28^ | Portugal (HIC) | Community | 27.2 | Primipara | 91 | MIBS | 3 months | Depression (EPDS)  Anxiety (STAI) | NR | L | N |
| **Fijalkowska**  **2019**^29^ | Poland (HIC) | Community | 26.75 | Primipara | 35 | MPAS | 2-8 weeks | Depression (EPDS) | 2-8 weeks PP | CS | Y |
| **Fleming**  **1988**^30^ | Canada (HIC) | Community | 28 | Primipara | 56 | MAS^1^  (Feelings of Attachment subscale) | 1 month  3 months | Mixed Anxiety & Depression (MSS) | 9 months PG  3 days PP  1 month PP  3 months PP | L | N |
| **Fransson**  **2020**^31^ | Sweden (HIC) | Community | 31.9 | Mixed | 1090 | PBQ | 6 months | Depression (EPDS) | 17 weeks PG  32 weeks PG  6 weeks PP  6 months PP | L/CS | Y |
| **Friedman**  **2008**^32^ | USA (HIC) | Community | 32.38 | Mixed | 50 | PBQ | 14.59 weeks | Depression (EPDS)  Anxiety (SCL-90) | 14.59 weeks PP | CS | Y |
| **Garcia-Esteve**  **2016**^33^ | Spain (HIC) | Clinical & community | 34 | Mixed | 840 | PBQ | 4-6 weeks | Depression (EPDS) | 4-6 weeks PP | CS | Y |
| **Gashe**  **2011**^34^ | UK (HIC) | Community | 30.73 | Mixed | 190 | PBQ | 6.2months | Depression (EPDS) | 6.2 months PP | CS | Y |
| **Hairston**  **2011**^35^ | USA (HIC) | Clinical & community | 18-47 | Primipara | 184 | PBQ | 4 months | Depression (PDSS) | 4 months PP | CS | N |
| **Hairston**  **2016**^36^ | Israel (HIC) | Community | Range  = 21-45 | NR | 152 | PBQ | 5.86 months | Depression (EPDS) | 5.86 months PP | CS | Y |
| **Hairston**  **2018**^37^ | Israel (HIC) | Community | 31.73 | Mixed | 114 | PBQ | 7.6 weeks | Depression (EPDS) | 7.6 weeks PP | CS | Y |
| **Hairston**  **2019**^38^ | Israel (HIC) | Community | 31.9 | Mixed | 271 | PBQ | 4.3 months | Depression (EPDS) | 4.3 months PP | CS | N |
| **Handelzalts**  **2019**^39^ | Israel (HIC) | Community | 31 | Mixed | 504 | PBQ | 5.2 months | Depression (EPDS) | 5.2 months PP | CS | Y |
| **Herguner**  **2014**^40^ | Turkey (UMIC) | Community | 28.8 | NR | 80 | MAI | 23.97 weeks | Depression (EPDS) | 23.97 weeks PP | CS | Y |
| **Hiroko**  **2020**^41^ | Japan (HIC) | Community | NR | NR | 3370 | MIBS | 0-120 days | Depression (EPDS) | 0-120 days PP | CS | N |
| **Høivik**  **2013**^42^ | Norway (HIC) | Community | 29.4 | Mixed | 20-55 | MABISC  PBQ | 2 months  4 months | Depression (EPDS) | 2 months PP  4 months PP | L/CS | Y |
| **Hrishikesh**  **2019**^43^ | India (LMIC) | NR | NR | NR | 100 | PBQ | 6 weeks | Depression (EPDS) | 6 weeks PP | CS | Y |
| **Jones**  **2011**^44^ | UK (HIC) | Community | 30.9 | Mixed | 178 | M-PHI (Relationship with Baby subscale) | 0-12 months | Depression (EPDS) | NR | CS | Y |
| **Jordan**  **2014**^45^ | Australia (HIC) | Parents of infants in neonatal care | 32.9 | Mixed | 74 | MPAS | 102.2 days | Depression (EPDS) | 102.2 days PP | CS | Y |
| **Kaneko**  **2014**^46^ | Japan (HIC) | Community | 29.8 | Mixed | 1786 | PBQ | 123.6 days | Depression (EPDS) | 123.6 days PP | CS | Y |
| **Kasamatsu**  **2019**^47^ | Japan (HIC) | Community | 31.4 | Mixed | 83109 | MIBS-J | 12 months | Depression (EPDS) | 1 month PP  6 months PP | L | Y |
| **Kerstis**  **2016**^48^ | Sweden (HIC) | Community | 31 | Mixed | 727 | PBQ | 6 months | Depression (EPDS) | 6 weeks PP | L/CS | Y |
| **Kita**  **2016**^49^ | Japan (HIC) | Community | 32.2 | Mixed | 562 | MIBS-J | 1 month | Depression (HADS)  Anxiety (HADS) | PG Trimester 3  1 month PP | L/CS | N |
| **Kita**  **2020**^50^ | Japan (HIC) | Community | 32.2 | Mixed | 562 | MIBS-J | 1 month | Depression (HADS)  Anxiety (HADS) | PG Trimester 3  1 month PP | L/CS | Y |
| **Kleiber**  **2015**^51^ | USA (HIC) | Community | 18.83 | Mixed | 102 | PBQ | 5.65 months | Depression (EPDS)  Stress (PSS) | 5.65 months PP | CS | Y |
| **Kokubu**  **2012**^52^ | Japan (HIC) | Community | 29.9 | Mixed | 99 | MIBS-J | 5 days PP  1 month PP | Depression (HADS)  Anxiety (HADS)  Blues (MBQ) | 33-35 weeks PG  5 days PP  1 month PP | L/CS | Y |
| **Lara-Cinisomo**  **2018**^53^ | USA (HIC) | Community | 18-45 years | Mixed | 28 | PBQ | 8 weeks | Depression (EPDS) | Trimester 3 PG  4 weeks PP  8 weeks PP | L/CS | Y |
| **Le Bas**  **2020**^54^ | Australia (HIC) | Clinical & community | NR | Mixed | 1579 | MPAS | 8 weeks  12 months | Depression (EPDS)  Anxiety (DASS-21)  Stress (DASS-21) | Trimester 1 PG  Trimester 2 PG  Trimester 3 PG  8 weeks PP  12 months PP | L/CS | Y |
| **Leahy-Warren**  **2020**^55^ | Ireland (HIC) | Parents of infants in neonatal care | Range =  20-40+ | Mixed | 140 | MPAS | 7 months | Depression (EPDS) | 7 months PP | CS | Y |
| **Lehnig**  **2019**^56^ | Germany (HIC) | Community | 30.58 | Mixed | 725 | PBQ-German | 8.09 weeks | Depression (BDI-II)  Anxiety (SCL-90) | 8.09 weeks PP | CS | Y |
| **Leserman**  **2011**^57^ | USA (HIC) | NR | 27 | NR | 70 | PBQ | 6 weeks | Depression (MINI) | 6 weeks PP | CS | Y |
| **Loh**  **2004**^58^ | UK (HIC) | Clinical | 28.2 | Mixed | 41 | PBQ | 16.1 weeks | Depression (EPDS) | 16.1 weeks PP | CS | N |
| **Lutkiewicz**  **2020**^59^ | Poland (HIC) | Community | 30.71 | Mixed | 150 | PBQ | 1-3 days | Depression (EPDS/PDSS)  Anxiety (GAD-7) | 1-3 days PP | CS | Y |
| **Luz**  **2017**^60^ | France (HIC) | Community | 30.2 | Mixed | 40 | MPAS | 2 months | Anxiety (HADS)  Depression (HADS) | Trimester 3 PG | L | Y |
| **Macdonald**  **2020**^61^ | Australia (HIC) | Community | Prospective data collected | Mixed | 474 - 582 | MPAS | 12 months | Depression (SMFQ/DASS-21)  Anxiety (RBPC/RCMAS-SF/DASS-21) | Prospective, maternal age:  13 years  15 years  17 years  19 years  23 years  27 years  and 12 months PP | L/CS | Y |
| **Mackie**  **2019**^62^ | UK (HIC) | Parents of twins | 28.76 | Mixed | 5 | MPAS | 6-10 weeks | Depression (EPDS) | 6-10 weeks PP | CS | Y |
| **Martini**  **2020**^63^ | Germany (HIC) | Community | Group means range = 27.1-28.5 | Mixed | 306 | PBQ | 2 months | Mixed Anxiety & Depression (CIDI-V) | From trimester 1 PG to 16 months PP | L | Y |
| **Mason**  **2011**^64^ | USA (HIC) | Community | 23.5 | Primipara | 276 | MPAS | 2.7 months | Depression (EPDS) | 2.7 months PP | CS | Y |
| **Matthies**  **2020**^65^ | Germany (HIC) | Community | 32.8 | Mixed | 249 | PBQ-German | 1 week | Depression (EPDS)  Anxiety (STAI) | 1 week PP | CS | N |
| **McErlean**  **2012**^66^ | Australia (HIC) | Community | 33.8 | Mixed | 77 | PBQ | 17.75 weeks | Depression (EPDS) | 17.75 weeks PP | CS | Y |
| **Mercer**  **1990**^67^ | USA (HIC) | Community | 28.8 | Mixed | 121-182 | HIFMBN | 1 week  8 months | Depression (CES-D)  Anxiety (STAI) | 1 week PP  8 months PP | CS | N |
| **Mercer**  **1994**^68^ | USA (HIC) | Community | 28 - 29 | Multipara/Primipara | 109 - 138 | HIFMBN | 1 week  4 months | Depression (CES-D)  Anxiety (STAI) | 1 week PP  4 months PP | CS | N |
| **Milne**  **2007**^69^ | Australia (HIC) | Community | 29.86 | NR | 139 | MAS^2^ | 7.66 months | Depression (EPDS) | 7.66 months PP | CS | Y |
| **Minamida**  **2020**^70^ | Japan (HIC) | Community | Group means range =  28.0 - 29.2 | Primipara | 185 | PBQ | 1 day  3 months | Depression (EPDS) | 1 day PP  3 months PP | CS | Y |
| **Moehler**  **2006**^71^ | Germany (HIC) | Community | 33.3 | NR | 101 | PBQ | 2 weeks  6 weeks  4 months | Depression (EPDS) | 4 months PP | L/CS | Y |
| **Morrison**  **2016**^72^ | USA (HIC) | Community | 25.3 | Mixed | 70 | MPAS | 32 days | Depression (EPDS) | 32 weeks PG  32 days PP | L/CS | Y |
| **Moser**  **1989**^73^ | USA (HIC) | Community | 31.4 | NR | 37 | PPAM | 9 months | Depression (BDI-II)  Stress (various) | 4 months PP  9 months PP | L/CS | Y |
| **Müller**  **2013**^74^ | Germany (HIC) | Community | 27.15 | Mixed | 66 | PBQ-German | 5.4 weeks | Depression (BDI-II) | 36.09 weeks PG  5.4 weeks PP | L | N |
| **Muzik**  **2013**^75^ | USA (HIC) | Community | 29 | NR | 150 | PBQ | 4 months  6 months | Depression (PPDS) | Various | L | N |
| **Muzik**  **2017**^76^ | USA (HIC) | Clinical & community | 29.18 | NR | 122 | PBQ | 6 months | Depression (PPDS) | 6 months PP | CS | Y |
| **Myers**  **2017**^77^ | UK (HIC) | Community | 31.7 | Mixed | 48 | MIBS | 1 month | Depression (EPDS & BPDS) | 6 months PP | L | Y |
| **Nagata**  **2000**^78^ | Japan (HIC) | Community | 29.9 | Mixed | 417 | MAS^2^ | 5.2 days | Depression (ZSDS) | 5.2 days PP | CS | Y |
| **Nagata**  **2004**^79^ | Japan (HIC) | Parents of infants in neonatal care | 29.9 | Mixed | 153 | MAS^2^ | 6.1 days | Depression (ZSDS) | 6.1 days PP | CS | Y |
| **Nakano**  **2019**^80^ | Japan (HIC) | Community | 29.9 | Mixed | 1060 | PBQ | 1-3 months - not clearly reported | Depression (EPDS) | 1-3 months PP | CS | Y |
| **Nakash**  **2016**^81^ | Israel (HIC) | Asylum seekers | 26 | NR | 38 | MPAS | 0-6 months | Depression (EPDS) | 0-6 months PP | CS | N |
| **Nath**  **2019**^82^ | UK (HIC) | Community | 32.86 | Mixed | 380-404 | PBQ | 3 months | Depression (EPDS/SCID-I)  Anxiety (SCID-I) | 14 weeks PG  29 weeks PG | L | Y |
| **Ngoma**  **2012**^83^ | Japan (HIC) | Community | 29 | Primipara/Multipara | 118 | MIBS-J | 1 month | Depression (EPDS) | 1 month PP | CS | Y |
| **Nolvi**  **2016**^84^ | Finland (HIC) | Community | 29.9 | Mixed | 102 | PBQ | 6 months | Depression (EPDS)  Anxiety (STAI) | 3 months PP | L | Y |
| **Nonnenmacher**  **2016**^85^ | Germany (HIC) | Clinical & community | Group means range = 33.75-34.32 | Mixed | 93 | PBQ-German | 14.59 weeks | Depression (SCID-I) | 14.59 weeks PP | CS | Y |
| **Noyman-Veksler**  **2015**^86^ | Israel (HIC) | Community | 28.7 | Primipara | 92 - 96 | PBQ | 6 weeks  12 weeks | Depression (EPDS) | 6 weeks PP  12 weeks PP | L/CS | Y |
| **O'Higgins**  **2013**^87^ | UK (HIC) | Community | Group means range = 32.3-34.6 | Mixed | 79 | MIBS | 1-4 weeks  9 weeks  16 weeks  12 months | Depression (EPDS) | 4 weeks PP | L | Y |
| **Oddo-Sommerfeld  2016**^88^ | Germany (HIC) | Community | 32.35 | Mixed | 266 | PBQ | 12 weeks | Depression  (EPDS/BDI-V)  Anxiety (STADI) | Trimester 3 PG  12 weeks PP | L/CS | Y |
| **Ohara**  **2016**^89^ | Japan (HIC) | Community | 32.1 | NR | 751 | MIBQ | 5 days  1 month | Depression (EPDS) | 5 days PP  1 month PP | CS | Y |
| **Ohara**  **2017 (a)** ^90^ | Japan (HIC) | Community | 32.4 | Mixed | 494 | MIBQ | 1 month | Depression (EPDS)  Anxiety (EPDS)  Anhedonia (EPDS) | <25 weeks PG  1 month PP | L/CS | N |
| **Ohara**  **2017 (b)** ^91^ | Japan (HIC) | Community | 32.1 | NR | 751 | MIBQ | 5 days | Depression (EPDS)  Anxiety (EPDS)  Anhedonia (EPDS) | <25 weeks PG  36 weeks PG  5 days PP | L/CS | Y |
| **Ohara**  **2018**^92^ | Japan (HIC) | Community | 32.4 | Mixed | 855 | MIBQ | 1 month | Depression (EPDS)  Anxiety (EPDS)  Anhedonia (EPDS) | <25 weeks PG  5 days PP  1 month PP | L/CS | Y |
| **Ohashi**  **2016**^93^ | Japan (HIC) | Community | 30.3 | Mixed | 364 | PBQ | 1 month | Depression (EPDS) | 1 month PP | CS | Y |
| **Ohoka**  **2014**^94^ | Japan (HIC) | Community | 31.7 | Mixed | 388 | MIBS | 5 days  1 month | Depression (EPDS) | 5 days PP  1 month PP | CS | N |
| **Olsson**  **2020**^95^ | Australia (HIC) | Community | 29-35 years | Mixed | 469-560 | PBQ | 2 months  12 months | Mixed Anxiety & Depression (CIS-R/GHQ-12) | Prospective, at maternal age:  14-17 years  19 years  20 years  24 years  29 years | L | Y |
| **Örün**  **2013**^96^ | Turkey (UMIC) | Community | 25.1 | Mixed | 189 | PBQ  MIBS | 1.5-2 months | Depression (EPDS/BSI)  Anxiety (BSI) | 1-3 days PP  1.5-2 months PP | L/CS | Y |
| **Oskovi-Kaplan**  **2020**^97^ | Turkey (UMIC) | Parents of infants in neonatal care | 26 | Mixed | 223 | MAI | 0-48 hours | Depression (EPDS) | 0-48 hours PP | CS | Y |
| **Parfitt**  **2014**^98^ | UK (HIC) | Community | 33.04 | Primipara | 48 | PBQ | 11.7 weeks | Mixed Anxiety & Depression (HADS) | >30 weeks PG  3 months PP | L | Y |
| **Pearson**  **2013**^99^ | UK (HIC) | Community | 30.1 | Mixed | 49 | PBQ | 18 weeks | Depression (EPDS) | NR | CS | Y |
| **Petri**  **2017**^100^ | Italy (HIC) | Community | 33.93 | Mixed | 106 | MPAS | 1 month | History of mood disorder (SCID-I) | 1 month PG | L | Y |
| **Rados**  **2020**^101^ | Croatia (HIC) | Community | 30.64 | Mixed | 603 | PBQ | 1-6 months  7-12 months | Depression (EPDS) | 1-6 months PP  7-12 months PP | CS | Y |
| **Reck**  **2006**^102^ | Germany (HIC) | Community | 33 | NR | 862 | PBQ-German | 2 weeks | Depression (SCID-I) | 2 weeks PP | CS | Y |
| **Reck**  **2015**^103^ | Germany (HIC) | Clinical & community | 32.3 | Mixed | 63 | PBQ-German | 3.9 months | Depression (SCID-I) | 3.9 months PP | CS | Y |
| **Riera-Martin**  **2018**^104^ | Spain (HIC) | Community | 34.13 | NR | 571 | MPAS | 6-11 months | Depression (EPDS) | 6-11 months PP | CS | Y |
| **Robakis**  **2015**^105^ | USA (HIC) | Community | 32.2 | Mixed | 90-118 | MIBS | 1 month  2 months  3 months | Depression (EPDS) | 32.8 weeks PG  1 month PP  2 months PP  3 months PP | L/CS | Y |
| **Rossen**  **2016**^106^ | Australia (HIC) | Clinical & community | 32.59 | Mixed | 368-370 | MPAS | 8 weeks | Depression (EPDS)  Anxiety (DASS-21)  Stress (DASS-21) | Trimester 1 PG  Trimester 2 PG  Trimester 3 PG  8 weeks PP | L/CS | N |
| **Rossen**  **2019**^107^ | Australia (HIC) | Clinical & community | 32.51 | Mixed | 308 | MPAS | 8 weeks  12 months | Depression (EPDS)  Anxiety (DASS-21)  Stress (DASS-21) | 8 weeks PP | L/CS | N |
| **Sawyer Cohen**  **2010**^108^ | USA (HIC) | Community | 30.32 | Primipara | 168-186 | MAI | 3-8 months | Depression (EPDS)  Anxiety (STAI) | Trimester 2-3 PG  3-8 months PP | L/CS | Y |
| **Schmidt**  **2017**^109^ | Germany (HIC) | Community | 29.86 | Mixed | 184 | PBQ | 15.53 weeks | Depression (EPDS) | 13.66 weeks PG | L | N |
| **Scopesi**  **2004**^110^ | Italy (HIC) | Community | 32.1 | Mixed | 208 | MPAS | 78.4 days | Depression (SRT)  Anxiety (SRT) | 78.4 days PP | CS | Y |
| **Seng**  **2013**^111^ | USA (HIC) | Community | 27 | Primipara | 566 | PBQ | 6 weeks | Depression (CIDI-D) | 6 weeks PP (retrospective report of history) | L | Y |
| **Sockol**  **2014**^112^ | USA (HIC) | Clinical | 29 | Mixed | 180 | PBQ | 9 weeks | Depression (EPDS) | 9 weeks PP | CS | Y |
| **Suetsugu**  **2015**^113^ | Japan (HIC) | Community | 30.67 | Mixed | 244 | PBQ | 4 weeks | Depression (EPDS) | 4 weeks PP | CS | Y |
| **Suetsugu  2020**^114^ | Japan (HIC) | Community | 31.1 | Mixed | 130 | PBQ | 1 month  4 months | Depression (EPDS) | 1 month PP  4 months PP | CS | N |
| **Sun-Hee**  **2019**^115^ | South Korea (HIC) | Community | 32.34 | Mixed | 217 | MAI | 1 month | Depression (Likert Question with 3 response options) | 1 month PP | CS | Y |
| **Talmon**  **2019**^116^ | Israel (HIC) | Community | 30.96 | NR | 394 | PBQ | 8.54 weeks | Depression (EPDS) | Pregnancy  8.54 weeks PP | L/CS | Y |
| **Talmon**  **2020**^117^ | Israel (HIC) | Community | 30.96 | Mixed | 393 | PBQ | 8.54 weeks | Depression (EPDS) | 8.54 weeks PP | CS | N |
| **Taylor  2005**^118^ | UK (HIC) | Community | 31.9 | Mixed | 144 | MIBS | 3 days  12 weeks | Depression (EPDS)  Blues (KBS) | 3 days PP  12 weeks PP | L/CS | Y |
| **Tester-Jones**  **2015**^119^ | UK (HIC) | Community | 31.45 | Mixed | 203 | PBQ | 11.28 months | Depression (EPDS) | 11.28 months PP | CS | Y |
| **Tietz**  **2014**^120^ | Germany (HIC) | Clinical & community | Group means range =  32.9 -33.73 | NR | 78 | PBQ-German | 4.1 months | Depression (EPDS)  Anxiety (SCID-I) | 4.1 months PP | CS | Y |
| **Tikotzky**  **2016**^121^ | Israel (HIC) | Community | 30.8 | Mixed | 80 | MPAS  PBQ | 8.8 months | Depression (EPDS) | 8.8 months PP | CS | Y |
| **Tolja**  **2020**^122^ | Croatia (HIC) | Community | 29.48 | NR | 241 | PBQ | 6.34 months | Depression (EPDS/History)  Anxiety (DASS-21) | 6.34 months PP | CS | Y |
| **Tsuchida**  **2019**^123^ | Japan (HIC) | Community | 31.5 | Mixed | 76373 | MIBS-J | 1 month | Depression (EPDS) | 1 month PP | CS | N |
| **VanBussel**  **2010**^124^ | Belgium (HIC) | Community | 30.41 | NR | 202-263 | MIBS  MPAS  PBQ | 20-25 weeks  8-12 weeks | Depression (EPDS)  Anxiety (HADS) | 20-25 weeks PP  8-12 weeks PP | CS | Y |
| **Vengadavaradan**  **2019**^125^ | India (LMIC) | Community | NR | NR | 250 | PBQ | 4 wks - 6 months | Depression (EPDS) | 4 weeks - 6 months PP | CS | Y |
| **Vreeswijk**  **2011**^126^ | The Netherlands (HIC) | NR | NR | NR | 385 | MPAS | 6 months | Depression (EPDS)  Anxiety (STAI) | 15 weeks PG | L | N |
| **Wikman**  **2020**^127^ | Sweden (HIC) | Community | NR | Mixed | 209-361 | PBQ | 6 weeks | Depression (EPDS) | 17 or 32 weeks PG  6 weeks PP  6 months PP | L/CS | Y |
| **Williams**  **2016**^128^ | UK (HIC) | Clinical & community | 31.46 | Mixed | 502 | MPAS | 26.7 weeks | Depression (EPDS) | 26.7 weeks PP | CS | Y |
| **Wittkowski**  **2007**^129^ | UK (HIC) | Community | 28.79 | Primipara | 96 | MIBS  PBQ | 2-4 days | Blues (KBS) | 2-4 days PP | L/CS | Y |
| **Wittkowski**  **2010**^130^ | UK (HIC) | Clinical | 29.17 | Mixed | 132 | PBQ | 3.07 months | Depression (BDI-II) | 3.07 months PP | CS | Y |
| **Yoshida**  **2012**^131^ | Japan (HIC) | Community | 29.6 | Mixed | 554 | MIBS-J | 5 days | Depression (EPDS) | 5 days PP  1 month PP  4 months PP | CS | Y |
| **Zeitlin**  **1999**^132^ | UK (HIC) | Mixed community & domestic abuse survivors | 25.3 - 27 | NR | 38 | BPNB | 0-12 months | Depression (EPDS) | 0-12 months PP | CS | N |
| **Zhang**  **2017**^133^ | China (UMIC) | Community | 29.78 | Primipara | 255 | MAI | 0-2 weeks | Depression (HAMD) | 37-42 weeks PG  0-2 weeks PP  2-6 weeks PP  4-8 weeks PP | L | Y |

**Abbreviations used in eTable 2:** Country income classification: HIC = high-income country, UMIC = upper-middle-income country, LMIC = low-middle-income country; Bonding Measures: BPNB = Birmingham Postnatal Bonding Questionnaire, HIFMBN = How I Feel About My Baby Now Scale, MABISC = Mother and Baby Interaction Scale, MAI = Maternal Attachment Inventory, MAS^1^ = Maternal Attitudes Scale (Fleming, 1988), MAS^2^ – Maternal Attachment Scale (Nagata, 2000), MIBS = Mother infant bonding scale, MIBS-J = Mother Infant Bonding Scale – Japanese, MPAS = Maternal Postnatal Attachment Scale, M-PHI = Mother’s Postnatal Health Instrument, PBQ = Postpartum Bonding Questionnaire, PBQ-German = PBQ 16-item German version, PPAM = Postpartum Attachment Questionnaire, PPBS = The Pre- and Postnatal Bonding Scale; Psychological distress measures: BAI = Beck Anxiety Inventory, BDI-II = Beck Depression Inventory – revised, BDI-V = Simplified Beck Depression Inventory, BSI = Brief Symptom Inventory, CES-D = Centre for Epidemiologic Studies Depression Scale, CIDI-D = Composite International Diagnostic Interview – Depression Module, CIDI-V = Composite International Diagnostic Interview for Women, CIS-R = Clinical Interview Schedule revised, DASS-42 = Depression, Anxiety, and Stress Scale, DASS-21 = Depression, Anxiety, and Stress Scale – short form, EPDS = Edinburgh Postnatal Depression Scale, GAD-7 = Generalized Anxiety Disorder Assessment, GHQ12 = General Health Questionnaire – 12-item version, HADS = Hospital Anxiety & Depression Scale, HAMD = Hamilton Rating Scale for Depression, K10 = Kessler Psychological Distress Scale, KBS = Kennerly Blues Scale, MBQ = Maternity Blues Questionnaire, MINI = Mini International Neuropsychiatric Interview, MSS = Mood State Scale, PDSS = Postpartum Depression Screening Scale, PHQ9 = Patient Health Questionnaire–9, PHS = Psychosocial Hassles Scale, PPDS = Postpartum Depression Screening Scale, PSS = Perceived Stress Scale, RBPC = Revised Behavior Problem Checklist, CMAS-SF = Revised Children’s Manifest Anxiety Scale, SCL-90 = Symptom Checklist-90-Revised, SCID-I = Structured Clinical Interview for DSM-IV, SLES = Stressful Life Events Scale, SMFQ = Short Mood and Feelings Questionnaire, SRT = Symptom Rating Test, STADI = State-Trait Anxiety Depression Inventory, STAI = Spielberger State Trait Anxiety Inventory, ZSDS = Zung Self-rating Depression Scale; Time of psychological distress assessment: PG = pregnancy, PP = postpartum; Analysis Design: CS = cross-sectional, L = longitudinal.

eTable 3. Articles excluded at full text.

|  | Article | Reason for exlusion |
| --- | --- | --- |
| 1. | Affonso DD. Assessment of women's postpartal adaptation as indicator of vulnerability to depression. US: ProQuest Information & Learning; 1982. | Did not examine association between bonding and psychological distress |
| 2. | Afolabi O, Bunce L, Lusher J, Banbury S. Postnatal depression, maternal–infant bonding and social support: a cross-cultural comparison of Nigerian and British mothers. *Journal of Mental Health* 2017: 1-7. | Bonding measured outside postnatal period |
| 3. | Afolabi O, Bunce L, Lusher J, Banbury S. Postnatal depression, maternal–infant bonding and social support: a cross-cultural comparison of Nigerian and British mothers. *Journal of Mental Health* 2020; **29**(4): 424-30. | Duplicate |
| 4. | Agostini F, Monti F, Dellabartola S, Neri E. Maternal prenatal anxiety and its relationship with early neonatal temperament, quality of caregiving and mother-infant interactions. *Archives of Women's Mental Health* 2013; **16**: S105. | No eligible bonding measure |
| 5. | Agterberg G, Hunfeld JAM, Wladimiroff JW, Passchier J. Mothers' trait anxiety and adaptation to an infant born subsequent to the loss of a late pregnancy. *Psychological Reports* 1997; **80**(1): 216-8. | No eligible bonding measure |
| 6. | Ahn HY, Lee J, Shin H-J. Kangaroo care on premature infant growth and maternal attachment and post-partum depression in South Korea. *Journal Of Tropical Pediatrics* 2010; **56**(5): 342-4. | Did not examine association between bonding and psychological distress |
| 7. | Ahn Y-M, Kim M-R. The effects of a home-visiting discharge education on maternal self-esteem, maternal attachment, postpartum depression and family function in the mothers of NICU infants. *Taehan Kanho Hakhoe Chi* 2004; **34**(8): 1468-76. | Did not examine association between bonding and psychological distress |
| 8. | Ahn Y-M, Kim M-R. The relationship between early neo-maternal exposure, and maternal attachment, maternal self-esteem and postpartum depression in the mothers of NICU infants. *Taehan Kanho Hakhoe Chi* 2005; **35**(5): 798-809. | Article not in English^a^ |
| 9. | Aiello R, Lancaster S. Influence of adolescent maternal characteristics on infant development. *Infant Mental Health Journal* 2007; **28**(5): 496-516. | No eligible psychological distress measure |
| 10. | Akbarzadeh M, Dokuhaki A, Joker A, Pishva N, Zare N. Teaching attachment behaviors to pregnant women: A randomized controlled trial of effects on infant mental health from birth to the age of three months. *Annals of Saudi Medicine* 2016; **36**(3): 175-83. | No eligible bonding measure |
| 11. | Alici-Evcimen Y, Sudak DM. Postpartum depression. *Primary Care Update for Ob/Gyns* 2003; **10**(5): 210-6. | Did not present original results |
| 12. | Allen M. The Effects of Resilience and Optimism in Maternal Role Attainment after Six to Twelve Months Postpartum in Women Hospitalized for Pregnancy Complications. *Effects of Resilience & Optimism in Maternal Role Attainment after Six to Twelve Months Postpartum in Women Hospitalized for Pregnancy Complications* 2017: 1. | No eligible bonding measure |
| 13. | Alway Y, Spry E, Romaniuk H, Olsson C, Patton G. Preconception common mental disorder and maternal-infant bonding problems: A prospective cohort study from adolescence. *Archives of Women's Mental Health* 2020; **23**(2): 283. | Duplicate |
| 14. | Ammerman SB. The impact of hearing loss on mother-infant bonding. US: ProQuest Information & Learning; 2010. | No eligible psychological distress measure |
| 15. | Antonio RD. A group intervention model for women diagnosed with cancer during the prenatal or postpartum period. US: ProQuest Information & Learning; 2010. | No eligible bonding measure |
| 16. | Apter G. Maternal borderline personality disorder and the peripartum: Challenges for mother and infant. *European Psychiatry* 2013; **28**. | No eligible bonding measure |
| 17. | Armstrong KL, Fraser JA, Dadds MR, Morris J. A randomized, controlled trial of nurse home visiting to vulnerable families with newborns. *Journal of Paediatrics and Child Health* 1999; **35**(3): 237-44. | No eligible bonding measure |
| 18. | Armstrong KL, Fraser JA, Dadds MR, Morris J. Promoting secure attachment, maternal mood and child health in a vulnerable population: A randomized controlled trial. *Journal of Paediatrics and Child Health* 2000; **36**(6): 555-62. | No eligible bonding measure |
| 19. | Armstrong VG, Howatson R. Parent–infant art psychotherapy: A creative dyadic approach to early intervention. *Infant Mental Health Journal* 2015; **36**(2): 213-22. | No eligible bonding measure |
| 20. | Arnold A, Lewis J, Maximovich A, Ickovics J, Kershaw T. Antecedents and Consequences of Caregiving Structure on Young Mothers and Their Infants. *Maternal & Child Health Journal* 2011; **15**(7): 1037-45. | No eligible bonding measure |
| 21. | Ashvini V, Bharadwaj B, Gopinath S, Jayalakshmi D. Can mother-infant bonding disorder be recognized by the members of the family? *Indian Journal of Psychiatry* 2018; **60**(5): S129. | Did not examine association between bonding and psychological distress |
| 22. | Athan A, Chung S, Sawyer Cohen J. Spiritual beliefs of mothers with potentially distressing pregnancies. *Spirituality in Clinical Practice* 2015; **2**(3): 216-32. | Did not examine association between bonding and psychological distress |
| 23. | Austin MP, Christl B, Reilly N, Yin C. Characteristics and outcomes of women admitted to a private psychiatric mother-baby unit. *Archives of Women's Mental Health* 2015; **18**(2): 377-8. | Did not examine association between bonding and psychological distress |
| 24. | Baines T, Wittkowski A, Wieck A. IPQ-R assessed illness perceptions in mothers with depression after childbirth. *Journal of Reproductive and Infant Psychology* 2011; **29**(3): e18-e9. | Duplicate |
| 25. | Baines T, Wittkowski A, Wieck A. Illness perceptions in mothers with postpartum depression. *Midwifery* 2013; **29**(7): 779-86. | Did not examine association between bonding and psychological distress |
| 26. | Ballen N, Gandhi J, Norris S, et al. Program evaluation of the Ottawa regional perinatal mental health program: Identifying barriers to care within the traditional academic hospital setting. *Archives of Women's Mental Health* 2013; **16**: S54. | Did not examine association between bonding and psychological distress |
| 27. | Banerjee I, Powis S, Shevlin M, Barnes J, Soo A, Sutcliffe AG. Health outcomes of children born to mothers with chronic kidney disease: A pilot study. *Pediatric Reports* 2010; **2**(1): 22-5. | No eligible bonding measure |
| 28. | Banti S, Borri C, Ramacciotti D, et al. Mother-baby attachment and perinatal depression. *Archives of Women's Mental Health* 2013; **16**: S85. | Did not examine association between bonding and psychological distress |
| 29. | Bayri Bingol F, Bal MD, Aydin Ozkan S, Zengin O, Civ B. The adaptation of the Postpartum-Specific Anxiety Scale into the Turkish language. *Journal of Reproductive and Infant Psychology* 2019. | No eligible bonding measure |
| 30. | Berman Z, Thiel F, Dishy GA, Chan SJ, Dekel S. Maternal psychological growth following childbirth. *Archives of Women's Mental Health* 2020. | Did not examine association between bonding and psychological distress |
| 31. | Bicking Kinsey C. Effect of prior perinatal loss on maternal depressive symptoms and maternal-infant bonding. US: ProQuest Information & Learning; 2015. | Duplicate |
| 32. | Bickle Graz M, Favrod C, Gilbert L, Morisod Harari M, Schneider J, Horsch A. Mental Health Of Parents After Perinatal Asphyxia. *European Journal of Pediatrics* 2016; **175**(11): 1809. | Did not examine association between bonding and psychological distress |
| 33. | Bittner A, Junge-Hoffmeister J, Treichel T, Coenen A, Weidner K. Maladaptive personality styles in a clinical sample of women with postpartum depression. *Journal of Affective Disorders* 2020; **263**: 318-25. | Did not examine association between bonding and psychological distress |
| 34. | Bonacquisti A. Psychological responses and treatment among mothers in the neonatal intensive care unit: the impact of attachment, attitudes, social support, and health behaviors. US: ProQuest Information & Learning; 2017. | Duplicate |
| 35. | Brassel A, Townsend ML, Pickard JA, Grenyer BFS. Maternal perinatal mental health: Associations with bonding, mindfulness, and self-criticism at 18 months’ postpartum. *Infant Mental Health Journal* 2020; **41**(1): 69-81. | Bonding measured outside postnatal period |
| 36. | Brents L, Young J, Knight B, et al. Maternal delay and social discounting behavior predicts impaired mother-infant bonding, maternal stress, and paternal involvement. *FASEB Journal* 2015; **29**(1). | Did not examine association between bonding and psychological distress |
| 37. | Brents LK, Young J, Knight BT, et al. Perceived social support and socioeconomic status predict maternal delay discounting behavior and neural function in healthy postpartum women. *FASEB Journal* 2016; **30**. | Did not examine association between bonding and psychological distress |
| 38. | Brockington IF, Fraser C, Wilson D. The Postpartum Bonding Questionnaire: A validation. *Archives of Women's Mental Health* 2006; **9**(5): 233-42. | Did not examine association between bonding and psychological distress |
| 39. | Brockington IF, Oates J, George S, et al. A screening questionnaire for mother-infant bonding disorders. *Archives of Women's Mental Health* 2001; **3**(4): 133-40. | Did not examine association between bonding and psychological distress |
| 40. | Brulja M, Clare CA, Inchiosa MA, Kubal KP. Patient evaluation for the development of postpartum depression. *Archives of Women's Mental Health* 2020; **23**(2): 289. | Bonding measured outside postnatal period |
| 41. | Buist A. Childhood abuse, parenting and postpartum depression. *Australian and New Zealand Journal of Psychiatry* 1998; **32**(4): 479-87. | No eligible bonding measure |
| 42. | Buultjens M, Murphy G, Milgrom J, Taket A, Poinen D. Supporting the transition to parenthood: Development of a group health-promoting programme. *British Journal of Midwifery* 2018; **26**(6): 387-97. | Did not examine association between bonding and psychological distress |
| 43. | Bytomski A, Ritschel G, Bierling A, Bendas J, Weidner K, Croy I. Maternal stroking is a fine-tuned mechanism relating to C-tactile afferent activation: An exploratory study. *Psychology and Neuroscience* 2020; **13**(2): 149-57. | Did not examine association between bonding and psychological distress |
| 44. | Camacho JM. The impact of mindfulness-based mom baby yoga on mindfulness, attachment, and stress during postpartum. 2016. | Intervention study did not report separately for control group |
| 45. | Capdevila E, Lasheras G, Porta R, Farré-Sender B, Farré JM. Postpartum depression, motherinfant bonding and postpartum coping strategies in mothers of newborns admitted to the neonatal unit. *Journal of Maternal-Fetal and Neonatal Medicine* 2016; **29**: 176-7. | Did not examine association between bonding and psychological distress |
| 46. | Capuzzi CO. Effects of stress and social support on maternal attachment with a handicapped infant. 1986; **8706827**: 476. | No eligible bonding measure |
| 47. | Castro RA. Maternal and paternal postpartum mood: Associations with early interactions with the baby. *Journal of Psychosomatic Research* 2019; **121**: 105. | No eligible bonding measure |
| 48. | Castro RA, Glover V, Ehlert U, Kammerer M. Maternal mental health in pregnancy: Association with breastfeeding, bonding and infant temperament at six months. *Archives of Women's Mental Health* 2015; **18**(2): 319-20. | Did not examine association between bonding and psychological distress |
| 49. | Chen S-L, Chen C-H. Effects of Lavender Tea on Fatigue, Depression, and Maternal-Infant Attachment in Sleep-Disturbed Postnatal Women. *Worldviews On Evidence-Based Nursing* 2015; **12**(6): 370-9. | Did not examine association between bonding and psychological distress |
| 50. | Cheng S, Kondo N, Aoki Y, Kitamura Y, Takeda Y, Yamagata Z. The effectiveness of early intervention and the factors related to child behavioural problems at age 2: a randomized controlled trial. *Early Human Development* 2007; **83**(10): 683-91. | No eligible bonding measure |
| 51. | Cheung SLF. Impact of maternal childbirth trauma on mother-child bonding, PTSD and depression. US: ProQuest Information & Learning; 2018. | Bonding measured outside postnatal period |
| 52. | Chiorino V, Cattaneo MC, Macchi EA, et al. The EMDR Recent Birth Trauma Protocol: a pilot randomised clinical trial after traumatic childbirth. *Psychology & Health* 2020; **35**(7): 795-810. | Did not examine association between bonding and psychological distress |
| 53. | Chittleborough C, Lawlor D, Lynch J. Prenatal Prediction of Poor Maternal and Offspring Outcomes: Implications for Selection into Intensive Parent Support Programs. *Maternal & Child Health Journal* 2012; **16**(4): 909-20. | No eligible bonding measure |
| 54. | Christl B, Reilly N, Yin C, Austin M-P. Clinical profile and outcomes of women admitted to a psychiatric mother-baby unit. *Archives of Women's Mental Health* 2015; **18**(6): 805-16. | Did not examine association between bonding and psychological distress |
| 55. | Cooijmans KHM, Beijers R, Rovers AC, de Weerth C. Effectiveness of skin-to-skin contact versus care-as-usual in mothers and their full-term infants: Study protocol for a parallel-group randomized controlled trial. *BMC Pediatrics* 2017; **17**(1). | Did not examine association between bonding and psychological distress |
| 56. | Cooklin AR, Rowe HJ, Fisher JRW. Paid parental leave supports breastfeeding and mother-infant relationship: a prospective investigation of maternal postpartum employment. *Australian & New Zealand Journal of Public Health* 2012; **36**(3): 249-56. | No eligible psychological distress measure |
| 57. | Cooper P, Murray L. Prediction, detection, and treatment of postnatal depression. *Archives Of Disease In Childhood* 1997; **77**(2): 97-9. | No eligible bonding measure |
| 58. | Cooper PJ, Landman M, Tomlinson M, Molteno C, Swartz L, Murray L. Impact of a mother-infant intervention in an indigent peri-urban South African context. Pilot study. *British Journal of Psychiatry* 2002; **180**(JAN.): 76-81. | No eligible bonding measure |
| 59. | Cooper PJ, Tomlinson M, Swartz L, Woolgar M, Murray L, Molteno C. Post-partum depression and the mother-infant relationship in a South African peri-urban settlement. *British Journal of Psychiatry* 1999; **175**(DEC.): 554-8. | No eligible bonding measure |
| 60. | Corfield FA. Attachment, affect and social processing in eating disorders. 2014; **10092871**. | Did not examine association between bonding and psychological distress |
| 61. | Cornish AM, McMahon C, Ungerer JA, Barnett B, Kowalenko N, Tennant C. Maternal depression and the experience of parenting in the second postnatal year. *Journal of Reproductive and Infant Psychology* 2006; **24**(2): 121-32. | Bonding measured outside postnatal period |
| 62. | Davies J, Slade P, Wright I, Stewart P. Posttraumatic stress symptoms following childbirth and mothers' perceptions of their infants. *Infant Mental Health Journal* 2008; **29**(6): 537-54. | Did not examine association between bonding and psychological distress |
| 63. | Davis GK, Roberts L, Henry A, et al. Postpartum physiology, psychology and paediatric study-P4 study. *Pregnancy Hypertension* 2016; **6**(3): 216-7. | Did not examine association between bonding and psychological distress |
| 64. | Dau ALBT, Callinan LS, Smith MV. An examination of the impact of maternal fetal attachment, postpartum depressive symptoms and parenting stress on maternal sensitivity. *Infant Behavior & Development* 2019; **54**: 99-107. | No eligible bonding measure |
| 65. | de Cock ESA, Henrichs J, Vreeswijk CMJM, Maas AJBM, Rijk CHAM, van Bakel HJA. Continuous feelings of love? The parental bond from pregnancy to toddlerhood. *Journal of Family Psychology* 2016; **30**(1): 125-34. | Bonding measured outside postnatal period |
| 66. | De Kruijff I, Choenni V, Groeneweg JT, et al. Gastrointestinal Symptoms in Infants of Mothers with a Psychiatric History and the Role of Depression and Bonding. *Journal of Pediatric Gastroenterology and Nutrition* 2019; **69**(6): 662-7. | Did not examine association between bonding and psychological distress |
| 67. | Dekel S, Thiel F, Dishy G, Ashenfarb AL. Is childbirth-induced PTSD associated with low maternal attachment? *Archives of Women's Mental Health* 2018: 1-4. | No eligible psychological distress measure |
| 68. | Dekel S, Thiel F, Dishy G, Ashenfarb AL. Is childbirth-induced PTSD associated with low maternal attachment? *Archives of Women's Mental Health* 2019; **22**(1): 119-22. | No eligible psychological distress measure |
| 69. | Diniz E, Koller SH, Volling BL. Social support and maternal depression from pregnancy to postpartum: The association with positive maternal behaviours among Brazilian adolescent mothers. *Early Child Development and Care* 2015; **185**(7): 1053-66. | No eligible bonding measure |
| 70. | Dolan R, Shaw J, Hann M. Pregnancy in prison, Mother and Baby Unit admission and impacts on perinatal depression and ‘quality of life’. *Journal of Forensic Psychiatry and Psychology* 2019; **30**(4): 551-69. | Did not examine association between bonding and psychological distress |
| 71. | Doster A, Wallwiener S, Müller M, et al. Reliability and validity of the German version of the Maternal–Fetal Attachment Scale. *Archives of Gynecology and Obstetrics* 2018; **297**(5): 1157-67. | Did not examine association between bonding and psychological distress |
| 72. | Eastwood J, Jalaludin B, Kemp L, Phung H, Barnett B, Tobin J. Social exclusion, infant behavior, social isolation, and maternal expectations independently predict maternal depressive symptoms. *Brain and Behavior* 2013; **3**(1): 14-23. | No eligible bonding measure |
| 73. | Eigner B. The role of playful interactions in the development of the early mother-child relationship--factors of risk and protection. *Psychiatria Hungarica: A Magyar Pszichiatriai Tarsasag Tudomanyos Folyoirata* 2015; **30**(1): 35-49. | Article not in English^a^ |
| 74. | Ekström A, Nissen E. A mother's feelings for her infant are strengthened by excellent breastfeeding counseling and continuity of care. *Pediatrics* 2006; **118**(2): e309-14. | No eligible psychological distress measure |
| 75. | Evans T, Whittingham K, Boyd R. What helps the mother of a preterm infant become securely attached, responsive and well-adjusted? *Infant Behavior and Development* 2012; **35**(1): 1-11. | Did not examine association between bonding and psychological distress |
| 76. | Fancourt D, Perkins R. Associations between singing to babies and symptoms of postnatal depression, wellbeing, self-esteem and mother-infant bond. *Public Health* 2017; **145**: 149-52. | No eligible bonding measure |
| 77. | Feldman R. What is resilience: an affiliative neuroscience approach. *World Psychiatry* 2020; **19**(2): 132-50. | Did not present original results |
| 78. | Feldman R, Weller A, Leckman JF, Kuint J, Eidelman AI. The nature of the mother's tie to her infant: Maternal bonding under conditions of proximity, separation, and potential loss. *Journal of Child Psychology and Psychiatry and Allied Disciplines* 1999; **40**(6): 929-39. | No eligible bonding measure |
| 79. | Fischer C. Effects of maternal postpartum depression on early childhood language development. US: ProQuest Information & Learning; 2016. | No eligible bonding measure |
| 80. | Fonseca VRJRM, Silva GAd, Otta E. The relationship between postpartum depression and maternal emotional availability. *Cadernos De Saude Publica* 2010; **26**(4): 738-46. | Article not in English^a^ |
| 81. | Foreman DM, Henshaw C. Objectivity and subjectivity in postnatally depressed mothers' perceptions of their infants. *Child Psychiatry and Human Development* 2002; **32**(4): 263-75. | No eligible bonding measure |
| 82. | Gabrieli C, Ciullo R, Sansone L, et al. Late-preterm delivery: Psychological distress and lactogenesis. *Archives of Disease in Childhood* 2012; **97**: A475. | Did not examine association between bonding and psychological distress |
| 83. | Galbally M, Watson SJ, Boyce P, Nguyen T, Lewis AJ. The mother, the infant and the mother-infant relationship: What is the impact of antidepressant medication in pregnancy. *Journal of Affective Disorders* 2020; **272**: 363-70. | No eligible bonding measure |
| 84. | Ganjekar S, Prakash A, Thippeswamy H, Desai G, Chandra PS. The NIMHANS (National Institute of Mental Health and Neuro Sciences) Maternal Behaviour Scale (NIMBUS): Development and validation of a scale for assessment of maternal behaviour among mothers with postpartum severe mental illness in low resource settings. *Asian Journal of Psychiatry* 2020; **47**. | Did not examine association between bonding and psychological distress |
| 85. | Garcia-Esteve L, Torres A, Farré-Sender B, et al. Mother's childhood abuse as risk factor for mother-infant bonding alteration. *Archives of Women's Mental Health* 2015; **18**(2): 327. | Duplicate |
| 86. | Gilden J, Molenaar NM, Smit AK, et al. Mother-to-infant bonding in women with postpartum psychosis and severe postpartum depression: A clinical cohort study. *Journal of Clinical Medicine* 2020; **9**(7): 1-10. | Did not examine association between bonding and psychological distress |
| 87. | Gordon H, Nath S, Trevillion K, et al. Self-harm, self-harm ideation, and mother-infant interactions: A prospective cohort study. *Journal of Clinical Psychiatry* 2019; **80**(5). | Did not examine association between bonding and psychological distress |
| 88. | Gray BA. Women's subjective appraisal of pregnancy risk and the effects of uncertainty, perceived control, coping and emotions on maternal attachment: Case Western Reserve University (Health Sciences); 2001. | Did not examine association between bonding and psychological distress |
| 89. | Hamm RF, Perelman S, Wang EY, Levine LD, Srinivas SK. Single-unit vs multiple-unit transfusion in hemodynamically stable postpartum anemia: a pragmatic randomized controlled trial. *American Journal of Obstetrics and Gynecology* 2020. | Did not examine association between bonding and psychological distress |
| 90. | Handelzalts JE, Krissi H, Levy S, et al. Personality, preterm labor contractions, and psychological consequences. *Archives of Gynecology and Obstetrics* 2016; **293**(3): 575-82. | Did not examine association between bonding and psychological distress |
| 91. | Hanko C, Bittner A, Junge-Hoffmeister J, Mogwitz S, Nitzsche K, Weidner K. Course of mental health and mother-infant bonding in hospitalized women with threatened preterm birth. *Archives of Gynecology and Obstetrics* 2020; **301**(1): 119-28. | Did not examine association between bonding and psychological distress |
| 92. | He Z, Lee SY. Stress to parenting competence among black mothers of preterm infants: The role of sleep. *Sleep* 2019; **42**: A272. | Did not examine association between bonding and psychological distress |
| 93. | Heberlein EC, Picklesimer AH, Billings DL, Covington-Kolb S, Farber N, Frongillo EA. The comparative effects of group prenatal care on psychosocial outcomes. *Archives of Women's Mental Health* 2016; **19**(2): 259-69. | Did not examine association between bonding and psychological distress |
| 94. | Henderson J, Alderdice F, Redshaw M. Factors associated with maternal postpartum fatigue: An observationalstudy. *BMJ Open* 2019; **9**(7). | No eligible bonding measure |
| 95. | Henderson J, Carson C, Redshaw M. Impact of preterm birth on maternal well-being and women's perceptions of their baby: a population-based survey. *BMJ Open* 2016; **6**(10): e012676-e. | No eligible bonding measure |
| 96. | Hergüner S, Annagür A, Çic¸ek E, Hergüner A, Örs R. Maternal attachment and postpartum depression in mothers of preterm infants and the role of attachment style. *Archives of Disease in Childhood* 2012; **97**: A368-A9. | Duplicate |
| 97. | Hergüner S, Çic¸ek E, Annagür A, Hergüner A, Örs R. Association of delivery type with postpartum depression and maternal attachment. *Archives of Disease in Childhood* 2012; **97**: A368. | Did not examine association between bonding and psychological distress |
| 98. | Hernández‐Martínez C, Voltas Moreso N, Arija Val V, Jardí Piñana C, Bedmar Carretero C, Canals J. The role of maternal emotional states during pregnancy and early infancy on infant cortisol levels: A prospective study. *Infant & Child Development* 2019; **28**(5): N.PAG-N.PAG. | No eligible bonding measure |
| 99. | Hirokawa K, Kimura T, Ikehara S, et al. Associations between broader autism phenotype (BAP) and maternal attachment are moderated by maternal postpartum depression when infants are one month old: A prospective study of the Japan environment & children's study. *Journal of Affective Disorders* 2019; **243**: 485-93. | Did not examine association between bonding and psychological distress |
| 100. | Høifødt RS, Nordahl D, Landsem IP, et al. Newborn Behavioral Observation, maternal stress, depressive symptoms and the mother-infant relationship: Results from the Northern Babies Longitudinal Study (NorBaby). *BMC Psychiatry* 2020; **20**(1). | Did not examine association between bonding and psychological distress |
| 101. | Honjo S, Murase S, Kaneko H, Arai S, Hashimoto O, Nomura K. Support for the family from infancy. *Seishin Shinkeigaku Zasshi = Psychiatria Et Neurologia Japonica* 2004; **106**(5): 602-7. | Article not in English^a^ |
| 102. | Hornstein C. Mother-child relationship quality improvement as effect of an interaction-focussed psychotherapy program for mothers with postpartum disorders. *Archives of Women's Mental Health* 2013; **16**: S81-S2. | Intervention study did not report separately for control group |
| 103. | Hornstein C, Trautmann-Villalba P, Hohm E, Rave E, Wortmann-Fleischer S, Schwarz M. Maternal bond and mother-child interaction in severe postpartum psychiatric disorders: Is there a link? *Archives of Women's Mental Health* 2006; **9**(5): 279-84. | Did not examine association between bonding and psychological distress |
| 104. | Horsch A, Jacobs I, Gilbert L, et al. Impact of perinatal asphyxia on parental mental health and bonding with the infant: a questionnaire survey of Swiss parents. *BMJ Paediatrics Open* 2017; **1**(1): e000059-e. | Bonding measured outside postnatal period |
| 105. | Howard M, Battle CL. Women & Infants Hospital MBU: USA model of care. *Archives of Women's Mental Health* 2020; **23**(2): 245-6. | Did not examine association between bonding and psychological distress |
| 106. | Howard M, Battle CL, Sockol LE, Deletto S. What about the baby? Postpartum depression and maternal bonding. *Archives of Women's Mental Health* 2015; **18**(2): 292-3. | Duplicate |
| 107. | Imura M. The psychological effects of aromatherapy-massage in healthy postpartum mothers. *Journal of Midwifery and Women's Health* 2006; **51**(2): e21-e7. | No eligible bonding measure |
| 108. | Ionio C, Colombo C, Brazzoduro V, et al. Mothers and Fathers in NICU: The Impact of Preterm Birth on Parental Distress. *Europe's Journal Of Psychology* 2016; **12**(4): 604-21. | Did not examine association between bonding and psychological distress |
| 109. | Irvine CD, Newton R. Maternal depression, maternal attachment and parental distress as predictors of child abuse. 2006; **1434529**: 54. | No eligible bonding measure |
| 110. | Jafarzadeh ZA, Maghsoudi J, Barekatain B, Marofi M. Effect of telenursing on attachment and stress in mothers of preterm infants. *Iranian Journal of Neonatology* 2019; **10**(1): 65-71. | Did not examine association between bonding and psychological distress |
| 111. | Jarvis S. An exploration of the contributions of posttraumatic growth following postnatal depression, perceived social support and current depression to the strength of the maternal bond. 2018; (13912179). | No eligible bonding measure |
| 112. | Jones R, Slade P, Pascalis O, Herbert JS. Infant interest in their mother's face is associated with maternal psychological health. *Infant Behavior and Development* 2013; **36**(4): 686-93. | No eligible bonding measure |
| 113. | Jordan B, Franich-Ray C, Albert N, et al. Characteristics of mother-infant relationships following neonatal cardiac surgery. *Journal of Paediatrics and Child Health* 2012; **48**: 36. | Duplicate |
| 114. | Junge-Hoffmeister J, Bittner A, Richter J, Schultz U, Joraschky P, Weidner K. Preventing peripartal psychosomatic symptoms in pregnant women - Is there an impact on pregnancy and birth complications or neonatal outcomes. *Archives of Women's Mental Health* 2011; **14**: S32. | Did not examine association between bonding and psychological distress |
| 115. | Kacar AS, Yilmaz O, Gogebakan E, et al. Negative psychosocial effect of dietary elimination on breastfeeding mothers having infants with and without food allergy. *Allergy: European Journal of Allergy and Clinical Immunology* 2019; **74**: 302-3. | Did not examine association between bonding and psychological distress |
| 116. | Kafumi S, Kiyoko K, Hiroya M. The Inter Relationship of Mental State between Antepartum and Postpartum Assessed by Depression and Bonding Scales in Mothers. *Health (1949-4998)* 2016; **8**(12): 1234-43. | Did not examine association between bonding and psychological distress |
| 117. | Karam F, Berard A, Sheehy O, et al. Impact of maternal attachment on infant development at one year of age: Results from the otis antidepressants in pregnancy study. *Birth Defects Research Part A - Clinical and Molecular Teratology* 2012; **94**(5): 347. | Duplicate |
| 118. | Karam F, Berard A, Sheehy O, et al. Impact of maternal attachment on infant development at 1-year of age: Results from the Otis antidepressants in pregnancy study. *Pharmacoepidemiology and Drug Safety* 2012; **21**: 151-2. | Did not examine association between bonding and psychological distress |
| 119. | Kenyon S, Jolly K, Hemming K, et al. Lay support for pregnant women with social risk: a randomised controlled trial. *BMJ Open* 2016; **6**(3): e009203-e. | Duplicate |
| 120. | Kenyon S, Jolly K, Hemming K, et al. Effects of additional lay support for pregnant women with social risk factors on antenatal attendance and maternal psychological health: A randomised controlled trial (ELSIPS). *Archives of Disease in Childhood: Fetal and Neonatal Edition* 2014; **99**: A18. | Did not examine association between bonding and psychological distress |
| 121. | Khazan IZ. Expectations-based intervention as a tool to reduce severity of postpartum depression and improve coparenting. US: ProQuest Information & Learning; 2006. | No eligible bonding measure |
| 122. | Kim P, Strathearn L, Swain JE. The maternal brain and its plasticity in humans. *Hormones and Behavior* 2016; **77**: 113-23. | Review or meta-analysis |
| 123. | Kita S, Hayashi M, Umeshita K, et al. Intimate partner violence and maternal child abuse: The mediating effects of mothers’ postnatal depression, mother-to-infant bonding failure, and hostile attributions to children’s behaviors. *Psychology of Violence* 2020; **10**(3): 279-89. | Did not examine association between bonding and psychological distress |
| 124. | Kraft A, Knappe S, Petrowski K, Petzoldt J, Martini J. Maternal bonding and infant attachment in women with and without social phobia. *Zeitschrift Fur Kinder- Und Jugendpsychiatrie Und Psychotherapie* 2017; **45**(1): 49-57. | Article not in English^a^ |
| 125. | Krieg DLB. The expanding family system: Accommodating the first or second child. US: ProQuest Information & Learning; 2001. | No eligible psychological distress measure |
| 126. | Kubota C, Okada T, Morikawa M, et al. Postpartum depression among women in Nagoya indirectly exposed to the Great East Japan Earthquake. *Scientific Reports* 2018; **8**(1): 11624-. | Did not examine association between bonding and psychological distress |
| 127. | Kumar RC. 'Anybody's child': Severe disorders of mother-to-infant bonding. *The British Journal of Psychiatry* 1997; **171**: 175-81. | Did not examine association between bonding and psychological distress |
| 128. | Lagerberg D, Magnusson M, Sundelin C. Drawing the line in the Edinburgh Postnatal Depression Scale (EPDS): A vital decision. *International Journal of Adolescent Medicine and Health* 2011; **23**(1): 27-32. | No eligible bonding measure |
| 129. | Lai BPY, Tang CSK, Tse WKL. A longitudinal study investigating disordered eating during the transition to motherhood among chinese women in Hong Kong. *International Journal of Eating Disorders* 2006; **39**(4): 303-11. | No eligible bonding measure |
| 130. | Lai M, D'Acunto G, Guzzetta A, et al. Premm: Preterm early massage by the mother-the effects of massage in very preterm infants. *Journal of Paediatrics and Child Health* 2016; **52**: 5. | Did not examine association between bonding and psychological distress |
| 131. | Lara MA, Navarrete L, Gómez MAE. Sexual abuse before age 17: A risk factor for postnatal depression, anxiety, and low self-esteem, maternal attachment, and self-efficacy: Implications for prevention. *Archives of Women's Mental Health* 2013; **16**: S49. | Did not examine association between bonding and psychological distress |
| 132. | Lasek DG. Mother-daughter attachment, social support, relationship with husband, and socioeconomic status as predictors of postpartum depression. US: ProQuest Information & Learning; 2000. | No eligible bonding measure |
| 133. | Lasheras G, Farré Sender B, Serra B, Serrano A, Caballero M. Two types of screening for postpartum depression (PD) and mother-infant bonding (MIB). *Archives of Women's Mental Health* 2015; **18**(2): 380-1. | Duplicate |
| 134. | Lasheras G, Farré-Sender B, Porta R, Mestre-Bach G. Risk factors for postpartum depression in mothers of newborns admitted to neonatal intensive care unit. *Journal of Reproductive and Infant Psychology* 2020. | Did not examine association between bonding and psychological distress |
| 135. | Lawless CM. Explorative study into psychological distress in parents of premature infants. 2007; **U239396**: 1. | Did not examine association between bonding and psychological distress |
| 136. | Lee SM. The effects of music therapy on postpartum blues and maternal attachment of puerperal women. *Journal Of Korean Academy Of Nursing* 2010; **40**(1): 60-8. | Article not in English^a^ |
| 137. | Lefkovics E, Rigó J, Kovács I, et al. Effect of maternal depression and anxiety on mother’s perception of child and the protective role of social support. *Journal of Reproductive and Infant Psychology* 2018: 1-15. | No eligible bonding measure |
| 138. | Letourneau NL, Dennis C-L, Benzies K, et al. Postpartum depression is a family affair: addressing the impact on mothers, fathers, and children. *Issues In Mental Health Nursing* 2012; **33**(7): 445-57. | Did not present original results |
| 139. | Lier-Schehl H, Turmes L, Pinnow M, El-Khechen W, Kramer M. Measuring mother-child relationship: a German instrument for both self-assessment and assessment by others (SF-MKI). *Praxis Der Kinderpsychologie Und Kinderpsychiatrie* 2011; **60**(3): 192-205. | Article not in English^a^ |
| 140. | Lilja G, Edhborg M, Nissen E. Depressive mood in women at childbirth predicts their mood and relationship with infant and partner during the first year postpartum. *Scandinavian Journal of Caring Sciences* 2012; **26**(2): 245-53. | No eligible bonding measure |
| 141. | Lim G, Farrell LM, Nam S, Wasan AD. Moderation-mediation effects between labor and postpartum pain, prenatal factors, and postpartum depression. *Journal of Women's Health* 2018; **27**(11): 1424-5. | Did not examine association between bonding and psychological distress |
| 142. | Lim SK. A behavioral intervention for mothers of colicky infants. US: ProQuest Information & Learning; 2014. | Did not examine association between bonding and psychological distress |
| 143. | Little CM. Maternal adaptation in women whose infants are born with a genetic anomaly or birth defect: Virginia Commonwealth University; 2008. | No eligible psychological distress measure |
| 144. | Little G, Bingham K, Anderson P, et al. Mental health for multiple birth families: An antenatal group for parents expecting multiples and a postnatal group for parents of multiples. *Twin Research and Human Genetics* 2017; **20**(6): 566. | No eligible bonding measure |
| 145. | Loughnan S. Regaining MUMentum: Findings from two randomized controlled trials evaluating brief internet cognitive behavioral therapy for perinatal distress, anxiety, and depression. *Archives of Women's Mental Health* 2019; **22**(5): 682. | Did not examine association between bonding and psychological distress |
| 146. | Loughnan SA, Sie A, Hobbs MJ, et al. A randomized controlled trial of 'MUMentum Pregnancy': Internet-delivered cognitive behavioral therapy program for antenatal anxiety and depression. *Journal of Affective Disorders* 2019; **243**: 381-90. | Did not examine association between bonding and psychological distress |
| 147. | Loughnan SAM, Newby J, Andrews G, Butler C. Regaining 'MUMentum': Randomized controlled trial of online CBT for perinatal distress, anxiety, and depression. *Archives of Women's Mental Health* 2019; **22**(5): 704. | No eligible bonding measure |
| 148. | Luoma I, Kaukonen P, Mäntymaa M, Puura K, Tamminen T, Salmelin R. A longitudinal study of maternal depressive symptoms, negative expectations and perceptions of child problems. *Child Psychiatry and Human Development* 2004; **35**(1): 37-53. | No eligible bonding measure |
| 149. | Luty SE, Kinley S. An adaptation of group interpersonal psychotherapy in the perinatal period. *Australian and New Zealand Journal of Psychiatry* 2016; **50**: 23-4. | Did not examine association between bonding and psychological distress |
| 150. | Macdonald JA, Youssef GJ, Phillips L, et al. The parental bonds of adolescent girls and next-generation maternal–infant bonding: findings from the Victorian Intergenerational Health Cohort Study. *Archives of Women's Mental Health* 2018; **21**(2): 171-80. | No eligible psychological distress measure |
| 151. | MacKie FL, Pattison H, Jankovic J, Morris RK, Kilby MD. Parental attachment and depressive symptoms in pregnancies complicated by twin-twin transfusion syndrome: A cohort study. *BMC Pregnancy and Childbirth* 2019; **20**(1). | Did not examine association between bonding and psychological distress |
| 152. | Mah BL, Van Ijzendoorn MH, Smith R, Bakermans-Kranenburg MJ. Oxytocin in postnatally depressed mothers: its influence on mood and expressed emotion. *Progress In Neuro-Psychopharmacology & Biological Psychiatry* 2013; **40**: 267-72. | No eligible bonding measure |
| 153. | Mahen HO, Ramchandani P, Imperial R, Halligan S. Developing a midwife-led, group supported guided self-help intervention for antenatal anxiety. *Archives of Women's Mental Health* 2015; **18**(2): 392. | Did not examine association between bonding and psychological distress |
| 154. | Maimburg RD, Væth M. Postpartum depression among first-time mothers - results from a parallel randomised trial. *Sexual and Reproductive Healthcare* 2015; **6**(2): 95-100. | Intervention study did not report separately for control group |
| 155. | Man C, Casillas E, Molas-Torreblanca K, Sapir H, Schrager S, Trost M. Screening for maternal postpartum depression during infant hospitalizations. *Journal of Investigative Medicine* 2015; **63**(1): 137-8. | Duplicate |
| 156. | Manurung S, Setyowati S, Ginanjar AS, Soesilo TEB, Tyastuti D. The Item Development of Maternal Blues Suryani (MBS) Scale in the antepartum period through bonding attachment that predicting postpartum blues. *Enfermeria Clinica* 2019; **29**: 752-9. | No eligible bonding measure |
| 157. | Martinez-Torteya C, Rosenblum KL, Beeghly M, Oppenheim D, Koren-Karie N, Muzik M. Maternal insightfulness protects against the detrimental effects of postpartum stress on positive parenting among at-risk mother-infant dyads. *Attachment & Human Development* 2018; **20**(3): 272-86. | No eligible bonding measure |
| 158. | Mason ZS. The role of maternal attachment: Its effects on postpartum depression and infant social-emotional development. US: ProQuest Information & Learning; 2010. | Duplicate |
| 159. | Matsunaga A, Takauma F, Tada K, Kitamura T. Discrete category of mother-to-infant bonding disorder and its identification by the Mother-to-Infant Bonding Scale: A study in Japanese mothers of a 1-month-old. *Early Human Development* 2017; **111**: 1-5. | Did not examine association between bonding and psychological distress |
| 160. | Matthey S, Speyer J. Changes in unsettled infant sleep and maternal mood following admission to a parentcraft residential unit. *Early Human Development* 2008; **84**(9): 623-9. | Did not examine association between bonding and psychological distress |
| 161. | Matvienko-Sikar K, Murphy G, Murphy M. The role of prenatal, obstetric, and post-partum factors in the parenting stress of mothers and fathers of 9-month old infants. *Journal of Psychosomatic Obstetrics and Gynecology* 2018; **39**(1): 47-55. | No eligible psychological distress measure |
| 162. | Mazúchová L, Kelčíková S, Porubská A, Malinovská N, Grendár M. Mother-infant bonding in the postpartum period and its predictors. *Central European Journal of Nursing & Midwifery* 2020; **11**(3): 121-9. | No eligible psychological distress measure |
| 163. | McDonald S, Slade P, Spiby H, Iles J. Post-traumatic stress symptoms, parenting stress and mother-child relationships following childbirth and at 2 years postpartum. *Journal of Psychosomatic Obstetrics and Gynecology* 2011; **32**(3): 141-6. | Bonding measured outside postnatal period |
| 164. | McErlean RA, Dadds MR, Austin MP. A randomised controlled trial on intranasal oxytocin as an adjunct to interaction coaching to improve maternal bonding in women with mild postpartum depression. *Biological Psychiatry* 2011; **69**(9): 134S. | Duplicate |
| 165. | McHaffie HE. Mothers of very low birthweight babies: how do they adjust? *Journal of Advanced Nursing* 1990; **15**(1): 6-11. | No eligible bonding measure |
| 166. | McKee MD, Zayas LH, Jankowski KRB. Breastfeeding intention and practice in an urban minority population: Relationship to maternal depressive symptoms and mother-infant closeness. *Journal of Reproductive and Infant Psychology* 2004; **22**(3): 167-81. | Did not examine association between bonding and psychological distress |
| 167. | McMahon C, Barnett B, Kowalenko N, Tennant C. Psychological factors associated with persistent postnatal depression: Past and current relationships, defence styles and the mediating role of insecure attachment style. *Journal of Affective Disorders* 2005; **84**(1): 15-24. | No eligible bonding measure |
| 168. | McMahon C, Trapolini T, Barnett B. Maternal state of mind regarding attachment predicts persistence of postnatal depression in the preschool years. *Journal of Affective Disorders* 2008; **107**(1-3): 199-203. | No eligible bonding measure |
| 169. | Mehler K, Hucklenbruch-Rother E, Trautmann-Villalba P, Becker I, Roth B, Kribs A. Delivery room skin-to-skin contact for preterm infants—A randomized clinical trial. *Acta Paediatrica, International Journal of Paediatrics* 2020; **109**(3): 518-26. | Did not examine association between bonding and psychological distress |
| 170. | Melnyk BM, Crean HF, Feinstein NF, Fairbanks E. Maternal anxiety and depression after a premature infant's discharge from the neonatal intensive care unit: explanatory effects of the creating opportunities for parent empowerment program. *Nursing Research* 2008; **57**(6): 383-94. | No eligible bonding measure |
| 171. | Meredith P, Noller P. Attachment and infant difficultiness in postnatal depression. *Journal of Family Issues* 2003; **24**(5): 668-86. | No eligible bonding measure |
| 172. | Mikoteit T, Brand S, Bürki N, Hösli I, Holsboer-Trachsler E. Postpartum depression: Infants' irritability is associated with mothers' parenting stress and poor postpartum bonding. *Swiss Medical Weekly* 2014; **144**: 11S. | Did not examine association between bonding and psychological distress |
| 173. | Mikoteit T, Brand S, Bürki N, Hösli I, Holsboer-Trachsler E. Prediction of postpartum depression by haircortisol and sleep-EEG. *Archives of Women's Mental Health* 2015; **18**(2): 405-6. | No eligible bonding measure |
| 174. | Mikoteit T, Brand S, Riecher-Rössler A, Holsboer-Trachsler E, Hatzinger M. Actigraphically measured poor sleep is related to cortisol awakening response and parenting stress in postpartum depression. *European Neuropsychopharmacology* 2012; **22**: S234. | Did not examine association between bonding and psychological distress |
| 175. | Milford R, Oates J. Universal screening and early intervention for maternal mental health and attachment difficulties. *Community Practitioner* 2009; **82**(8): 30-3. | No eligible bonding measure |
| 176. | Milgrom J. Mother-infant interactions in postpartum depression: an early intervention program. *The Australian Journal Of Advanced Nursing: A Quarterly Publication Of The Royal Australian Nursing Federation* 1994; **11**(4): 29-38. | Did not present original results |
| 177. | Milgrom J, Burrows GD, Snellen M, Stamboulakis W, Burrows K. Psychiatric illness in women: A review of the function of a specialist mother-baby unit. *Australian and New Zealand Journal of Psychiatry* 1998; **32**(5): 680-6. | No eligible bonding measure |
| 178. | Milgrom J, McCloud P. Parenting stress and postnatal depression. *Stress Medicine* 1996; **12**(3): 177-86. | No eligible bonding measure |
| 179. | Miller ML, O'Hara MW. Obsessive-compulsive symptoms, intrusive thoughts and depressive symptoms: a longitudinal study examining relation to maternal responsiveness. *Journal of Reproductive & Infant Psychology* 2020; **38**(3): 226-42. | No eligible bonding measure |
| 180. | Moayedoddin A, Moser D, Nanzer N. The impact of brief psychotherapy centred on parenthood on the anxio-depressive symptoms of mothers during the perinatal period. *Swiss Medical Weekly* 2013; **143**: w13769-w. | No eligible bonding measure |
| 181. | Morais AODdS, Simões VMF, Rodrigues LDS, et al. Maternal depressive symptoms and anxiety and interference in the mother/child relationship based on a prenatal cohort: an approach with structural equations modeling. *Cadernos De Saude Publica* 2017; **33**(6): e00032016-e. | Article not in English^a^ |
| 182. | Morisod-Harari M, Borghini A, Hohlfeld P, Forcada-Guex M, Muller-Nix C. Influence of prenatal hospitalization on parental stressful experience in the case of a premature birth. *Journal De Gynecologie, Obstetrique Et Biologie De La Reproduction* 2013; **42**(1): 64-70. | Article not in English^a^ |
| 183. | Moschner SL, Achtergarde S, Ramsauer B. Treatment Satisfaction of Mothers with Postpartum Depression Concerning Circle of Security Intervention. *Praxis Der Kinderpsychologie Und Kinderpsychiatrie* 2018; **67**(4): 351-66. | Article not in English^a^ |
| 184. | Mulcahy R, Reay RE, Wilkinson RB, Owen C. A randomised control trial for the effectiveness of group interpersonal psychotherapy for postnatal depression. *Archives of Women's Mental Health* 2010; **13**(2): 125-39. | Did not examine association between bonding and psychological distress |
| 185. | Müller M, Tronick E, Zietlow A-L, Nonnenmacher N, Verschoor S, Träuble B. Effects of Maternal Anxiety Disorders on Infant Self-Comforting Behaviors: The Role of Maternal Bonding, Infant Gender and Age. *Psychopathology* 2016; **49**(4): 295-304. | No eligible psychological distress measure |
| 186. | Müller ME. Prenatal and postnatal attachment: a modest correlation. *Journal Of Obstetric, Gynecologic, And Neonatal Nursing: JOGNN* 1996; **25**(2): 161-6. | No eligible psychological distress measure |
| 187. | Murray L. The development of children of postnatally depressed mothers: Evidence from the Cambridge longitudinal study. *Psychoanalytic Psychotherapy* 2009; **23**(3): 185-99. | Did not examine association between bonding and psychological distress |
| 188. | Murray L. The development of offspring of postnatally depressed mothers: Evidence from the Cambridge longitudinal study and implications for intervention. *Archives of Women's Mental Health* 2015; **18**(2): 307. | No eligible bonding measure |
| 189. | Murray L, Cooper PJ. The role of infant and maternal factors in postpartum depression, mother–infant interactions, and infant outcome. New York, NY, US: Guilford Press; 1997: 111-35. | No eligible bonding measure |
| 190. | Murray L, Cooper PJ, Wilson A, Romaniuk H. Controlled trial of the short- and long-term effect of psychological treatment of post-partum depression. 2. Impact on the mother--child relationship and child outcome. *The British Journal of Psychiatry* 2003; **182**(5): 420-7. | No eligible bonding measure |
| 191. | Muzik M. Maternal perinatal depression: Impact on infant emotion regulation and later toddler behavior problems. *Archives of Women's Mental Health* 2011; **14**: S107-S8. | No eligible bonding measure |
| 192. | Muzik M, Marcus SM, Flynn HA. Psychotherapeutic treatment options for perinatal depression: Emphasis on maternal-infant dyadic outcomes. *Journal of Clinical Psychiatry* 2009; **70**(9): 1318. | Did not present original results |
| 193. | Muzik M, McGinnis EW, Bocknek E, et al. PTSD symptoms across pregnancy and early postpartum among women with lifetime PTSD diagnosis *Depression and Anxiety* 2016; **33**(7): 584-91. | Did not examine association between bonding and psychological distress |
| 194. | Myersn S, Johns SE. Postnatal depression is associated with detrimental life-long and multigenerational impacts on relationship quality. *PeerJ* 2018; **2018**(2). | Bonding measured outside postnatal period |
| 195. | Nagata M, Nagai Y, Sobajima H, Ando T, Honjo S. Depression in the mother and maternal attachment - Results from a follow-up study at 1 year postpartum. *Psychopathology* 2003; **36**(3): 142-51. | Bonding measured outside postnatal period |
| 196. | Nair NS, Chen YS. Pilot survey of postnatal women at Jurong polyclinic. *Annals of the Academy of Medicine Singapore* 2010; **39**(11): S21. | No eligible bonding measure |
| 197. | Nath S, Pearson R, Moran P, et al. The impact of anxiety disorders in pregnancy on mother-infant interactions and bonding at 3 months postpartum. *Archives of Women's Mental Health* 2019; **22**(5): 662. | Duplicate |
| 198. | Nath S, Pearson R, Moran P, Pawlby S, Molyneaux E, Howard LM. The impact of personality dysfunction in pregnancy on mother-infant interactions and bonding at 3 months postpartum. *Archives of Women's Mental Health* 2019; **22**(5): 661. | Did not examine association between bonding and psychological distress |
| 199. | Nath S, Pearson RM, Moran P, Pawlby S, Molyneaux E, Howard LM. Maternal personality traits, antenatal depressive symptoms and the postpartum mother-infant relationship: a prospective observational study. *Social psychiatry and psychiatric epidemiology* 2020; **55**(5): 621-34. | Did not examine association between bonding and psychological distress |
| 200. | Neel ML, Hay K, Moore-Clingenpeel M, et al. Increasing synchrony of maternal/infant cortical responses as mothers increase sensory scaffolding during interactions. *Developmental Medicine and Child Neurology* 2019; **61**: 56-7. | Did not examine association between bonding and psychological distress |
| 201. | Newland RP, Parade SH, Dickstein S, Seifer R. Goodness of fit between prenatal maternal sleep and infant sleep: Associations with maternal depression and attachment security. *Infant Behavior & Development* 2016; **44**: 179-88. | Bonding measured outside postnatal period |
| 202. | Nieto L, Lara MA, Navarrete L. Prenatal Predictors of Maternal Attachment and Their Association with Postpartum Depressive Symptoms in Mexican Women at Risk of Depression. *Maternal And Child Health Journal* 2017; **21**(6): 1250-9. | Intervention study did not report separately for control group |
| 203. | Nishigori H, Obara T, Nishigori T, et al. Mother-to-infant bonding failure and intimate partner violence during pregnancy as risk factors for father-to-infant bonding failure at 1 month postpartum: an adjunct study of the Japan Environment and Children’s Study. *Journal of Maternal-Fetal and Neonatal Medicine* 2020; **33**(16): 2789-96. | Did not examine association between bonding and psychological distress |
| 204. | Nishioka E, Haruna M, Ota E, et al. A prospective study of the relationship between breastfeeding and postpartum depressive symptoms appearing at 1–5months after delivery. *Journal of Affective Disorders* 2011; **133**(3): 553-9. | No eligible bonding measure |
| 205. | Noorlander Y, Bergink V, Van Den Berg MP. Perceived and observed mother-child interaction at time of hospitalization and release in postpartum depression and psychosis. *Archives of Women's Mental Health* 2008; **11**(1): 49-56. | Did not examine association between bonding and psychological distress |
| 206. | Norris S, Ballen N, Gandhi J, Sutherland K. Psychosocial interventions offered at the Ottawa perinatal mental health program and their impact on the maternal-infant dyad. *Archives of Women's Mental Health* 2013; **16**: S54-S5. | No eligible bonding measure |
| 207. | Ntaouti E, Gonidakis F, Nikaina E, et al. Maternity blues: risk factors in Greek population and validity of the Greek version of Kennerley and Gath’s blues questionnaire. *Journal of Maternal-Fetal and Neonatal Medicine* 2020; **33**(13): 2253-62. | No eligible bonding measure |
| 208. | Ohiggins M. Improving mother-infant outcomes after postnatal depression. US: ProQuest Information & Learning; 2018. | Duplicate |
| 209. | Okano T, Kokubu M, Sugiyama T. A longitudinal study of effect of postnatal depression onmaternal bonding and attitudes towards pregnancy. *Archives of Women's Mental Health* 2013; **16**: S132. | Duplicate |
| 210. | Oyelohunnu MA, Campbell EA, Eigbike M, Oshodi YO. Maternal-child attachment and psychiatric morbidity among mothers attending routine community health clinics in Southwest Nigeria. *Neuropsychiatrie de l'Enfance et de l'Adolescence* 2012; **60**(5): S135. | Article not in English^a^ |
| 211. | Özcan NK, Boyacıoğlu NE, Dikeç G, Dinç H, Enginkaya S, Tomruk N. Prenatal and postnatal attachment among Turkish mothers diagnosed with a mental health disorder. *Issues in Mental Health Nursing* 2018; **39**(9): 795-801. | No eligible psychological distress measure |
| 212. | Palacios-Hernández B. Comparative study of the factors involved in the mother-infant bonding in women with and without postpartum depression. 2015. | Duplicate |
| 213. | Palacios-Hernández B, Torres A, Lasheras G, et al. Mother-infant bonding in women with postpartum depression. *Archives of Women's Mental Health* 2015; **18**(2): 380. | Duplicate |
| 214. | Pascoe JM, French J. Development of positive feelings in primiparous mothers toward their normal newborns: a descriptive study. *Clinical Pediatrics* 1989; **28**(10): 452-6. | No eligible bonding measure |
| 215. | Pearce H, Ayers S. The expected child versus the actual child: Implications for the mother-baby bond. *Journal of Reproductive and Infant Psychology* 2005; **23**(1): 89-102. | No eligible bonding measure |
| 216. | Pearlman C. Parenting an infant after prolonged infertility: Maternal depression, anxiety, confidence, enjoyment, and bonding as well as infant sleep quality and routine, and infant crying. *Fertility and Sterility* 2013; **100**(3): S25-S6. | Did not examine association between bonding and psychological distress |
| 217. | Pearson R, Lightman S, Evans J. Attentional processing of infant emotion during pregnancy and how it is related to depressive symptoms and mother-infant relationships after birth. *Archives of Women's Mental Health* 2013; **16**: S38. | Duplicate |
| 218. | Perry DF, Ettinger AK, Mendelson T, Le H-N. Prenatal depression predicts postpartum maternal attachment in low-income Latina mothers with infants. *Infant Behavior & Development* 2011; **34**(2): 339-50. | Intervention study did not report separately for control group |
| 219. | Persico G, Antolini L, Vergani P, Costantini W, Nardi MT, Bellotti L. Maternal singing of lullabies during pregnancy and after birth: Effects on mother-infant bonding and on newborns' behaviour. Concurrent Cohort Study. *Women And Birth: Journal Of The Australian College Of Midwives* 2017; **30**(4): e214-e20. | No eligible psychological distress measure |
| 220. | Petri E, Bacci O, Palagini L, et al. Early screening during pregnancy for maternal psychopathology reduced post partum depressive symptoms and improved mother-infant attachment. *European Neuropsychopharmacology* 2017; **27**: S804-S5. | Did not examine association between bonding and psychological distress |
| 221. | Pineda M, Jones NA. Kangaroo care with full-term infants: Maternal behaviors and mother-infant bonding. 2014; **1527809**: 55. | No eligible psychological distress measure |
| 222. | Pisoni C, Spairani S, Fauci F, et al. Effect of maternal psychopathology on neurodevelopmental outcome and quality of the dyadic relationship in preterm infants: an explorative study. *Journal of Maternal-Fetal and Neonatal Medicine* 2020; **33**(1): 103-12. | No eligible bonding measure |
| 223. | Popo E, Kenyon S, Dann S-A, MacArthur C, Blissett J. Effects of lay support for pregnant women with social risk factors on infant development and maternal psychological health at 12 months postpartum. *Plos One* 2017; **12**(8): e0182544-e. | Did not examine association between bonding and psychological distress |
| 224. | Posmontier B, Neugebauer R, Stuart S, Chittams J, Shaughnessy R. Telephone‐administered interpersonal psychotherapy by nurse‐midwives for postpartum depression. *Journal of Midwifery & Women's Health* 2016; **61**(4): 456-66. | Duplicate |
| 225. | Posmontier B, Stuart S, Neugebauer R, Shaughnessy R. Multidisciplinary model of nurse midwife administered psychotherapy for postpartum depression. *Archives of Women's Mental Health* 2013; **16**: S7. | Did not examine association between bonding and psychological distress |
| 226. | Powers KL, Singh-Carlson S. Real-time video streaming: Impact on maternal anxiety and the maternal-infant bond. 2012; **1517537**: 55. | Qualitative only study |
| 227. | Quinlivan JA, Evans SF. Impact of domestic violence and drug abuse in pregnancy on maternal attachment and infant temperament in teenage mothers in the setting of best clinical practice. *Archives of Women's Mental Health* 2005; **8**(3): 191-9. | Did not examine association between bonding and psychological distress |
| 228. | Raoul-Duval A, Bertrand-Servais M, Frydman R. Comparative prospective study of the psychological development of children born by in vitro fertilization and their mothers. *Journal of Psychosomatic Obstetrics and Gynaecology* 1993; **14**(2): 117-26. | No eligible bonding measure |
| 229. | Razurel C, Antonietti J-P, Rulfi F, Pasquier N, Domingues-Montanari S, Darwiche J. The impact of pre- and post-natal psycho-educational intervention on the construction of parenthood. *Archives Of Women's Mental Health* 2017; **20**(3): 469-72. | Intervention study did not report separately for control group |
| 230. | Reck C, Tietz A, Müller M. On the influence of postpartum anxiety disorders on mother-child interaction and child development. *Archives of Women's Mental Health* 2013; **16**: S99. | Duplicate |
| 231. | Reilly N, Brake E, Briggs N, Austin MP. Trajectories of clinical and parenting outcomes following admission to an inpatient mother-baby unit. *BMC Psychiatry* 2019; **19**(1). | Did not examine association between bonding and psychological distress |
| 232. | Ricbourg A, Gosme C, Gayat E, Ventre C, Barranger E, Mebazaa A. Emotional impact of severe post-partum haemorrhage on women and their partners: An observational, case-matched, prospective, single-centre pilot study. *European Journal of Obstetrics Gynecology and Reproductive Biology* 2015; **193**: 140-3. | Did not examine association between bonding and psychological distress |
| 233. | Righetti-Veltema M, Conne-Perréard E, Bousquet A, Manzano J. Postpartum depression and mother-infant relationship at 3 months old. *Journal of Affective Disorders* 2002; **70**(3): 291-306. | No eligible bonding measure |
| 234. | Roberts S, Branjerdporn G, Henderson K. Clinical outcomes of mothers with severe mental illness admitted to Queensland's first public mother-baby unit. *Journal of Paediatrics and Child Health* 2019; **55**: 10. | Did not examine association between bonding and psychological distress |
| 235. | Robinson MP. The influence of maternal and child factors on maternal depression and quality of interaction and toddler mastery behaviors and interactive play maturity. US: ProQuest Information & Learning; 1993. | Full text not available |
| 236. | Robson KM, Kumar R. Delayed onset of maternal affection after childbirth. *British Journal of Psychiatry* 1980; **136**(4): 347-53. | No eligible bonding measure |
| 237. | Rossen L, Mattick RP, Wilson J, et al. Mother–Infant and Partner–Infant Emotional Availability at 12 Months of Age: Findings From an Australian Longitudinal Study. *Infancy* 2018; **23**(6): 893-916. | Did not examine association between bonding and psychological distress |
| 238. | Rowe H, Sperlich M, Cameron H, Seng J. A quasi‐experimental outcomes analysis of a psychoeducation intervention for pregnant women with abuse‐related posttraumatic stress. *Journal of Obstetric, Gynecologic, & Neonatal Nursing: Clinical Scholarship for the Care of Women, Childbearing Families, & Newborns* 2014; **43**(3): 282-93. | Did not examine association between bonding and psychological distress |
| 239. | Rutherford HJV, Potenza MN, Mayes LC, Scheinost D. The Application of Connectome-Based Predictive Modeling to the Maternal Brain: Implications for Mother-Infant Bonding. *Cerebral Cortex* 2020; **30**(3): 1538-47. | Did not examine association between bonding and psychological distress |
| 240. | Salomonsson B, Sandell R. Maternal experiences and the mother-infant dyad's development: Introducing the Interview of Mother's Experiences (I-ME). *Journal of Reproductive and Infant Psychology* 2012; **30**(1): 21-50. | No eligible bonding measure |
| 241. | Sargent SP. Prepartum maternal attitudes, neonatal characteristics, and postpartum adaptation of mother and infant. US: ProQuest Information & Learning; 1977. | Full text not available |
| 242. | Schlesinger Y, Hamiel D, Rousseau S, et al. Preventing risk for posttraumatic stress following childbirth: Visual biofeedback during childbirth increases maternal connectedness to her newborn thereby preventing risk for posttraumatic stress following childbirth. *Psychological Trauma: Theory, Research, Practice, and Policy* 2020. | No eligible bonding measure |
| 243. | Scott D. Nursing the impaired mother-infant relationship in puerperal depression. *The Australian Journal Of Advanced Nursing: A Quarterly Publication Of The Royal Australian Nursing Federation* 1984; **1**(4): 50-6. | Did not present original results |
| 244. | Seah CKF, Morawska A. When mum is stressed, is dad just as stressed? Predictors of paternal stress in the first six months of having a baby. *Infant Mental Health Journal* 2016; **37**(1): 45-55. | Did not examine association between bonding and psychological distress |
| 245. | Seeley S, Murray L, Cooper PJ. Postnatal depression: the outcome for mothers and babies of health visitor intervention. *Health Visitor* 1996; **69**(4): 135-8. | No eligible bonding measure |
| 246. | Seymour M, Giallo R, Cooklin A, Dunning M. Maternal anxiety, risk factors and parenting in the first post-natal year. *Child: Care, Health And Development* 2015; **41**(2): 314-23. | No eligible bonding measure |
| 247. | Shaw RJ, Sweester CJ, St. John N, et al. Prevention of Postpartum Traumatic Stress in Mothers with Preterm Infants: Manual Development and Evaluation. *Issues in Mental Health Nursing* 2013; **34**(8): 578-86. | No eligible bonding measure |
| 248. | Shin H, Park Y-J, Kim MJ. Predictors of maternal sensitivity during the early postpartum period. *Journal Of Advanced Nursing* 2006; **55**(4): 425-34. | No eligible psychological distress measure |
| 249. | Shishido E, Shuo T, Takahata K, Horiuchi S. Changes in salivary oxytocin levels and bonding disorder in women from late pregnancy to early postpartum: A pilot study. *PLoS ONE* 2019; **14**(9). | Did not examine association between bonding and psychological distress |
| 250. | Sidor A, Cierpka M. The Family Questionnaire (FB-K) - A Short Version of the General Family Questionnaire and its Reliability and Validity. *Praxis Der Kinderpsychologie Und Kinderpsychiatrie* 2016; **65**(1): 40-56. | Article not in English^a^ |
| 251. | Sidor A, Thiel-Bonney C, Kunz E, Eickhorst A, Cierpka M. Persistent, excessive crying in 5-month-old infants and the pre-, peri- and postnatal adversities of their mothers in a high-risk sample. *Zeitschrift Fur Kinder- Und Jugendpsychiatrie Und Psychotherapie* 2012; **40**(4): 239-50. | Article not in English^a^ |
| 252. | Siu BWM, Ip P, Chow HMT, et al. Impairment of mother-infant relationship: validation of the Chinese version of Postpartum Bonding Questionnaire. *The Journal Of Nervous And Mental Disease* 2010; **198**(3): 174-9. | Did not examine association between bonding and psychological distress |
| 253. | Slade P, Emerson DJM, Freedlander E. A longitudinal comparison of the psychological impact on mothers of neonatal and 3 month repair of cleft lip. *British Journal of Plastic Surgery* 1999; **52**(1): 1-5. | No eligible bonding measure |
| 254. | Soubieux MJ. Psychological impact of antenatal diagnosis of increased nuchal translucency a woman's pregnancy experience and early mother-child relationship. *Journal De Gynecologie, Obstetrique Et Biologie De La Reproduction* 2005; **34**(1 Suppl): S103-S9. | Article not in English^a^ |
| 255. | Sousa Albuquerque CM, Lourenc¸o SA, Amante MJ. Psycho-pathological disorders in teenage mothers: Implications in the process of mother-child bond. *European Psychiatry* 2012; **27**. | Did not examine association between bonding and psychological distress |
| 256. | Sperlich M, Rowe H, Cameron H, Seng J. The survivor moms companion: Assessing the effect on posttraumatic stress during pregnancy. *Archives of Women's Mental Health* 2013; **16**: S25. | Duplicate |
| 257. | Sperlich MI. Trauma exposure, posttraumatic stress, and depression in a community sample of first-time mothers. US: ProQuest Information & Learning; 2015. | Did not examine association between bonding and psychological distress |
| 258. | Spieker SJ, Gillmore MR, Lewis SM, Morrison DM, Lohr MJ. Psychological distress and substance use by adolescent mothers: Associations with parenting attitudes and the quality of mother–child interaction. *Journal of Psychoactive Drugs* 2001; **33**(1): 83-93. | No eligible bonding measure |
| 259. | Spinelli MG, Endicott J. Controlled clinical trial of antepartum interpersonal psychotherapy versus parenting education program at 3 NYC sites. *Archives of Women's Mental Health* 2011; **14**: S49. | No eligible bonding measure |
| 260. | Suzuki S, Sekizawa A, Tanaka M, Okai T, Kinoshita K. Current status of women requiring perinatal mental health care for protecting their children in Japan. *Asian Journal of Psychiatry* 2016; **22**: 93-. | Did not present original results |
| 261. | Sved-Williams A, Yelland C, Girke T, Hollamby S, Braithwaite K. Women with borderline personality disorder (BPD) and their infants in an MBU. *Archives of Women's Mental Health* 2015; **18**(2): 378-9. | Did not examine association between bonding and psychological distress |
| 262. | Szpunar M, Nguyen T, Early J, Hammer M, Reminick A. Incidence of synthetic oxytocin administration and time to improvement of depression and anxiety symptoms in the UCSD intensive outpatient treatment program for postpartum depression. *Biological Psychiatry* 2018; **83**(9): S185. | Did not examine association between bonding and psychological distress |
| 263. | Tabrizi FM, Nournezhad H. Investigating the Effect of Counseling Based on Family Support on Mother-Child Bonding at 4-6 Weeks of Postpartum in Primiparous Women: A Randomized Clinical Trial. *International Journal of Pediatrics (2345-5047)* 2018; **6**(9): 8261-73. | Did not examine association between bonding and psychological distress |
| 264. | Tandberg BS, Flacking R, Markestad T, Grundt H, Moen A. Parent psychological wellbeing in a single-family room versus an open bay neonatal intensive care unit. *PLoS ONE* 2019; **14**(11). | Did not examine association between bonding and psychological distress |
| 265. | Taylor A, O'Higgins M, St James Roberts I, Glover V. The relationship between postnatal depression and mother-infant bonding over the first year of life. *Archives of Women's Mental Health* 2013; **16**: S141. | Duplicate |
| 266. | Tester-Jones MC. The role of rumination in the relationship between postnatal depressive symptoms and maternal attunement. 2014; **10091837**. | Duplicate |
| 267. | Thomas N, Komiti A, Judd F. 'Pilot early intervention antenatal group program for pregnant women with anxiety and depression': Erratum. *Archives of Women's Mental Health* 2014; **17**(6): 601-. | No eligible bonding measure |
| 268. | Tikotzky L, Chambers AS, Kent J, Gaylor E, Manber R. Postpartum maternal sleep and mothers' perceptions of their attachment relationship with the infant among women with a history of depression during pregnancy. *International Journal of Behavioral Development* 2012; **36**(6): 440-8. | Intervention study did not report separately for control group |
| 269. | Toosi M, Akbarzadeh M, Ghaemi Z. The Effect of Relaxation on Mother's Anxiety and Maternal-Fetal Attachment in Primiparous IVF Mothers. *Journal Of The National Medical Association* 2017; **109**(3): 164-71. | No eligible bonding measure |
| 270. | Topan A, Demirel S, Alkan I, Ayyıldız TK, Doğru S. Evaluation of Newborn Mothers' Status of Bonding to the Baby, Experiencing Depression, and Associated Factors. *Medical Journal of Bakirkoy* 2019; **15**(2): 160-9. | Did not examine association between bonding and psychological distress |
| 271. | Townsend ML, Brassel AK, aafi M, Grenyer BFS. Childbirth satisfaction and perceptions of control: postnatal psychological implications. *British Journal of Midwifery* 2020; **28**(4): 225-33. | Did not examine association between bonding and psychological distress |
| 272. | Trautmann-Villalba P, Hornstein C. Different aspects of peripartum psychiatry and psychotherapy (symposium of the German-speaking group of the marcé society). *Archives of Women's Mental Health* 2013; **16**: S81. | Duplicate |
| 273. | Trost MJ, Molas-Torreblanca K, Man C, Casillas E, Sapir H, Schrager SM. Screening for maternal postpartum depression during infant hospitalizations. *Journal of Hospital Medicine* 2016; **11**(12): 840-6. | Intervention study did not report separately for control group |
| 274. | Tsivos Z-L, Calam R, Sanders MR, Wittkowski A. A pilot randomised controlled trial to evaluate the feasibility and acceptability of the Baby Triple P Positive Parenting Programme in mothers with postnatal depression. *Clinical Child Psychology & Psychiatry* 2015; **20**(4): 532-54. | Did not examine association between bonding and psychological distress |
| 275. | Tsuchiya S, Tsuchiya M, Momma H, et al. Association of cleft lip and palate on mother-to-infant bonding: A cross-sectional study in the Japan Environment and Children's Study (JECS). *BMC Pediatrics* 2019; **19**(1). | Did not examine association between bonding and psychological distress |
| 276. | Turner KE, Berg-Cross L, So D. Breastfeeding Behaviors as a Function of Sleep Deprivation, Bonding, Depression, and Social Support. 2013; **1553201**: 67. | Did not examine association between bonding and psychological distress |
| 277. | Unternaehrer E, Cost KT, Bouvette-Turcot AA, et al. Dissecting maternal care: Patterns of maternal parenting in a prospective cohort study. *Journal of Neuroendocrinology* 2019; **31**(9). | No eligible bonding measure |
| 278. | van Doesum KTM, Riksen-Walraven JM, Hosman CMH, Hoefnagels C. A randomized controlled trial of a home-visiting intervention aimed at preventing relationship problems in depressed mothers and their infants. *Child Development* 2008; **79**(3): 547-61. | No eligible bonding measure |
| 279. | Van Lieshout RJ, Yang L, Haber E, Ferro MA. Evaluating the effectiveness of a brief group cognitive behavioural therapy intervention for perinatal depression. *Archives of Women's Mental Health* 2017; **20**(1): 225-8. | Did not examine association between bonding and psychological distress |
| 280. | Van Rij B, Wierdsma A, Bergink V, Koorengevel KM. Well-being of spouses from mothers admitted at the psychiatric mother-baby unit. *Archives of Women's Mental Health* 2015; **18**(2): 315. | Did not examine association between bonding and psychological distress |
| 281. | Veddovi M, Gibson F, Kenny DT, Bowen J, Starte D. Preterm behavior, maternal adjustment, and competencies in the newborn period: What influence do they have at 12 months postnatal age? *Infant Mental Health Journal* 2004; **25**(6): 580-99. | No eligible bonding measure |
| 282. | Vengadavaradan A, Bharadwaj B, Sathyanarayanan G, Durairaj J. Frequency and correlates of mother-infant bonding disorders among postpartum women in India. *Asian Journal of Psychiatry* 2019; **44**: 72-9. | Did not examine association between bonding and psychological distress |
| 283. | Viaux S, Maurice P, Cohen D, Jouannic JM. Giving birth under lockdown during the COVID-19 epidemic. *Journal of Gynecology Obstetrics and Human Reproduction* 2020; **49**(6). | Did not examine association between bonding and psychological distress |
| 284. | Viveros MP. Maternal separation, mother-infant bonding and postpartum depression. *Archives of Women's Mental Health* 2015; **18**(2): 379. | Did not examine association between bonding and psychological distress |
| 285. | Vliegen N, Casalin S, Luyten P, et al. Hospitalization-based treatment for postpartum depressed mothers and their babies: Rationale, principles, and preliminary follow-up data. *Psychiatry (New York)* 2013; **76**(2): 150-68. | No eligible bonding measure |
| 286. | Vliegen N, Luyten P, Biringen Z. A multimethod perspective on emotional availability in the postpartum period. *Parenting: Science & Practice* 2009; **9**(3/4): 228-43. | No eligible bonding measure |
| 287. | Weidner K. Traumatic birth consequences and treatment: Experiencesfrom the mother baby unit at Dresden University. *Archives of Women's Mental Health* 2019; **22**(5): 655. | Bonding measured outside postnatal period |
| 288. | Weis K, Lederman RP. Prenatal spousal military deployment and maternal prenatal adaptation as predictors of postpartum maternal-infant attachment. *Southern Online Journal of Nursing Research* 2010; **10**(3): 7p-p. | No eligible bonding measure |
| 289. | Wenderlein JM, Wilhelm RM. Low birth weight is a negative factor in the mother-infant relationship. . *Geburtshilfe Und Frauenheilkunde* 1982; **42**(6): 450-2. | Article not in English^a^ |
| 290. | Williams C. Posttraumatic stress following childbirth and maternal perceptions of the mother-infant bond: The role of attachment experiences and metacognition. 2012; **U608488**: 1. | Duplicate |
| 291. | Yalçin SS, Orün E, Mutlu B, et al. Why are they having infant colic? A nested case-control study. *Paediatric And Perinatal Epidemiology* 2010; **24**(6): 584-96. | Did not examine association between bonding and psychological distress |
| 292. | Yalçin SS, Örün E, Yalçn S, Aykut O. Organochlorine pesticide residues in breast milk and maternal psychopathologies and infant growth from suburban area of Ankara, Turkey. *International Journal of Environmental Health Research* 2015; **25**(4): 364-72. | Did not examine association between bonding and psychological distress |
| 293. | Yamashita H, Yoshida K. Impact of perinatal stress onmother-infant interaction: Relationship between intimate partner violence and maternal bonding failure. *Archives of Women's Mental Health* 2013; **16**: S104. | Did not examine association between bonding and psychological distress |
| 294. | Yamashita H, Yoshida K. Impact of perinatal stress on mother-infant interaction: Mother-infant interaction and infant outcome in mothers with mood and/or anxiety disorder. *Archives of Women's Mental Health* 2013; **16**: S145. | Did not examine association between bonding and psychological distress |
| 295. | Yamashita H, Yoshida K, Kanba S. Maternal attachment style, mother-infant interaction and their toddlers' socio-emotional outcome. *Archives of Women's Mental Health* 2015; **18**(2): 326-7. | Did not examine association between bonding and psychological distress |
| 296. | Yelland C, Girke T, Tottman C, Williams AS. Clinical characteristics and mental health outcomes for women admitted to an Australian Mother-Baby Unit: a focus on borderline personality disorder and emotional dysregulation? *Australasian Psychiatry: Bulletin Of Royal Australian And New Zealand College Of Psychiatrists* 2015; **23**(6): 683-7. | Did not examine association between bonding and psychological distress |
| 297. | Yoshida K, Yamashita H. Links between disturbance of mother to infant bonding and early maternal mood in the mothers who received postpartum home visit service from the nationwide cross survey in Japan. *Archives of Women's Mental Health* 2015; **18**(2): 352. | Duplicate |
| 298. | Young SM, Gryder LK, Cross C, Zava D, Kimball DW, Benyshek DC. Placentophagy's effects on postpartum maternal affect, health, and recovery. *American Journal of Physical Anthropology* 2017; **162**: 418. | Did not examine association between bonding and psychological distress |
| 299. | Young SM, Gryder LK, Cross C, Zava D, Kimball DW, Benyshek DC. Placentophagy's effects on mood, bonding, and fatigue: A pilot trial, part 2. *Women and Birth* 2018; **31**(4): e258-e71. | Duplicate |
| 300. | Zanardo V, Bertin M, Sansone L, Felice L. The adaptive psychological changes of elective induction of labor in breastfeeding women. *Early Human Development* 2017; **104**: 13-6. | Did not examine association between bonding and psychological distress |
| 301. | Zeinali S, Mazeheri MA, Sadeghi MS, Jabari M. The relationships of mother's attachment to infant and mothers' psychological characteristics to feeding problems in infants. *Developmental Psychology: Journal of Iranian Psychologists* 2011; **8**(29): 55-66. | Article not in English^a^ |

*Note*: ^a^Research team did not have access to translation services and so articles not available in English were excluded.

eTable 4. Included articles by year of publication.

| Year/s | Number of publications  (n = 133) | % | Cumulative % |
| --- | --- | --- | --- |
| 1988 | 1 | 0.75 | 0.75 |
| 1989 | 1 | 0.75 | 1.50 |
| 1990 | 1 | 0.75 | 2.26 |
| 1991 - 1993 | 0 | 0.00 | 2.26 |
| 1994 | 1 | 0.75 | 3.01 |
| 1995 - 1997 | 0 | 0.00 | 3.01 |
| 1998 | 1 | 0.75 | 3.76 |
| 1999 | 1 | 0.75 | 4.51 |
| 2000 | 1 | 0.75 | 5.26 |
| 2001 - 2003 | 0 | 0.00 | 5.26 |
| 2004 | 4 | 3.01 | 8.27 |
| 2005 | 2 | 1.50 | 9.77 |
| 2006 | 2 | 1.50 | 11.28 |
| 2007 | 2 | 1.50 | 12.78 |
| 2008 | 1 | 0.75 | 13.53 |
| 2009 | 2 | 1.50 | 15.04 |
| 2010 | 2 | 1.50 | 16.54 |
| 2011 | 10 | 7.52 | 24.06 |
| 2012 | 4 | 3.01 | 27.07 |
| 2013 | 8 | 6.02 | 33.08 |
| 2014 | 8 | 6.02 | 39.10 |
| 2015 | 10 | 7.52 | 46.62 |
| 2016 | 16 | 12.03 | 58.65 |
| 2017 | 10 | 7.52 | 66.17 |
| 2018 | 8 | 6.02 | 72.18 |
| 2019 | 18 | 13.53 | 85.71 |
| 2020 | 19 | 14.29 | 100 |

eTable 5. Meta-regression analyses of various moderators on overall meta-analysis results.

| Moderator | k | n | *r* | 95% CIs | I^2^ | *Tau^2^* | *p* |
| --- | --- | --- | --- | --- | --- | --- | --- |
| **Sample type** | 96 | 365 |  |  | 97.08 | 0.07 | .58 |
| Clinical | 4 | 9 | 0.37 | (-0.13, 0.72) | 94.31 | 0.09 |  |
| Community | 79 | 304 | 0.37 | (0.33, 0.40) | 97.37 | 0.07 |  |
| Mixed (clinical  & community) | 7 | 43 | 0.45 | (0.32, 0.57) | 94.53 | 0.05 |  |
| Parents of infants in  Neonatal care | 6 | 9 | 0.34 | (0.16, 0.50) | 75.02 | 0.02 |  |
| Parents of twins | NA | NA | NA | NA | NA | NA |  |
| Pairwise comparisons |  |  |  |  |  |  | NA |
| **Parity** | 83 | 313 |  |  | 97.41 | 0.08 | .375 |
| Mixed | 71 | 289 | 0.36 | (0.32, 0.40) | 97.70 | 0.08 |  |
| Primipara | 12 | 24 | 0.42 | (0.27, 0.54) | 89.72 | 0.06 |  |
| Multipara | NA | NA | NA | NA | NA | NA |  |
|  |  |  |  |  |  |  |  |
| **Article type** | 99 | 368 |  |  | 97.25 | 0.07 | .653 |
| Published | 86 | 327 | 0.38 | (0.34, 0.41) | 97.55 | 0.08 |  |
| Conference abstract | 2 | 2 | 0.48 | (-0.70, 0.96) | 47.06 | 0.01 |  |
| Thesis | 11 | 39 | 0.35 | (0.25, 0.43) | 65.21 | 0.02 |  |
| Pairwise comparisons |  |  |  |  |  |  | NA |
| **Article type** | 99 | 368 |  |  | 97.23 | 0.07 | .831 |
| Published | 86 | 327 | 0.38 | (0.34, 0.41) | 97.55 | 0.08 |  |
| Grey | 13 | 41 | 0.37 | (0.28, 0.45) | 65.67 | 0.02 |  |
| Pairwise comparisons |  |  |  |  |  |  | NA |
| **Country income**  **classification** | 98 | 367 |  |  | 97.15 | 0.07 | .19 |
| HIC | 88 | 347 | 0.37 | (0.33, 0.40) | 97.34 | 0.07 |  |
| LMIC | 3 | 4 | 0.48 | (0.18, 0.70) | 80.51 | 0.02 |  |
| UMIC | 7 | 16 | 0.39 | (0.22, 0.54) | 89.52 | 0.05 |  |
| Pairwise comparisons |  |  |  |  |  |  | NA |
| **Bonding measure used**  **(Overall)** | 95 | 333 |  |  | 91.61 | 0.04 | .026 |
| MPAS | 19 | 71 | 0.42 | (0.32, 0.52) | 87.58 | 0.03 |  |
| PBQ | 48 | 147 | 0.43 | (0.38, 0.49) | 93.05 | 0.05 |  |
| PBQ-GER | 7 | 15 | 0.40 | (0.24, 0.56) | 86.93 | 0.03 |  |
| MIBS | 11 | 57 | 0.25 | (0.2, 0.31) | 48.67 | 0.01 |  |
| MIBS-J | 6 | 26 | 0.27 | (0.1, 0.43) | 95.98 | 0.05 |  |
| MAI | 6 | 11 | 0.48 | (0.24, 0.71) | 88.52 | 0.04 |  |
| MAS | 3 | 6 | 0.38 | (0.19, 0.57) | 59.27 | 0.01 |  |
| Pairwise comparisons | MIBS < MPAS; MIBS < PBQ | | | | | | |
| **Bonding measure used**  **(Depression)** | 90 | 216 |  |  | 91.66 | 0.04 | .045 |
| MPAS | 18 | 38 | 0.43 | (0.34, 0.53) | 85.46 | 0.03 |  |
| PBQ | 44 | 103 | 0.45 | (0.39, 0.51) | 93.11 | 0.05 |  |
| PBQ-GER | 7 | 9 | 0.44 | (0.27, 0.61) | 89.4 | 0.04 |  |
| MIBS | 10 | 35 | 0.27 | (0.21, 0.33) | 45.44 | 0.01 |  |
| MIBS-J | 6 | 18 | 0.28 | (0.11, 0.45) | 96.31 | 0.05 |  |
| MAI | 6 | 7 | 0.46 | (0.21, 0.72) | 90.05 | 0.05 |  |
| MAS | 3 | 6 | 0.38 | (0.19, 0.57) | 59.27 | 0.01 |  |
| Pairwise comparisons | MIBS < MPAS; MIBS < PBQ | | | | | | |
| **Bonding measure used**  **(Anxiety)** | 25 | 66 |  |  | 79.34 | 0.02 | .87 |
| MPAS | 6 | 22 | 0.24 | (0.1, 0.38) | 82.85 | 0.01 |  |
| PBQ | 10 | 16 | 0.34 | (0.23, 0.45) | 84.11 | 0.02 |  |
| PBQ-GER | 3 | 6 | 0.3 | (0.17, 0.43) | 0 | 0 |  |
| MIBS | 4 | 13 | 0.23 | (0.1, 0.36) | 58.56 | 0.01 |  |
| MAI | 2 | 3 | 0.24 | (-0.23, 0.72) | 55.75 | 0.01 |  |
| Pairwise comparisons |  |  |  |  |  |  | NA |
| *Note*: k = number of included studies; n = number of included effects; NA = insufficient effect sizes for analysis; NS = not significant. | | | | | | | |

**Forest plots and tables for overall meta-analyses.**

The following series of forest plots and tables relates to the meta-analyses in Table 1 of the manuscript. Please note, several meta-analyses included a substantial number of effect size coefficients (up to 368) and therefore could not be meaningfully displayed in a forest plot figure. Forest plot data for these large meta-analyses are instead presented below in table format.

eTable 6. Forest plot data for overall meta-analysis of all included effects.

| Study | Effect size descriptor | N | r | Fisher Z | Fisher Z variance | Standard error |
| --- | --- | --- | --- | --- | --- | --- |
| Agbagwara-Osuji 2015 | State anxiety: STAI, Time=PG3; Bonding: MIBS (Total), Time=1 | 223 | .200 | 0.203 | 0.005 | 0.067 |
| Agbagwara-Osuji 2015 | Trait anxiety: STAI, Time=PG3; Bonding: MIBS (Total), Time=1 | 223 | .250 | 0.255 | 0.005 | 0.067 |
| Agbagwara-Osuji 2015 | Depression: EPDS, Time=PG3; Bonding: MIBS (Total), Time=1 | 223 | .250 | 0.255 | 0.005 | 0.067 |
| Agbagwara-Osuji 2015 | Stress: SLES, Time=PG3; Bonding: MIBS (Total), Time=1 | 223 | .040 | 0.040 | 0.005 | 0.067 |
| Agbagwara-Osuji 2015 | State anxiety: STAI, Time=PG3; Bonding: MIBS (Total), Time=2 | 223 | .290 | 0.299 | 0.005 | 0.067 |
| Agbagwara-Osuji 2015 | Trait anxiety: STAI, Time=PG3; Bonding: MIBS (Total), Time=2 | 223 | .300 | 0.310 | 0.005 | 0.067 |
| Agbagwara-Osuji 2015 | Depression: EPDS, Time=PG3; Bonding: MIBS (Total), Time=2 | 223 | .360 | 0.377 | 0.005 | 0.067 |
| Agbagwara-Osuji 2015 | Stress: SLES, Time=PG3; Bonding: MIBS (Total), Time=2 | 223 | .060 | 0.060 | 0.005 | 0.067 |
| Agbagwara-Osuji 2015 | State anxiety: STAI, Time=2; Bonding: MIBS (Total), Time=2 | 223 | .370 | 0.388 | 0.005 | 0.067 |
| Agbagwara-Osuji 2015 | Trait anxiety: STAI, Time=2; Bonding: MIBS (Total), Time=2 | 223 | .360 | 0.377 | 0.005 | 0.067 |
| Agbagwara-Osuji 2015 | Depression: EPDS, Time=2; Bonding: MIBS (Total), Time=2 | 223 | .400 | 0.424 | 0.005 | 0.067 |
| Agbagwara-Osuji 2015 | Stress: SLES, Time=2; Bonding: MIBS (Total), Time=2 | 223 | .050 | 0.050 | 0.005 | 0.067 |
| Agbagwara-Osuji 2015 | State anxiety: STAI, Time=2; Bonding: MIBS (Total), Time=1 | 223 | .340 | 0.354 | 0.005 | 0.067 |
| Agbagwara-Osuji 2015 | Trait anxiety: STAI, Time=2; Bonding: MIBS (Total), Time=1 | 223 | .330 | 0.343 | 0.005 | 0.067 |
| Agbagwara-Osuji 2015 | Depression: EPDS, Time=2; Bonding: MIBS (Total), Time=1 | 223 | .390 | 0.412 | 0.005 | 0.067 |
| Agbagwara-Osuji 2015 | Stress: SLES, Time=2; Bonding: MIBS (Total), Time=1 | 223 | .120 | 0.121 | 0.005 | 0.067 |
| Badr 2018 | Depression: Historical, Time=H; Bonding: PBQ (Total), Time=2 | 125 | .390 | 0.412 | 0.008 | 0.091 |
| Badr 2018 | Depression: EPDS, Time=1; Bonding: PBQ (Total), Time=2 | 125 | .250 | 0.255 | 0.008 | 0.091 |
| Behrendt 2019 | Depression: BDI-II, Time=4; Bonding: MPAS (Total), Time=4 | 61 | .261 | 0.267 | 0.017 | 0.131 |
| Bienfait 2011 | Depression: EPDS, Time=1; Bonding: MIBS (Total), Time=1 | 78 | .110 | 0.110 | 0.013 | 0.115 |
| Bonacquisti 2020 | Depression: EPDS, Time=6; Bonding: MAI (Total), Time=6 | 127 | .127 | 0.128 | 0.008 | 0.090 |
| Bonacquisti 2020 | Anxiety: DASS-42, Time=6; Bonding: MAI (Total), Time=6 | 127 | .199 | 0.202 | 0.008 | 0.090 |
| Bonacquisti 2020 | Stress: DASS-42, Time=6; Bonding: MAI (Total), Time=6 | 127 | .188 | 0.190 | 0.008 | 0.090 |
| Busonera 2017 | Depression: CES-D, Time=3; Bonding: PBQ (Total), Time=3 | 123 | .630 | 0.741 | 0.008 | 0.091 |
| Carter 2015 | Depression: EPDS, Time=6; Bonding: MPAS (Total), Time=6 | 43 | .483 | 0.527 | 0.025 | 0.158 |
| Chenery 2013 | Depression: EPDS, Time=2; Bonding: MPAS (Total), Time=2 | 64 | .538 | 0.601 | 0.016 | 0.128 |
| Chrzan-Dętkoś 2015 | Depression: EPDS, Time=2; Bonding: PBQ (Infant focused anxiety)*, Time=2 | 64 | .350 | 0.365 | 0.016 | 0.128 |
| Chrzan-Dętkoś 2015 | Depression: EPDS, Time=2; Bonding: PBQ (Impaired bonding), Time=2 | 64 | .370 | 0.388 | 0.016 | 0.128 |
| Condon 1998 | Depression: ZSDS, Time=3; Bonding: MPAS (Total), Time=3 | 210 | .340 | 0.354 | 0.005 | 0.070 |
| Condon 1998 | Depression: HADS, Time=3; Bonding: MPAS (Total), Time=3 | 210 | .460 | 0.497 | 0.005 | 0.070 |
| Condon 1998 | Anxiety: HADS, Time=3; Bonding: MPAS (Total), Time=3 | 210 | .340 | 0.354 | 0.005 | 0.070 |
| Cuijlits 2016 | Depression: EPDS, Time=4; Bonding: PPBS (Total), Time=4 | 1050 | .230 | 0.234 | 0.001 | 0.031 |
| Cuijlits 2016 | Depression: EPDS, Time=4; Bonding: PPBS (Total), Time=4 | 1050 | .250 | 0.255 | 0.001 | 0.031 |
| Daglar 2018 | Depression: CES-D, Time=PG3; Bonding: MIBS (Total), Time=2 | 227 | .174 | 0.176 | 0.004 | 0.067 |
| Daglar 2018 | Depression: CES-D, Time=2; Bonding: MIBS (Total), Time=2 | 227 | .221 | 0.225 | 0.004 | 0.067 |
| Daglar 2018 | Anxiety: BAI, Time=PG3; Bonding: MIBS (Total), Time=2 | 227 | .115 | 0.116 | 0.004 | 0.067 |
| Daglar 2018 | Anxiety: BAI, Time=2; Bonding: MIBS (Total), Time=2 | 227 | .151 | 0.152 | 0.004 | 0.067 |
| Dubber 2015 | Depression: EPDS, Time=PG3; Bonding: PBQ (Total), Time=2 | 54 | .262 | 0.268 | 0.020 | 0.140 |
| Dubber 2015 | Depression: EPDS, Time=2; Bonding: PBQ (Total), Time=2 | 77 | .417 | 0.444 | 0.014 | 0.116 |
| Dubber 2015 | State anxiety: STAI, Time=2; Bonding: PBQ (Total), Time=2 | 79 | .197 | 0.200 | 0.013 | 0.115 |
| Dubber 2015 | State anxiety: STAI, Time=PG3; Bonding: PBQ (Total), Time=2 | 54 | .199 | 0.202 | 0.020 | 0.140 |
| Dubber 2015 | Trait anxiety: STAI, Time=2; Bonding: PBQ (Total), Time=2 | 77 | .315 | 0.326 | 0.014 | 0.116 |
| Dubber 2015 | Trait anxiety: STAI, Time=PG3; Bonding: PBQ (Total), Time=2 | 55 | .247 | 0.252 | 0.019 | 0.139 |
| Edhborg 2011 | Depression: EPDS, Time=2; Bonding: PBQ (Total), Time=2 | 671 | .339 | 0.353 | 0.001 | 0.039 |
| Edhborg 2011 | State anxiety: STAI, Time=2; Bonding: PBQ (Total), Time=2 | 671 | .404 | 0.428 | 0.001 | 0.039 |
| Faisal-Cury 2020 | Mod-severe Depression: PHQ9, Time=4; Bonding: PBQ (Total), Time=4 | 266 | .430 | 0.460 | 0.004 | 0.062 |
| Faisal-Cury 2020 | Mild Depression: PHQ9, Time=4; Bonding: PBQ (Total), Time=4 | 281 | .176 | 0.178 | 0.004 | 0.060 |
| Fallon 2019 | Depression: BDI-II, Time=3; Bonding: PBQ (Total), Time=3 | 261 | .590 | 0.678 | 0.004 | 0.062 |
| Fallon 2019 | State anxiety: STAI, Time=3; Bonding: PBQ (Total), Time=3 | 261 | .560 | 0.633 | 0.004 | 0.062 |
| Fallon 2019 | Trait anxiety: STAI, Time=3; Bonding: PBQ (Total), Time=3 | 261 | .550 | 0.618 | 0.004 | 0.062 |
| Farré-Sender 2018 | Depression: EPDS, Time=PG; Bonding: PBQ (Total), Time=2 | 251 | .166 | 0.168 | 0.004 | 0.064 |
| Farré-Sender 2018 | State anxiety: STAI, Time=PG; Bonding: PBQ (Total), Time=2 | 249 | .189 | 0.191 | 0.004 | 0.064 |
| Figueiredo 2009 | Depression: EPDS, Time=1; Bonding: MIBS (Total), Time=1 | 315 | .280 | 0.288 | 0.003 | 0.057 |
| Fijalkowska 2019 | Depression: EPDS, Time=2; Bonding: MPAS (Total), Time=2 | 35 | .831 | 1.191 | 0.031 | 0.177 |
| Fransson 2020 | Depression: EPDS, Time=PG2; Bonding: PBQ (Total), Time=4 | 1090 | .210 | 0.213 | 0.001 | 0.030 |
| Fransson 2020 | Depression: EPDS, Time=PG3; Bonding: PBQ (Total), Time=4 | 1090 | .238 | 0.243 | 0.001 | 0.030 |
| Fransson 2020 | Depression: EPDS, Time=2; Bonding: PBQ (Total), Time=4 | 1090 | .285 | 0.293 | 0.001 | 0.030 |
| Fransson 2020 | Depression: EPDS, Time=4; Bonding: PBQ (Total), Time=4 | 1090 | .290 | 0.299 | 0.001 | 0.030 |
| Friedman 2008 | Depression: EPDS, Time=3; Bonding: PBQ (Total), Time=3 | 50 | .417 | 0.444 | 0.021 | 0.146 |
| Friedman 2008 | Anxiety: SCL-90, Time=3; Bonding: PBQ (Total), Time=3 | 50 | .330 | 0.343 | 0.021 | 0.146 |
| Garcia-Esteve 2016 | Depression: EPDS, Time=2; Bonding: PBQ (Infant focused anxiety)*, Time=2 | 840 | .510 | 0.563 | 0.001 | 0.035 |
| Garcia-Esteve 2016 | Depression: EPDS, Time=2; Bonding: PBQ (General factor)*, Time=2 | 840 | .590 | 0.678 | 0.001 | 0.035 |
| Garcia-Esteve 2016 | Depression: EPDS, Time=2; Bonding: PBQ (Impaired bonding)*, Time=2 | 840 | .530 | 0.590 | 0.001 | 0.035 |
| Garcia-Esteve 2016 | Depression: EPDS, Time=2; Bonding: PBQ (Lack of enjoyment)*, Time=2 | 840 | .400 | 0.424 | 0.001 | 0.035 |
| Garcia-Esteve 2016 | Depression: EPDS, Time=2; Bonding: PBQ (Rejection & risk of abuse)*, Time=2 | 840 | .320 | 0.332 | 0.001 | 0.035 |
| Gashe 2011 | Depression: EPDS, Time=4; Bonding: PBQ (Impaired bonding), Time=4 | 190 | .540 | 0.604 | 0.005 | 0.073 |
| Gashe 2011 | Depression: EPDS, Time=4; Bonding: PBQ (Infant focused anxiety)*, Time=4 | 190 | .470 | 0.510 | 0.005 | 0.073 |
| Gashe 2011 | Depression: EPDS, Time=4; Bonding: PBQ (Rejection & Anger), Time=4 | 190 | .450 | 0.485 | 0.005 | 0.073 |
| Hairston 2016 | Depression: EPDS, Time=3; Bonding: PBQ (Total), Time=3 | 152 | .406 | 0.431 | 0.007 | 0.082 |
| Hairston 2018 | Depression: EPDS, Time=2; Bonding: PBQ (Total), Time=2 | 114 | .446 | 0.480 | 0.009 | 0.095 |
| Handelzalts 2019 | Depression: EPDS, Time=3; Bonding: PBQ (Total), Time=3 | 504 | .480 | 0.523 | 0.002 | 0.045 |
| Herguner 2014 | Depression: EPDS, Time=3; Bonding: MAI (Total), Time=3 | 80 | .591 | 0.679 | 0.013 | 0.114 |
| Høivik 2013 | Depression: EPDS, Time=2; Bonding: PBQ (Total), Time=2 | 20 | .758 | 0.991 | 0.059 | 0.243 |
| Høivik 2013 | Depression: EPDS, Time=3; Bonding: PBQ (Total), Time=2 | 20 | .801 | 1.101 | 0.059 | 0.243 |
| Høivik 2013 | Depression: EPDS, Time=2; Bonding: MABISC (Total), Time=2 | 52 | .546 | 0.613 | 0.020 | 0.143 |
| Høivik 2013 | Depression: EPDS, Time=3; Bonding: MABISC (Total), Time=2 | 52 | .588 | 0.675 | 0.020 | 0.143 |
| Høivik 2013 | Depression: EPDS, Time=2; Bonding: MABISC (Total), Time=3 | 52 | .315 | 0.326 | 0.020 | 0.143 |
| Høivik 2013 | Depression: EPDS, Time=3; Bonding: MABISC (Total), Time=3 | 55 | .488 | 0.533 | 0.019 | 0.139 |
| Høivik 2013 | Depression: EPDS, Time=2; Bonding: PBQ (Total), Time=3 | 35 | .520 | 0.576 | 0.031 | 0.177 |
| Høivik 2013 | Depression: EPDS, Time=3; Bonding: PBQ (Total), Time=3 | 35 | .721 | 0.910 | 0.031 | 0.177 |
| Hrishikesh 2019 | Depression: EPDS, Time=2; Bonding: PBQ (Total), Time=2 | 100 | .550 | 0.618 | 0.010 | 0.102 |
| Jones 2011 | Depression: EPDS, Time=NR; Bonding: MPHI (Rel'ship w baby), Time=6 | 178 | .310 | 0.321 | 0.006 | 0.076 |
| Jordan 2014 | Depression: EPDS, Time=3; Bonding: MPAS (Total), Time=3 | 74 | .440 | 0.472 | 0.014 | 0.119 |
| Kaneko 2014 | Depression: EPDS, Time=3; Bonding: PBQ (Total), Time=3 | 1786 | .460 | 0.497 | 0.001 | 0.024 |
| Kaneko 2014 | Depression: EPDS, Time=3; Bonding: PBQ (Total), Time=3 | 1786 | .470 | 0.510 | 0.001 | 0.024 |
| Kasamatsu 2019 | Depression: EPDS, Time=2; Bonding: MIBS-J (Total), Time=4 | 83109 | .029 | 0.029 | 0.000 | 0.003 |
| Kasamatsu 2019 | Depression: EPDS, Time=4; Bonding: MIBS-J (Total), Time=4 | 83109 | .029 | 0.029 | 0.000 | 0.003 |
| Kerstis 2016 | Depression: EPDS, Time=2; Bonding: PBQ (Impaired bonding), Time=4 | 727 | .157 | 0.158 | 0.001 | 0.037 |
| Kerstis 2016 | Depression: EPDS, Time=4; Bonding: PBQ (Impaired bonding), Time=4 | 727 | .202 | 0.205 | 0.001 | 0.037 |
| Kita 2020 | Anxiety: HADS, Time=PG3; Bonding: MIBS-J (Anger & Rejection), Time=2 | 562 | .210 | 0.213 | 0.002 | 0.042 |
| Kita 2020 | Depression: HADS, Time=PG3; Bonding: MIBS-J (Anger & Rejection), Time=2 | 562 | .230 | 0.234 | 0.002 | 0.042 |
| Kita 2020 | Anxiety: HADS, Time=2; Bonding: MIBS-J (Anger & Rejection), Time=2 | 562 | .390 | 0.412 | 0.002 | 0.042 |
| Kita 2020 | Depression: HADS, Time=2; Bonding: MIBS-J (Anger & Rejection), Time=2 | 562 | .380 | 0.400 | 0.002 | 0.042 |
| Kita 2020 | Anxiety: HADS, Time=PG3; Bonding: MIBS-J (Lack of Affection), Time=2 | 562 | .150 | 0.151 | 0.002 | 0.042 |
| Kita 2020 | Depression: HADS, Time=PG3; Bonding: MIBS-J (Lack of Affection), Time=2 | 562 | .270 | 0.277 | 0.002 | 0.042 |
| Kita 2020 | Anxiety: HADS, Time=2; Bonding: MIBS-J (Lack of Affection), Time=2 | 562 | .320 | 0.332 | 0.002 | 0.042 |
| Kita 2020 | Depression: HADS, Time=2; Bonding: MIBS-J (Lack of Affection), Time=2 | 562 | .420 | 0.448 | 0.002 | 0.042 |
| Kleiber 2015 | Depression: EPDS, Time=3; Bonding: PBQ (Infant Focused Anxiety), Time=3 | 102 | .260 | 0.266 | 0.010 | 0.101 |
| Kleiber 2015 | Stress: PSS, Time=3; Bonding: PBQ (Infant Focused Anxiety), Time=3 | 102 | .260 | 0.266 | 0.010 | 0.101 |
| Kleiber 2015 | Depression: EPDS, Time=3; Bonding: PBQ (Impaired bonding), Time=3 | 102 | .430 | 0.460 | 0.010 | 0.101 |
| Kleiber 2015 | Stress: PSS, Time=3; Bonding: PBQ (Impaired bonding), Time=3 | 102 | .520 | 0.576 | 0.010 | 0.101 |
| Kleiber 2015 | Depression: EPDS, Time=3; Bonding: PBQ (Rejection & Anger), Time=3 | 102 | .210 | 0.213 | 0.010 | 0.101 |
| Kleiber 2015 | Stress: PSS, Time=3; Bonding: PBQ (Rejection & Anger), Time=3 | 102 | .210 | 0.213 | 0.010 | 0.101 |
| Kokubu 2012 | Anxiety: HADS, Time=PG3; Bonding: MIBS-J (Total), Time=1 | 99 | .180 | 0.182 | 0.010 | 0.102 |
| Kokubu 2012 | Depression: HADS, Time=PG3; Bonding: MIBS-J (Total), Time=1 | 99 | .320 | 0.332 | 0.010 | 0.102 |
| Kokubu 2012 | Blues: Maternity Blues Questionnaire, Time=1; Bonding: MIBS-J (Total), Time=1 | 99 | .310 | 0.321 | 0.010 | 0.102 |
| Kokubu 2012 | Depression: EPDS, Time=2; Bonding: MIBS-J (Total), Time=1 | 99 | .240 | 0.245 | 0.010 | 0.102 |
| Kokubu 2012 | Anxiety: HADS, Time=PG3; Bonding: MIBS-J (Total), Time=2 | 99 | .310 | 0.321 | 0.010 | 0.102 |
| Kokubu 2012 | Depression: HADS, Time=PG3; Bonding: MIBS-J (Total), Time=2 | 99 | .290 | 0.299 | 0.010 | 0.102 |
| Kokubu 2012 | Blues: Maternity Blues Questionnaire, Time=1; Bonding: MIBS-J (Total), Time=2 | 99 | .170 | 0.172 | 0.010 | 0.102 |
| Kokubu 2012 | Depression: EPDS, Time=2; Bonding: MIBS-J (Total), Time=2 | 99 | .460 | 0.497 | 0.010 | 0.102 |
| Lara-Cinisomo 2018 | Depression: EPDS, Time=PG3; Bonding: PBQ (Infant focused anxiety)*, Time=2 | 28 | .530 | 0.590 | 0.040 | 0.200 |
| Lara-Cinisomo 2018 | Depression: EPDS, Time=2; Bonding: PBQ (Infant Focused Anxiety), Time=2 | 28 | .500 | 0.549 | 0.040 | 0.200 |
| Lara-Cinisomo 2018 | Depression: EPDS, Time=2; Bonding: PBQ (Infant Focused Anxiety), Time=2 | 28 | .590 | 0.678 | 0.040 | 0.200 |
| Lara-Cinisomo 2018 | Depression: EPDS, Time=2; Bonding: PBQ (Impaired bonding), Time=2 | 28 | .420 | 0.448 | 0.040 | 0.200 |
| Lara-Cinisomo 2018 | Depression: EPDS, Time=2; Bonding: PBQ (Impaired bonding), Time=2 | 28 | .560 | 0.633 | 0.040 | 0.200 |
| Leahy-Warren 2020 | Depression: EPDS, Time=4; Bonding: MPAS (Total), Time=4 | 140 | .540 | 0.604 | 0.007 | 0.085 |
| Le Bas 2020 | Depression: EPDS, Time=PG1; Bonding: MPAS (Total), Time=2 | 1579 | .160 | 0.161 | 0.001 | 0.025 |
| Le Bas 2020 | Depression: EPDS, Time=PG2; Bonding: MPAS (Total), Time=2 | 1579 | .210 | 0.213 | 0.001 | 0.025 |
| Le Bas 2020 | Depression: EPDS, Time=PG3; Bonding: MPAS (Total), Time=2 | 1579 | .250 | 0.255 | 0.001 | 0.025 |
| Le Bas 2020 | Depression: EPDS, Time=2; Bonding: MPAS (Total), Time=2 | 1579 | .440 | 0.472 | 0.001 | 0.025 |
| Le Bas 2020 | Depression: EPDS, Time=4; Bonding: MPAS (Total), Time=2 | 1579 | .230 | 0.234 | 0.001 | 0.025 |
| Le Bas 2020 | Anxiety: DASS-21, Time=PG1; Bonding: MPAS (Total), Time=2 | 1579 | .080 | 0.080 | 0.001 | 0.025 |
| Le Bas 2020 | Anxiety: DASS-21, Time=PG2; Bonding: MPAS (Total), Time=2 | 1579 | .110 | 0.110 | 0.001 | 0.025 |
| Le Bas 2020 | Anxiety: DASS-21, Time=PG3; Bonding: MPAS (Total), Time=2 | 1579 | .120 | 0.121 | 0.001 | 0.025 |
| Le Bas 2020 | Anxiety: DASS-21, Time=2; Bonding: MPAS (Total), Time=2 | 1579 | .230 | 0.234 | 0.001 | 0.025 |
| Le Bas 2020 | Anxiety: DASS-21, Time=4; Bonding: MPAS (Total), Time=2 | 1579 | .110 | 0.110 | 0.001 | 0.025 |
| Le Bas 2020 | Stress: DASS-21, Time=PG1; Bonding: MPAS (Total), Time=2 | 1579 | .170 | 0.172 | 0.001 | 0.025 |
| Le Bas 2020 | Stress: DASS-21, Time=PG2; Bonding: MPAS (Total), Time=2 | 1579 | .220 | 0.224 | 0.001 | 0.025 |
| Le Bas 2020 | Stress: DASS-21, Time=PG3; Bonding: MPAS (Total), Time=2 | 1579 | .240 | 0.245 | 0.001 | 0.025 |
| Le Bas 2020 | Stress: DASS-21, Time=2; Bonding: MPAS (Total), Time=2 | 1579 | .480 | 0.523 | 0.001 | 0.025 |
| Le Bas 2020 | Stress: DASS-21, Time=4; Bonding: MPAS (Total), Time=2 | 1579 | .280 | 0.288 | 0.001 | 0.025 |
| Le Bas 2020 | Depression: EPDS, Time=PG1; Bonding: MPAS (Total), Time=4 | 1579 | .240 | 0.245 | 0.001 | 0.025 |
| Le Bas 2020 | Depression: EPDS, Time=PG2; Bonding: MPAS (Total), Time=4 | 1579 | .280 | 0.288 | 0.001 | 0.025 |
| Le Bas 2020 | Depression: EPDS, Time=PG3; Bonding: MPAS (Total), Time=4 | 1579 | .280 | 0.288 | 0.001 | 0.025 |
| Le Bas 2020 | Depression: EPDS, Time=2; Bonding: MPAS (Total), Time=4 | 1579 | .310 | 0.321 | 0.001 | 0.025 |
| Le Bas 2020 | Depression: EPDS, Time=4; Bonding: MPAS (Total), Time=4 | 1579 | .420 | 0.448 | 0.001 | 0.025 |
| Le Bas 2020 | Anxiety: DASS-21, Time=PG1; Bonding: MPAS (Total), Time=4 | 1579 | .110 | 0.110 | 0.001 | 0.025 |
| Le Bas 2020 | Anxiety: DASS-21, Time=PG2; Bonding: MPAS (Total), Time=4 | 1579 | .140 | 0.141 | 0.001 | 0.025 |
| Le Bas 2020 | Anxiety: DASS-21, Time=PG3; Bonding: MPAS (Total), Time=4 | 1579 | .130 | 0.131 | 0.001 | 0.025 |
| Le Bas 2020 | Anxiety: DASS-21, Time=2; Bonding: MPAS (Total), Time=4 | 1579 | .170 | 0.172 | 0.001 | 0.025 |
| Le Bas 2020 | Anxiety: DASS-21, Time=4; Bonding: MPAS (Total), Time=4 | 1579 | .260 | 0.266 | 0.001 | 0.025 |
| Le Bas 2020 | Stress: DASS-21, Time=PG1; Bonding: MPAS (Total), Time=4 | 1579 | .220 | 0.224 | 0.001 | 0.025 |
| Le Bas 2020 | Stress: DASS-21, Time=PG2; Bonding: MPAS (Total), Time=4 | 1579 | .270 | 0.277 | 0.001 | 0.025 |
| Le Bas 2020 | Stress: DASS-21, Time=PG3; Bonding: MPAS (Total), Time=4 | 1579 | .270 | 0.277 | 0.001 | 0.025 |
| Le Bas 2020 | Stress: DASS-21, Time=2; Bonding: MPAS (Total), Time=4 | 1579 | .350 | 0.365 | 0.001 | 0.025 |
| Le Bas 2020 | Stress: DASS-21, Time=4; Bonding: MPAS (Total), Time=4 | 1579 | .470 | 0.510 | 0.001 | 0.025 |
| Lehnig 2019 | Depression: BDI-II, Time=2; Bonding: PBQ (Total), Time=2 | 725 | .473 | 0.514 | 0.001 | 0.037 |
| Lehnig 2019 | Anxiety: SCL-90 R, Time=2; Bonding: PBQ (Total), Time=2 | 725 | .289 | 0.297 | 0.001 | 0.037 |
| Leserman 2011 | History of depression: MINI, Time=2; Bonding: PBQ (Total), Time=2 | 70 | .380 | 0.400 | 0.015 | 0.122 |
| Lutkiewicz 2020 | Depression: EPDS, Time=1; Bonding: PBQ (Infant Focused Anxiety), Time=1 | 150 | .305 | 0.315 | 0.007 | 0.082 |
| Lutkiewicz 2020 | Depression: PDSS, Time=1; Bonding: PBQ (Infant Focused Anxiety), Time=1 | 150 | .367 | 0.385 | 0.007 | 0.082 |
| Lutkiewicz 2020 | Anxiety: GAD-7, Time=1; Bonding: PBQ (Infant Focused Anxiety), Time=1 | 150 | .194 | 0.196 | 0.007 | 0.082 |
| Lutkiewicz 2020 | Depression: EPDS, Time=1; Bonding: PBQ (Risk of abuse), Time=1 | 150 | .417 | 0.444 | 0.007 | 0.082 |
| Lutkiewicz 2020 | Depression: PDSS, Time=1; Bonding: PBQ (Risk of abuse), Time=1 | 150 | .381 | 0.401 | 0.007 | 0.082 |
| Lutkiewicz 2020 | Anxiety: GAD-7, Time=1; Bonding: PBQ (Risk of abuse), Time=1 | 150 | .266 | 0.273 | 0.007 | 0.082 |
| Lutkiewicz 2020 | Depression: EPDS, Time=1; Bonding: PBQ (Impaired bonding), Time=1 | 150 | .456 | 0.492 | 0.007 | 0.082 |
| Lutkiewicz 2020 | Depression: PDSS, Time=1; Bonding: PBQ (Impaired bonding), Time=1 | 150 | .512 | 0.565 | 0.007 | 0.082 |
| Lutkiewicz 2020 | Anxiety: GAD-7, Time=1; Bonding: PBQ (Impaired bonding), Time=1 | 150 | .316 | 0.327 | 0.007 | 0.082 |
| Lutkiewicz 2020 | Depression: EPDS, Time=1; Bonding: PBQ (Rejection & Anger), Time=1 | 150 | .437 | 0.469 | 0.007 | 0.082 |
| Lutkiewicz 2020 | Depression: PDSS, Time=1; Bonding: PBQ (Rejection & Anger), Time=1 | 150 | .494 | 0.541 | 0.007 | 0.082 |
| Lutkiewicz 2020 | Anxiety: GAD-7, Time=1; Bonding: PBQ (Rejection & Anger), Time=1 | 150 | .345 | 0.360 | 0.007 | 0.082 |
| Luz 2017 | Anxiety: HADS, Time=PG3; Bonding: MPAS (Total), Time=2 | 40 | -.050 | -0.050 | 0.027 | 0.164 |
| Luz 2017 | Depression: HADS, Time=H; Bonding: MPAS (Total), Time=2 | 40 | .320 | 0.332 | 0.027 | 0.164 |
| Macdonald 2020 | Anxiety: RBPC, Time=Maternal age 13yrs; Bonding: MPAS (Total), Time=4 | 474 | .162 | 0.163 | 0.002 | 0.046 |
| Macdonald 2020 | Depression: SMFQ, Time=Maternal age 13yrs; Bonding: MPAS (Total), Time=4 | 476 | .147 | 0.148 | 0.002 | 0.046 |
| Macdonald 2020 | Depression: SMFQ, Time=Maternal age 15yrs; Bonding: MPAS (Total), Time=4 | 492 | .206 | 0.209 | 0.002 | 0.045 |
| Macdonald 2020 | Depression: DASS-21, Time=Maternal age 23yrs; Bonding: MPAS (Total), Time=4 | 492 | .170 | 0.171 | 0.002 | 0.045 |
| Macdonald 2020 | Anxiety: DASS-21, Time=Maternal age 23yrs; Bonding: MPAS (Total), Time=4 | 492 | .108 | 0.108 | 0.002 | 0.045 |
| Macdonald 2020 | Depression: DASS-21, Time=Maternal age 19yrs; Bonding: MPAS (Total), Time=4 | 493 | .083 | 0.083 | 0.002 | 0.045 |
| Macdonald 2020 | Anxiety: DASS-21, Time=Maternal age 19yrs; Bonding: MPAS (Total), Time=4 | 493 | .105 | 0.105 | 0.002 | 0.045 |
| Macdonald 2020 | Anxiety: RCMA-SF, Time=Maternal age 15yrs; Bonding: MPAS (Total), Time=4 | 494 | .181 | 0.182 | 0.002 | 0.045 |
| Macdonald 2020 | Depression: SMFQ, Time=Maternal age 17yrs; Bonding: MPAS (Total), Time=4 | 510 | .156 | 0.158 | 0.002 | 0.044 |
| Macdonald 2020 | Anxiety: RCMA-SF, Time=Maternal age 17yrs; Bonding: MPAS (Total), Time=4 | 510 | .177 | 0.179 | 0.002 | 0.044 |
| Macdonald 2020 | Depression: DASS-21, Time=Maternal age 27yrs; Bonding: MPAS (Total), Time=4 | 523 | .196 | 0.198 | 0.002 | 0.044 |
| Macdonald 2020 | Anxiety: DASS-21, Time=Maternal age 27yrs; Bonding: MPAS (Total), Time=4 | 523 | .163 | 0.164 | 0.002 | 0.044 |
| Macdonald 2020 | Depression: DASS-21, Time=4; Bonding: MPAS (Total), Time=4 | 582 | .389 | 0.410 | 0.002 | 0.042 |
| Macdonald 2020 | Anxiety: DASS-21, Time=4; Bonding: MPAS (Total), Time=4 | 582 | .273 | 0.280 | 0.002 | 0.042 |
| Mackie 2019 | Depression: EPDS, Time=3; Bonding: MPAS (Total), Time=3 | 5 | .400 | 0.424 | 0.500 | 0.707 |
| Martini 2020 | Mixed Anx/Dep: CIDI-V, Time=6; Bonding: PBQ (Total), Time=2 | 306 | .279 | 0.287 | 0.003 | 0.057 |
| Mason 2011 | Depression: EPDS, Time=2; Bonding: MPAS (Total), Time=2 | 276 | .380 | 0.400 | 0.004 | 0.061 |
| McErlean 2012 | Depression: EPDS, Time=3; Bonding: PBQ (Total), Time=3 | 77 | .431 | 0.461 | 0.014 | 0.116 |
| Milne 2007 | Depression: EPDS, Time=4; Bonding: MAS (Anxious to child), Time=4 | 139 | .531 | 0.592 | 0.007 | 0.086 |
| Milne 2007 | Depression: EPDS, Time=4; Bonding: MAS (Positive to child), Time=4 | 139 | .354 | 0.370 | 0.007 | 0.086 |
| Minamida 2020 | Depression: EPDS, Time=1; Bonding: PBQ (Total), Time=1 | 185 | .184 | 0.186 | 0.005 | 0.074 |
| Minamida 2020 | Depression: EPDS, Time=1; Bonding: PBQ (Total), Time=1 | 185 | .092 | 0.093 | 0.005 | 0.074 |
| Minamida 2020 | Depression: EPDS, Time=2; Bonding: PBQ (Total), Time=2 | 185 | .449 | 0.483 | 0.005 | 0.074 |
| Moehler 2006 | Depression: EPDS, Time=3; Bonding: PBQ (Total), Time=2 | 101 | .280 | 0.288 | 0.010 | 0.101 |
| Moehler 2006 | Depression: EPDS, Time=3; Bonding: PBQ (Total), Time=2 | 101 | .390 | 0.412 | 0.010 | 0.101 |
| Moehler 2006 | Depression: EPDS, Time=3; Bonding: PBQ (Total), Time=3 | 101 | .350 | 0.365 | 0.010 | 0.101 |
| Morrison 2016 | Depression: EPDS, Time=PG3; Bonding: MPAS (Total), Time=2 | 70 | .243 | 0.248 | 0.015 | 0.122 |
| Morrison 2016 | Depression: EPDS, Time=2; Bonding: MPAS (Total), Time=2 | 70 | .109 | 0.109 | 0.015 | 0.122 |
| Moser 1989 | Depression: BDI, Time=3; Bonding: PPAM (Total), Time=4 | 37 | .330 | 0.343 | 0.029 | 0.171 |
| Moser 1989 | Stress: Combined measures, Time=4; Bonding: PPAM (Total), Time=4 | 37 | .320 | 0.332 | 0.029 | 0.171 |
| Muzik 2017 | Depression: PPDS, Time=4; Bonding: PBQ (Total), Time=4 | 122 | .320 | 0.332 | 0.008 | 0.092 |
| Myers 2017 | Depression: EPDS & BPDS, Time=4; Bonding: MIBS (Total), Time=2 | 48 | .107 | 0.107 | 0.022 | 0.149 |
| Nagata 2000 | Depression: ZSDS , Time=1; Bonding: MAS (Anxiety re Child), Time=1 | 417 | .390 | 0.412 | 0.002 | 0.049 |
| Nagata 2000 | Depression: ZSDS , Time=1; Bonding: MAS (Core Maternal Attachment), Time=1 | 417 | .300 | 0.310 | 0.002 | 0.049 |
| Nagata 2004 | Depression: ZSDS , Time=1; Bonding: MAS (Anxiety re Child), Time=1 | 153 | .330 | 0.343 | 0.007 | 0.082 |
| Nagata 2004 | Depression: ZSDS , Time=1; Bonding: MAS (Core Maternal Attachment), Time=1 | 153 | .270 | 0.277 | 0.007 | 0.082 |
| Nakano 2019 | Depression: EPDS, Time=2; Bonding: PBQ (Total), Time=2 | 1060 | .107 | 0.107 | 0.001 | 0.031 |
| Nakano 2019 | Depression: EPDS, Time=2; Bonding: PBQ (Total), Time=2 | 1060 | .593 | 0.682 | 0.001 | 0.031 |
| Nath 2019 | Depression: EPDS, Time=PG2; Bonding: PBQ (Total), Time=3 | 400 | .213 | 0.216 | 0.003 | 0.050 |
| Nath 2019 | Depression: EPDS, Time=PG3; Bonding: PBQ (Total), Time=3 | 380 | .123 | 0.124 | 0.003 | 0.052 |
| Nath 2019 | Anxiety: SCID, Time=PG2; Bonding: PBQ (Total), Time=3 | 404 | .127 | 0.127 | 0.002 | 0.050 |
| Nath 2019 | Depression: SCID, Time=PG2; Bonding: PBQ (Total), Time=3 | 404 | .202 | 0.205 | 0.002 | 0.050 |
| Nath 2019 | Depression: EPDS, Time=PG; Bonding: PBQ (Total), Time=3 | 404 | .212 | 0.215 | 0.002 | 0.050 |
| Ngoma 2012 | Depression: EPDS, Time=2; Bonding: MIBS-J (Total), Time=2 | 42 | .470 | 0.510 | 0.026 | 0.160 |
| Ngoma 2012 | Depression: EPDS, Time=2; Bonding: MIBS-J (Total), Time=2 | 76 | .220 | 0.224 | 0.014 | 0.117 |
| Nolvi 2016 | Depression: EPDS, Time=3; Bonding: PBQ (Total), Time=4 | 102 | .253 | 0.259 | 0.010 | 0.101 |
| Nolvi 2016 | State anxiety: STAI, Time=3; Bonding: PBQ (Total), Time=4 | 102 | .260 | 0.266 | 0.010 | 0.101 |
| Nonnenmacher 2016 | Depression: SCID-I, Time=3; Bonding: PBQ (Total), Time=3 | 93 | .610 | 0.709 | 0.011 | 0.105 |
| Noyman-Veksler 2015 | Depression: EPDS, Time=2; Bonding: PBQ (Total), Time=2 | 92 | .300 | 0.310 | 0.011 | 0.106 |
| Noyman-Veksler 2015 | Depression: EPDS, Time=2; Bonding: PBQ (Total), Time=2 | 92 | .380 | 0.400 | 0.011 | 0.106 |
| Noyman-Veksler 2015 | Depression: EPDS, Time=2; Bonding: PBQ (Total), Time=2 | 92 | .300 | 0.310 | 0.011 | 0.106 |
| Noyman-Veksler 2015 | Depression: EPDS, Time=2; Bonding: PBQ (Total), Time=2 | 96 | .620 | 0.725 | 0.011 | 0.104 |
| O'Higgins 2013 | Depression: EPDS, Time=2; Bonding: MIBS (Total), Time=2 | 79 | .440 | 0.472 | 0.013 | 0.115 |
| O'Higgins 2013 | Depression: EPDS, Time=2; Bonding: MIBS (Total), Time=2 | 79 | .340 | 0.354 | 0.013 | 0.115 |
| O'Higgins 2013 | Depression: EPDS, Time=2; Bonding: MIBS (Total), Time=3 | 79 | .260 | 0.266 | 0.013 | 0.115 |
| O'Higgins 2013 | Depression: EPDS, Time=2; Bonding: MIBS (Total), Time=4 | 79 | .260 | 0.266 | 0.013 | 0.115 |
| Oddo-Sommerfeld 2016 | Depression: BDI-V, Time=PG3; Bonding: PBQ (Impaired bonding), Time=2 | 266 | .190 | 0.192 | 0.004 | 0.062 |
| Oddo-Sommerfeld 2016 | State anxiety: STADI, Time=PG3; Bonding: PBQ (Impaired bonding), Time=2 | 266 | .190 | 0.192 | 0.004 | 0.062 |
| Oddo-Sommerfeld 2016 | State anxiety: STADI, Time=2; Bonding: PBQ (Impaired bonding), Time=2 | 266 | .360 | 0.377 | 0.004 | 0.062 |
| Oddo-Sommerfeld 2016 | Depression: EPDS, Time=2; Bonding: PBQ (Impaired bonding), Time=2 | 266 | .440 | 0.472 | 0.004 | 0.062 |
| Ohara 2016 | Depression: EPDS, Time=1; Bonding: MIBQ (Anger & Rejection), Time=1 | 751 | .250 | 0.255 | 0.001 | 0.037 |
| Ohara 2016 | Depression: EPDS, Time=1; Bonding: MIBQ (Lack of Affection), Time=1 | 751 | .170 | 0.172 | 0.001 | 0.037 |
| Ohara 2017 (b) | Anxiety: EPDS, Time=PG; Bonding: MIBQ (Lack of Affection), Time=1 | 751 | .060 | 0.060 | 0.001 | 0.037 |
| Ohara 2017 (b) | Depression: EPDS, Time=PG; Bonding: MIBQ (Lack of Affection), Time=1 | 751 | .040 | 0.040 | 0.001 | 0.037 |
| Ohara 2017 (b) | Anhedonia: EPDS, Time=PG; Bonding: MIBQ (Lack of Affection), Time=1 | 751 | .100 | 0.100 | 0.001 | 0.037 |
| Ohara 2017 (b) | Anxiety: EPDS, Time=PG3; Bonding: MIBQ (Lack of Affection), Time=1 | 751 | .080 | 0.080 | 0.001 | 0.037 |
| Ohara 2017 (b) | Depression: EPDS, Time=PG3; Bonding: MIBQ (Lack of Affection), Time=1 | 751 | .050 | 0.050 | 0.001 | 0.037 |
| Ohara 2017 (b) | Anhedonia: EPDS, Time=PG3; Bonding: MIBQ (Lack of Affection), Time=1 | 751 | .080 | 0.080 | 0.001 | 0.037 |
| Ohara 2017 (b) | Anxiety: EPDS, Time=PG; Bonding: MIBQ (Anger & Rejection), Time=1 | 751 | .110 | 0.110 | 0.001 | 0.037 |
| Ohara 2017 (b) | Depression: EPDS, Time=PG; Bonding: MIBQ (Anger & Rejection), Time=1 | 751 | .110 | 0.110 | 0.001 | 0.037 |
| Ohara 2017 (b) | Anhedonia: EPDS, Time=PG; Bonding: MIBQ (Anger & Rejection), Time=1 | 751 | .090 | 0.090 | 0.001 | 0.037 |
| Ohara 2017 (b) | Anxiety: EPDS, Time=PG3; Bonding: MIBQ (Anger & Rejection), Time=1 | 751 | .160 | 0.161 | 0.001 | 0.037 |
| Ohara 2017 (b) | Depression: EPDS, Time=PG3; Bonding: MIBQ (Anger & Rejection), Time=1 | 751 | .110 | 0.110 | 0.001 | 0.037 |
| Ohara 2017 (b) | Anhedonia: EPDS, Time=PG3; Bonding: MIBQ (Anger & Rejection), Time=1 | 751 | .050 | 0.050 | 0.001 | 0.037 |
| Ohara 2018 | Anxiety: EPDS, Time=PG2; Bonding: MIBQ (Anger & Rejection), Time=2 | 855 | .140 | 0.141 | 0.001 | 0.034 |
| Ohara 2018 | Depression: EPDS, Time=PG2; Bonding: MIBQ (Anger & Rejection), Time=2 | 855 | .130 | 0.131 | 0.001 | 0.034 |
| Ohara 2018 | Anhedonia: EPDS, Time=PG2; Bonding: MIBQ (Anger & Rejection), Time=2 | 855 | .110 | 0.110 | 0.001 | 0.034 |
| Ohara 2018 | Anxiety: EPDS, Time=2; Bonding: MIBQ (Anger & Rejection), Time=2 | 855 | .290 | 0.299 | 0.001 | 0.034 |
| Ohara 2018 | Depression: EPDS, Time=2; Bonding: MIBQ (Anger & Rejection), Time=2 | 855 | .310 | 0.321 | 0.001 | 0.034 |
| Ohara 2018 | Anhedonia: EPDS, Time=2; Bonding: MIBQ (Anger & Rejection), Time=2 | 855 | .210 | 0.213 | 0.001 | 0.034 |
| Ohara 2018 | Anxiety: EPDS, Time=PG2; Bonding: MIBQ (Lack of Affection), Time=2 | 855 | .020 | 0.020 | 0.001 | 0.034 |
| Ohara 2018 | Depression: EPDS, Time=PG2; Bonding: MIBQ (Lack of Affection), Time=2 | 855 | .050 | 0.050 | 0.001 | 0.034 |
| Ohara 2018 | Anhedonia: EPDS, Time=PG2; Bonding: MIBQ (Lack of Affection), Time=2 | 855 | .040 | 0.040 | 0.001 | 0.034 |
| Ohara 2018 | Anxiety: EPDS, Time=2; Bonding: MIBQ (Lack of Affection), Time=2 | 855 | .130 | 0.131 | 0.001 | 0.034 |
| Ohara 2018 | Depression: EPDS, Time=2; Bonding: MIBQ (Lack of Affection), Time=2 | 855 | .270 | 0.277 | 0.001 | 0.034 |
| Ohara 2018 | Anhedonia: EPDS, Time=2; Bonding: MIBQ (Lack of Affection), Time=2 | 855 | .240 | 0.245 | 0.001 | 0.034 |
| Ohashi 2016 | Depression: EPDS, Time=2; Bonding: PBQ (Anger & Restrictedness)*, Time=2 | 364 | .490 | 0.536 | 0.003 | 0.053 |
| Ohashi 2016 | Depression: EPDS, Time=2; Bonding: PBQ (Lack of Affection)*, Time=2 | 364 | .210 | 0.213 | 0.003 | 0.053 |
| Ohashi 2016 | Depression: EPDS, Time=2; Bonding: PBQ (Rejection & Fear)*, Time=2 | 364 | .320 | 0.332 | 0.003 | 0.053 |
| Olsson 2020 | Mixed Anx/Dep: CIS/GHQ-12, Time=Maternal age 14-17yrs; Bonding: PBQ (Total), Time=2 | 469 | .044 | 0.044 | 0.002 | 0.046 |
| Olsson 2020 | Mixed Anx/Dep: CIS/GHQ-12, Time=Maternal age 14-17yrs; Bonding: PBQ (Total), Time=2 | 473 | .034 | 0.034 | 0.002 | 0.046 |
| Olsson 2020 | Mixed Anx/Dep: CIS/GHQ-12, Time=Maternal age 14-17yrs; Bonding: PBQ (Total), Time=2 | 474 | .112 | 0.113 | 0.002 | 0.046 |
| Olsson 2020 | Mixed Anx/Dep: CIS/GHQ-12, Time=Maternal age 14-17yrs; Bonding: PBQ (Total), Time=2 | 477 | .153 | 0.155 | 0.002 | 0.046 |
| Olsson 2020 | Mixed Anx/Dep: CIS/GHQ-12, Time=Maternal age 14-17yrs; Bonding: PBQ (Total), Time=2 | 478 | .143 | 0.144 | 0.002 | 0.046 |
| Olsson 2020 | Mixed Anx/Dep: CIS/GHQ-12, Time=Maternal adolescence(persistent); Bonding: PBQ (Total), Time=2 | 488 | .084 | 0.084 | 0.002 | 0.045 |
| Olsson 2020 | Mixed Anx/Dep: GHQ-12, Time=Maternal age 24yrs; Bonding: PBQ (Total), Time=2 | 488 | .163 | 0.165 | 0.002 | 0.045 |
| Olsson 2020 | Mixed Anx/Dep: CIS/GHQ-12, Time=Maternal age 20yrs; Bonding: PBQ (Total), Time=2 | 490 | .107 | 0.108 | 0.002 | 0.045 |
| Olsson 2020 | Mixed Anx/Dep: GHQ-12, Time=Maternal age 29yrs; Bonding: PBQ (Total), Time=2 | 499 | .130 | 0.130 | 0.002 | 0.045 |
| Olsson 2020 | Mixed Anx/Dep: CIS/GHQ-12, Time=Maternal young adulthood(persistent); Bonding: PBQ (Total), Time=2 | 506 | .179 | 0.181 | 0.002 | 0.045 |
| Olsson 2020 | Mixed Anx/Dep: CIS/GHQ-12, Time=Maternal age 14-17yrs; Bonding: PBQ (Total), Time=4 | 518 | .098 | 0.098 | 0.002 | 0.044 |
| Olsson 2020 | Mixed Anx/Dep: CIS/GHQ-12, Time=Maternal age 14-17yrs; Bonding: PBQ (Total), Time=4 | 523 | .135 | 0.135 | 0.002 | 0.044 |
| Olsson 2020 | Mixed Anx/Dep: CIS/GHQ-12, Time=Maternal age 14-17yrs; Bonding: PBQ (Total), Time=4 | 524 | .118 | 0.118 | 0.002 | 0.044 |
| Olsson 2020 | Mixed Anx/Dep: CIS/GHQ-12, Time=Maternal age 14-17yrs; Bonding: PBQ (Total), Time=4 | 524 | .083 | 0.083 | 0.002 | 0.044 |
| Olsson 2020 | Mixed Anx/Dep: CIS/GHQ-12, Time=Maternal age 14-17yrs; Bonding: PBQ (Total), Time=4 | 528 | .191 | 0.194 | 0.002 | 0.044 |
| Olsson 2020 | Mixed Anx/Dep: GHQ-12, Time=Maternal age 24yrs; Bonding: PBQ (Total), Time=4 | 536 | .202 | 0.205 | 0.002 | 0.043 |
| Olsson 2020 | Mixed Anx/Dep: CIS/GHQ-12, Time=Maternal adolescence(persistent); Bonding: PBQ (Total), Time=4 | 540 | .122 | 0.123 | 0.002 | 0.043 |
| Olsson 2020 | Mixed Anx/Dep: CIS/GHQ-12, Time=Maternal age 20yrs; Bonding: PBQ (Total), Time=4 | 540 | .146 | 0.147 | 0.002 | 0.043 |
| Olsson 2020 | Mixed Anx/Dep: GHQ-12, Time=Maternal age 29yrs; Bonding: PBQ (Total), Time=4 | 553 | .125 | 0.126 | 0.002 | 0.043 |
| Olsson 2020 | Mixed Anx/Dep: CIS/GHQ-12, Time=Maternal age 20yrs; Bonding: PBQ (Total), Time=4 | 560 | .199 | 0.202 | 0.002 | 0.042 |
| Örün 2013 | Depression: BSI, Time=1; Bonding: PBQ (Total), Time=2 | 189 | .225 | 0.229 | 0.005 | 0.073 |
| Örün 2013 | Depression: BSI, Time=1; Bonding: MIBS (Total), Time=2 | 189 | .150 | 0.151 | 0.005 | 0.073 |
| Örün 2013 | Anxiety: BSI, Time=1; Bonding: MIBS (Total), Time=2 | 189 | .185 | 0.187 | 0.005 | 0.073 |
| Örün 2013 | Depression: EPDS, Time=2; Bonding: MIBS (Total), Time=2 | 189 | .377 | 0.397 | 0.005 | 0.073 |
| Örün 2013 | Depression: EPDS, Time=2; Bonding: PBQ (Total), Time=2 | 189 | .449 | 0.483 | 0.005 | 0.073 |
| Oskovi-Kaplan 2020 | Depression: EPDS, Time=1; Bonding: MAI (Total), Time=1 | 223 | .418 | 0.445 | 0.005 | 0.067 |
| Parfitt 2014 | Mixed Anx/Dep: HADS, Time=PG3; Bonding: PBQ (Total), Time=2 | 48 | .490 | 0.536 | 0.022 | 0.149 |
| Parfitt 2014 | Mixed Anx/Dep: HADS, Time=3; Bonding: PBQ (Total), Time=2 | 48 | .370 | 0.388 | 0.022 | 0.149 |
| Pearson 2013 | Depression: EPDS, Time=NR; Bonding: PBQ (Total), Time=3 | 49 | .200 | 0.203 | 0.022 | 0.147 |
| Petri 2017 | History of mood disorder: SCID-I, Time=PG1; Bonding: MPAS (Total), Time=2 | 106 | .314 | 0.325 | 0.010 | 0.099 |
| Rados 2020 | Depression: EPDS, Time=6; Bonding: PBQ (Total), Time=6 | 603 | .550 | 0.618 | 0.002 | 0.041 |
| Rados 2020 | Depression: EPDS, Time=6; Bonding: PBQ (Total), Time=6 | 603 | .720 | 0.908 | 0.002 | 0.041 |
| Reck 2006 | Depression: SCID-I, Time=2; Bonding: PBQ (Total), Time=2 | 862 | .190 | 0.192 | 0.001 | 0.034 |
| Reck 2015 | Depression: SCID-I, Time=3; Bonding: PBQ (Total), Time=3 | 63 | .450 | 0.485 | 0.017 | 0.129 |
| Riera-Martin 2018 | Depression: EPDS, Time=4; Bonding: MPAS (Total), Time=4 | 571 | .410 | 0.436 | 0.002 | 0.042 |
| Robakis 2015 | Depression: EPDS, Time=3; Bonding: MIBS (Total), Time=2 | 90 | .272 | 0.279 | 0.011 | 0.107 |
| Robakis 2015 | Depression: EPDS, Time=2; Bonding: MIBS (Total), Time=3 | 91 | .337 | 0.351 | 0.011 | 0.107 |
| Robakis 2015 | Depression: EPDS, Time=3; Bonding: MIBS (Total), Time=2 | 95 | .375 | 0.394 | 0.011 | 0.104 |
| Robakis 2015 | Depression: EPDS, Time=PG3; Bonding: MIBS (Total), Time=3 | 97 | .341 | 0.355 | 0.011 | 0.103 |
| Robakis 2015 | Depression: EPDS, Time=2; Bonding: MIBS (Total), Time=3 | 97 | .344 | 0.359 | 0.011 | 0.103 |
| Robakis 2015 | Depression: EPDS, Time=3; Bonding: MIBS (Total), Time=3 | 97 | .384 | 0.405 | 0.011 | 0.103 |
| Robakis 2015 | Depression: EPDS, Time=2; Bonding: MIBS (Total), Time=2 | 105 | .302 | 0.312 | 0.010 | 0.099 |
| Robakis 2015 | Depression: EPDS, Time=2; Bonding: MIBS (Total), Time=2 | 106 | .383 | 0.404 | 0.010 | 0.099 |
| Robakis 2015 | Depression: EPDS, Time=PG3; Bonding: MIBS (Total), Time=2 | 107 | .400 | 0.424 | 0.010 | 0.098 |
| Robakis 2015 | Depression: EPDS, Time=2; Bonding: MIBS (Total), Time=2 | 107 | .350 | 0.365 | 0.010 | 0.098 |
| Robakis 2015 | Depression: EPDS, Time=PG3; Bonding: MIBS (Total), Time=2 | 118 | .309 | 0.319 | 0.009 | 0.093 |
| Robakis 2015 | Depression: EPDS, Time=2; Bonding: MIBS (Total), Time=2 | 118 | .476 | 0.518 | 0.009 | 0.093 |
| Rossen 2019 | Depression: DASS-21, Time=2; Bonding: MPAS (Total), Time=2 | 308 | .370 | 0.388 | 0.003 | 0.057 |
| Rossen 2019 | Depression: DASS-21, Time=2; Bonding: MPAS (Total), Time=4 | 308 | .320 | 0.332 | 0.003 | 0.057 |
| Sawyer Cohen 2010 | Trait anxiety: STAI, Time=6; Bonding: MAI (Total), Time=6 | 168 | .366 | 0.384 | 0.006 | 0.078 |
| Sawyer Cohen 2010 | Depression: EPDS, Time=6; Bonding: MAI (Total), Time=6 | 180 | .315 | 0.326 | 0.006 | 0.075 |
| Sawyer Cohen 2010 | Trait anxiety: STAI, Time=PG; Bonding: MAI (Total), Time=6 | 185 | .168 | 0.170 | 0.005 | 0.074 |
| Sawyer Cohen 2010 | Depression: EPDS, Time=PG; Bonding: MAI (Total), Time=6 | 186 | .108 | 0.108 | 0.005 | 0.074 |
| Scopesi 2004 | Depression: SRT, Time=2; Bonding: MPAS (Total), Time=2 | 208 | .323 | 0.335 | 0.005 | 0.070 |
| Scopesi 2004 | Anxiety: SRT, Time=2; Bonding: MPAS (Total), Time=2 | 208 | .313 | 0.324 | 0.005 | 0.070 |
| Seng 2013 | Depression: CIDI-D, Time=H; Bonding: PBQ (Total), Time=2 | 566 | .121 | 0.122 | 0.002 | 0.042 |
| Sockol 2014 | Depression: EPDS, Time=2; Bonding: PBQ (Impaired bonding), Time=2 | 180 | .200 | 0.203 | 0.006 | 0.075 |
| Sockol 2014 | Depression: EPDS, Time=2; Bonding: PBQ (Infant Focused Anxiety), Time=2 | 180 | .200 | 0.203 | 0.006 | 0.075 |
| Sockol 2014 | Depression: EPDS, Time=2; Bonding: PBQ (Rejection & Anger), Time=2 | 180 | .200 | 0.203 | 0.006 | 0.075 |
| Sockol 2014 | Depression: EPDS, Time=2; Bonding: PBQ (Risk of abuse), Time=2 | 180 | .200 | 0.203 | 0.006 | 0.075 |
| Suetsugu 2015 | Depression: EPDS, Time=2; Bonding: PBQ (Total), Time=2 | 244 | .559 | 0.631 | 0.004 | 0.064 |
| Sun-Hee 2019 | Depression: Likert questions, Time=2; Bonding: MAI (Total), Time=2 | 217 | .536 | 0.599 | 0.005 | 0.068 |
| Talmon 2019 | Depression: EPDS, Time=PG; Bonding: PBQ (Impaired bonding), Time=2 | 394 | .140 | 0.141 | 0.003 | 0.051 |
| Talmon 2019 | Depression: EPDS, Time=2; Bonding: PBQ (Impaired bonding), Time=2 | 394 | .470 | 0.510 | 0.003 | 0.051 |
| Talmon 2019 | Depression: EPDS, Time=PG; Bonding: PBQ (Infant Focused Anxiety), Time=2 | 394 | .220 | 0.224 | 0.003 | 0.051 |
| Talmon 2019 | Depression: EPDS, Time=2; Bonding: PBQ (Infant Focused Anxiety), Time=2 | 394 | .550 | 0.618 | 0.003 | 0.051 |
| Talmon 2019 | Depression: EPDS, Time=PG; Bonding: PBQ (Rejection & Anger), Time=2 | 394 | .090 | 0.090 | 0.003 | 0.051 |
| Talmon 2019 | Depression: EPDS, Time=2; Bonding: PBQ (Rejection & Anger), Time=2 | 394 | .440 | 0.472 | 0.003 | 0.051 |
| Taylor 2005 | Depression: EPDS, Time=1; Bonding: MIBS (Total), Time=1 | 144 | .155 | 0.156 | 0.007 | 0.084 |
| Taylor 2005 | Depression: EPDS, Time=2; Bonding: MIBS (Total), Time=1 | 144 | .057 | 0.057 | 0.007 | 0.084 |
| Taylor 2005 | Blues: Kennerley Blues Scale, Time=1; Bonding: MIBS (Total), Time=1 | 144 | .114 | 0.114 | 0.007 | 0.084 |
| Taylor 2005 | Depression: EPDS, Time=1; Bonding: MIBS (Total), Time=2 | 144 | .244 | 0.249 | 0.007 | 0.084 |
| Taylor 2005 | Depression: EPDS, Time=2; Bonding: MIBS (Total), Time=2 | 144 | .310 | 0.321 | 0.007 | 0.084 |
| Taylor 2005 | Blues: Kennerley Blues Scale, Time=1; Bonding: MIBS (Total), Time=2 | 144 | .166 | 0.168 | 0.007 | 0.084 |
| Taylor 2005 | Depression: EPDS, Time=1; Bonding: MIBS (Total), Time=2 | 144 | .078 | 0.078 | 0.007 | 0.084 |
| Taylor 2005 | Depression: EPDS, Time=2; Bonding: MIBS (Total), Time=2 | 144 | .181 | 0.183 | 0.007 | 0.084 |
| Taylor 2005 | Blues: Kennerley Blues Scale, Time=1; Bonding: MIBS (Total), Time=2 | 144 | .025 | 0.025 | 0.007 | 0.084 |
| Tester-Jones 2015 | Depression: EPDS, Time=4; Bonding: PBQ (Total), Time=4 | 203 | .530 | 0.590 | 0.005 | 0.071 |
| Tietz 2014 | Depression: EPDS, Time=3; Bonding: PBQ (Total), Time=3 | 78 | .492 | 0.539 | 0.013 | 0.115 |
| Tietz 2014 | Anxiety: SCID-I, Time=3; Bonding: PBQ (Total), Time=3 | 78 | .380 | 0.400 | 0.013 | 0.115 |
| Tikotzky 2016 | Depression: EPDS, Time=4; Bonding: PBQ (Total), Time=4 | 80 | .590 | 0.678 | 0.013 | 0.114 |
| Tikotzky 2016 | Depression: EPDS, Time=4; Bonding: MPAS (Total), Time=4 | 80 | .390 | 0.412 | 0.013 | 0.114 |
| Tolja 2020 | Depression: EPDS, Time=4; Bonding: PBQ (Total), Time=4 | 241 | .520 | 0.576 | 0.004 | 0.065 |
| Tolja 2020 | Anxiety: DASS-21, Time=4; Bonding: PBQ (Total), Time=4 | 241 | .360 | 0.377 | 0.004 | 0.065 |
| Tolja 2020 | Depression: Historical, Time=4; Bonding: PBQ (Total), Time=4 | 241 | .420 | 0.448 | 0.004 | 0.065 |
| VanBussel 2010 | Depression: EPDS, Time=3; Bonding: MPAS (Total), Time=3 | 202 | .320 | 0.332 | 0.005 | 0.071 |
| VanBussel 2010 | Anxiety: HADS, Time=3; Bonding: MPAS (Total), Time=3 | 202 | .340 | 0.354 | 0.005 | 0.071 |
| VanBussel 2010 | Depression: EPDS, Time=3; Bonding: PBQ (Total), Time=3 | 202 | .390 | 0.412 | 0.005 | 0.071 |
| VanBussel 2010 | Anxiety: HADS, Time=3; Bonding: PBQ (Total), Time=3 | 202 | .370 | 0.388 | 0.005 | 0.071 |
| VanBussel 2010 | Depression: EPDS, Time=3; Bonding: MIBS (Total), Time=3 | 202 | .290 | 0.299 | 0.005 | 0.071 |
| VanBussel 2010 | Anxiety: HADS, Time=3; Bonding: MIBS (Total), Time=3 | 202 | .160 | 0.161 | 0.005 | 0.071 |
| VanBussel 2010 | Depression: EPDS, Time=2; Bonding: MPAS (Total), Time=2 | 263 | .300 | 0.310 | 0.004 | 0.062 |
| VanBussel 2010 | Anxiety: HADS, Time=2; Bonding: MPAS (Total), Time=2 | 263 | .400 | 0.424 | 0.004 | 0.062 |
| VanBussel 2010 | Depression: EPDS, Time=2; Bonding: PBQ (Total), Time=2 | 263 | .410 | 0.436 | 0.004 | 0.062 |
| VanBussel 2010 | Anxiety: HADS, Time=2; Bonding: PBQ (Total), Time=2 | 263 | .440 | 0.472 | 0.004 | 0.062 |
| VanBussel 2010 | Depression: EPDS, Time=2; Bonding: MIBS (Total), Time=2 | 263 | .370 | 0.388 | 0.004 | 0.062 |
| VanBussel 2010 | Anxiety: HADS, Time=2; Bonding: MIBS (Total), Time=2 | 263 | .350 | 0.365 | 0.004 | 0.062 |
| Vengadavaradan 2019 | Depression: EPDS, Time=6; Bonding: PBQ (Total), Time=6 | 250 | .528 | 0.587 | 0.004 | 0.064 |
| Wikman 2020 | Depression: EPDS, Time=PG; Bonding: PBQ (Total), Time=2 | 209 | .092 | 0.092 | 0.005 | 0.070 |
| Wikman 2020 | Depression: EPDS, Time=2; Bonding: PBQ (Total), Time=2 | 270 | .631 | 0.743 | 0.004 | 0.061 |
| Wikman 2020 | Depression: EPDS, Time=4; Bonding: PBQ (Total), Time=2 | 132 | .666 | 0.804 | 0.008 | 0.088 |
| Wikman 2020 | Depression: EPDS, Time=6; Bonding: PBQ (Total), Time=2 | 361 | .687 | 0.842 | 0.003 | 0.053 |
| Williams 2016 | Depression: EPDS, Time=4; Bonding: MPAS (Total), Time=4 | 502 | .588 | 0.675 | 0.002 | 0.045 |
| Wittkowski 2007 | Blues: Kennerley Blues Scale, Time=1; Bonding: PBQ (Infant Focused Anxiety), Time=1 | 96 | .262 | 0.268 | 0.011 | 0.104 |
| Wittkowski 2007 | Blues: Kennerley Blues Scale, Time=1; Bonding: MIBS (Total), Time=1 | 96 | .357 | 0.373 | 0.011 | 0.104 |
| Wittkowski 2007 | Blues: Kennerley Blues Scale, Time=1; Bonding: MIBS (Total), Time=1 | 96 | .335 | 0.348 | 0.011 | 0.104 |
| Wittkowski 2007 | Blues: Kennerley Blues Scale, Time=1; Bonding: PBQ (Total), Time=1 | 96 | .457 | 0.494 | 0.011 | 0.104 |
| Wittkowski 2010 | Depression: BDI-II, Time=3; Bonding: PBQ (Total), Time=3 | 132 | .712 | 0.891 | 0.008 | 0.088 |
| Yoshida 2012 | Depression: EPDS, Time=1; Bonding: MIBS-J (Anger & Rejection), Time=1 | 554 | .360 | 0.377 | 0.002 | 0.043 |
| Yoshida 2012 | Depression: EPDS, Time=2; Bonding: MIBS-J (Anger & Rejection), Time=2 | 554 | .514 | 0.568 | 0.002 | 0.043 |
| Yoshida 2012 | Depression: EPDS, Time=3; Bonding: MIBS-J (Anger & Rejection), Time=3 | 554 | .392 | 0.414 | 0.002 | 0.043 |
| Yoshida 2012 | Depression: EPDS, Time=1; Bonding: MIBS-J (Lack of Affection), Time=1 | 554 | .181 | 0.183 | 0.002 | 0.043 |
| Yoshida 2012 | Depression: EPDS, Time=2; Bonding: MIBS-J (Lack of Affection), Time=2 | 554 | .224 | 0.228 | 0.002 | 0.043 |
| Yoshida 2012 | Depression: EPDS, Time=3; Bonding: MIBS-J (Lack of Affection), Time=3 | 554 | .199 | 0.202 | 0.002 | 0.043 |
| Zhang 2017 | Depression: HAMD, Time=6; Bonding: MAI (Total), Time=6 | 255 | .615 | 0.717 | 0.004 | 0.063 |

eTable 7. Forest plot data meta-analysis of association between depression and bonding.

| Study | Effect size descriptor | N | r | Fisher Z | Fisher Z variance | Standard error |
| --- | --- | --- | --- | --- | --- | --- |
| Agbagwara-Osuji 2015 | Depression: EPDS, Time=PG3; Bonding: MIBS (Total), Time=1 | 223 | .250 | 0.255 | 0.005 | 0.067 |
| Agbagwara-Osuji 2015 | Depression: EPDS, Time=PG3; Bonding: MIBS (Total), Time=2 | 223 | .360 | 0.377 | 0.005 | 0.067 |
| Agbagwara-Osuji 2015 | Depression: EPDS, Time=2; Bonding: MIBS (Total), Time=2 | 223 | .400 | 0.424 | 0.005 | 0.067 |
| Agbagwara-Osuji 2015 | Depression: EPDS, Time=2; Bonding: MIBS (Total), Time=1 | 223 | .390 | 0.412 | 0.005 | 0.067 |
| Badr 2018 | Depression: Historical, Time=H; Bonding: PBQ (Total), Time=2 | 125 | .390 | 0.412 | 0.008 | 0.091 |
| Badr 2018 | Depression: EPDS, Time=1; Bonding: PBQ (Total), Time=2 | 125 | .250 | 0.255 | 0.008 | 0.091 |
| Behrendt 2019 | Depression: BDI-II, Time=4; Bonding: MPAS (Total), Time=4 | 61 | .261 | 0.267 | 0.017 | 0.131 |
| Bienfait 2011 | Depression: EPDS, Time=1; Bonding: MIBS (Total), Time=1 | 78 | .110 | 0.110 | 0.013 | 0.115 |
| Bonacquisti 2020 | Depression: EPDS, Time=6; Bonding: MAI (Total), Time=6 | 127 | .127 | 0.128 | 0.008 | 0.090 |
| Busonera 2017 | Depression: CES-D, Time=3; Bonding: PBQ (Total), Time=3 | 123 | .630 | 0.741 | 0.008 | 0.091 |
| Carter 2015 | Depression: EPDS, Time=6; Bonding: MPAS (Total), Time=6 | 43 | .483 | 0.527 | 0.025 | 0.158 |
| Chenery 2013 | Depression: EPDS, Time=2; Bonding: MPAS (Total), Time=2 | 64 | .538 | 0.601 | 0.016 | 0.128 |
| Chrzan-Dętkoś 2015 | Depression: EPDS, Time=2; Bonding: PBQ (Infant focused anxiety)*, Time=2 | 64 | .350 | 0.365 | 0.016 | 0.128 |
| Chrzan-Dętkoś 2015 | Depression: EPDS, Time=2; Bonding: PBQ (Impaired bonding), Time=2 | 64 | .370 | 0.388 | 0.016 | 0.128 |
| Condon 1998 | Depression: ZSDS, Time=3; Bonding: MPAS (Total), Time=3 | 210 | .340 | 0.354 | 0.005 | 0.070 |
| Condon 1998 | Depression: HADS, Time=3; Bonding: MPAS (Total), Time=3 | 210 | .460 | 0.497 | 0.005 | 0.070 |
| Cuijlits 2016 | Depression: EPDS, Time=4; Bonding: PPBS (Total), Time=4 | 1050 | .230 | 0.234 | 0.001 | 0.031 |
| Cuijlits 2016 | Depression: EPDS, Time=4; Bonding: PPBS (Total), Time=4 | 1050 | .250 | 0.255 | 0.001 | 0.031 |
| Daglar 2018 | Depression: CES-D, Time=PG3; Bonding: MIBS (Total), Time=2 | 227 | .174 | 0.176 | 0.004 | 0.067 |
| Daglar 2018 | Depression: CES-D, Time=2; Bonding: MIBS (Total), Time=2 | 227 | .221 | 0.225 | 0.004 | 0.067 |
| Dubber 2015 | Depression: EPDS, Time=PG3; Bonding: PBQ (Total), Time=2 | 54 | .262 | 0.268 | 0.020 | 0.140 |
| Dubber 2015 | Depression: EPDS, Time=2; Bonding: PBQ (Total), Time=2 | 77 | .417 | 0.444 | 0.014 | 0.116 |
| Edhborg 2011 | Depression: EPDS, Time=2; Bonding: PBQ (Total), Time=2 | 671 | .339 | 0.353 | 0.001 | 0.039 |
| Faisal-Cury 2020 | Mod-severe Depression: PHQ9, Time=4; Bonding: PBQ (Total), Time=4 | 266 | .430 | 0.460 | 0.004 | 0.062 |
| Faisal-Cury 2020 | Mild Depression: PHQ9, Time=4; Bonding: PBQ (Total), Time=4 | 281 | .176 | 0.178 | 0.004 | 0.060 |
| Fallon 2019 | Depression: BDI-II, Time=3; Bonding: PBQ (Total), Time=3 | 261 | .590 | 0.678 | 0.004 | 0.062 |
| Farré-Sender 2018 | Depression: EPDS, Time=PG; Bonding: PBQ (Total), Time=2 | 251 | .166 | 0.168 | 0.004 | 0.064 |
| Figueiredo 2009 | Depression: EPDS, Time=1; Bonding: MIBS (Total), Time=1 | 315 | .280 | 0.288 | 0.003 | 0.057 |
| Fijalkowska 2019 | Depression: EPDS, Time=2; Bonding: MPAS (Total), Time=2 | 35 | .831 | 1.191 | 0.031 | 0.177 |
| Fransson 2020 | Depression: EPDS, Time=PG2; Bonding: PBQ (Total), Time=4 | 1090 | .210 | 0.213 | 0.001 | 0.030 |
| Fransson 2020 | Depression: EPDS, Time=PG3; Bonding: PBQ (Total), Time=4 | 1090 | .238 | 0.243 | 0.001 | 0.030 |
| Fransson 2020 | Depression: EPDS, Time=2; Bonding: PBQ (Total), Time=4 | 1090 | .285 | 0.293 | 0.001 | 0.030 |
| Fransson 2020 | Depression: EPDS, Time=4; Bonding: PBQ (Total), Time=4 | 1090 | .290 | 0.299 | 0.001 | 0.030 |
| Friedman 2008 | Depression: EPDS, Time=3; Bonding: PBQ (Total), Time=3 | 50 | .417 | 0.444 | 0.021 | 0.146 |
| Garcia-Esteve 2016 | Depression: EPDS, Time=2; Bonding: PBQ (Infant focused anxiety)*, Time=2 | 840 | .510 | 0.563 | 0.001 | 0.035 |
| Garcia-Esteve 2016 | Depression: EPDS, Time=2; Bonding: PBQ (General factor)*, Time=2 | 840 | .590 | 0.678 | 0.001 | 0.035 |
| Garcia-Esteve 2016 | Depression: EPDS, Time=2; Bonding: PBQ (Impaired bonding)*, Time=2 | 840 | .530 | 0.590 | 0.001 | 0.035 |
| Garcia-Esteve 2016 | Depression: EPDS, Time=2; Bonding: PBQ (Lack of enjoyment)*, Time=2 | 840 | .400 | 0.424 | 0.001 | 0.035 |
| Garcia-Esteve 2016 | Depression: EPDS, Time=2; Bonding: PBQ (Rejection & risk of abuse)*, Time=2 | 840 | .320 | 0.332 | 0.001 | 0.035 |
| Gashe 2011 | Depression: EPDS, Time=4; Bonding: PBQ (Impaired bonding), Time=4 | 190 | .540 | 0.604 | 0.005 | 0.073 |
| Gashe 2011 | Depression: EPDS, Time=4; Bonding: PBQ (Infant focused anxiety)*, Time=4 | 190 | .470 | 0.510 | 0.005 | 0.073 |
| Gashe 2011 | Depression: EPDS, Time=4; Bonding: PBQ (Rejection & Anger), Time=4 | 190 | .450 | 0.485 | 0.005 | 0.073 |
| Hairston 2016 | Depression: EPDS, Time=3; Bonding: PBQ (Total), Time=3 | 152 | .406 | 0.431 | 0.007 | 0.082 |
| Hairston 2018 | Depression: EPDS, Time=2; Bonding: PBQ (Total), Time=2 | 114 | .446 | 0.480 | 0.009 | 0.095 |
| Handelzalts 2019 | Depression: EPDS, Time=3; Bonding: PBQ (Total), Time=3 | 504 | .480 | 0.523 | 0.002 | 0.045 |
| Herguner 2014 | Depression: EPDS, Time=3; Bonding: MAI (Total), Time=3 | 80 | .591 | 0.679 | 0.013 | 0.114 |
| Høivik 2013 | Depression: EPDS, Time=2; Bonding: PBQ (Total), Time=2 | 20 | .758 | 0.991 | 0.059 | 0.243 |
| Høivik 2013 | Depression: EPDS, Time=3; Bonding: PBQ (Total), Time=2 | 20 | .801 | 1.101 | 0.059 | 0.243 |
| Høivik 2013 | Depression: EPDS, Time=2; Bonding: MABISC (Total), Time=2 | 52 | .546 | 0.613 | 0.020 | 0.143 |
| Høivik 2013 | Depression: EPDS, Time=3; Bonding: MABISC (Total), Time=2 | 52 | .588 | 0.675 | 0.020 | 0.143 |
| Høivik 2013 | Depression: EPDS, Time=2; Bonding: MABISC (Total), Time=3 | 52 | .315 | 0.326 | 0.020 | 0.143 |
| Høivik 2013 | Depression: EPDS, Time=3; Bonding: MABISC (Total), Time=3 | 55 | .488 | 0.533 | 0.019 | 0.139 |
| Høivik 2013 | Depression: EPDS, Time=2; Bonding: PBQ (Total), Time=3 | 35 | .520 | 0.576 | 0.031 | 0.177 |
| Høivik 2013 | Depression: EPDS, Time=3; Bonding: PBQ (Total), Time=3 | 35 | .721 | 0.910 | 0.031 | 0.177 |
| Hrishikesh 2019 | Depression: EPDS, Time=2; Bonding: PBQ (Total), Time=2 | 100 | .550 | 0.618 | 0.010 | 0.102 |
| Jones 2011 | Depression: EPDS, Time=NR; Bonding: MPHI (Rel'ship w baby), Time=6 | 178 | .310 | 0.321 | 0.006 | 0.076 |
| Jordan 2014 | Depression: EPDS, Time=3; Bonding: MPAS (Total), Time=3 | 74 | .440 | 0.472 | 0.014 | 0.119 |
| Kaneko 2014 | Depression: EPDS, Time=3; Bonding: PBQ (Total), Time=3 | 1786 | .460 | 0.497 | 0.001 | 0.024 |
| Kaneko 2014 | Depression: EPDS, Time=3; Bonding: PBQ (Total), Time=3 | 1786 | .470 | 0.510 | 0.001 | 0.024 |
| Kasamatsu 2019 | Depression: EPDS, Time=2; Bonding: MIBS-J (Total), Time=4 | 83109 | .029 | 0.029 | 0.000 | 0.003 |
| Kasamatsu 2019 | Depression: EPDS, Time=4; Bonding: MIBS-J (Total), Time=4 | 83109 | .029 | 0.029 | 0.000 | 0.003 |
| Kerstis 2016 | Depression: EPDS, Time=2; Bonding: PBQ (Impaired bonding), Time=4 | 727 | .157 | 0.158 | 0.001 | 0.037 |
| Kerstis 2016 | Depression: EPDS, Time=4; Bonding: PBQ (Impaired bonding), Time=4 | 727 | .202 | 0.205 | 0.001 | 0.037 |
| Kita 2020 | Depression: HADS, Time=PG3; Bonding: MIBS-J (Anger & Rejection), Time=2 | 562 | .230 | 0.234 | 0.002 | 0.042 |
| Kita 2020 | Depression: HADS, Time=2; Bonding: MIBS-J (Anger & Rejection), Time=2 | 562 | .380 | 0.400 | 0.002 | 0.042 |
| Kita 2020 | Depression: HADS, Time=PG3; Bonding: MIBS-J (Lack of Affection), Time=2 | 562 | .270 | 0.277 | 0.002 | 0.042 |
| Kita 2020 | Depression: HADS, Time=2; Bonding: MIBS-J (Lack of Affection), Time=2 | 562 | .420 | 0.448 | 0.002 | 0.042 |
| Kleiber 2015 | Depression: EPDS, Time=3; Bonding: PBQ (Infant Focused Anxiety), Time=3 | 102 | .260 | 0.266 | 0.010 | 0.101 |
| Kleiber 2015 | Depression: EPDS, Time=3; Bonding: PBQ (Impaired bonding), Time=3 | 102 | .430 | 0.460 | 0.010 | 0.101 |
| Kleiber 2015 | Depression: EPDS, Time=3; Bonding: PBQ (Rejection & Anger), Time=3 | 102 | .210 | 0.213 | 0.010 | 0.101 |
| Kokubu 2012 | Depression: HADS, Time=PG3; Bonding: MIBS-J (Total), Time=1 | 99 | .320 | 0.332 | 0.010 | 0.102 |
| Kokubu 2012 | Depression: EPDS, Time=2; Bonding: MIBS-J (Total), Time=1 | 99 | .240 | 0.245 | 0.010 | 0.102 |
| Kokubu 2012 | Depression: HADS, Time=PG3; Bonding: MIBS-J (Total), Time=2 | 99 | .290 | 0.299 | 0.010 | 0.102 |
| Kokubu 2012 | Depression: EPDS, Time=2; Bonding: MIBS-J (Total), Time=2 | 99 | .460 | 0.497 | 0.010 | 0.102 |
| Lara-Cinisomo 2018 | Depression: EPDS, Time=PG3; Bonding: PBQ (Infant focused anxiety)*, Time=2 | 28 | .530 | 0.590 | 0.040 | 0.200 |
| Lara-Cinisomo 2018 | Depression: EPDS, Time=2; Bonding: PBQ (Infant Focused Anxiety), Time=2 | 28 | .500 | 0.549 | 0.040 | 0.200 |
| Lara-Cinisomo 2018 | Depression: EPDS, Time=2; Bonding: PBQ (Infant Focused Anxiety), Time=2 | 28 | .590 | 0.678 | 0.040 | 0.200 |
| Lara-Cinisomo 2018 | Depression: EPDS, Time=2; Bonding: PBQ (Impaired bonding), Time=2 | 28 | .420 | 0.448 | 0.040 | 0.200 |
| Lara-Cinisomo 2018 | Depression: EPDS, Time=2; Bonding: PBQ (Impaired bonding), Time=2 | 28 | .560 | 0.633 | 0.040 | 0.200 |
| Leahy-Warren 2020 | Depression: EPDS, Time=4; Bonding: MPAS (Total), Time=4 | 140 | .540 | 0.604 | 0.007 | 0.085 |
| Le Bas 2020 | Depression: EPDS, Time=PG1; Bonding: MPAS (Total), Time=2 | 1579 | .160 | 0.161 | 0.001 | 0.025 |
| Le Bas 2020 | Depression: EPDS, Time=PG2; Bonding: MPAS (Total), Time=2 | 1579 | .210 | 0.213 | 0.001 | 0.025 |
| Le Bas 2020 | Depression: EPDS, Time=PG3; Bonding: MPAS (Total), Time=2 | 1579 | .250 | 0.255 | 0.001 | 0.025 |
| Le Bas 2020 | Depression: EPDS, Time=2; Bonding: MPAS (Total), Time=2 | 1579 | .440 | 0.472 | 0.001 | 0.025 |
| Le Bas 2020 | Depression: EPDS, Time=4; Bonding: MPAS (Total), Time=2 | 1579 | .230 | 0.234 | 0.001 | 0.025 |
| Le Bas 2020 | Depression: EPDS, Time=PG1; Bonding: MPAS (Total), Time=4 | 1579 | .240 | 0.245 | 0.001 | 0.025 |
| Le Bas 2020 | Depression: EPDS, Time=PG2; Bonding: MPAS (Total), Time=4 | 1579 | .280 | 0.288 | 0.001 | 0.025 |
| Le Bas 2020 | Depression: EPDS, Time=PG3; Bonding: MPAS (Total), Time=4 | 1579 | .280 | 0.288 | 0.001 | 0.025 |
| Le Bas 2020 | Depression: EPDS, Time=2; Bonding: MPAS (Total), Time=4 | 1579 | .310 | 0.321 | 0.001 | 0.025 |
| Le Bas 2020 | Depression: EPDS, Time=4; Bonding: MPAS (Total), Time=4 | 1579 | .420 | 0.448 | 0.001 | 0.025 |
| Lehnig 2019 | Depression: BDI-II, Time=2; Bonding: PBQ (Total), Time=2 | 725 | .473 | 0.514 | 0.001 | 0.037 |
| Leserman 2011 | History of depression: MINI, Time=2; Bonding: PBQ (Total), Time=2 | 70 | .380 | 0.400 | 0.015 | 0.122 |
| Lutkiewicz 2020 | Depression: EPDS, Time=1; Bonding: PBQ (Infant Focused Anxiety), Time=1 | 150 | .305 | 0.315 | 0.007 | 0.082 |
| Lutkiewicz 2020 | Depression: PDSS, Time=1; Bonding: PBQ (Infant Focused Anxiety), Time=1 | 150 | .367 | 0.385 | 0.007 | 0.082 |
| Lutkiewicz 2020 | Depression: EPDS, Time=1; Bonding: PBQ (Risk of abuse), Time=1 | 150 | .417 | 0.444 | 0.007 | 0.082 |
| Lutkiewicz 2020 | Depression: PDSS, Time=1; Bonding: PBQ (Risk of abuse), Time=1 | 150 | .381 | 0.401 | 0.007 | 0.082 |
| Lutkiewicz 2020 | Depression: EPDS, Time=1; Bonding: PBQ (Impaired bonding), Time=1 | 150 | .456 | 0.492 | 0.007 | 0.082 |
| Lutkiewicz 2020 | Depression: PDSS, Time=1; Bonding: PBQ (Impaired bonding), Time=1 | 150 | .512 | 0.565 | 0.007 | 0.082 |
| Lutkiewicz 2020 | Depression: EPDS, Time=1; Bonding: PBQ (Rejection & Anger), Time=1 | 150 | .437 | 0.469 | 0.007 | 0.082 |
| Lutkiewicz 2020 | Depression: PDSS, Time=1; Bonding: PBQ (Rejection & Anger), Time=1 | 150 | .494 | 0.541 | 0.007 | 0.082 |
| Luz 2017 | Depression: HADS, Time=H; Bonding: MPAS (Total), Time=2 | 40 | .320 | 0.332 | 0.027 | 0.164 |
| Macdonald 2020 | Depression: SMFQ, Time=Maternal age 13yrs; Bonding: MPAS (Total), Time=4 | 476 | .147 | 0.148 | 0.002 | 0.046 |
| Macdonald 2020 | Depression: SMFQ, Time=Maternal age 15yrs; Bonding: MPAS (Total), Time=4 | 492 | .206 | 0.209 | 0.002 | 0.045 |
| Macdonald 2020 | Depression: DASS-21, Time=Maternal age 23yrs; Bonding: MPAS (Total), Time=4 | 492 | .170 | 0.171 | 0.002 | 0.045 |
| Macdonald 2020 | Depression: DASS-21, Time=Maternal age 19yrs; Bonding: MPAS (Total), Time=4 | 493 | .083 | 0.083 | 0.002 | 0.045 |
| Macdonald 2020 | Depression: SMFQ, Time=Maternal age 17yrs; Bonding: MPAS (Total), Time=4 | 510 | .156 | 0.158 | 0.002 | 0.044 |
| Macdonald 2020 | Depression: DASS-21, Time=Maternal age 27yrs; Bonding: MPAS (Total), Time=4 | 523 | .196 | 0.198 | 0.002 | 0.044 |
| Macdonald 2020 | Depression: DASS-21, Time=4; Bonding: MPAS (Total), Time=4 | 582 | .389 | 0.410 | 0.002 | 0.042 |
| Mackie 2019 | Depression: EPDS, Time=3; Bonding: MPAS (Total), Time=3 | 5 | .400 | 0.424 | 0.500 | 0.707 |
| Mason 2011 | Depression: EPDS, Time=2; Bonding: MPAS (Total), Time=2 | 276 | .380 | 0.400 | 0.004 | 0.061 |
| McErlean 2012 | Depression: EPDS, Time=3; Bonding: PBQ (Total), Time=3 | 77 | .431 | 0.461 | 0.014 | 0.116 |
| Milne 2007 | Depression: EPDS, Time=4; Bonding: MAS (Anxious to child), Time=4 | 139 | .531 | 0.592 | 0.007 | 0.086 |
| Milne 2007 | Depression: EPDS, Time=4; Bonding: MAS (Positive to child), Time=4 | 139 | .354 | 0.370 | 0.007 | 0.086 |
| Minamida 2020 | Depression: EPDS, Time=1; Bonding: PBQ (Total), Time=1 | 185 | .184 | 0.186 | 0.005 | 0.074 |
| Minamida 2020 | Depression: EPDS, Time=1; Bonding: PBQ (Total), Time=1 | 185 | .092 | 0.093 | 0.005 | 0.074 |
| Minamida 2020 | Depression: EPDS, Time=2; Bonding: PBQ (Total), Time=2 | 185 | .449 | 0.483 | 0.005 | 0.074 |
| Moehler 2006 | Depression: EPDS, Time=3; Bonding: PBQ (Total), Time=2 | 101 | .280 | 0.288 | 0.010 | 0.101 |
| Moehler 2006 | Depression: EPDS, Time=3; Bonding: PBQ (Total), Time=2 | 101 | .390 | 0.412 | 0.010 | 0.101 |
| Moehler 2006 | Depression: EPDS, Time=3; Bonding: PBQ (Total), Time=3 | 101 | .350 | 0.365 | 0.010 | 0.101 |
| Morrison 2016 | Depression: EPDS, Time=PG3; Bonding: MPAS (Total), Time=2 | 70 | .243 | 0.248 | 0.015 | 0.122 |
| Morrison 2016 | Depression: EPDS, Time=2; Bonding: MPAS (Total), Time=2 | 70 | .109 | 0.109 | 0.015 | 0.122 |
| Moser 1989 | Depression: BDI, Time=3; Bonding: PPAM (Total), Time=4 | 37 | .330 | 0.343 | 0.029 | 0.171 |
| Muzik 2017 | Depression: PPDS, Time=4; Bonding: PBQ (Total), Time=4 | 122 | .320 | 0.332 | 0.008 | 0.092 |
| Myers 2017 | Depression: EPDS & BPDS, Time=4; Bonding: MIBS (Total), Time=2 | 48 | .107 | 0.107 | 0.022 | 0.149 |
| Nagata 2000 | Depression: ZSDS , Time=1; Bonding: MAS (Anxiety re Child), Time=1 | 417 | .390 | 0.412 | 0.002 | 0.049 |
| Nagata 2000 | Depression: ZSDS , Time=1; Bonding: MAS (Core Maternal Attachment), Time=1 | 417 | .300 | 0.310 | 0.002 | 0.049 |
| Nagata 2004 | Depression: ZSDS , Time=1; Bonding: MAS (Anxiety re Child), Time=1 | 153 | .330 | 0.343 | 0.007 | 0.082 |
| Nagata 2004 | Depression: ZSDS , Time=1; Bonding: MAS (Core Maternal Attachment), Time=1 | 153 | .270 | 0.277 | 0.007 | 0.082 |
| Nakano 2019 | Depression: EPDS, Time=2; Bonding: PBQ (Total), Time=2 | 1060 | .107 | 0.107 | 0.001 | 0.031 |
| Nakano 2019 | Depression: EPDS, Time=2; Bonding: PBQ (Total), Time=2 | 1060 | .593 | 0.682 | 0.001 | 0.031 |
| Nath 2019 | Depression: EPDS, Time=PG2; Bonding: PBQ (Total), Time=3 | 400 | .213 | 0.216 | 0.003 | 0.050 |
| Nath 2019 | Depression: EPDS, Time=PG3; Bonding: PBQ (Total), Time=3 | 380 | .123 | 0.124 | 0.003 | 0.052 |
| Nath 2019 | Depression: SCID, Time=PG2; Bonding: PBQ (Total), Time=3 | 404 | .202 | 0.205 | 0.002 | 0.050 |
| Nath 2019 | Depression: EPDS, Time=PG; Bonding: PBQ (Total), Time=3 | 404 | .212 | 0.215 | 0.002 | 0.050 |
| Ngoma 2012 | Depression: EPDS, Time=2; Bonding: MIBS-J (Total), Time=2 | 42 | .470 | 0.510 | 0.026 | 0.160 |
| Ngoma 2012 | Depression: EPDS, Time=2; Bonding: MIBS-J (Total), Time=2 | 76 | .220 | 0.224 | 0.014 | 0.117 |
| Nolvi 2016 | Depression: EPDS, Time=3; Bonding: PBQ (Total), Time=4 | 102 | .253 | 0.259 | 0.010 | 0.101 |
| Nonnenmacher 2016 | Depression: SCID-I, Time=3; Bonding: PBQ (Total), Time=3 | 93 | .610 | 0.709 | 0.011 | 0.105 |
| Noyman-Veksler 2015 | Depression: EPDS, Time=2; Bonding: PBQ (Total), Time=2 | 92 | .300 | 0.310 | 0.011 | 0.106 |
| Noyman-Veksler 2015 | Depression: EPDS, Time=2; Bonding: PBQ (Total), Time=2 | 92 | .380 | 0.400 | 0.011 | 0.106 |
| Noyman-Veksler 2015 | Depression: EPDS, Time=2; Bonding: PBQ (Total), Time=2 | 92 | .300 | 0.310 | 0.011 | 0.106 |
| Noyman-Veksler 2015 | Depression: EPDS, Time=2; Bonding: PBQ (Total), Time=2 | 96 | .620 | 0.725 | 0.011 | 0.104 |
| O'Higgins 2013 | Depression: EPDS, Time=2; Bonding: MIBS (Total), Time=2 | 79 | .440 | 0.472 | 0.013 | 0.115 |
| O'Higgins 2013 | Depression: EPDS, Time=2; Bonding: MIBS (Total), Time=2 | 79 | .340 | 0.354 | 0.013 | 0.115 |
| O'Higgins 2013 | Depression: EPDS, Time=2; Bonding: MIBS (Total), Time=3 | 79 | .260 | 0.266 | 0.013 | 0.115 |
| O'Higgins 2013 | Depression: EPDS, Time=2; Bonding: MIBS (Total), Time=4 | 79 | .260 | 0.266 | 0.013 | 0.115 |
| Oddo-Sommerfeld 2016 | Depression: BDI-V, Time=PG3; Bonding: PBQ (Impaired bonding), Time=2 | 266 | .190 | 0.192 | 0.004 | 0.062 |
| Oddo-Sommerfeld 2016 | Depression: EPDS, Time=2; Bonding: PBQ (Impaired bonding), Time=2 | 266 | .440 | 0.472 | 0.004 | 0.062 |
| Ohara 2016 | Depression: EPDS, Time=1; Bonding: MIBQ (Anger & Rejection), Time=1 | 751 | .250 | 0.255 | 0.001 | 0.037 |
| Ohara 2016 | Depression: EPDS, Time=1; Bonding: MIBQ (Lack of Affection), Time=1 | 751 | .170 | 0.172 | 0.001 | 0.037 |
| Ohara 2017 (b) | Depression: EPDS, Time=PG; Bonding: MIBQ (Lack of Affection), Time=1 | 751 | .040 | 0.040 | 0.001 | 0.037 |
| Ohara 2017 (b) | Depression: EPDS, Time=PG3; Bonding: MIBQ (Lack of Affection), Time=1 | 751 | .050 | 0.050 | 0.001 | 0.037 |
| Ohara 2017 (b) | Depression: EPDS, Time=PG; Bonding: MIBQ (Anger & Rejection), Time=1 | 751 | .110 | 0.110 | 0.001 | 0.037 |
| Ohara 2017 (b) | Depression: EPDS, Time=PG3; Bonding: MIBQ (Anger & Rejection), Time=1 | 751 | .110 | 0.110 | 0.001 | 0.037 |
| Ohara 2018 | Depression: EPDS, Time=PG2; Bonding: MIBQ (Anger & Rejection), Time=2 | 855 | .130 | 0.131 | 0.001 | 0.034 |
| Ohara 2018 | Depression: EPDS, Time=2; Bonding: MIBQ (Anger & Rejection), Time=2 | 855 | .310 | 0.321 | 0.001 | 0.034 |
| Ohara 2018 | Depression: EPDS, Time=PG2; Bonding: MIBQ (Lack of Affection), Time=2 | 855 | .050 | 0.050 | 0.001 | 0.034 |
| Ohara 2018 | Depression: EPDS, Time=2; Bonding: MIBQ (Lack of Affection), Time=2 | 855 | .270 | 0.277 | 0.001 | 0.034 |
| Ohashi 2016 | Depression: EPDS, Time=2; Bonding: PBQ (Anger & Restrictedness)*, Time=2 | 364 | .490 | 0.536 | 0.003 | 0.053 |
| Ohashi 2016 | Depression: EPDS, Time=2; Bonding: PBQ (Lack of Affection)*, Time=2 | 364 | .210 | 0.213 | 0.003 | 0.053 |
| Ohashi 2016 | Depression: EPDS, Time=2; Bonding: PBQ (Rejection & Fear)*, Time=2 | 364 | .320 | 0.332 | 0.003 | 0.053 |
| Örün 2013 | Depression: BSI, Time=1; Bonding: PBQ (Total), Time=2 | 189 | .225 | 0.229 | 0.005 | 0.073 |
| Örün 2013 | Depression: BSI, Time=1; Bonding: MIBS (Total), Time=2 | 189 | .150 | 0.151 | 0.005 | 0.073 |
| Örün 2013 | Depression: EPDS, Time=2; Bonding: MIBS (Total), Time=2 | 189 | .377 | 0.397 | 0.005 | 0.073 |
| Örün 2013 | Depression: EPDS, Time=2; Bonding: PBQ (Total), Time=2 | 189 | .449 | 0.483 | 0.005 | 0.073 |
| Oskovi-Kaplan 2020 | Depression: EPDS, Time=1; Bonding: MAI (Total), Time=1 | 223 | .418 | 0.445 | 0.005 | 0.067 |
| Pearson 2013 | Depression: EPDS, Time=NR; Bonding: PBQ (Total), Time=3 | 49 | .200 | 0.203 | 0.022 | 0.147 |
| Rados 2020 | Depression: EPDS, Time=6; Bonding: PBQ (Total), Time=6 | 603 | .550 | 0.618 | 0.002 | 0.041 |
| Rados 2020 | Depression: EPDS, Time=6; Bonding: PBQ (Total), Time=6 | 603 | .720 | 0.908 | 0.002 | 0.041 |
| Reck 2006 | Depression: SCID-I, Time=2; Bonding: PBQ (Total), Time=2 | 862 | .190 | 0.192 | 0.001 | 0.034 |
| Reck 2015 | Depression: SCID-I, Time=3; Bonding: PBQ (Total), Time=3 | 63 | .450 | 0.485 | 0.017 | 0.129 |
| Riera-Martin 2018 | Depression: EPDS, Time=4; Bonding: MPAS (Total), Time=4 | 571 | .410 | 0.436 | 0.002 | 0.042 |
| Robakis 2015 | Depression: EPDS, Time=3; Bonding: MIBS (Total), Time=2 | 90 | .272 | 0.279 | 0.011 | 0.107 |
| Robakis 2015 | Depression: EPDS, Time=2; Bonding: MIBS (Total), Time=3 | 91 | .337 | 0.351 | 0.011 | 0.107 |
| Robakis 2015 | Depression: EPDS, Time=3; Bonding: MIBS (Total), Time=2 | 95 | .375 | 0.394 | 0.011 | 0.104 |
| Robakis 2015 | Depression: EPDS, Time=PG3; Bonding: MIBS (Total), Time=3 | 97 | .341 | 0.355 | 0.011 | 0.103 |
| Robakis 2015 | Depression: EPDS, Time=2; Bonding: MIBS (Total), Time=3 | 97 | .344 | 0.359 | 0.011 | 0.103 |
| Robakis 2015 | Depression: EPDS, Time=3; Bonding: MIBS (Total), Time=3 | 97 | .384 | 0.405 | 0.011 | 0.103 |
| Robakis 2015 | Depression: EPDS, Time=2; Bonding: MIBS (Total), Time=2 | 105 | .302 | 0.312 | 0.010 | 0.099 |
| Robakis 2015 | Depression: EPDS, Time=2; Bonding: MIBS (Total), Time=2 | 106 | .383 | 0.404 | 0.010 | 0.099 |
| Robakis 2015 | Depression: EPDS, Time=PG3; Bonding: MIBS (Total), Time=2 | 107 | .400 | 0.424 | 0.010 | 0.098 |
| Robakis 2015 | Depression: EPDS, Time=2; Bonding: MIBS (Total), Time=2 | 107 | .350 | 0.365 | 0.010 | 0.098 |
| Robakis 2015 | Depression: EPDS, Time=PG3; Bonding: MIBS (Total), Time=2 | 118 | .309 | 0.319 | 0.009 | 0.093 |
| Robakis 2015 | Depression: EPDS, Time=2; Bonding: MIBS (Total), Time=2 | 118 | .476 | 0.518 | 0.009 | 0.093 |
| Rossen 2019 | Depression: DASS-21, Time=2; Bonding: MPAS (Total), Time=2 | 308 | .370 | 0.388 | 0.003 | 0.057 |
| Rossen 2019 | Depression: DASS-21, Time=2; Bonding: MPAS (Total), Time=4 | 308 | .320 | 0.332 | 0.003 | 0.057 |
| Sawyer Cohen 2010 | Depression: EPDS, Time=6; Bonding: MAI (Total), Time=6 | 180 | .315 | 0.326 | 0.006 | 0.075 |
| Sawyer Cohen 2010 | Depression: EPDS, Time=PG; Bonding: MAI (Total), Time=6 | 186 | .108 | 0.108 | 0.005 | 0.074 |
| Scopesi 2004 | Depression: SRT, Time=2; Bonding: MPAS (Total), Time=2 | 208 | .323 | 0.335 | 0.005 | 0.070 |
| Seng 2013 | Depression: CIDI-D, Time=H; Bonding: PBQ (Total), Time=2 | 566 | .121 | 0.122 | 0.002 | 0.042 |
| Sockol 2014 | Depression: EPDS, Time=2; Bonding: PBQ (Impaired bonding), Time=2 | 180 | .200 | 0.203 | 0.006 | 0.075 |
| Sockol 2014 | Depression: EPDS, Time=2; Bonding: PBQ (Infant Focused Anxiety), Time=2 | 180 | .200 | 0.203 | 0.006 | 0.075 |
| Sockol 2014 | Depression: EPDS, Time=2; Bonding: PBQ (Rejection & Anger), Time=2 | 180 | .200 | 0.203 | 0.006 | 0.075 |
| Sockol 2014 | Depression: EPDS, Time=2; Bonding: PBQ (Risk of abuse), Time=2 | 180 | .200 | 0.203 | 0.006 | 0.075 |
| Suetsugu 2015 | Depression: EPDS, Time=2; Bonding: PBQ (Total), Time=2 | 244 | .559 | 0.631 | 0.004 | 0.064 |
| Sun-Hee 2019 | Depression: Likert questions, Time=2; Bonding: MAI (Total), Time=2 | 217 | .536 | 0.599 | 0.005 | 0.068 |
| Talmon 2019 | Depression: EPDS, Time=PG; Bonding: PBQ (Impaired bonding), Time=2 | 394 | .140 | 0.141 | 0.003 | 0.051 |
| Talmon 2019 | Depression: EPDS, Time=2; Bonding: PBQ (Impaired bonding), Time=2 | 394 | .470 | 0.510 | 0.003 | 0.051 |
| Talmon 2019 | Depression: EPDS, Time=PG; Bonding: PBQ (Infant Focused Anxiety), Time=2 | 394 | .220 | 0.224 | 0.003 | 0.051 |
| Talmon 2019 | Depression: EPDS, Time=2; Bonding: PBQ (Infant Focused Anxiety), Time=2 | 394 | .550 | 0.618 | 0.003 | 0.051 |
| Talmon 2019 | Depression: EPDS, Time=PG; Bonding: PBQ (Rejection & Anger), Time=2 | 394 | .090 | 0.090 | 0.003 | 0.051 |
| Talmon 2019 | Depression: EPDS, Time=2; Bonding: PBQ (Rejection & Anger), Time=2 | 394 | .440 | 0.472 | 0.003 | 0.051 |
| Taylor 2005 | Depression: EPDS, Time=1; Bonding: MIBS (Total), Time=1 | 144 | .155 | 0.156 | 0.007 | 0.084 |
| Taylor 2005 | Depression: EPDS, Time=2; Bonding: MIBS (Total), Time=1 | 144 | .057 | 0.057 | 0.007 | 0.084 |
| Taylor 2005 | Depression: EPDS, Time=1; Bonding: MIBS (Total), Time=2 | 144 | .244 | 0.249 | 0.007 | 0.084 |
| Taylor 2005 | Depression: EPDS, Time=2; Bonding: MIBS (Total), Time=2 | 144 | .310 | 0.321 | 0.007 | 0.084 |
| Taylor 2005 | Depression: EPDS, Time=1; Bonding: MIBS (Total), Time=2 | 144 | .078 | 0.078 | 0.007 | 0.084 |
| Taylor 2005 | Depression: EPDS, Time=2; Bonding: MIBS (Total), Time=2 | 144 | .181 | 0.183 | 0.007 | 0.084 |
| Tester-Jones 2015 | Depression: EPDS, Time=4; Bonding: PBQ (Total), Time=4 | 203 | .530 | 0.590 | 0.005 | 0.071 |
| Tietz 2014 | Depression: EPDS, Time=3; Bonding: PBQ (Total), Time=3 | 78 | .492 | 0.539 | 0.013 | 0.115 |
| Tikotzky 2016 | Depression: EPDS, Time=4; Bonding: PBQ (Total), Time=4 | 80 | .590 | 0.678 | 0.013 | 0.114 |
| Tikotzky 2016 | Depression: EPDS, Time=4; Bonding: MPAS (Total), Time=4 | 80 | .390 | 0.412 | 0.013 | 0.114 |
| Tolja 2020 | Depression: EPDS, Time=4; Bonding: PBQ (Total), Time=4 | 241 | .520 | 0.576 | 0.004 | 0.065 |
| Tolja 2020 | Depression: Historical, Time=4; Bonding: PBQ (Total), Time=4 | 241 | .420 | 0.448 | 0.004 | 0.065 |
| VanBussel 2010 | Depression: EPDS, Time=3; Bonding: MPAS (Total), Time=3 | 202 | .320 | 0.332 | 0.005 | 0.071 |
| VanBussel 2010 | Depression: EPDS, Time=3; Bonding: PBQ (Total), Time=3 | 202 | .390 | 0.412 | 0.005 | 0.071 |
| VanBussel 2010 | Depression: EPDS, Time=3; Bonding: MIBS (Total), Time=3 | 202 | .290 | 0.299 | 0.005 | 0.071 |
| VanBussel 2010 | Depression: EPDS, Time=2; Bonding: MPAS (Total), Time=2 | 263 | .300 | 0.310 | 0.004 | 0.062 |
| VanBussel 2010 | Depression: EPDS, Time=2; Bonding: PBQ (Total), Time=2 | 263 | .410 | 0.436 | 0.004 | 0.062 |
| VanBussel 2010 | Depression: EPDS, Time=2; Bonding: MIBS (Total), Time=2 | 263 | .370 | 0.388 | 0.004 | 0.062 |
| Vengadavaradan 2019 | Depression: EPDS, Time=6; Bonding: PBQ (Total), Time=6 | 250 | .528 | 0.587 | 0.004 | 0.064 |
| Wikman 2020 | Depression: EPDS, Time=PG; Bonding: PBQ (Total), Time=2 | 209 | .092 | 0.092 | 0.005 | 0.070 |
| Wikman 2020 | Depression: EPDS, Time=2; Bonding: PBQ (Total), Time=2 | 270 | .631 | 0.743 | 0.004 | 0.061 |
| Wikman 2020 | Depression: EPDS, Time=4; Bonding: PBQ (Total), Time=2 | 132 | .666 | 0.804 | 0.008 | 0.088 |
| Wikman 2020 | Depression: EPDS, Time=6; Bonding: PBQ (Total), Time=2 | 361 | .687 | 0.842 | 0.003 | 0.053 |
| Williams 2016 | Depression: EPDS, Time=4; Bonding: MPAS (Total), Time=4 | 502 | .588 | 0.675 | 0.002 | 0.045 |
| Wittkowski 2010 | Depression: BDI-II, Time=3; Bonding: PBQ (Total), Time=3 | 132 | .712 | 0.891 | 0.008 | 0.088 |
| Yoshida 2012 | Depression: EPDS, Time=1; Bonding: MIBS-J (Anger & Rejection), Time=1 | 554 | .360 | 0.377 | 0.002 | 0.043 |
| Yoshida 2012 | Depression: EPDS, Time=2; Bonding: MIBS-J (Anger & Rejection), Time=2 | 554 | .514 | 0.568 | 0.002 | 0.043 |
| Yoshida 2012 | Depression: EPDS, Time=3; Bonding: MIBS-J (Anger & Rejection), Time=3 | 554 | .392 | 0.414 | 0.002 | 0.043 |
| Yoshida 2012 | Depression: EPDS, Time=1; Bonding: MIBS-J (Lack of Affection), Time=1 | 554 | .181 | 0.183 | 0.002 | 0.043 |
| Yoshida 2012 | Depression: EPDS, Time=2; Bonding: MIBS-J (Lack of Affection), Time=2 | 554 | .224 | 0.228 | 0.002 | 0.043 |
| Yoshida 2012 | Depression: EPDS, Time=3; Bonding: MIBS-J (Lack of Affection), Time=3 | 554 | .199 | 0.202 | 0.002 | 0.043 |
| Zhang 2017 | Depression: HAMD, Time=6; Bonding: MAI (Total), Time=6 | 255 | .615 | 0.717 | 0.004 | 0.063 |

eTable 8. Forest plot data for meta-analysis of association between anxiety and bonding.

| Study | Effect size descriptor | N | r | Fisher Z | Fisher Z variance | Standard error |
| --- | --- | --- | --- | --- | --- | --- |
| Agbagwara-Osuji 2015 | State anxiety: STAI, Time=PG3; Bonding: MIBS (Total), Time=1 | 223 | .200 | 0.203 | 0.005 | 0.067 |
| Agbagwara-Osuji 2015 | Trait anxiety: STAI, Time=PG3; Bonding: MIBS (Total), Time=1 | 223 | .250 | 0.255 | 0.005 | 0.067 |
| Agbagwara-Osuji 2015 | State anxiety: STAI, Time=PG3; Bonding: MIBS (Total), Time=2 | 223 | .290 | 0.299 | 0.005 | 0.067 |
| Agbagwara-Osuji 2015 | Trait anxiety: STAI, Time=PG3; Bonding: MIBS (Total), Time=2 | 223 | .300 | 0.310 | 0.005 | 0.067 |
| Agbagwara-Osuji 2015 | State anxiety: STAI, Time=2; Bonding: MIBS (Total), Time=2 | 223 | .370 | 0.388 | 0.005 | 0.067 |
| Agbagwara-Osuji 2015 | Trait anxiety: STAI, Time=2; Bonding: MIBS (Total), Time=2 | 223 | .360 | 0.377 | 0.005 | 0.067 |
| Agbagwara-Osuji 2015 | State anxiety: STAI, Time=2; Bonding: MIBS (Total), Time=1 | 223 | .340 | 0.354 | 0.005 | 0.067 |
| Agbagwara-Osuji 2015 | Trait anxiety: STAI, Time=2; Bonding: MIBS (Total), Time=1 | 223 | .330 | 0.343 | 0.005 | 0.067 |
| Bonacquisti 2020 | Anxiety: DASS-42, Time=6; Bonding: MAI (Total), Time=6 | 127 | .199 | 0.202 | 0.008 | 0.090 |
| Condon 1998 | Anxiety: HADS, Time=3; Bonding: MPAS (Total), Time=3 | 210 | .340 | 0.354 | 0.005 | 0.070 |
| Daglar 2018 | Anxiety: BAI, Time=PG3; Bonding: MIBS (Total), Time=2 | 227 | .115 | 0.116 | 0.004 | 0.067 |
| Daglar 2018 | Anxiety: BAI, Time=2; Bonding: MIBS (Total), Time=2 | 227 | .151 | 0.152 | 0.004 | 0.067 |
| Dubber 2015 | State anxiety: STAI, Time=2; Bonding: PBQ (Total), Time=2 | 79 | .197 | 0.200 | 0.013 | 0.115 |
| Dubber 2015 | State anxiety: STAI, Time=PG3; Bonding: PBQ (Total), Time=2 | 54 | .199 | 0.202 | 0.020 | 0.140 |
| Dubber 2015 | Trait anxiety: STAI, Time=2; Bonding: PBQ (Total), Time=2 | 77 | .315 | 0.326 | 0.014 | 0.116 |
| Dubber 2015 | Trait anxiety: STAI, Time=PG3; Bonding: PBQ (Total), Time=2 | 55 | .247 | 0.252 | 0.019 | 0.139 |
| Edhborg 2011 | State anxiety: STAI, Time=2; Bonding: PBQ (Total), Time=2 | 671 | .404 | 0.428 | 0.001 | 0.039 |
| Fallon 2019 | State anxiety: STAI, Time=3; Bonding: PBQ (Total), Time=3 | 261 | .560 | 0.633 | 0.004 | 0.062 |
| Fallon 2019 | Trait anxiety: STAI, Time=3; Bonding: PBQ (Total), Time=3 | 261 | .550 | 0.618 | 0.004 | 0.062 |
| Farré-Sender 2018 | State anxiety: STAI, Time=PG; Bonding: PBQ (Total), Time=2 | 249 | .189 | 0.191 | 0.004 | 0.064 |
| Friedman 2008 | Anxiety: SCL-90, Time=3; Bonding: PBQ (Total), Time=3 | 50 | .330 | 0.343 | 0.021 | 0.146 |
| Kita 2020 | Anxiety: HADS, Time=PG3; Bonding: MIBS-J (Anger & Rejection), Time=2 | 562 | .210 | 0.213 | 0.002 | 0.042 |
| Kita 2020 | Anxiety: HADS, Time=2; Bonding: MIBS-J (Anger & Rejection), Time=2 | 562 | .390 | 0.412 | 0.002 | 0.042 |
| Kita 2020 | Anxiety: HADS, Time=PG3; Bonding: MIBS-J (Lack of Affection), Time=2 | 562 | .150 | 0.151 | 0.002 | 0.042 |
| Kita 2020 | Anxiety: HADS, Time=2; Bonding: MIBS-J (Lack of Affection), Time=2 | 562 | .320 | 0.332 | 0.002 | 0.042 |
| Kokubu 2012 | Anxiety: HADS, Time=PG3; Bonding: MIBS-J (Total), Time=1 | 99 | .180 | 0.182 | 0.010 | 0.102 |
| Kokubu 2012 | Anxiety: HADS, Time=PG3; Bonding: MIBS-J (Total), Time=2 | 99 | .310 | 0.321 | 0.010 | 0.102 |
| Le Bas 2020 | Anxiety: DASS-21, Time=PG1; Bonding: MPAS (Total), Time=2 | 1579 | .080 | 0.080 | 0.001 | 0.025 |
| Le Bas 2020 | Anxiety: DASS-21, Time=PG2; Bonding: MPAS (Total), Time=2 | 1579 | .110 | 0.110 | 0.001 | 0.025 |
| Le Bas 2020 | Anxiety: DASS-21, Time=PG3; Bonding: MPAS (Total), Time=2 | 1579 | .120 | 0.121 | 0.001 | 0.025 |
| Le Bas 2020 | Anxiety: DASS-21, Time=2; Bonding: MPAS (Total), Time=2 | 1579 | .230 | 0.234 | 0.001 | 0.025 |
| Le Bas 2020 | Anxiety: DASS-21, Time=4; Bonding: MPAS (Total), Time=2 | 1579 | .110 | 0.110 | 0.001 | 0.025 |
| Le Bas 2020 | Anxiety: DASS-21, Time=PG1; Bonding: MPAS (Total), Time=4 | 1579 | .110 | 0.110 | 0.001 | 0.025 |
| Le Bas 2020 | Anxiety: DASS-21, Time=PG2; Bonding: MPAS (Total), Time=4 | 1579 | .140 | 0.141 | 0.001 | 0.025 |
| Le Bas 2020 | Anxiety: DASS-21, Time=PG3; Bonding: MPAS (Total), Time=4 | 1579 | .130 | 0.131 | 0.001 | 0.025 |
| Le Bas 2020 | Anxiety: DASS-21, Time=2; Bonding: MPAS (Total), Time=4 | 1579 | .170 | 0.172 | 0.001 | 0.025 |
| Le Bas 2020 | Anxiety: DASS-21, Time=4; Bonding: MPAS (Total), Time=4 | 1579 | .260 | 0.266 | 0.001 | 0.025 |
| Lehnig 2019 | Anxiety: SCL-90 R, Time=2; Bonding: PBQ (Total), Time=2 | 725 | .289 | 0.297 | 0.001 | 0.037 |
| Lutkiewicz 2020 | Anxiety: GAD-7, Time=1; Bonding: PBQ (Infant Focused Anxiety), Time=1 | 150 | .194 | 0.196 | 0.007 | 0.082 |
| Lutkiewicz 2020 | Anxiety: GAD-7, Time=1; Bonding: PBQ (Risk of abuse), Time=1 | 150 | .266 | 0.273 | 0.007 | 0.082 |
| Lutkiewicz 2020 | Anxiety: GAD-7, Time=1; Bonding: PBQ (Impaired bonding), Time=1 | 150 | .316 | 0.327 | 0.007 | 0.082 |
| Lutkiewicz 2020 | Anxiety: GAD-7, Time=1; Bonding: PBQ (Rejection & Anger), Time=1 | 150 | .345 | 0.360 | 0.007 | 0.082 |
| Luz 2017 | Anxiety: HADS, Time=PG3; Bonding: MPAS (Total), Time=2 | 40 | -.050 | -0.050 | 0.027 | 0.164 |
| Macdonald 2020 | Anxiety: RBPC, Time=Maternal age 13yrs; Bonding: MPAS (Total), Time=4 | 474 | .162 | 0.163 | 0.002 | 0.046 |
| Macdonald 2020 | Anxiety: DASS-21, Time=Maternal age 23yrs; Bonding: MPAS (Total), Time=4 | 492 | .108 | 0.108 | 0.002 | 0.045 |
| Macdonald 2020 | Anxiety: DASS-21, Time=Maternal age 19yrs; Bonding: MPAS (Total), Time=4 | 493 | .105 | 0.105 | 0.002 | 0.045 |
| Macdonald 2020 | Anxiety: RCMA-SF, Time=Maternal age 15yrs; Bonding: MPAS (Total), Time=4 | 494 | .181 | 0.182 | 0.002 | 0.045 |
| Macdonald 2020 | Anxiety: RCMA-SF, Time=Maternal age 17yrs; Bonding: MPAS (Total), Time=4 | 510 | .177 | 0.179 | 0.002 | 0.044 |
| Macdonald 2020 | Anxiety: DASS-21, Time=Maternal age 27yrs; Bonding: MPAS (Total), Time=4 | 523 | .163 | 0.164 | 0.002 | 0.044 |
| Macdonald 2020 | Anxiety: DASS-21, Time=4; Bonding: MPAS (Total), Time=4 | 582 | .273 | 0.280 | 0.002 | 0.042 |
| Nath 2019 | Anxiety: SCID, Time=PG2; Bonding: PBQ (Total), Time=3 | 404 | .127 | 0.127 | 0.002 | 0.050 |
| Nolvi 2016 | State anxiety: STAI, Time=3; Bonding: PBQ (Total), Time=4 | 102 | .260 | 0.266 | 0.010 | 0.101 |
| Oddo-Sommerfeld 2016 | State anxiety: STADI, Time=PG3; Bonding: PBQ (Impaired bonding), Time=2 | 266 | .190 | 0.192 | 0.004 | 0.062 |
| Oddo-Sommerfeld 2016 | State anxiety: STADI, Time=2; Bonding: PBQ (Impaired bonding), Time=2 | 266 | .360 | 0.377 | 0.004 | 0.062 |
| Ohara 2017 (b) | Anxiety: EPDS, Time=PG; Bonding: MIBQ (Lack of Affection), Time=1 | 751 | .060 | 0.060 | 0.001 | 0.037 |
| Ohara 2017 (b) | Anxiety: EPDS, Time=PG3; Bonding: MIBQ (Lack of Affection), Time=1 | 751 | .080 | 0.080 | 0.001 | 0.037 |
| Ohara 2017 (b) | Anxiety: EPDS, Time=PG; Bonding: MIBQ (Anger & Rejection), Time=1 | 751 | .110 | 0.110 | 0.001 | 0.037 |
| Ohara 2017 (b) | Anxiety: EPDS, Time=PG3; Bonding: MIBQ (Anger & Rejection), Time=1 | 751 | .160 | 0.161 | 0.001 | 0.037 |
| Ohara 2018 | Anxiety: EPDS, Time=PG2; Bonding: MIBQ (Anger & Rejection), Time=2 | 855 | .140 | 0.141 | 0.001 | 0.034 |
| Ohara 2018 | Anxiety: EPDS, Time=2; Bonding: MIBQ (Anger & Rejection), Time=2 | 855 | .290 | 0.299 | 0.001 | 0.034 |
| Ohara 2018 | Anxiety: EPDS, Time=PG2; Bonding: MIBQ (Lack of Affection), Time=2 | 855 | .020 | 0.020 | 0.001 | 0.034 |
| Ohara 2018 | Anxiety: EPDS, Time=2; Bonding: MIBQ (Lack of Affection), Time=2 | 855 | .130 | 0.131 | 0.001 | 0.034 |
| Örün 2013 | Anxiety: BSI, Time=1; Bonding: MIBS (Total), Time=2 | 189 | .185 | 0.187 | 0.005 | 0.073 |
| Sawyer Cohen 2010 | Trait anxiety: STAI, Time=6; Bonding: MAI (Total), Time=6 | 168 | .366 | 0.384 | 0.006 | 0.078 |
| Sawyer Cohen 2010 | Trait anxiety: STAI, Time=PG; Bonding: MAI (Total), Time=6 | 185 | .168 | 0.170 | 0.005 | 0.074 |
| Scopesi 2004 | Anxiety: SRT, Time=2; Bonding: MPAS (Total), Time=2 | 208 | .313 | 0.324 | 0.005 | 0.070 |
| Tietz 2014 | Anxiety: SCID-I, Time=3; Bonding: PBQ (Total), Time=3 | 78 | .380 | 0.400 | 0.013 | 0.115 |
| Tolja 2020 | Anxiety: DASS-21, Time=4; Bonding: PBQ (Total), Time=4 | 241 | .360 | 0.377 | 0.004 | 0.065 |
| VanBussel 2010 | Anxiety: HADS, Time=3; Bonding: MPAS (Total), Time=3 | 202 | .340 | 0.354 | 0.005 | 0.071 |
| VanBussel 2010 | Anxiety: HADS, Time=3; Bonding: PBQ (Total), Time=3 | 202 | .370 | 0.388 | 0.005 | 0.071 |
| VanBussel 2010 | Anxiety: HADS, Time=3; Bonding: MIBS (Total), Time=3 | 202 | .160 | 0.161 | 0.005 | 0.071 |
| VanBussel 2010 | Anxiety: HADS, Time=2; Bonding: MPAS (Total), Time=2 | 263 | .400 | 0.424 | 0.004 | 0.062 |
| VanBussel 2010 | Anxiety: HADS, Time=2; Bonding: PBQ (Total), Time=2 | 263 | .440 | 0.472 | 0.004 | 0.062 |
| VanBussel 2010 | Anxiety: HADS, Time=2; Bonding: MIBS (Total), Time=2 | 263 | .350 | 0.365 | 0.004 | 0.062 |

eFigure 1. Forest plot: Associations between Stress and Bonding.
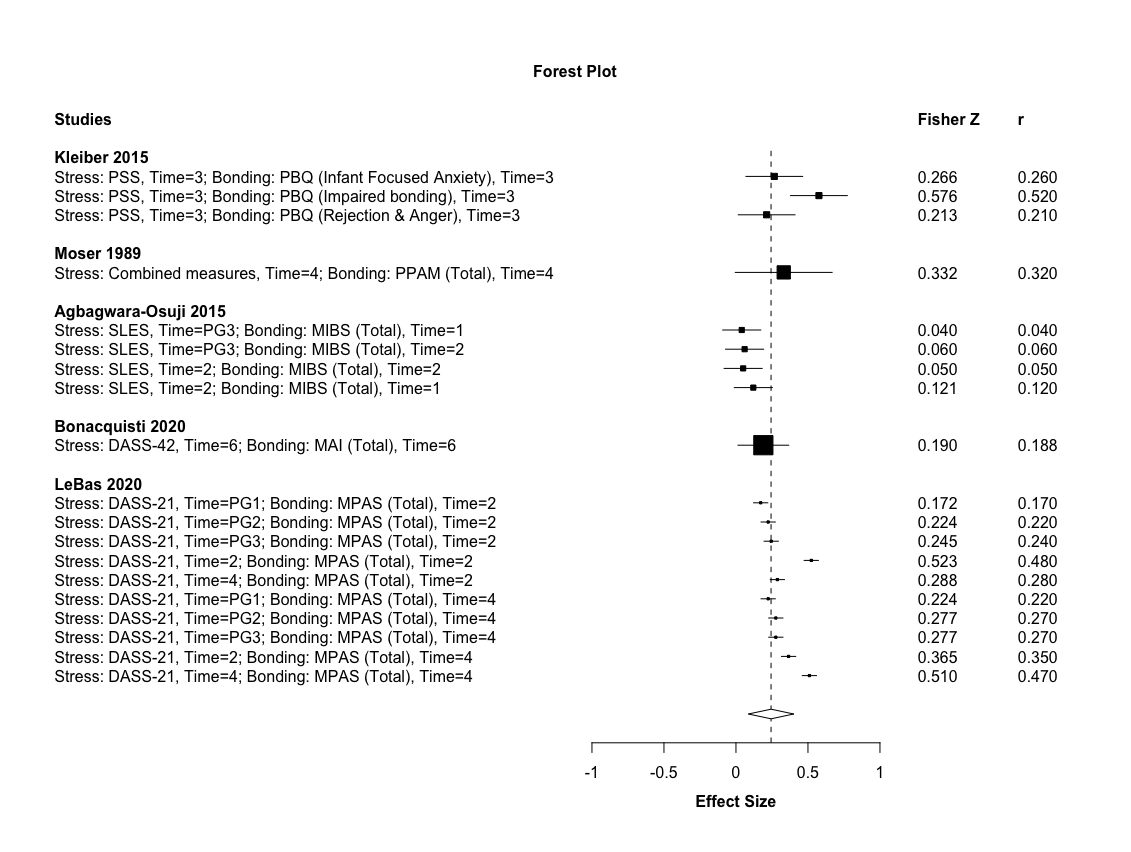


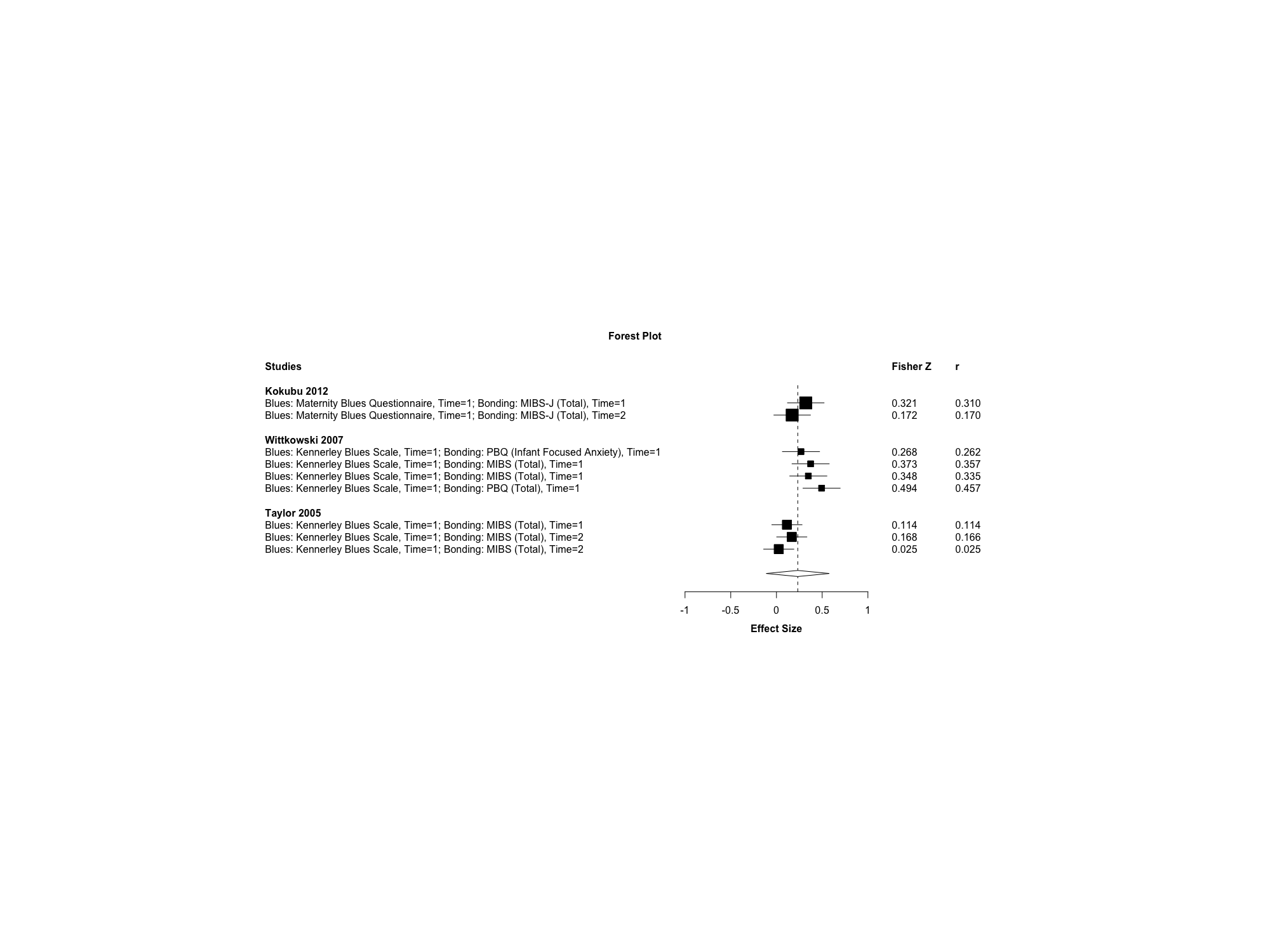
eFigure 2. Forest plot of all associations between postpartum blues and poorer mother-infant bonding.

**Forest plots for cross-sectional meta-analyses.**

The following series of forest plots relates to the meta-analyses of cross-sectional postpartum effects presented in Table 2 of the manuscript.


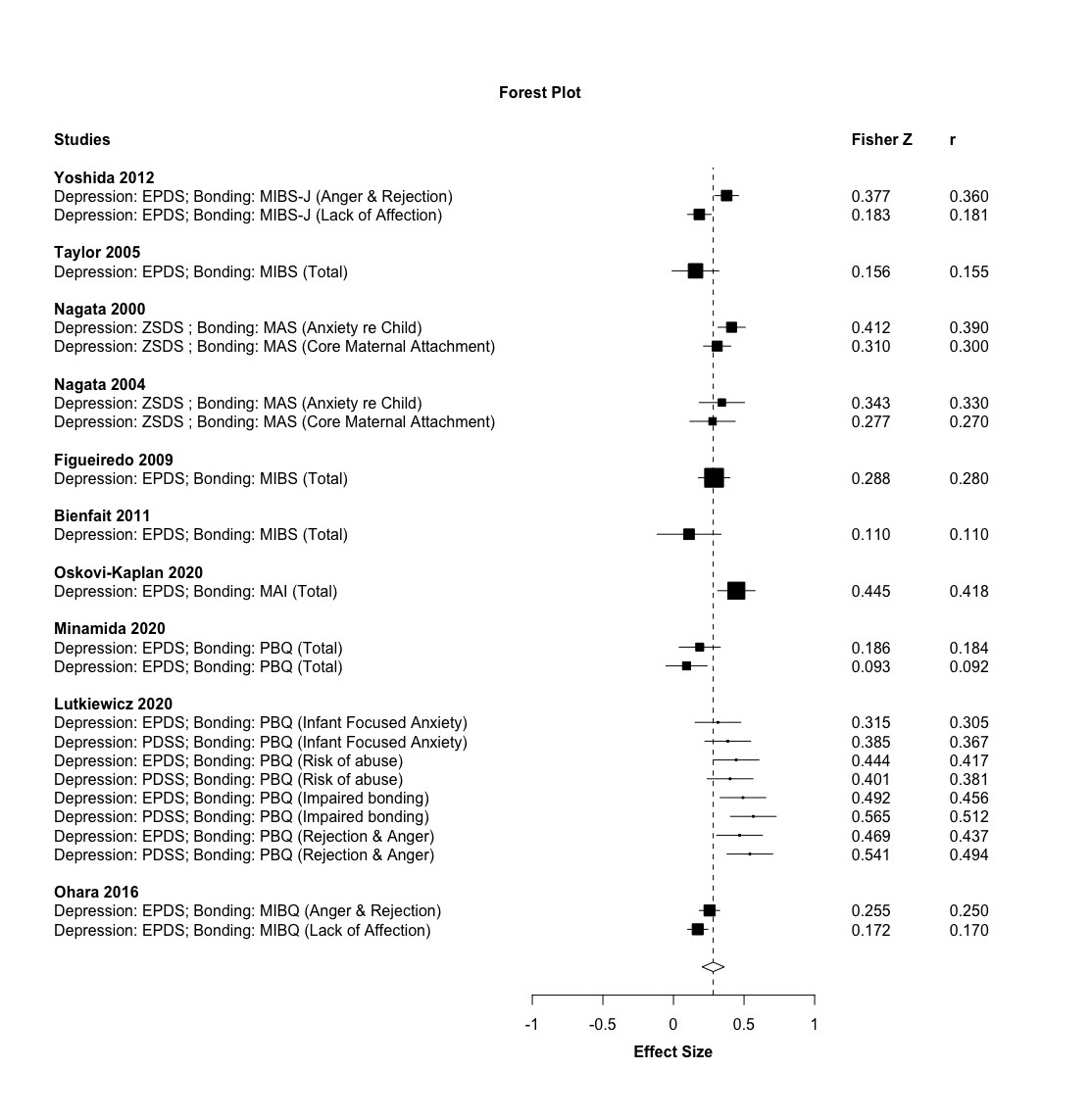
eFigure 3. Forest plot of cross-sectional associations between depression and poorer mother-infant bonding at Time 1 (birth to 1 week).


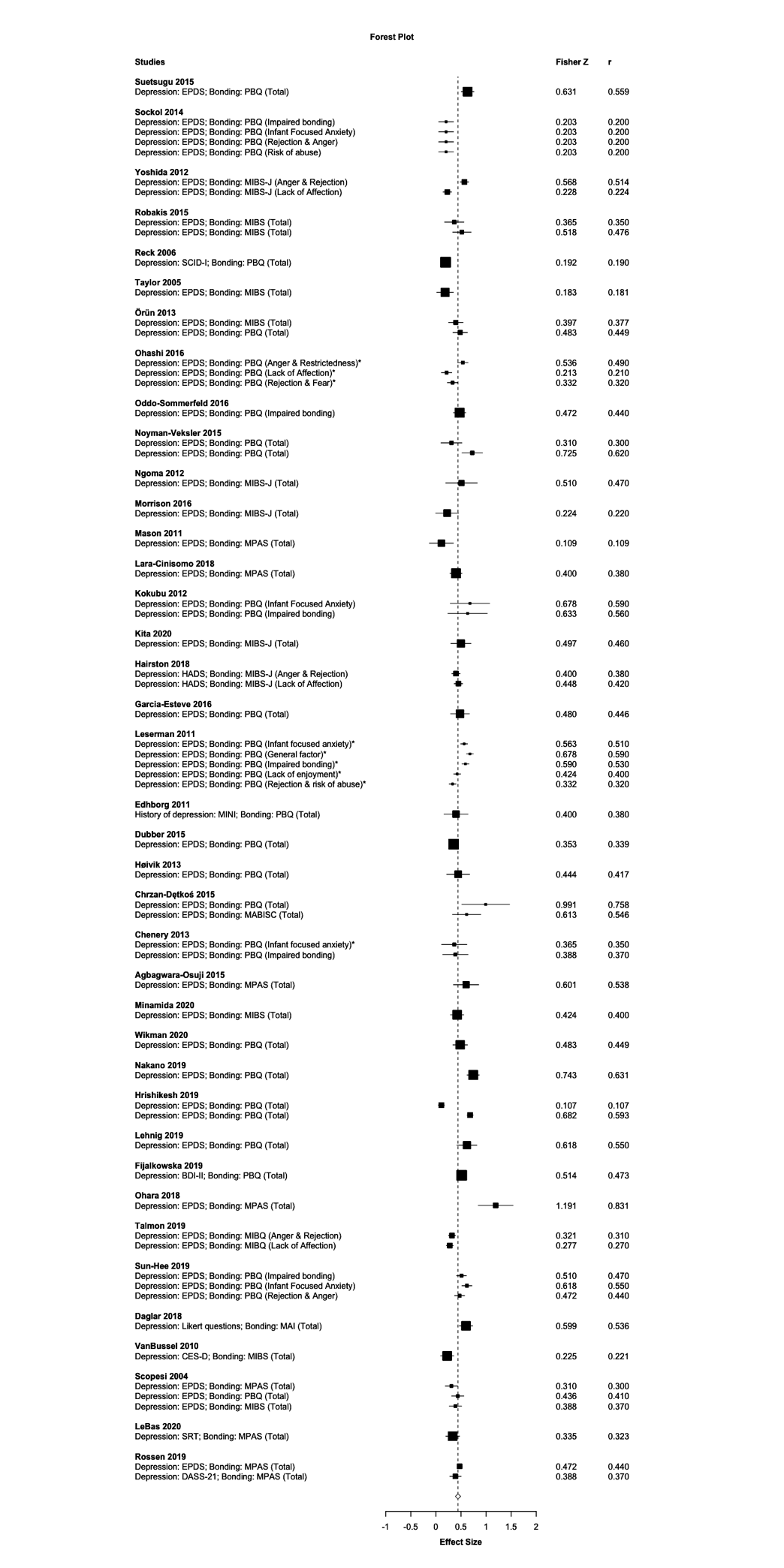
eFigure 4. Forest plot of cross-sectional associations between depression and poorer mother-infant bonding at Time 2 (>1 week to <3 months).

eFigure 5. Forest plot of cross-sectional associations between depression and poorer mother-infant bonding at Time 3 (3 months to <6 months).


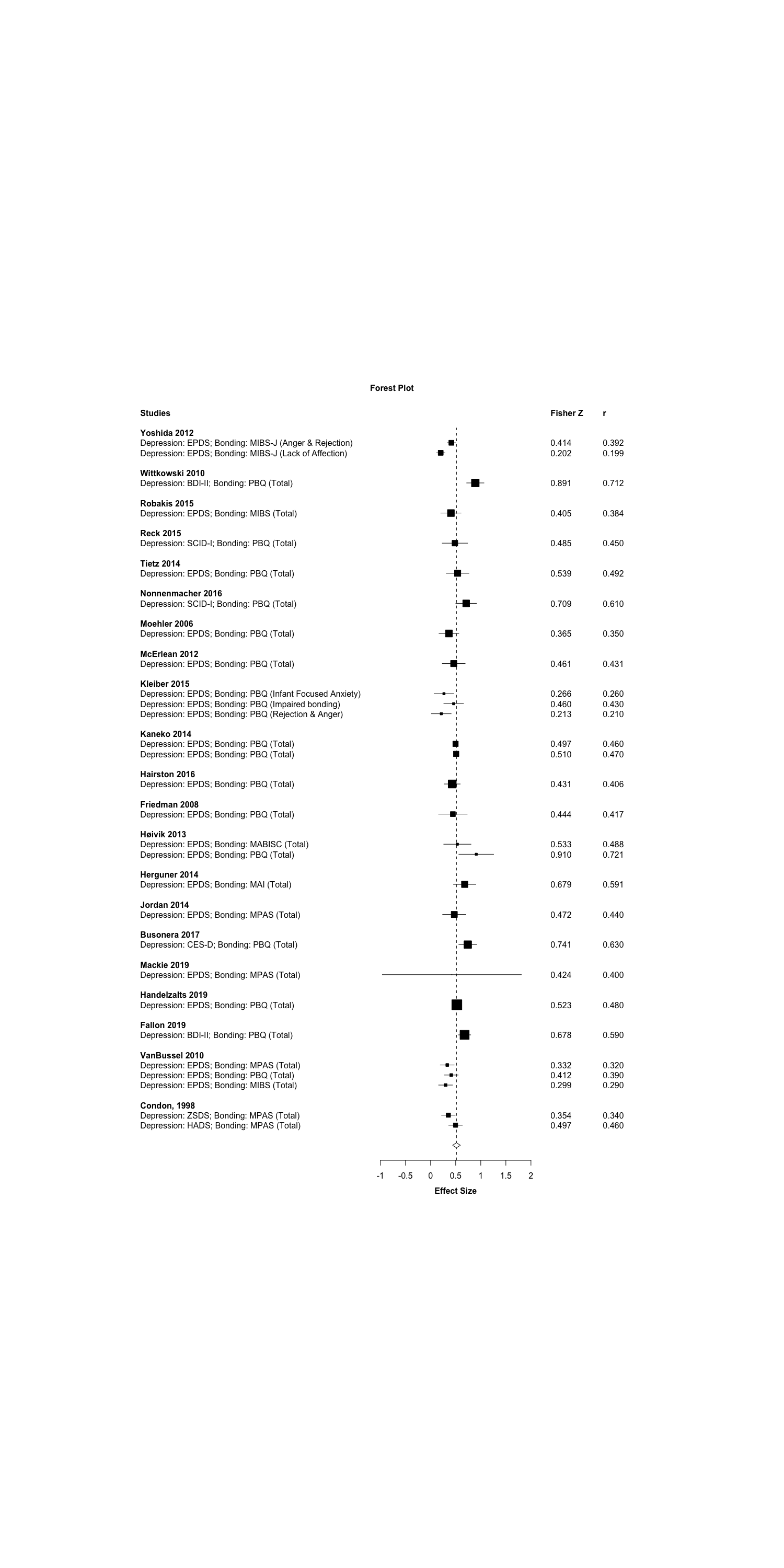


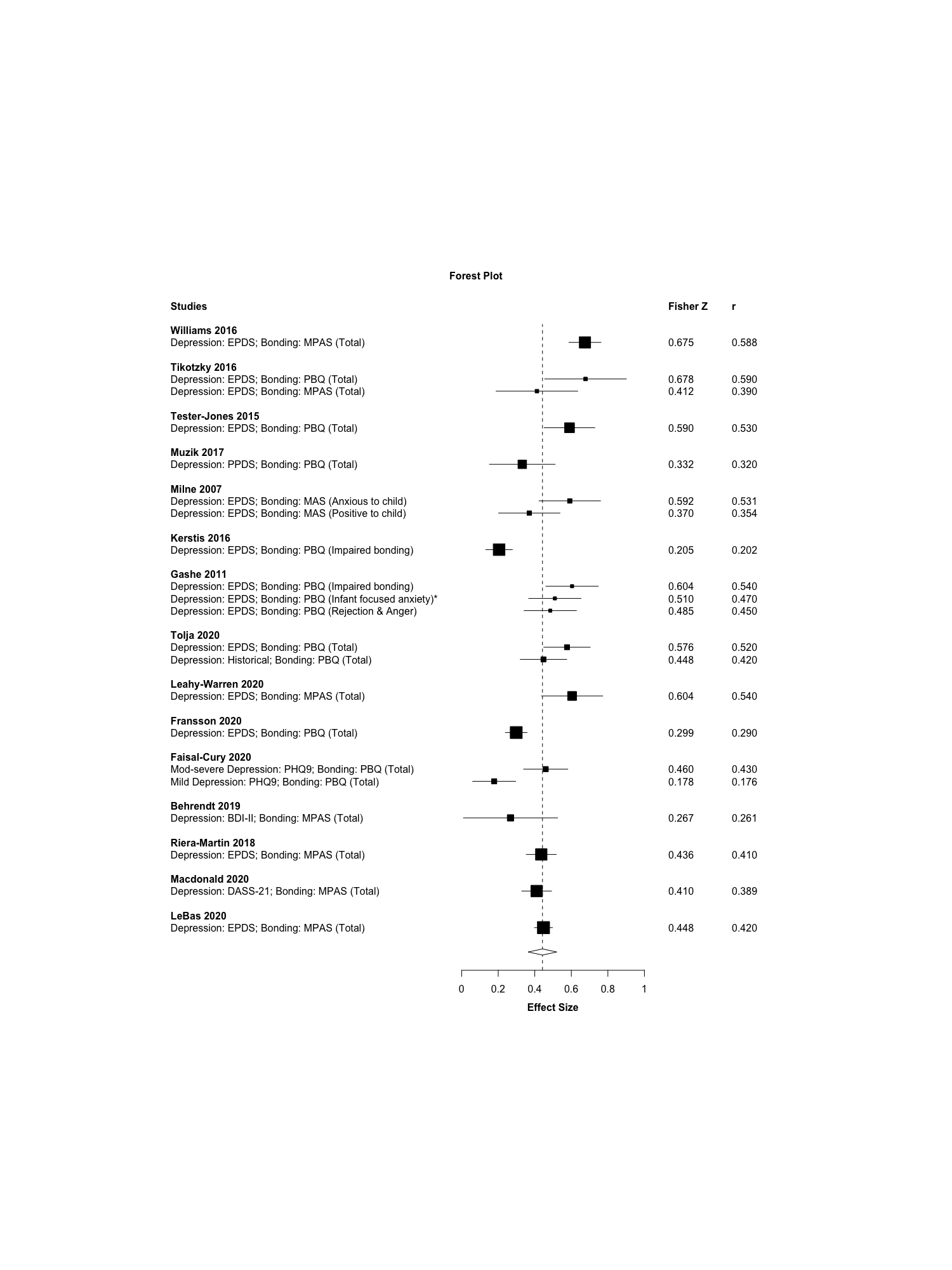
eFigure 6. Forest plot of cross-sectional associations between depression and poorer mother-infant bonding at Time 4 (6 months to 12 months).

eFigure 7. Forest plot of cross-sectional associations between anxiety and poorer mother-infant bonding at Time 2 (>1 week to <3 months).


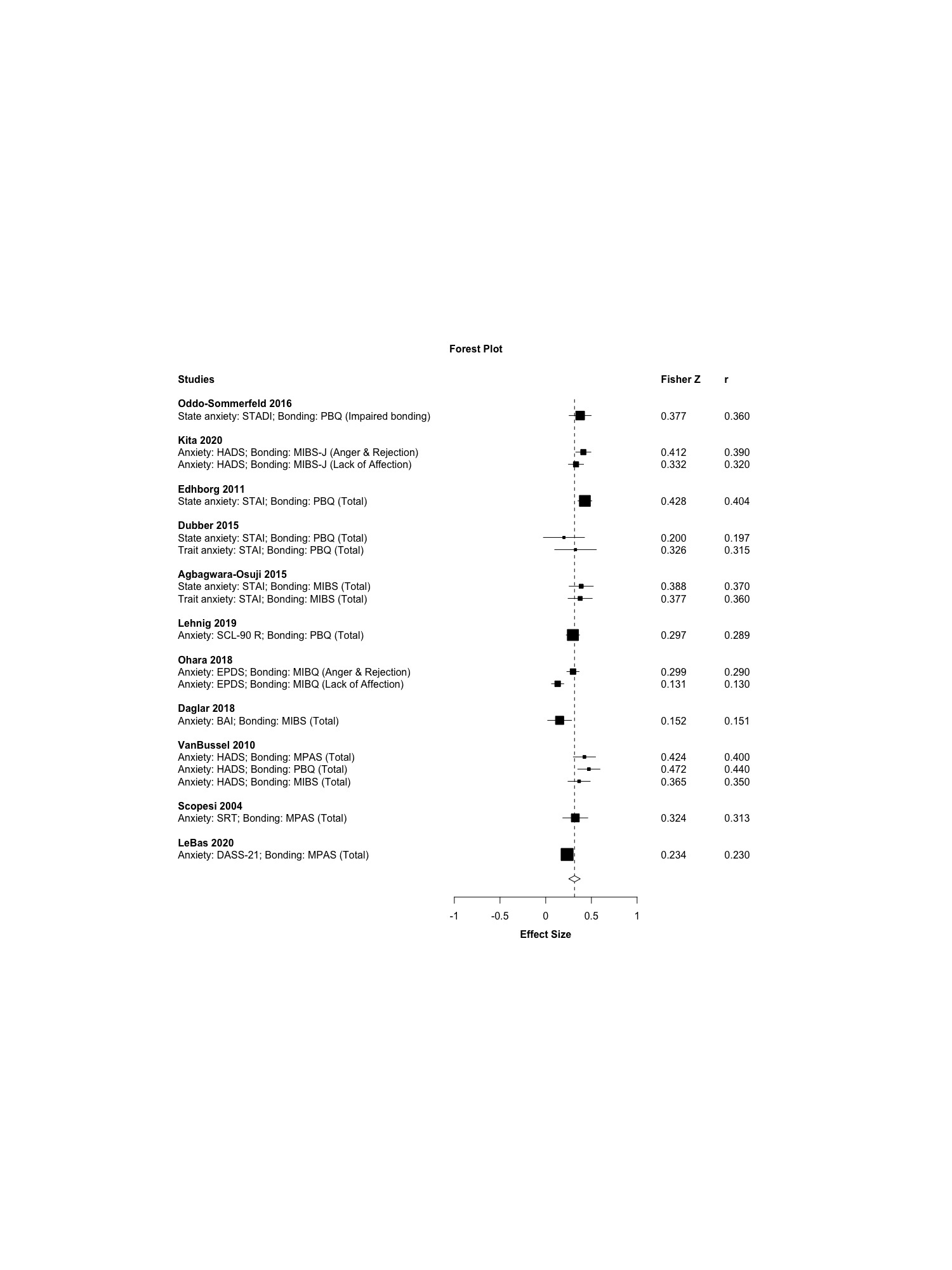


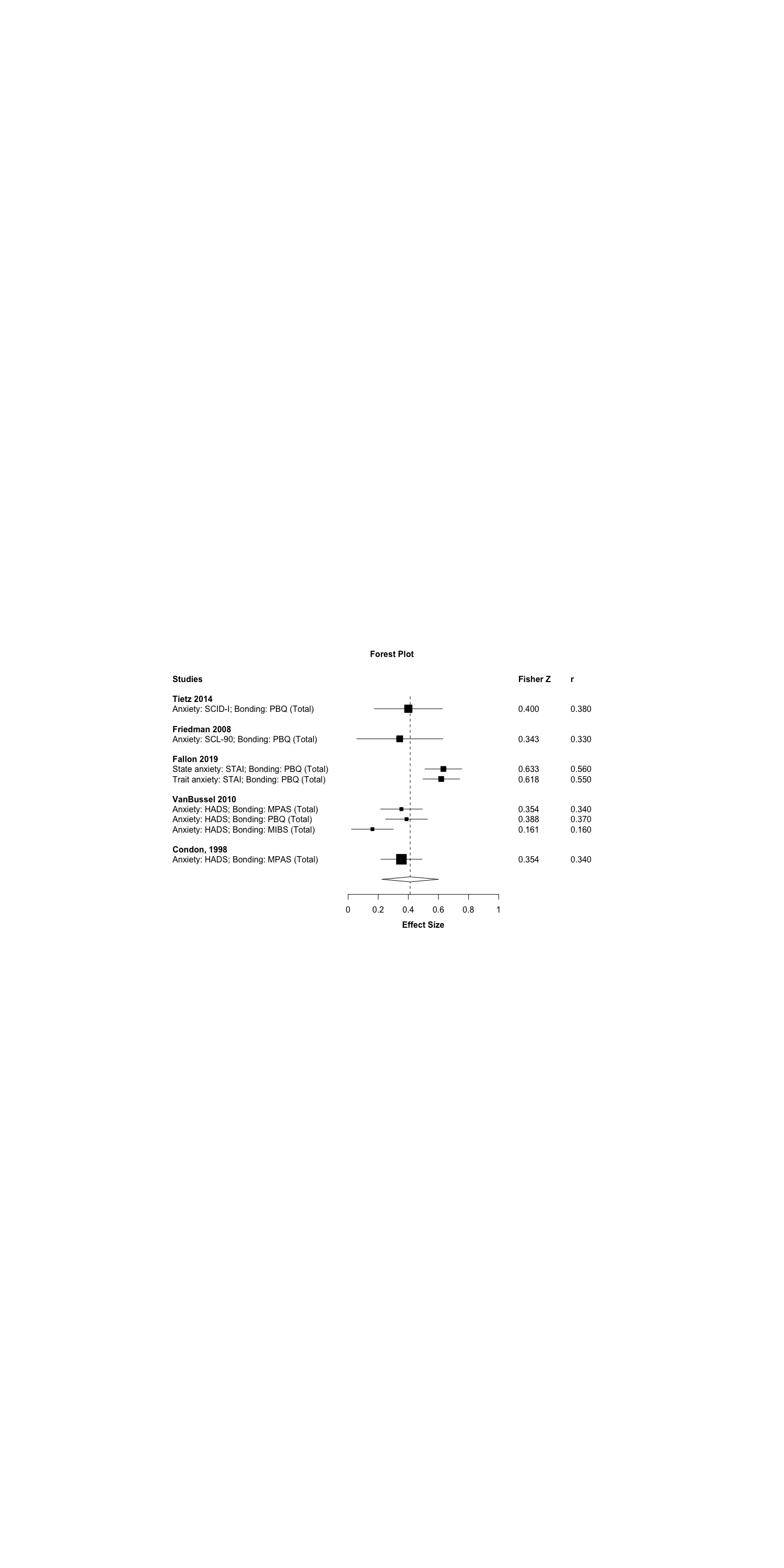
eFigure 8. Forest plot of cross-sectional associations between anxiety and poorer mother-infant bonding at Time 3 (3 months to <6months).


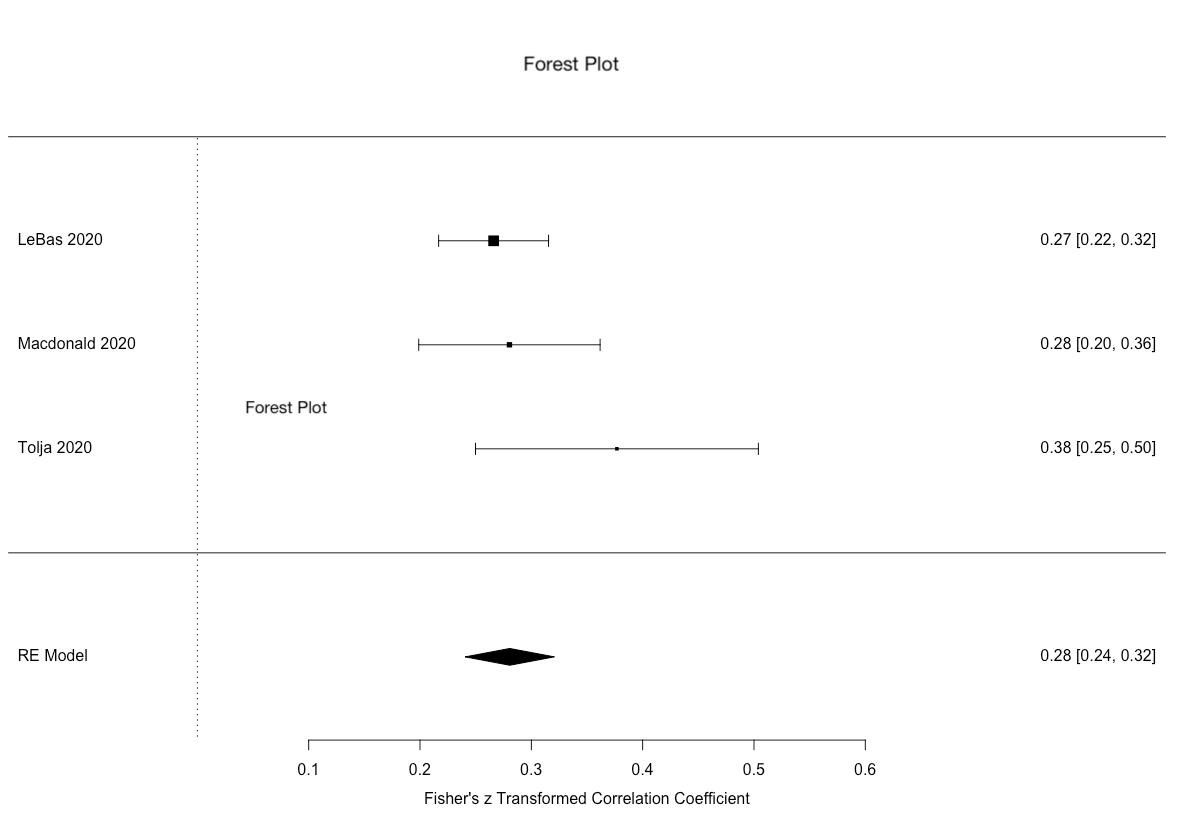
eFigure 9. Forest plot of cross-sectional associations between anxiety and poorer mother-infant bonding at Time 4 (6 months to 12 months).


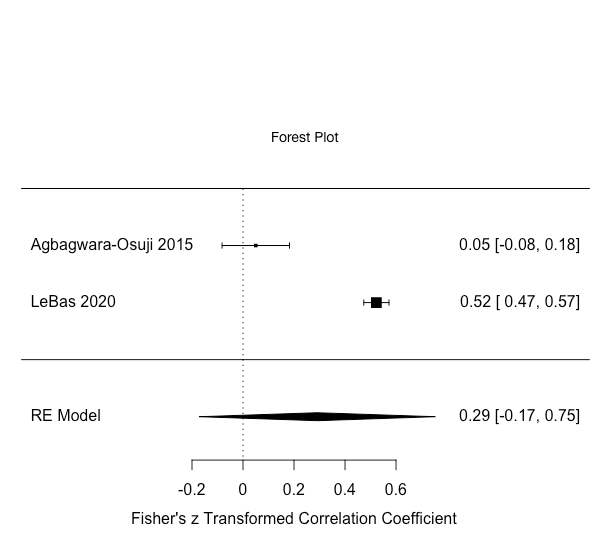
eFigure 10. Forest plot of cross-sectional associations between stress and poorer mother-infant bonding at Time 2 (>1 week to <3 months).


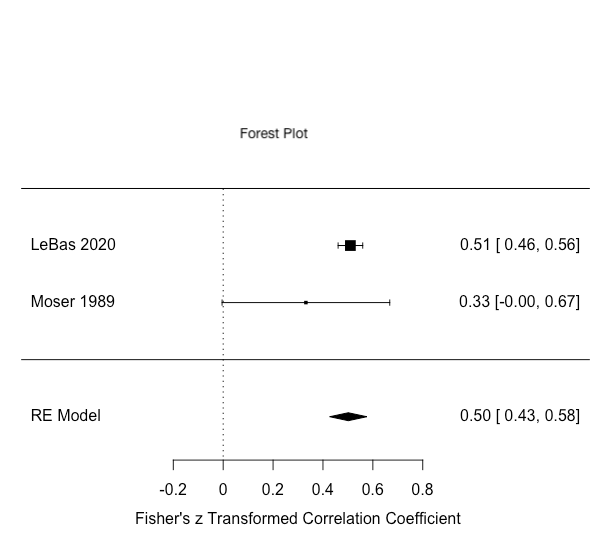
eFigure 11. Forest plot of cross-sectional associations between stress and poorer mother-infant bonding at Time 4 (6 months to 12 months).


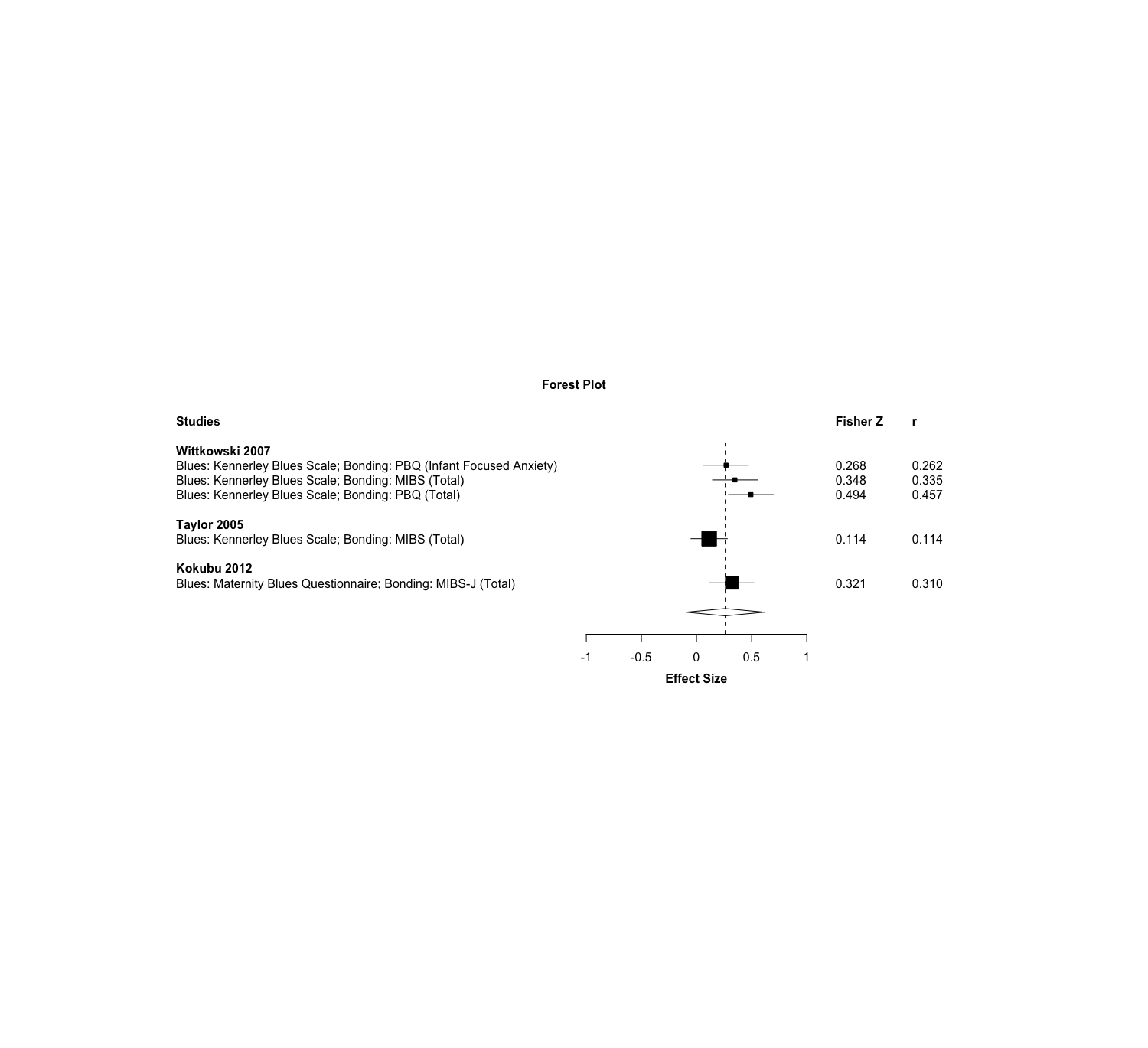
eFigure 12. Forest plot of cross-sectional associations between stress and poorer mother-infant bonding at Time 1 (birth to 1 week).

**Forest plots for longitudinal meta-analyses.**

The following series of forest plots relates to the meta-analyses presented in Table 3 of the manuscript.


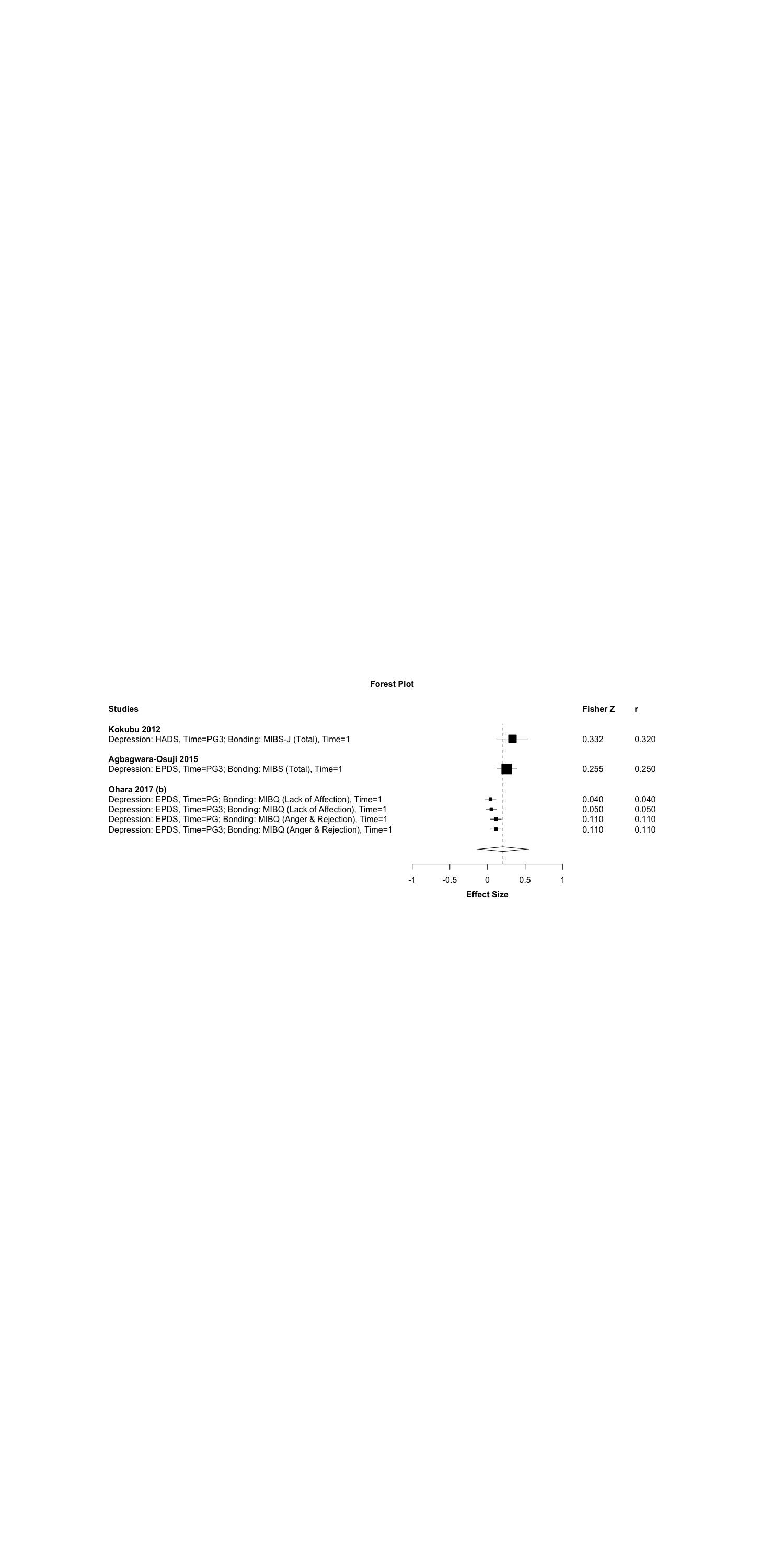
eFigure 13. Forest plot of longitudinal associations between depression in pregnancy and poorer mother-infant bonding at Time 1 (birth to 1 week).

eFigure 14. Forest plot of longitudinal associations between depression in pregnancy and poorer mother-infant bonding at Time 2 (>1 week to <3 months).


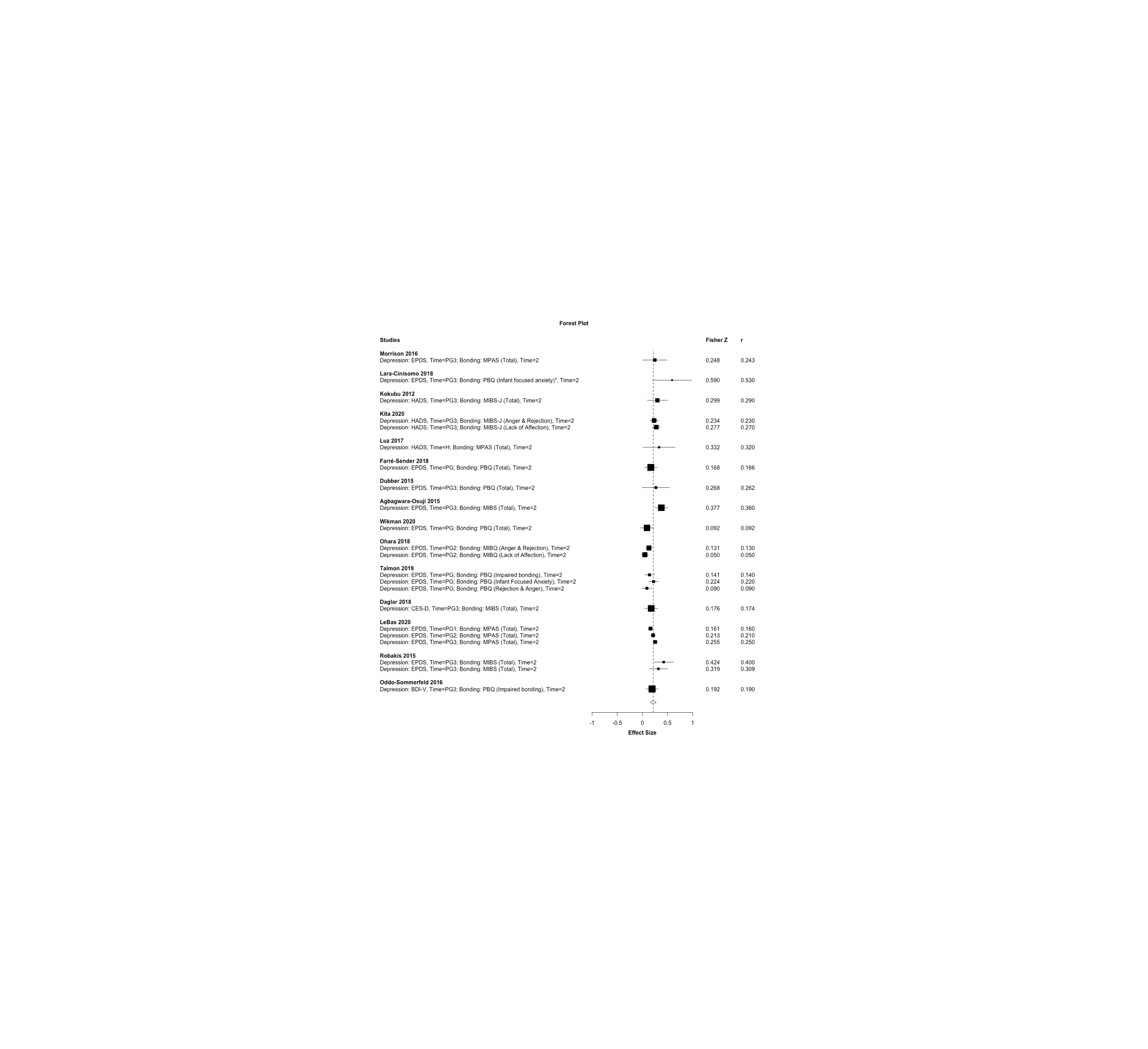


eFigure 15. Forest plot of longitudinal associations between depression in pregnancy and poorer mother-infant bonding at Time 3 (3 months to <6 months).


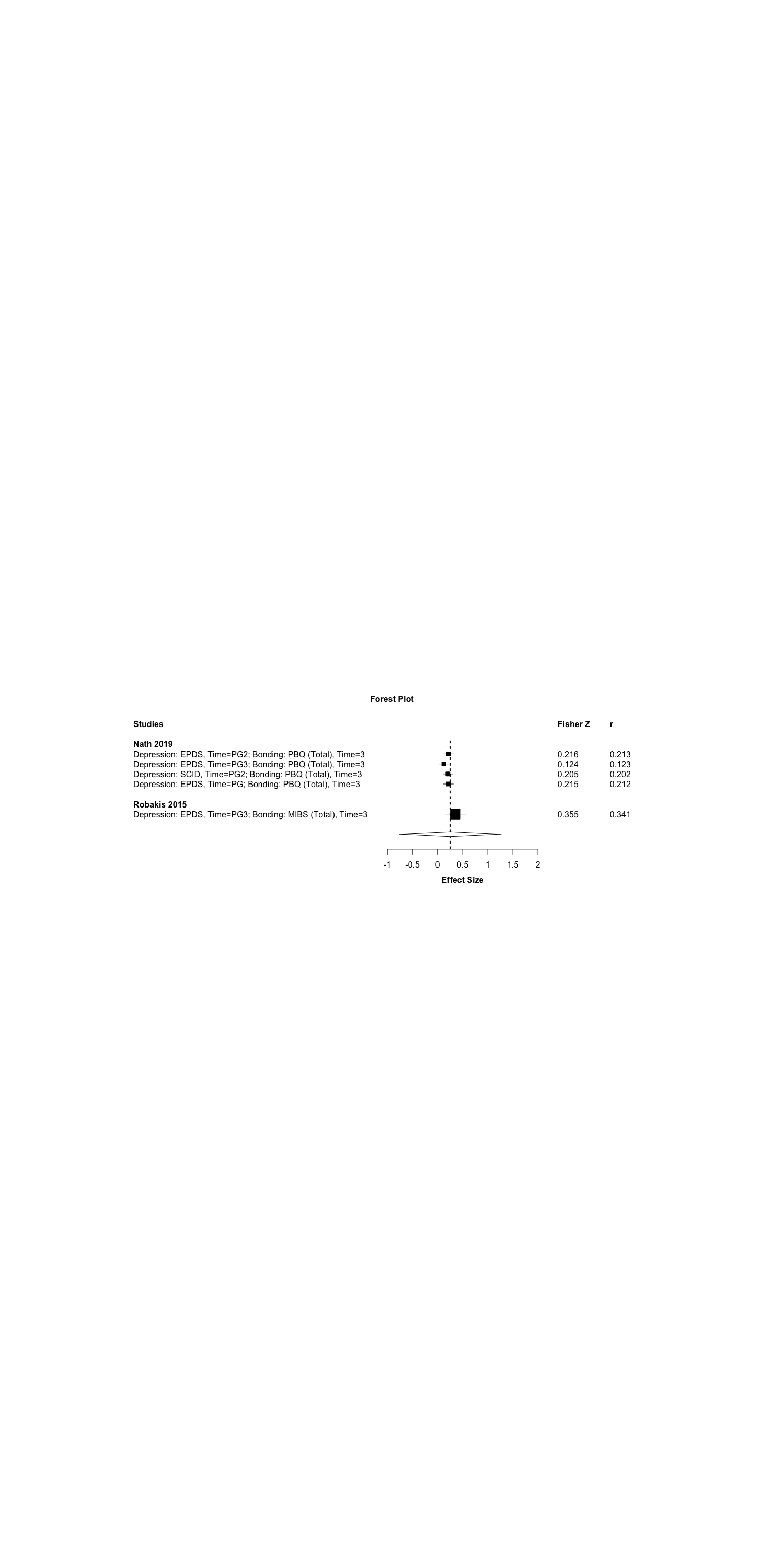


eFigure 16. Forest plot of longitudinal associations between depression in pregnancy and poorer mother-infant bonding at Time 4 (6 months to 12 months).


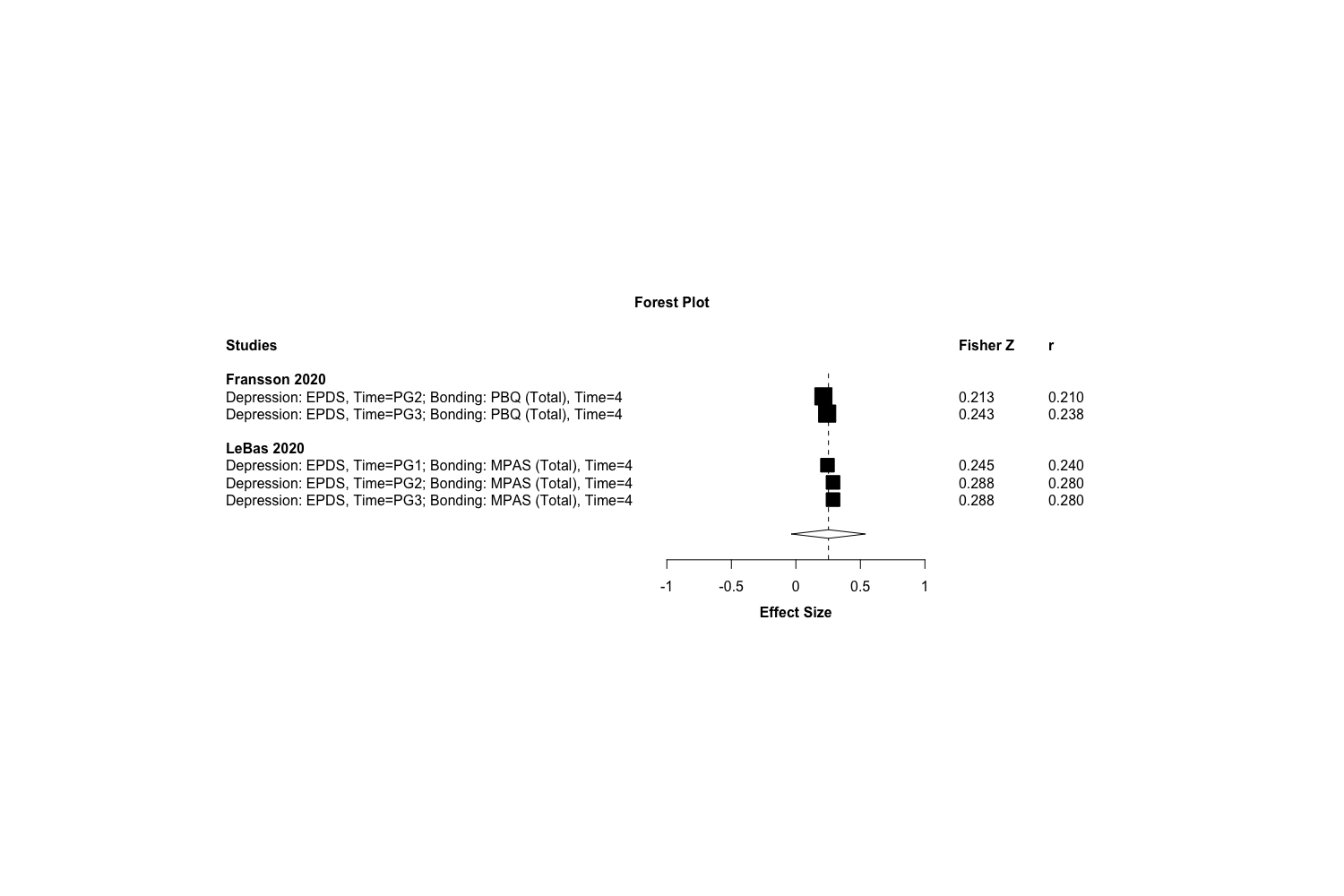


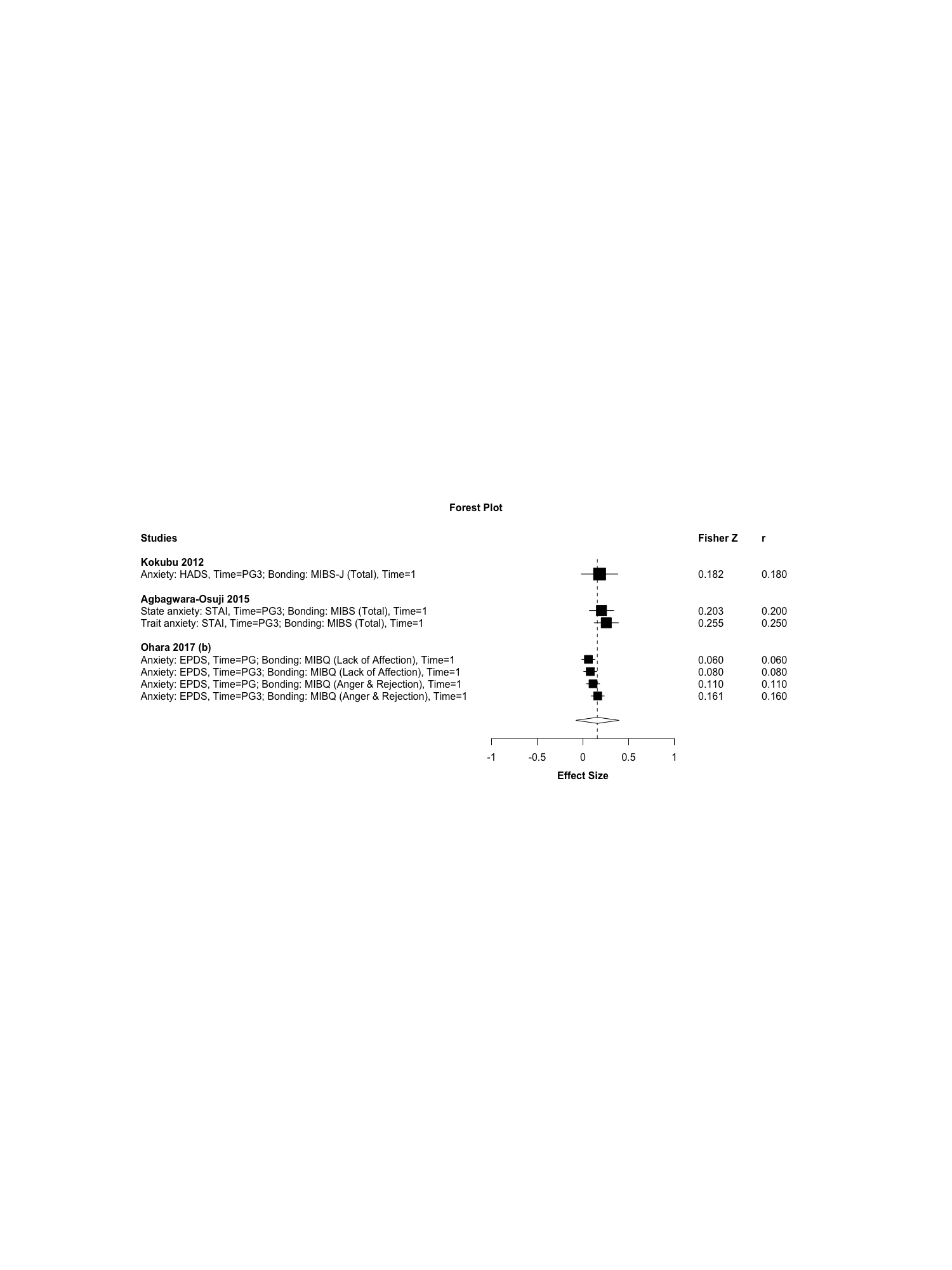
eFigure 17. Forest plot of longitudinal associations between anxiety in pregnancy and poorer mother-infant bonding at Time 1 (birth to 1 week).


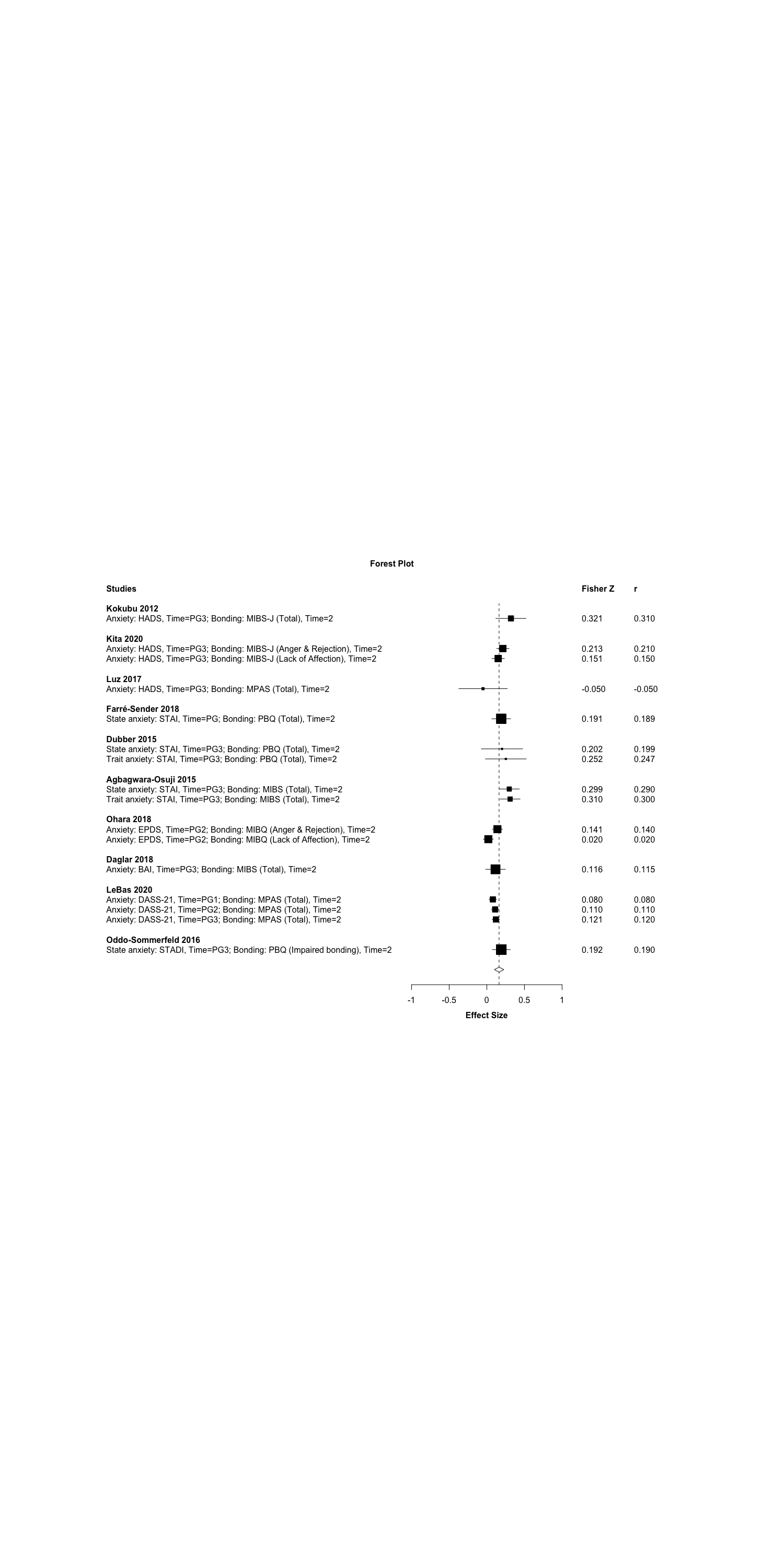
eFigure 18. Forest plot of longitudinal associations between anxiety in pregnancy and poorer mother-infant bonding at Time 2 (>1 week to <3 months).


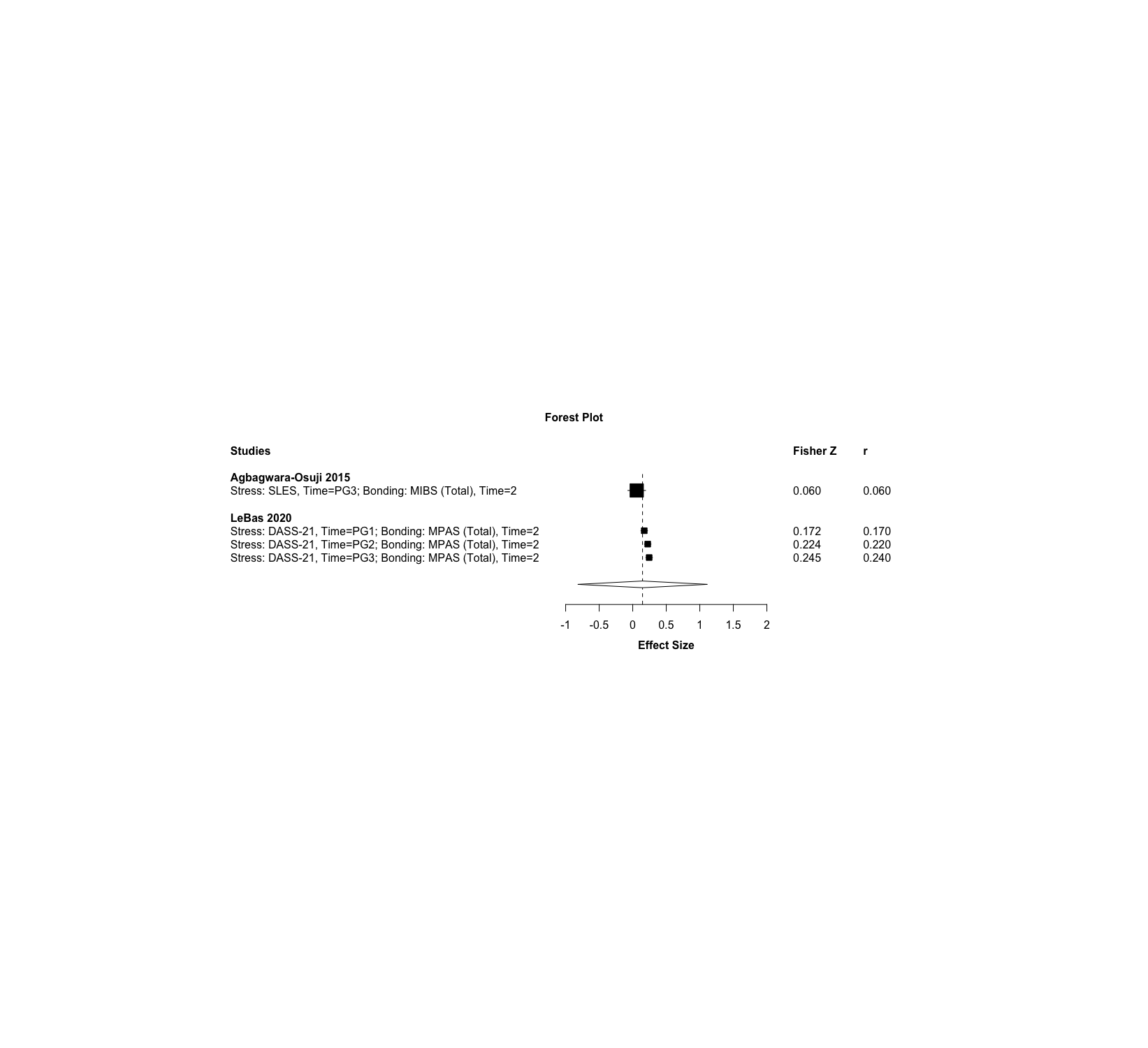
eFigure 19. Forest plot of longitudinal associations between stress in pregnancy and poorer mother-infant bonding at Time 2 (>1 week to <3 months).

eTable 9. Risk of bias of included studies.

| Citation | Clear research question | Study population clearly defined | Participation rate >50% | Attrition rate <20%? | Sample size justification | Exposure measured prior to outcome? | Continuous Psychological distress variable? | Continuous bonding variable? | Psychological distress measures  valid and reliable? | Bonding measures valid and reliable? |
| --- | --- | --- | --- | --- | --- | --- | --- | --- | --- | --- |
| Agbagwara-Osuji (2015)* | Y | Y | Y | N | Y | Y | Y | Y | Y | Y |
| Badr et al. (2018) | Y | Y | Y | Y | Y | Y | N | N | Y/N | Y |
| Behrendt et al. (2016) | Y | N | NR | NR | N | N | Y/N | Y | Y | Y |
| Behrendt et al. ( 2019) | Y | N | NR | NR | N | N | Y | Y | Y | Y |
| Bicking Kinsey et al. (2014) | Y | Y | NR | Y | N | N | Y | Y | N | N |
| Bienfait et al. (2011) | Y | Y | Y | NA | N | N | Y | Y | Y | Y |
| Bonacquisti et al. (2020) | Y | Y | Y | N | N | N | Y | Y | Y | Y |
| Borji et al. (2018) | Y | Y | NR | NA | Y | N | Y | Y | Y | Y |
| Borschmann et al. (2019) | Y | Y | Y | N | N | Y | Y | Y | Y | Y |
| Busonera et al. (2017) | Y | Y | NR | NA | N | N | Y | Y | Y | Y |
| Carter (2018)* | Y | N | N | N | N | N | Y | Y | Y | Y |
| Chenery (2013)* | Y | Y | Y | NA | Y | N | Y | Y | Y | N |
| Choi et al. (2017) | Y | N** | N | NR | Y | Y | Y | Y | Y | Y |
| Chrzan-Dętkoś et al. (2015) | Y | N | NR | NR | N | N | Y | Y | Y | Y |
| Condon & Corkindale (1998) | Y | N | NR | Y | N | N | Y | Y | Y | Y |
| Cuijlits et al. (2016) | Y | Y | Y | NR | N | N | Y | Y | Y | Y |
| Cuijlits et al. (2019) | Y | Y** | Y | N | Y | N | Y | N | Y | Y |
| Daglar & Nur, (2018) | Y | Y | Y | NR | N | Y | Y | Y | Y | Y |
| Damato (2004) | Y | N | NR | N | Y | N | Y | Y | Y | Y |
| Dubber et al. (2015) | Y | Y | NR | NR | Y | Y | Y | Y | Y | Y |
| Edhborg et al. (2005) | Y | Y | Y | N | N | Y | Y | Y | Y | Y |
| Edhborg et al. (2011) | Y | N | NR | Y | Y | N | Y | Y | Y | Y |
| Edhborg et al. (2013) | Y | N | Y | Y | Y | N | N | Y | Y | Y |
| Faisal-Cury et al. (2020) | Y | Y | Y | NA | N | N | N | N | Y | Y |
| Fallon et al. (2019) | Y | N | Y | N | N | N | Y | Y | Y | Y |
| Farré-Sender et al. (2018) | Y | N | NR | N | Y | Y | Y | N | Y | Y |
| Figueiredo et al. (2009) | Y | Y | Y | NA | N | N | N | N | Y | Y |
| Figueiredo & Costa (2009) | Y | N | Y | NR | N | Y | Y | Y | Y | Y |
| Fijalkowska & Bielawska-Batorowicz, (2019) | Y | N | NR | N | N | N | Y | Y | Y | Y |
| Fleming et al. (1988) | Y | N | NR | Y | N | Y | Y | Y | NR | Y |
| Fransson et al. (2020) | Y | Y | NR | N | N | Y | Y | Y | Y | Y |
| Friedman (2008)* | Y | N | Y | NA | N | N | Y | Y | Y | Y |
| Garcia-Esteve et al. (2016) | Y | N | NR | NA | N | N | Y | Y | Y | Y |
| Gashe (2011)* | Y | N | NR | NR | Y | N | Y | Y | Y | Y |
| Hairston et al. (2011) | Y | Y** | NR | NR | N | N | Y | Y | Y | N |
| Hairston et al. (2016) | Y | N | NR | NA | N | N | Y | Y | Y | Y |
| Hairston et al. (2018) | Y | N | NR | NA | N | N | Y | Y | Y | N |
| Hairston et al. (2019) | Y | N | NR | N | N | N | Y | Y | Y | Y |
| Handelzalts et al. (2019) | Y | N** | NR | NA | N | N | Y | Y | Y | Y |
| Herguner et al. (2014) | Y | N | NR | NA | N | N | Y | Y | Y | Y |
| Hiroko et al. (2020)* | N | N | NR | NA | N | N | NR | NR | Y | Y |
| Høivik et al. (2013) | Y | Y | Y | N | N | Y | Y | Y | Y | Y |
| Hrishikesh (2019)* | Y | N | NR | NR | N | N | Y | Y | Y | Y |
| Jones et al. (2011) | Y | N | NR | NA | Y | NR | Y | Y | Y | Y |
| Jordan et al. (2014) | Y | N | Y | NA | N | N | Y | Y | Y | Y |
| Kaneko & Honjo (2014) | Y | Y | NR | NA | N | N | N | Y | Y | Y |
| Kasamatsu et al. (2020) | Y | Y** | Y | NR | N | Y | N | Y | Y | Y |
| Kerstis et al. (2016) | Y | Y | Y | NR | N | Y | Y | N | Y | Y |
| Kita et al. (2016) | Y | Y | Y | N | N | Y | Y | Y | Y | Y |
| Kita et al. (2020) | Y | Y | Y | N | N | Y | Y | Y | Y | Y |
| Kleiber (2015)* | Y | N | Y | NA | N | N | Y | Y | Y | N |
| Kokubu et al. (2012) | Y | Y | Y | Y | N | Y | Y | Y | Y | Y |
| Lara-Cinisomo et al. (2018) | Y | Y | NR | Y | N | Y | Y | Y | Y | Y |
| Leahy-Warren et al. (2020) | Y | Y | NR | NA | Y | N | Y | Y | Y | Y |
| Le Bas et al. (2020) | Y | Y | NR | NR | N | Y | Y | Y | Y | Y |
| Lehnig et al. (2019) | Y | Y | NR | NA | N | N | N | Y | Y | Y |
| Leserman et al. (2011)* | Y | N | NR | NR | N | N | N | NR | Y | Y |
| Loh & Vostanis (2004) | Y | Y | NR | NA | N | N | Y | N | Y | Y |
| Lutkiewicz et al. (2020) | Y | Y** | NR | NA | N | N | Y | Y | Y | Y |
| Luz et al. (2017) | Y | Y | N | N | N | Y | Y | Y | Y | Y |
| Macdonald et al. (2020) | Y | Y | Y | Y | N | Y | Y | Y | Y | Y |
| Mackie et al. (2019) | Y | Y | NR | N | Y | N | Y | Y | Y | Y |
| Martini et al. (2020) | Y | Y | Y | Y | N | Y | N | N | Y | Y |
| Mason et al. (2011) | Y | N | NR | N | Y | N | N | Y | Y | Y |
| Matthies et al. (2020) | Y | Y | NR | N | N | N | Y | Y | Y | Y |
| McErlean (2012)* | Y | N | NR | Y | N | N | Y | Y | Y | Y |
| Mercer & Ferketich (1990) | Y | N | Y | N | N | N | Y | Y | Y | Y |
| Mercer & Ferketich (1994) | Y | N | NR | N | N | N | Y | Y | Y | Y |
| Milne et al. (2007) | Y | N | NR | NA | N | N | Y | Y | Y | Y |
| Minamida et al. (2020) | Y | Y | Y | N | Y | N | N | Y | Y | Y |
| Moehler et al. (2006) | Y | N | NR | Y | Y | Y | Y | Y | Y | Y |
| Morrison (2016)* | Y | N | NR | NR | Y | Y | Y | Y | Y | Y |
| Moser (1989)** | Y | N | NR | Y | N | Y | Y | Y | Y/N | Y |
| Müller et al. (2013) | Y | N | NR | N | Y | Y | Y | Y | Y | Y |
| Muzik et al. (2013) | Y | Y** | NR | N | N | N | Y | Y | Y | N |
| Muzik et al. (2017) | Y | Y** | NR | NA | N | N | Y | Y | Y | N |
| Myers (2017)* | Y | N | NR | N | N | Y | Y/N | Y/N | Y/N | Y |
| Nagata et al. (2000) | Y | Y | Y | NA | N | N | Y | Y | Y | Y |
| Nagata et al. (2004) | Y | Y | Y | NA | N | N | Y | Y | Y | Y |
| Nakano et al. (2019) | Y | Y | NR | NR | N | N | N | N | Y | Y |
| Nakash et al. (2016) | Y | Y | NR | NA | N | N | Y | Y | Y | Y |
| Nath et al. (2019) | Y | Y | NR | Y | N | Y | Y | Y | Y | Y |
| Ngoma et al. (2012) | Y | Y | Y | NA | N | N | Y | Y | Y | Y |
| Nolvi et al. (2016) | Y | Y** | NR | N | N | Y | Y | Y | Y | N |
| Nonnenmacher et al. (2016) | Y | Y | NR | Y | N | N | N | Y | Y | Y |
| Noyman-Veksler et al. (2015) | Y | N | NR | N | N | Y | Y | Y | Y | Y |
| O'Higgins et al. (2013) | Y | N** | N | N | N | Y | N | Y | Y | Y |
| Oddo-Sommerfeld et al. (2016) | Y | N** | N | Y | N | Y | Y | Y | Y/N | Y |
| Ohara et al. (2016) | Y | Y | NR | N | N | N | Y | Y | Y | Y |
| Ohara et al. (2017a) | Y | Y | NR | N | N | Y | Y | Y | Y | Y |
| Ohara et al. (2017b) | Y | Y | NR | N | N | Y | Y | Y | Y | Y |
| Ohara et al. (2018) | Y | Y | NR | N | N | Y | Y | Y | Y | Y |
| Ohashi et al. (2016) | Y | Y | N | NA | N | N | Y | Y | Y | Y |
| Ohoka et al. (2014) | Y | Y** | NR | N | N | N | Y | Y | Y | Y |
| Olsson et al. (2020) | Y | Y | Y | NR | N | Y | Y | Y | Y | Y |
| Örün et al. (2013) | Y | Y** | NR | NR | N | Y | Y | Y | Y | Y |
| Oskovi-Kaplan et al. (2020) | Y | Y | N | NA | Y | N | N | Y | Y | Y |
| Parfitt et al. (2014) | Y | N | NR | N | N | Y | Y | Y | Y | Y |
| Pearson et al. (2013) | Y | Y | NR | N | Y | N | Y | Y | Y | Y |
| Petri et al. (2017) | Y | Y | NR | N | N | Y | N | Y | N | Y |
| Rados et al. (2020) | Y | N | NR | NA | N | N | Y | Y | Y | Y |
| Reck et al. (2006) | Y | Y | NR | NR | N | N | N | Y | Y | Y |
| Reck et al. (2015) | Y | Y | NR | N | Y | N | Y | Y | Y | Y |
| Riera-Martin et al. (2018) | Y | N | Y | N | N | N | Y | Y | Y | Y |
| Robakis et al. (2015) | Y | Y | NR | Y | N | Y | Y | Y | Y | Y |
| Rossen et al. (2016) | Y | Y | NR | NR | N | Y | Y | Y | Y | Y |
| Rossen et al. (2019) | Y | Y** | NR | NR | N | Y | Y | Y | Y | Y |
| Sawyer Cohen (2011)* | Y | N | Y | N | N | Y | Y | Y | Y | Y |
| Schmidt et al. (2017) | Y | N | NR | N | N | Y | Y | Y | Y | Y |
| Scopesi et al. (2004) | Y | N | NR | NA | N | N | Y | Y | Y | Y |
| Seng et al. (2013) | Y | Y | Y | N | N | Y | Y | Y | N | Y |
| Sockol et al. (2014) | Y | Y | Y | NA | N | N | Y | Y | Y | Y |
| Suetsugu et al. (2015) | Y | Y | NR | N | N | N | Y | Y | Y | Y |
| Suetsugu et al. (2020) | Y | Y | Y | N | N | N | Y | Y | Y | Y |
| Sun-Hee (2019) | Y | Y | NR | NA | Y | N | N | Y | N | Y |
| Talmon et al. (2019) | Y | N | NR | N | N | Y | Y | Y | Y | Y |
| Talmon et al. (2020) | Y | N | NR | N | N | Y | Y | Y | Y | Y |
| Taylor et al. (2005) | Y | N | NR | Y | N | Y | Y | Y | Y | Y |
| Tester-Jones et al. (2015) | Y | N | NR | NA | N | N | Y | Y | Y | Y |
| Tietz et al. (2014) | Y | Y | NR | NA | N | N | Y | Y | Y | Y |
| Tikotzky (2016) | Y | Y | NR | NA | N | N | Y | Y | Y | N |
| Tolja et al. (2020) | Y | Y | NR | NA | N | N | Y | Y | Y/N | Y |
| Tsuchida et al. (2019) | Y | Y** | Y | NR | N | N | Y | Y | Y | Y |
| VanBussel et al. (2010) | Y | Y | NR | N | N | N | Y | Y | Y | Y |
| Vengadavaradan et al. (2019) | Y | N | NR | NR | N | N | Y | Y | Y | Y |
| Vreeswijk et al. (2011)* | Y | N | NR | NR | N | Y | Y | Y | Y | Y |
| Wikman et al. (2020) | Y | Y | N | N | N | Y | N | N | Y/N | Y |
| Williams et al. (2016) | Y | Y | NR | NA | Y | Y | Y | Y | Y | Y |
| Wittkowski et al. (2007) | Y | N | NR | NR | N | N | Y | Y | Y | Y |
| Wittkowski et al. (2010) | Y | N | NR | NA | N | N | Y | Y | Y | Y |
| Yoshida et al. (2012) | Y | Y | Y | N | N | N | Y | Y | Y | Y |
| Zeitlin et al. (1999) | Y | N | NR | NA | N | N | Y | Y | Y | Y |
| Zhang et al. (2017) | Y | Y | NR | NR | N | N | Y | Y | Y | Y |

*Note: * = grey literature; ** = this data was retrieved from the cohort profile paper of the study; Y = Yes; N = No; NR = Not reported; NA = Not applicable.* Assessment criteria adapted from the NHLBI Quality Assessment Tool for Observational Cohort and Cross-Sectional Studies^.^[[1]](#footnote-1)^^

eTable 10. Study risk of bias assessment criteria.

| Item | Criteria | Option |
| --- | --- | --- |
| 1. Clear research question | Was the research question or objective in this paper clearly stated? | Y/N |
| 2. Study population clearly defined | Did the study describe  a) the group of people from which the study participants were recruited  b) the location recruited from AND  c) the date/time period of recruitment AND d) defined eligibility and exclusion criteria? | Y/N *All elements of this criteria must be met in order to receive a Yes on this criteria. |
| 3. Participation rate >50% | Was the participation rate of eligible persons at least 50%? | Y/N |
| 4. Attrition rate <20%? | Was loss to follow-up after baseline 20% or less? | Y/N |
| 5. Sample size justification | Was a sample size justification, power description, or variance and effect estimates provided? | Y/N |
| 6. Exposure measured prior to outcome? | For the analyses included in this review, were the exposure(s) of interest measured prior to the outcome(s) being measured? | Y = at least one included analysis was longitudinal; N = all included analyses were cross-sectional. |
| 7. Continuous psychological distress variable? | For psychological distress variables that can vary in amount or level, did the study examine different levels of the exposure as related to the outcome (e.g., multiple categories of exposure, or exposure measured as continuous variable)? | Y = continuous or multi-level variable used in included analyses N = dichotomous categorical variable used in included analyses Y/N = both continuous and categorical variables used in included analyses |
| 8. Continuous bonding variable? | For bonding variables that can vary in amount or level, did the study examine different levels of the exposure as related to the outcome (e.g., multiple categories of exposure, or exposure measured as continuous variable)? | Y = continuous or multi-level variable used in analysis N = dichotomous categorical variable used in analysis Y/N = both continuous and categorical variables used in analysis |
| 9. Psychological distress assessment measures valid and reliable? | Were the psychological distress measures clearly defined, valid, reliable, and implemented consistently across all study participants? | Y = all included measures met criteria N = included measures did not meet criteria Y/N = at least one included measure met criteria and at least one included measure did not meet criteria |
| 10. Bonding assessment measures valid and reliable?* | Were the self-report bonding measures clearly defined, valid, reliable, and implemented consistently across all study participants? | Y/N *Where a subscale was dropped (eg, PBQ risk of abuse) and a total score still calculated, this received "No" on this criteria. |

**Publication bias assessment of included studies.**

**Egger’s test of asymmetry**

In order to conduct Egger’s test, we first aggregated non-independent effects and grouped by psychological distress domain. An extreme case was identified in the depression group due to this study having a very small sample size (n = 5)^[[2]](#footnote-2)^. Therefore, Egger’s test was conducted for depression both with, and without, the outlier included. The results of the Egger’s tests of asymmetry were not significant for any psychological distress domain, and thus did not provide evidence for presence of publication bias.

eTable 11. Egger's test of asymmetry for publication bias.

| Plot | Psychological distress domain | k | *z* | *p* |
| --- | --- | --- | --- | --- |
| 1. | Depression | 94 | 1·7524 | 0·0797 |
| 2. | Depression (outlier removed) | 93 | 1·9156 | 0·0554 |
| 3. | Anxiety | 26 | 0·0374 | 0·9702 |
| 4. | Stress | 5 | 0·2126 | 0·8317 |
| 5. | Blues | 3 | 1·893 | 0·0584 |
| *Note*: *k* = number of studies. | | | | |

**Funnel Plots**

Funnel plots were also visually inspected for indication of publication bias. Below are the five funnel plots generated for Depression, Depression with extreme estimate removed, Anxiety, Stress, and Postnatal Blues. Consistent with the results of Egger’s tests, the symmetrical nature of the funnel plots did not provide evidence of publication bias .

eFigure 20. Funnel plot of effects of associations between depression and postnatal bonding.


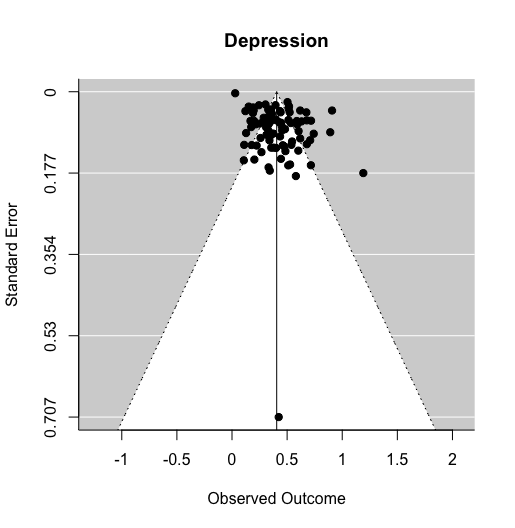


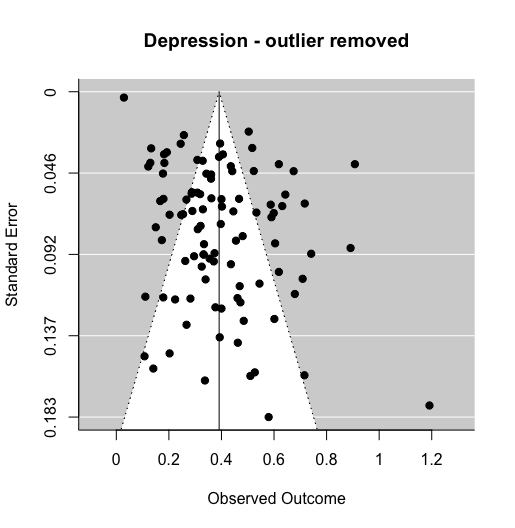
eFigure 21. Funnel plot of effects of associations between depression and postnatal bonding (outlier removed).

eFigure 22. Funnel plot of effects of associations between anxiety and postnatal bonding.


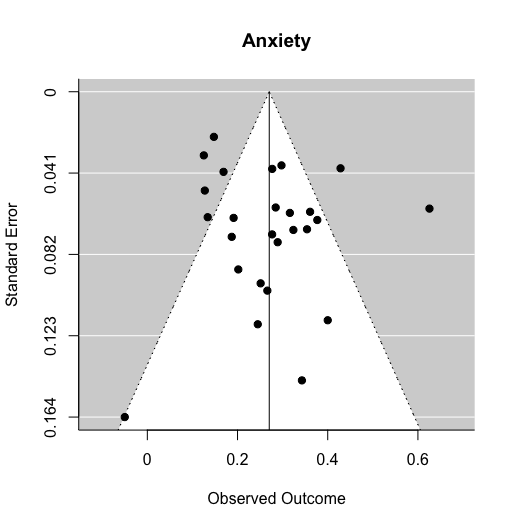


eFigure 23. Funnel plot of effects of associations between stress and postnatal bonding.


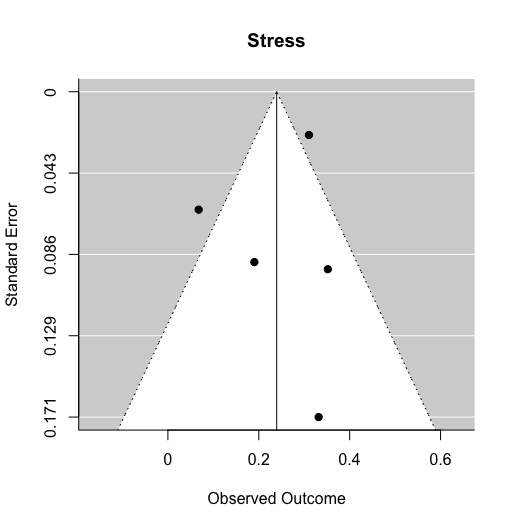


eFigure 24. Funnel plot of effects of associations between postnatal blues and postnatal bonding.


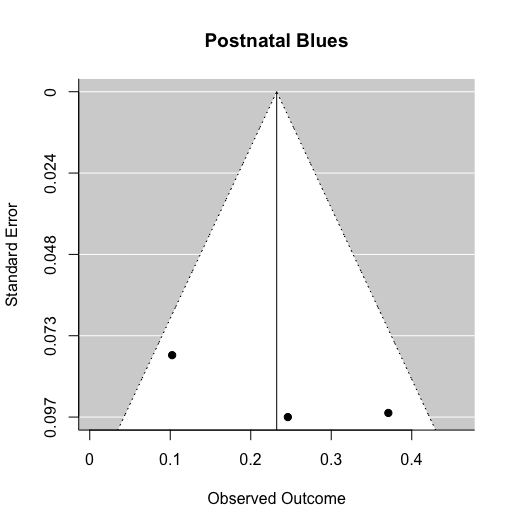


**Moderation analyses for published and grey literature.**

A series of meta-regression analyses was conducted to examine whether there were differences in the magnitude of effect sizes from published studies versus unpublished studies across each psychological distress domain. The results are presented in eTable 12 below and show that publication type had no effect on meta-analytic effects of associations of psychological distress with mother-infant bonding.

eTable 12. Moderation analysis for type of article (published or grey literature).

|  | *k* | *n* | N | *r* | 95% CIs | I^2^ | Tau^2^ | *p* |
| --- | --- | --- | --- | --- | --- | --- | --- | --- |
| All psychological distress domains | |  |  |  |  |  |  |  |
| Published | 88 | 327 | 109726 | 0·38 | (0·34, 0·41) | 97·55 | 0·08 | ·831 |
| Grey | 13 | 41 | 1242 | 0·37 | (0·28, 0·45) | 65·67 | 0·02 |  |
| Depression |  |  |  |  |  |  |  |  |
| Published | 81 | 212 | 108703 | 0·39 | (0·35, 0·43) | 97·72 | 0·08 | ·731 |
| Grey | 13 | 22 | 1254 | 0·38 | (0·29, 0·46) | 60·57 | 0·02 |  |
| Anxiety |  |  |  |  |  |  |  |  |
| Published | 23 | 63 | 7894 | 0·26 | (0·21, 0·31) | 84·33 | 0·02 | ·323 |
| Grey | 3 | 11 | 441 | 0·30 | (0·21, 0·38) | 38·78 | 0·003 |  |
| Stress |  |  |  |  |  |  |  |  |
| Published | 2 | 11 | 1706 | 0·25 | (-0·47, 0·77) | 94·71 | 0·09 | ·916 |
| Grey | 3 | 8 | 362 | 0·23 | (-0·22, 0·60) | 76·89 | 0·04 |  |
| Blues |  |  |  |  |  |  |  |  |
| Published | NA |  |  |  |  |  |  |  |
| Grey | NA |  |  |  |  |  |  |  |
| *Note*: *k* = number of studies; *n* = number of effects; N = combined number of participants in included studies; NA = insufficient effect sizes to meta-analyse; *p* values indicate formal test of difference in magnitude of effect sizes of published and grey literature. | | | | | | | | |

eReferences: Reference list of included papers.

1. Agbagwara-Osuji BO. *Antenatal predictors of early mother-to-infant bonding failure: a prospective cohort study*. United Kingdom, University of London, King's College (United Kingdom); 2015.

2. Badr LK, Ayvazian N, Lameh S, Charafeddine L. Is the Effect of Postpartum Depression on Mother-Infant Bonding Universal? *Infant Behavior & Development.* 2018;51:15-23.

3. Behrendt HF, Konrad K, Goecke TW, Fakhrabadi R, Herpertz-Dahlmann B, Firk C. Postnatal Mother-to-Infant Attachment in Subclinically Depressed Mothers: Dyads at Risk? *Psychopathology.* 2016;49(4):269-276.

4. Behrendt HF, Scharke W, Herpertz-Dahlmann B, Konrad K, Firk C. Like mother, like child? Maternal determinants of children's early social-emotional development. *Infant Mental Health Journal.* 2019;40(2):234-247.

5. Bicking Kinsey C, Baptiste-Roberts K, Junjia Z, Kjerulff KH. Birth-related, psychosocial, and emotional correlates of positive maternal--infant bonding in a cohort of first-time mothers. *Midwifery.* 2014;30(5):e188-194.

6. Bienfait M, Maury M, Haquet A, et al. Pertinence of the self-report mother-to-infant bonding scale in the neonatal unit of a maternity ward. *Early Human Development.* 2011;87(4):281-287.

7. Bonacquisti A, Geller PA, Patterson CA. Maternal depression, anxiety, stress, and maternal-infant attachment in the neonatal intensive care unit. *Journal of Reproductive & Infant Psychology.* 2020;38(3):297-310.

8. Borji M, Shahbazi F, Nariman S, Otaghi M, Safari S. Investigating the relationship between mother-child bonding and maternal mental health. *Journal of Comprehensive Pediatrics.* 2018;9(1).

9. Borschmann R, Molyneaux E, Spry E, et al. Pre-conception self-harm, maternal mental health and mother–infant bonding problems: a 20-year prospective cohort study. *Psychological Medicine.* 2019;49(16):2727-2735.

10. Busonera A, Cataudella S, Lampis J, Tommasi M, Zavattini GC. Psychometric properties of the Postpartum Bonding Questionnaire and correlates of mother–infant bonding impairment in Italian new mothers. *Midwifery.* 2017;55:15-22.

11. Carter E. *Maternal attachment as a predictor of depression among pregnant and postpartum Latina women*. California, US: Pacific Graduate School of Psychology, Palo Alto University; 2015.

12. Chenery A. *Postnatal sleep and its impact on first-time mothers*. United Kingdom: Clinical Psychology, University of Sheffield; 2013.

13. Choi KW, Sikkema KJ, Vythilingum B, et al. Maternal childhood trauma, postpartum depression, and infant outcomes: Avoidant affective processing as a potential mechanism. *Journal of Affective Disorders.* 2017;211:107-115.

14. Chrzan-Dętkoś M, Łockiewicz M. Maternal romantic attachment, and antenatal and postnatal mother–infant attachment in a sample of polish women. *European Journal of Developmental Psychology.* 2015;12(4):429-442.

15. Condon JT, Corkindale CJ. The assessment of parent-to-infant attachment: development of a self-report questionnaire instrument. *Journal of Reproductive and Infant Psychology.* 1998;16(1):57-76.

16. Cuijlits I, van de Wetering AP, Endendijk JJ, van Baar AL, Potharst ES, Pop VJM. Risk and protective factors for pre- and postnatal bonding. *Infant Mental Health Journal.* 2019;40(6):768-785.

17. Cuijlits I, Van deWetering A, Truijens S, Pop V. Development of the Pre-and Postnatal Bonding Scale (PPBS). *Archives of Women's Mental Health.* 2020;23(2):283-284.

18. Daglar G, Nur N. Level of mother-baby bonding and influencing factors during pregnancy and postpartum period. *Psychiatria Danubina.* 2018;30(4):433-440.

19. Damato EG. Prenatal attachment and other correlates of postnatal maternal attachment to twins. *Advances In Neonatal Care: Official Journal Of The National Association Of Neonatal Nurses.* 2004;4(5):274-291.

20. Dubber S, Reck C, Müller M, Gawlik S. Postpartum bonding: the role of perinatal depression, anxiety and maternal–fetal bonding during pregnancy. *Archives of Women's Mental Health.* 2015;18(2):187-195.

21. Edhborg M, Hogg B, Hashima EN, Nahar Kabir Z. Impact of postnatal maternal depressive symptoms and infant's sex on mother-infant interaction among Bangladeshi women. *Health.* 2013;5(2):237-244.

22. Edhborg M, Matthiesen A, Lundh W, Widström A. Some early indicators for depressive symptoms and bonding 2 months postpartum -- a study of new mothers and fathers. *Archives of Women's Mental Health.* 2005;8(4):221-231.

23. Edhborg M, Nasreen H-E, Kabir Z. Impact of postpartum depressive and anxiety symptoms on mothers' emotional tie to their infants 2-3 months postpartum: a population-based study from rural Bangladesh. *Archives of Women's Mental Health.* 2011;14(4):307-316.

24. Faisal-Cury A, Bertazzi Levy R, Kontos A, Tabb K, Matijasevich A. Postpartum bonding at the beginning of the second year of child’s life: the role of postpartum depression and early bonding impairment. *Journal of Psychosomatic Obstetrics and Gynecology.* 2020;41(3):224-230.

25. Fallon V, Silverio SA, Halford JCG, Bennett KM, Harrold JA. Postpartum-specific anxiety and maternal bonding: Further evidence to support the use of childbearing specific mood tools. *Journal of Reproductive and Infant Psychology.* 2019.

26. Farré-Sender B, Torres A, Gelabert E, et al. Mother–infant bonding in the postpartum period: assessment of the impact of pre-delivery factors in a clinical sample. *Archives of Women's Mental Health.* 2018;21(3):287-297.

27. Figueiredo B, Costa R. Mother's stress, mood and emotional involvement with the infant: 3 months before and 3 months after childbirth. *Archives of Women's Mental Health.* 2009;12(3):143-153.

28. Figueiredo B, Costa R, Pacheco A, Pais A. Mother-to-infant emotional involvement at birth. *Maternal And Child Health Journal.* 2009;13(4):539-549.

29. Fijałkowska D, Bielawska-Batorowicz E. A longitudinal study of parental attachment: pre- and postnatal study with couples. *Journal of Reproductive and Infant Psychology.* 2019.

30. Fleming AS, Ruble DN, Flett GL, Shaul DL. Postpartum adjustment in first-time mothers: Relations between mood, maternal attitudes, and mother-infant interactions. *Developmental Psychology.* 1988;24(1):71-81.

31. Fransson E, Sörensen F, Kunovac Kallak T, et al. Maternal perinatal depressive symptoms trajectories and impact on toddler behavior—The importance of symptom duration and maternal bonding. *Journal of Affective Disorders.* 2020;273:542-551.

32. Friedman KB. *Are symptoms of postpartum depression associated with deficits in facial and auditory emotional recognition?* US, Drexel University; 2008.

33. Garcia-Esteve L, Torres A, Lasheras G, et al. Assessment of psychometric properties of the Postpartum Bonding Questionnaire (PBQ) in Spanish mothers. *Archives of Women's Mental Health.* 2016;19(2):385-394.

34. Gashe C. *Infant temperament, maternal attributions, mood and rumination, in predicting maternal problem-solving and mother-infant bonding in the postnatal period*. United Kingdom, University of Exeter; 2011.

35. Hairston IS, Handelzalts JE, Assis C, Kovo M. Postpartum bonding difficulties and adult attachment styles: the mediating role of postpartum depression and childbirth-related PTSD. *Infant Mental Health Journal.* 2018;39(2):198-208.

36. Hairston IS, Handelzalts JE, Lehman-Inbar T, Kovo M. Mother-infant bonding is not associated with feeding type: A community study sample. *BMC Pregnancy and Childbirth.* 2019;19(1).

37. Hairston IS, Solnik-Menilo T, Deviri D, Handelzalts JE. Maternal depressed mood moderates the impact of infant sleep on mother-infant bonding. *Archives Of Women's Mental Health.* 2016;19(6):1029-1039.

38. Hairston IS, Waxler E, Seng JS, Fezzey AG, Rosenblum KL, Muzik M. The role of infant sleep in intergenerational transmission of trauma. *Sleep.* 2011;34(10):1373-1383.

39. Handelzalts JE, Hairston IS, Muzik M, Matatyahu Tahar A, Levy S. A paradoxical role of childbirth-related posttraumatic stress disorder (PTSD) symptoms in the association between personality factors and mother–infant bonding: A cross-sectional study. *Psychological Trauma: Theory, Research, Practice, and Policy.* 2019.

40. Herguner S, Cicek E, Annagur A, Herguner A, Ors R. Association of delivery type with postpartum depression, perceived social support and maternal attachment. *Dusunen Adam: The Journal of Psychiatry and Neurological Sciences* 2014;27(1):15-20.

41. Hiroko S, Yamashita H, Yoshida K. Establishment of community-based mental healthcare. Archives of Women's Mental Health; 2020.

42. Høivik MS, Burkeland NA, Linaker OM, Berg-Nielsen TS. The Mother and Baby Interaction Scale: a valid broadband instrument for efficient screening of postpartum interaction? A preliminary validation in a Norwegian community sample. *Scandinavian Journal of Caring Sciences.* 2013;27(3):733-739.

43. Hrishikesh BN. Exploring socio-obstetric and nutritional determinants of maternal bonding failure in women with postpartum depression-a cross sectional study from a tertiary care center. *Indian Journal of Psychiatry.* 2019;61(9):S358.

44. Jones G, Morrell C, Cooke J, Speier D, Anumba D, Stewart-Brown S. The development of two postnatal health instruments: one for mothers (M-PHI) and one for fathers (F-PHI) to measure health during the first year of parenting. *Quality of Life Research.* 2011;20(7):1011-1022.

45. Jordan B, Franich-Ray C, Albert N, et al. Early mother-infant relationships after cardiac surgery in infancy. *Archives of Disease in Childhood: Education and Practice Edition.* 2014;99(7):641-645.

46. Kaneko H, Honjo S. The psychometric properties and factor structure of the Postpartum Bonding Questionnaire in Japanese mothers. *Psychology.* 2014;5(9):1135-1142.

47. Kasamatsu H, Tsuchida A, Matsumura K, Shimao M, Hamazaki K, Inadera H. Understanding the relationship between postpartum depression one month and six months after delivery and mother-infant bonding failure one-year after birth: results from the Japan Environment and Children's study (JECS). *Psychological Medicine.* 2020;50(1):161-169.

48. Kerstis B, Aarts C, Tillman C, et al. Association between parental depressive symptoms and impaired bonding with the infant. *Archives of Women's Mental Health.* 2016;19(1):87-94.

49. Kita S, Haruna M, Matsuzaki M, Kamibeppu K. Associations between intimate partner violence (IPV) during pregnancy, mother-to-infant bonding failure, and postnatal depressive symptoms. *Archives of Women's Mental Health.* 2016;19(4):623-634.

50. Kita S, Haruna M, Matsuzaki M, Kamibeppu K. Does Antenatal Social Support Affect the Relationships Between Intimate Partner Violence During Pregnancy And Perinatal Mental Health? *Violence Against Women.* 2020;26(6/7):573-589.

51. Kleiber BV. *Postpartum depression among adolescent mothers: Examining and treating low-income adolescents with symptoms of postpartum depression*. US: Department of Psychology and Neuroscience, University of Colorado; 2015.

52. Kokubu M, Okano A, Sugiyama T. Postnatal depression, maternal bonding failure, and negative attitudes towards pregnancy: A longitudinal study of pregnant women in Japan. *Archives of Women's Mental Health.* 2012;15(3):211-216.

53. Lara-Cinisomo S, Zhu K, Fei K, Bu Y, Weston AP, Ravat U. Traumatic events: Exploring associations with maternal depression, infant bonding, and oxytocin in Latina mothers. *BMC Women's Health.* 2018;18(1).

54. Le Bas GA, Youssef GJ, Macdonald JA, et al. Maternal bonding, negative affect, and infant social-emotional development: A prospective cohort study. *Journal of Affective Disorders.* 2020.

55. Leahy-Warren P, Coleman C, Bradley R, Mulcahy H. The experiences of mothers with preterm infants within the first-year post discharge from NICU: Social support, attachment and level of depressive symptoms. *BMC Pregnancy and Childbirth.* 2020;20(1).

56. Lehnig F, Nagl M, Stepan H, Wagner B, Kersting A. Associations of postpartum mother-infant bonding with maternal childhood maltreatment and postpartum mental health: A cross-sectional study. *BMC Pregnancy and Childbirth.* 2019;19(1).

57. Leserman J, Stansbury M, Garcia N, Pedersen C. Trauma history is related to oxytocin and maternal-infant bonding. Psychosomatic Medicine; 2011.

58. Loh C-C, Vostanis P. Perceived mother-infant relationship difficulties in postnatal depression. *Infant and Child Development.* 2004;13(2):159-171.

59. Lutkiewicz K, Bieleninik Ł, Cieślak M, Bidzan M. Maternal–infant bonding and its relationships with maternal depressive symptoms, stress and anxiety in the early postpartum period in a polish sample. *International Journal of Environmental Research and Public Health.* 2020;17(15):1-12.

60. Luz R, George A, Vieux R, Spitz E. Antenatal determinants of parental attachment and parenting alliance: how do mothers and fathers differ? *Infant Mental Health Journal.* 2017;38(2):183-197.

61. Macdonald JA, Greenwood C, Letcher P, et al. From adolescence to parenthood: a multi-decade study of preconception mental health problems and postpartum parent–infant bonds. *Social Psychiatry and Psychiatric Epidemiology.* 2020:1-10.

62. Mackie FL, Pattison H, Jankovic J, Morris RK, Kilby MD. Parental attachment and depressive symptoms in pregnancies complicated by twin-twin transfusion syndrome: a cohort study. *BMC pregnancy and childbirth.* 2020;20(1):1-8.

63. Martini J, Beesdo-Baum K, Garthus-Niegel S, Wittchen HU. The course of panic disorder during the peripartum period and the risk for adverse child development: A prospective-longitudinal study. *Journal of Affective Disorders.* 2020;266:722-730.

64. Mason ZS, Briggs RD, Silver EJ. Maternal attachment feelings mediate between maternal reports of depression, infant social–emotional development, and parenting stress. *Journal of Reproductive & Infant Psychology.* 2011;29(4):382-394.

65. Matthies LM, Müller M, Doster A, et al. Maternal-fetal attachment protects against postpartum anxiety: the mediating role of postpartum bonding and partnership satisfaction. *Archives of Gynecology & Obstetrics.* 2020;301(1):107-117.

66. McErlean RA. *Oxytocin and maternal behaviour in the early postpartum*

*A randomised, placebo‐controlled trial of oxytocin nasal spray upon maternal bonding* [Doctoral thesis]: School of Psychology, Faculty of Science, University of New South Wales, Sydney, Australia; 2012.

67. Mercer RT, Ferketich SL. Predictors of parental attachment during early parenthood. *Journal of Advanced Nursing.* 1990;15(3):268-280.

68. Mercer RT, Ferketich SL. Maternal-infant attachment of experienced and inexperienced mothers during infancy. *Nursing Research.* 1994;43(6):344-351.

69. Milne LC, Greenway P, Hansen L. Predictors of postnatal depression in a community sample. *Neonatal, Paediatric & Child Health Nursing.* 2007;10(1):20-26.

70. Minamida T, Iseki A, Sakai H, Imura M, Okano T, Tanii H. Do postpartum anxiety and breastfeeding self-efficacy and bonding at early postpartum predict postpartum depression and the breastfeeding method? *Infant Mental Health Journal.* 2020;41(5):662-676.

71. Moehler E, Brunner R, Wiebel A, Reck C, Resch F. Maternal depressive symptoms in the postnatal period are associated with long-term impairment of mother-child bonding. *Archives of Women's Mental Health.* 2006;9(5):273-278.

72. Morrison CI. *Trauma, depression, early attachment, and neonatal health outcomes among African American mothers and their infants.* US, University of Maryland; 2018.

73. Moser MH. *The effects of infant temperament, maternal stress, and maternal employment on maternal attachment to the infant and infant attachment to the mother*. Ann Arbor, George Mason University; 1989.

74. Müller D, Teismann T, Havemann B, Michalak J, Seehagen S. Ruminative thinking as a predictor of perceived postpartum mother–infant bonding. *Cognitive Therapy and Research.* 2013;37(1):89-96.

75. Muzik M, Bocknek EL, Broderick A, et al. Mother–infant bonding impairment across the first 6 months postpartum: The primacy of psychopathology in women with childhood abuse and neglect histories. *Archives of Women's Mental Health.* 2013;16(1):29-38.

76. Muzik M, Morelen D, Hruschak J, Rosenblum KL, Bocknek E, Beeghly M. Psychopathology and parenting: An examination of perceived and observed parenting in mothers with depression and PTSD. *Journal of Affective Disorders.* 2017;207:242-250.

77. Myers S. *Maternal investment and postnatal depression: an evolutionary approach*. Ann Arbor, University of Kent at Canterbury (United Kingdom); 2017.

78. Nagata M, Nagai Y, Sobajima H, Ando T, Honjo S. Depression in the early postpartum period and attachment to children--in mothers of NICU infants. *Infant and Child Development.* 2004;13(2):93-110.

79. Nagata M, Nagai Y, Sobajima H, Ando T, Nishide Y, Honjo S. Maternity blues and attachment to children in mothers of full-term normal infants. *Acta Psychiatrica Scandinavica.* 2000;101(3):209-217.

80. Nakano M, Upadhyaya S, Chudal R, et al. Risk factors for impaired maternal bonding when infants are 3 months old: A longitudinal population based study from Japan. *BMC Psychiatry.* 2019;19(1).

81. Nakash O, Nagar M, Lurie I. The Association Between Postnatal Depression, Acculturation and Mother-Infant Bond Among Eritrean Asylum Seekers in Israel. *Journal Of Immigrant And Minority Health.* 2016;18(5):1232-1236.

82. Nath S, Pearson RM, Moran P, et al. The association between prenatal maternal anxiety disorders and postpartum perceived and observed mother-infant relationship quality. *Journal of Anxiety Disorders.* 2019;68.

83. Ngoma AM, Goto A, Suzuki Y, Tsutomi H, Yasumura S. Support-seeking behavior among Japanese mothers at high-risk of mental health problems: a community-based study at a city health center. *Fukushima Journal Of Medical Science.* 2012;58(2):117-126.

84. Nolvi S, Karlsson L, Bridgett DJ, Pajulo M, Tolvanen M, Karlsson H. Maternal postnatal psychiatric symptoms and infant temperament affect early mother-infant bonding. *Infant Behavior and Development.* 2016;43:13-23.

85. Nonnenmacher N, Noe D, Ehrenthal JC, Reck C. Postpartum bonding: the impact of maternal depression and adult attachment style. *Archives of Women's Mental Health.* 2016;19(5):927-935.

86. Noyman-Veksler G, Herishanu-Gilutz S, Kofman O, Holchberg G, Shahar G. Post-natal psychopathology and bonding with the infant among first-time mothers undergoing a caesarian section and vaginal delivery: Sense of coherence and social support as moderators. *Psychology & Health.* 2015;30(4):441-455.

87. O'Higgins M, Roberts I, Glover V, Taylor A. Mother-child bonding at 1 year; associations with symptoms of postnatal depression and bonding in the first few weeks. *Archives of Women's Mental Health.* 2013;16(5):381-389.

88. Oddo-Sommerfeld S, Hain S, Louwen F, Schermelleh-Engel K. Longitudinal effects of dysfunctional perfectionism and avoidant personality style on postpartum mental disorders: Pathways through antepartum depression and anxiety. *Journal of Affective Disorders.* 2016;191:280-288.

89. Ohara M, Nakatochi M, Okada T, et al. Impact of perceived rearing and social support on bonding failure and depression among mothers: A longitudinal study of pregnant women. *Journal of Psychiatric Research.* 2018;105:71-77.

90. Ohara M, Okada T, Aleksic B, et al. Social support helps protect against perinatal bonding failure and depression among mothers: a prospective cohort study. *Scientific Reports.* 2017;7(1):9546-9546.

91. Ohara M, Okada T, Kubota C, et al. Relationship between maternal depression and bonding failure: A prospective cohort study of pregnant women. *Psychiatry and Clinical Neurosciences.* 2017;71(10):733-741.

92. Ohara M, Okada T, Kubota C, et al. Validation and factor analysis of mother-infant bonding questionnaire in pregnant and postpartum women in Japan. *BMC Psychiatry.* 2016;16(1).

93. Ohashi Y, Kitamura T, Sakanashi K, Tanaka T. Postpartum Bonding Disorder: Factor Structure, Validity, Reliability and a Model Comparison of the Postnatal Bonding Questionnaire in Japanese Mothers of Infants. *Healthcare.* 2016;4(3).

94. Ohoka H, Koide T, Goto S, et al. Effects of maternal depressive symptomatology during pregnancy and the postpartum period on infant–mother attachment. *Psychiatry and Clinical Neurosciences.* 2014;68(8):631-639.

95. Olsson CA, Spry EA, Alway Y, et al. Preconception depression and anxiety symptoms and maternal-infant bonding: a 20-year intergenerational cohort study. *Archives of women's mental health.* 2020:1-11.

96. Örün E, Yalçin SS, Mutlu B. Relations of maternal psychopathologies, social-obstetrical factors and mother-infant bonding at 2-month postpartum: A sample of Turkish mothers. *World Journal of Pediatrics.* 2013;9(4):350-355.

97. Oskovi-Kaplan ZA, Buyuk GN, Ozgu-Erdinc AS, Keskin HL, Ozbas A, Moraloglu Tekin O. The Effect of COVID-19 Pandemic and Social Restrictions on Depression Rates and Maternal Attachment in Immediate Postpartum Women: a Preliminary Study. *Psychiatric Quarterly.* 2020.

98. Parfitt Y, Ayers S, Pike A, Jessop DC, Ford E. A prospective study of the parent–baby bond in men and women 15 months after birth. *Journal of Reproductive and Infant Psychology.* 2014;32(5):441-456.

99. Pearson RM, Lightman SL, Evans J. Attentional processing of infant emotion during late pregnancy and mother-infant relations after birth. *Archives of Women's Mental Health.* 2011;14(1):23-31.

100. Petri E, Palagini L, Bacci O, et al. Maternal-foetal attachment independently predicts the quality of maternal-infant bonding and post-partum psychopathology. *Journal Of Maternal-Fetal & Neonatal Medicine.* 2017:1-7.

101. Radoš SN, Matijaš M, Anđelinović M, Čartolovni A, Ayers S. The role of posttraumatic stress and depression symptoms in mother-infant bonding. *Journal of Affective Disorders.* 2020;268:134-140.

102. Reck C, Klier CM, Pabst K, et al. The German version of the postpartum bonding instrument: Psychometric properties and association with postpartum depression. *Archives of Women's Mental Health.* 2006;9(5):265-271.

103. Reck C, Zietlow AL, Müller M, Dubber S. Perceived parenting stress in the course of postpartum depression: the buffering effect of maternal bonding. *Archives of Women's Mental Health.* 2015:1-10.

104. Riera-Martín A, Oliver-Roig A, Martínez-Pampliega A, Cormenzana-Redondo S, Clement-Carbonell V, Richart-Martínez M. A single spanish version of maternal and paternal postnatal attachment scales: Validation and conceptual analysis. *PeerJ.* 2018(11).

105. Robakis T, Williams K, Crowe S, Kenna H, Gannon J, Rasgon N. Optimistic outlook regarding maternity protects against depressive symptoms postpartum. *Archives of Women's Mental Health.* 2015;18(2):197-208.

106. Rossen L, Hutchinson D, Wilson J, et al. Predictors of postnatal mother-infant bonding: the role of antenatal bonding, maternal substance use and mental health. *Archives Of Women's Mental Health.* 2016;19(4):609-622.

107. Rossen L, Mattick RP, Wilson J, et al. Mother–Infant Bonding and Emotional Availability at 12-Months of Age: The Role of Early Postnatal Bonding, Maternal Substance Use and Mental Health. *Maternal & Child Health Journal.* 2019;23(12):1686-1698.

108. Sawyer Cohen J. *Mindfulness and self-compassion in the transition to motherhood: A prospective study of postnatal mood and attachment* [Doctoral thesis]. US: Graduate School of Arts and Sciences, Columbia University; 2010.

109. Schmidt D, Seehagen S, Hirschfeld G, Vocks S, Schneider S, Teismann T. Repetitive Negative Thinking and Impaired Mother–Infant Bonding: A Longitudinal Study. *Cognitive Therapy and Research.* 2017;41(3):498-507.

110. Scopesi A, Viterbori P, Sponza S, Zucchinetti P. Assessing mother‐to‐infant attachment: the Italian adaptation of a self‐report questionnaire. *Journal of reproductive and infant psychology.* 2004;22(2):99-109.

111. Seng JS, Sperlich M, Low LK, Ronis DL, Muzik M, Liberzon I. Childhood Abuse History, Posttraumatic Stress Disorder, Postpartum Mental Health, and Bonding: A Prospective Cohort Study. *Journal of Midwifery & Women's Health.* 2013;58(1):57-68.

112. Sockol L, Battle C, Howard M, Davis T. Correlates of impaired mother-infant bonding in a partial hospital program for perinatal women. *Archives of Women's Mental Health.* 2014;17(5):465-469.

113. Suetsugu Y, Haruna M, Kamibeppu K. A longitudinal study of bonding failure related to aspects of posttraumatic stress symptoms after childbirth among Japanese mothers. *BMC Pregnancy and Childbirth.* 2020;20(1).

114. Suetsugu Y, Honjo S, Ikeda M, Kamibeppu K. The Japanese version of the Postpartum Bonding Questionnaire: Examination of the reliability, validity, and scale structure. *Journal of Psychosomatic Research.* 2015;79(1):55-61.

115. Sun-Hee K. Factors associated with Maternal Attachment of Breastfeeding Mothers. *Child Health Nursing Research.* 2019;25(1):65-73.

116. Talmon A, Horovitz M, Shabat N, Haramati OS, Ginzburg K. “Neglected moms” - The implications of emotional neglect in childhood for the transition to motherhood. *Child Abuse and Neglect.* 2019;88:445-454.

117. Talmon A, Shaham Salomon N, Ginzburg K. Differentiation of the self and the body and adjustment to motherhood – A latent class analysis. *Journal of Affective Disorders.* 2020;276:287-296.

118. Taylor A, Atkins R, Kumar R, Adams D, Glover V. A new mother-to-infant bonding scale: Links with early maternal mood. *Archives of Women's Mental Health.* 2005;8(1):45-51.

119. Tester-Jones M, O'Mahen H, Watkins E, Karl A. The impact of maternal characteristics, infant temperament and contextual factors on maternal responsiveness to infant. *Infant Behavior and Development.* 2015;40:1-11.

120. Tietz A, Zietlow AL, Reck C. Maternal bonding in mothers with postpartum anxiety disorder: the crucial role of subclinical depressive symptoms and maternal avoidance behaviour. *Archives of Women's Mental Health.* 2014.

121. Tikotzky L. Postpartum maternal sleep, maternal depressive symptoms and self-perceived mother–infant emotional relationship. *Behavioral Sleep Medicine.* 2016;14(1):5-22.

122. Tolja R, Nakić Radoš S, Anđelinović M. The role of maternal mental health, infant temperament, and couple's relationship quality for mother-infant bonding. *Journal of Reproductive & Infant Psychology.* 2020;38(4):395-407.

123. Tsuchida A, Hamazaki K, Matsumura K, et al. Changes in the association between postpartum depression and mother-infant bonding by parity: Longitudinal results from the Japan Environment and Children's Study. *Journal of Psychiatric Research.* 2019;110:110-116.

124. Van Bussel JCH, Spitz B, Demyttenaere K. Three self-report questionnaires of the early mother-to-infant bond: reliability and validity of the Dutch version of the MPAS, PBQ and MIBS. *Archives of women's mental health.* 2010;13(5):373-384.

125. Vengadavaradan A, Bharadwaj B, Sathynarayanan G, Durairaj J, Rajaa S. Translation, validation and factor structure of the Tamil version of the Postpartum Bonding Questionnaire (PBQ-T). *Asian Journal of Psychiatry.* 2019;40:62-67.

126. Vreeswijk C, Maas J, Van Bakel H. Mothers' emotions during pregnancy in relation to the antenatal and postnatal mother-infant relationship. Paper presented at: Journal of Reproductive and Infant Psychology2011.

127. Wikman A, Axfors C, Iliadis SI, Cox J, Fransson E, Skalkidou A. Characteristics of women with different perinatal depression trajectories. *Journal of Neuroscience Research.* 2020;98(7):1268-1282.

128. Williams C, Taylor EP, Schwannauer M. A web‐based survey of mother–infant bond, attachment experiences, and metacognition in posttraumatic stress following childbirth. *Infant Mental Health Journal.* 2016;37(3):259-273.

129. Wittkowski A, Wieck A, Mann S. An evaluation of two bonding questionnaires: a comparison of the Mother-to-Infant Bonding Scale with the Postpartum Bonding Questionnaire in a sample of primiparous mothers. *Archives of Women's Mental Health.* 2007;10(4):171-175.

130. Wittkowski A, Williams J, Wieck A. An examination of the psychometric properties and factor structure of the Post-partum Bonding Questionnaire in a clinical inpatient sample. *British Journal of Clinical Psychology.* 2010;49(2):163-172.

131. Yoshida K, Yamashita H, Conroy S, Marks M, Kumar C. A Japanese version of mother-to-infant bonding scale: Factor structure, longitudinal changes and links with maternal mood during the early postnatal period in Japanese mothers. *Archives of Women's Mental Health.* 2012;15(5):343-352.

132. Zeitlin D, Dhanjal T, Colmsee M. Maternal-foetal bonding: The impact of domestic violence on the bonding process between a mother and child. *Archives of Women's Mental Health.* 1999;2(4):183-189.

133. Zhang H, Su Q, Yao D, et al. Prolactin, a potential mediator of reduced social interactive behavior in newborn infants following maternal perinatal depressive symptoms. *Journal of Affective Disorders.* 2017;215:274-280.

1. NHLBI. Study Quality Assessment Tools. 2018. <https://www.nhlbi.nih.gov/health-topics/study-quality-assessment-tools> (accessed 20 August 2018). [↑](#footnote-ref-1)
2. Mackie et al., 2020 [↑](#footnote-ref-2)
